# A mouthpart transcriptome for *Spodoptera frugiperda* adults: identification of candidate chemoreceptors and investigation of expression patterns

**DOI:** 10.3389/fphys.2023.1193085

**Published:** 2023-04-25

**Authors:** Jun-Feng Dong, Zhen-Jie Hu, Bing-Xin Dong, Cai-Hong Tian

**Affiliations:** ^1^ College of Horticulture and Plant Protection, Henan University of Science and Technology, Luoyang, Henan Province, China; ^2^ Institute of Plant Protection, Henan Academy of Agricultural Sciences, Zhengzhou, Henan Province, China

**Keywords:** chemoreceptor, transcriptome, mouthpart, expression profiling, *Spodoptera frugiperda*

## Abstract

Moth mouthparts, consisting of labial palps and proboscis, not only are the feeding device but also are chemosensory organs for the detection of chemical signals from surrounding environment. Up to now, the chemosensory systems in the mouthpart of moths are largely unknown. Here, we performed systematic analyses of the mouthpart transcriptome of adult *Spodoptera frugiperda* (Lepidoptera: Noctuidae), a notorious pest that spreads worldwide. A total of 48 chemoreceptors, including 29 odorant receptors (ORs), 9 gustatory receptors (GRs), and 10 ionotropic receptors (IRs), were annotated. Further phylogenetic analyses with these genes and homologs from other insect species determined that specific genes, including ORco, carbon dioxide receptors, pheromone receptor, IR co-receptors, and sugar receptors, were transcribed in the mouthpart of *S. frugiperda* adults. Subsequently, expression profiling in different chemosensory tissues demonstrated that the annotated *ORs* and *IRs* were mainly expressed in *S. frugiperda* antennae, but one *IR* was also highly expressed in the mouthparts. In comparison, *SfruGR*s were mainly expressed in the mouthparts, but 3 GRs were also highly expressed in the antennae or the legs. Further comparison of the mouthpart-biased chemoreceptors using RT-qPCR revealed that the expression of these genes varied significantly between labial palps and proboscises. This study provides the first large-scale description of chemoreceptors in the mouthpart of adult *S*. *frugiperda* and provides a foundation for further functional studies of chemoreceptors in the mouthpart of *S*. *frugiperda* as well as of other moth species.

## Introduction

Insect head is the primary center for the communication with surrounding environment. Most insects possess two cephalic sensory organs, i.e., antenna and mouthpart. These two organs play vital roles during the life cycle of insects, such as locating host plants, feeding, and searching mates (Hansson and Stensmyr, 2011; Leal, 2013). Antennae are known to be the most crucial sensory organ of insects, they can perceive various external stimulation, such as odorants, flavors, carbon dioxide, and humidity (Dahanukar et al., 2005). In comparison, mouthparts mainly act as the device for feeding, and they were also demonstrated to have the function in chemosensation (Stocker, 1994; Kaissling, 1996; Krenn, 2010).

In Lepidoptera, most adults have a siphoning mouthpart including a retractable proboscis and a pair of labial palps. Proboscises act as the feeding device, and they consist of two elongated maxillae galeae and a hollow straw, which functions as a system for sucking liquid substances. A variety of chemosensory sensilla are distributed on the surface of the mouthpart. For example, more than 1,200 sensilla were spread on the labial-palp pit organ in *Mythimna separata* (Dong et al., 2014). Three major sensilla types (styloconica, chaetica, and basiconica) were determined on the proboscis of *Helicoverpa armigera* (Guo et al., 2018), and similar findings were reported in *Athetis lepigone* (Hu et al., 2021).

Chemosensory sensilla are porous hair-like structures innervated by the dendrites of chemosensory neurons (CSNs). Three chemoreceptor families, including gustatory receptors (GRs), odorant receptors (ORs), and ionotropic receptors (IRs), are expressed on the dendrite membrane of CSNs (Kaissling, 1996). These receptors can selectively identify chemical molecules and play a central role in the determination of the detection spectrum of chemosensory sensilla (Robertson, 2018; Guo et al., 2022). It is well known that ORs mediate the perception of odorant cues, whereas GRs are responsible for the detection of taste cues. According to the ligand properties, ORs can be classified into pheromone receptor (PRs) and ordinary receptors (Yang and Wang, 2020), whereas GRs can be divided into bitter, sugar, and CO_2_ receptors (Jones et al., 2007; Sato et al., 2011; Miyamoto et al., 2012; Fujii et al., 2015; Xu et al., 2016). Insects IRs were extensively researched in *Drosophila melanogaster*, and their functions mainly include olfaction, gustation, thermosensation, and hygrosensation (Chen et al., 2015; Enjin et al., 2016; Knecht et al., 2016). With the development of research techniques, IRs in moths have also been extensively studied in recent years (Zhang J. et al., 2019; Tang et al., 2020; Hou et al., 2022). Members in IR family are further divided into antennal IRs that exist in most insect species and divergent IRs of which numbers and gene types are different among species (Benton et al., 2009; Liu et al., 2018).

*Spodoptera frugiperda*, also called fall armyworm, is a notorious pest with more than 300 host plant species. It preferred to feed on Poaceae Barnhart species such as rice, maize, and sugarcane (Sparks, 1979). *S*. *frugiperda* is native to Americas. As the adults can undertake seasonal migrations covering long distances, it has spread to Africa and Asia in last 5 years (Goergen et al., 2016; Cock et al., 2017; Wu et al., 2019). *S. frugiperda* invaded Yunnan province of China in 2019 (Zhang L. et al., 2019), and has become an important agricultural pest across China in recent years (Sun et al., 2021). Adults of most Lepidopteran species (including *S. frugiperda*) adopt flower-visiting strategies, and floral nectar is crucial for them to sustain growth and reproduction (Krenn, 2010). Studies using anatomical, neurophysiological, and molecular approaches showed that flower evaluation by *Manduca sexta* is governed by specific CSNs housed in the sensilla on the moth’s proboscis (Haverkamp et al., 2016). However, identities of chemosensory receptors in the mouthpart and their functions in the feeding are still enigmatic for most Lepidoptera.

To reveal potential chemosensory systems of the mouthpart in *S*. *frugiperda*, we systematically investigated the chemoreceptors using Illumina sequencing. The candidate GR, OR, and IR genes were annotated and phylogenetic relationships between these genes and homologs from other insect species were analyzed. Lastly, expression levels of candidate genes in different chemosensory tissues were investigated by RT-qPCR. This work contributes to further functional researches on chemoreceptors in the mouthpart of *S. frugiperda* as well as of other moth species.

## Materials and methods

### Insect rearing

*S. frugiperda* larvae were collected from Baoshan, Yunnan Province, China. The larvae were reared with artificial diet, and moths were fed with 15% (V: V) honey water (Guo et al., 2022). Twenty moths were kept in a plastic bucket (25 cm in diameter, 30 cm in length, the opening side is wrapped with a piece of medical gauze) for mating in a sex ratio of 1: 1. The gauze was collected daily and kept in valve bags until the larvae hatched out. Successive generations were maintained in an incubator under 16 h L: 8 h D cycle at 25°C ± 1°C and 60% relative humidity. For transcriptome sequencing, mouthparts were separately collected from the 2- to 3-day old virgin male and female moths and then kept in −80°C freezer until they were used.

### Transcriptome sequencing, assembly, and gene annotation

Total RNA was isolated from the sample (one biological replicate) using Trizol reagent (Invitrogen, Carlsbad, CA, United States). Concentration of the RNA was measured with an ND-2000 spectrophotometer (Nanodrop, Wilmington, DE, United States). Genomic DNA mixed in the total RNA was eliminated by DNase I (RQ1, Promega, Madison, WI, United States). mRNA was then isolated with Oligo (dT) from 5 µg of the total RNA by Dynabeads mRNA purification kit (Invitrogen, United States). RNA-seq libraries were constructed following the protocol of Illumina’s library. The prepared libraries were then sequenced on the Illumina HiSeq 2000 platform (Illumina, United States) at Sangon Biotech.

*De novo* assembly was performed as we previously described (Sun et al., 2022). Raw reads were processed to remove low quality sequences, adaptors, and reads with microbes (Bolger et al., 2014). The results were verified with The FastQC package. The clean reads were then *de novo* assembled to obtain contigs using Trinity v 2.4.0. As the genome data of *S*. *frugiperda* had been made public (Gouin et al., 2017), a genome-based mapping assembling strategy was also combined to improve the quality of data analysis results.

We used BlastX tool (E-value < 1e-5) to search the Nr, Swiss-Prot, KEGG, and COG databases to retrieve putative GR, OR, and IR transcripts from the contigs. ORFs (open reading frames) of the transcripts encoding putative SfruGRs, SfruORs, and SfruIRs were predicted with ORFfinder (https://www.ncbi.nlm.nih.gov/orffinder), the obtained ORFs were then manually checked through comparing by BlastX. Transcripts Per Kilobase of exon model per Million mapped reads (TPM) values of candidate transcripts were calculated with the RSEM package to evaluate the expression abundance in male or female mouthparts of *S. frugiperda*.

### Phylogenetic analysis of GRs, ORs, and IRs

Phylogenetic trees of candidate chemoreceptors (SfruGRs, SfruORs, and SfruIRs) were built using the neighbor-joining method in MEGA 11 and then edited with FigTree (v1.4.2). The evolutionary distance was calculated with the Jones-Taylor-Thornton matrix-based method (Jones et al., 1992). Node support was evaluated with a bootstrap method of 1,000 replicates. The amino acid sequences of GR, OR, and IR genes used in the phylogenetic analysis are listed in (Supplementary Table S1).

### Expression analysis

Real-time quantitative PCR (RT-qPCR) was conducted to compare the expression levels of candidate chemoreceptors in different tissues of *S. frugiperda*. Male and female antennae, mouthparts, legs, proboscises, and labial palps were collected separately from 50 to 100 individuals. Total RNA of different samples was extracted following the Trizol reagent (Invitrogen, Carlsbad, CA, United States) manual. First strand cDNA was then synthesized using reverse transcriptase (M-MLV, Promega, WI, United States). The cDNA could be directly used in RT-qPCR reaction.

RT-qPCR was performed on Roche LightCycler 480 (F. Hoffmann-La Roche Ltd., Basel, Switzerland). The solution for each reaction (total volume 20 µL) includes a 10-µL of SYBR Premix Ex TaqII (TaKaRa, Dalian, China), 2.5 ng of template cDNA, 0.4 mM of primer (forward or reverse), and right amount of sterilized deionized H_2_O. The reaction condition is: 1 cycle of 95°C for 30 s; 40 cycles of 95°C for 5 s, 60°C for 30 s, and 72°C for 30 s; 1 cycle of 95°C for 45 s, 55°C for 1 min. Reactions for different samples were performed with three biological replicates, and each replicate was performed in triplicate. Expression levels of tested genes in different tissues were calculated using the 2^−ΔΔCT^ method (Schmittgen and Livak, 2008), we use *β*-*actin* as the internal control. CT values for the reactions of the *β*-*actin* gene range from 17.2 to 18.2, this indicates it is consistent across different tissues and suitable for sample normalization. The primer efficiency was evaluated by standard amplification curves constructed with 5-fold dilutions of cDNA samples. The efficiency percentage and *R* ^2^ values were validated within the acceptable range. Ten PCR products were randomly selected and sequenced to ensure that the target genes were amplified. Primers used in the RT-qPCR were designed with Primer Premier v 6.0 (Supplementary Table S2).

### Data analysis

RT-qPCR data were analyzed with ANOVA followed by Tukey’s test (*p* < 0.05). The results are presented as means ± (standard error), data are averages of three replicates. The figures were constructed with GraphPad Prism v 6.0.

## Results

### Transcriptome sequencing, assembly, and identification of candidate chemoreceptors

In the current study, we sequenced the transcriptome of *S*. *frugiperda* mouthpart using the Illumina HiSeq 2000 platform. Clean reads from female and male samples were then combined and generated an assembly of 119,928 unigenes with an N_50_ length of 785 bp and a mean length of 568 bp. Based on the sequence length distribution, 14,295 (11.91%) of the unigenes were ≥1000 bp (Supplementary Table S3).

According to the sequence analysis, we identified a total of 29 ORs in the transcriptome of *S*. *frugiperda* mouthpart. Twenty-seven of these genes possess putative complete ORFs. To keep uniformity, candidate genes in this study, if possible, were named referring to the previously reported sequences of *S*. *frugiperda*, or numbered following the names of the best hit genes in other lepidopteran species. Among the 29 ORs, 8 (SfruOR2/6/18/23/49a/67a/67c/85c) had not been annotated in previous genome or transcriptome studies of this species (Gouin et al., 2017; Sun et al., 2022).

A total of 9 GRs were identified in the transcriptome of *S*. *frugiperda* mouthpart. Except for SfruGR6, all the other annotated GRs possess full length ORFs (Supplementary Tables S4, S5). Among the 9 GRs, SfruGR1/2/3/9 had been previously reported in the head transcriptome of *S*. *frugiperda* larvae (Sun et al., 2022), while the other 5 GRs (SfruGR4/5/6/7/8) had not been reported in previous studies of this species. Moreover, we annotated 10 IRs in the mouthpart of *S*. *frugiperda*. All of these candidate IRs have complete ORFs (Supplementary Tables S4, S5). Based on the Blastx result, SfruIR8a had not been reported in previous studies of *S. frugiperda*. Detailed information for the candidate genes in this study, including names, accession numbers, BlastX best hits, and sequences are listed in Supplementary Tables S4, S5.

### Phylogenetic analysis of candidate chemoreceptors

**FIGURE 1:**
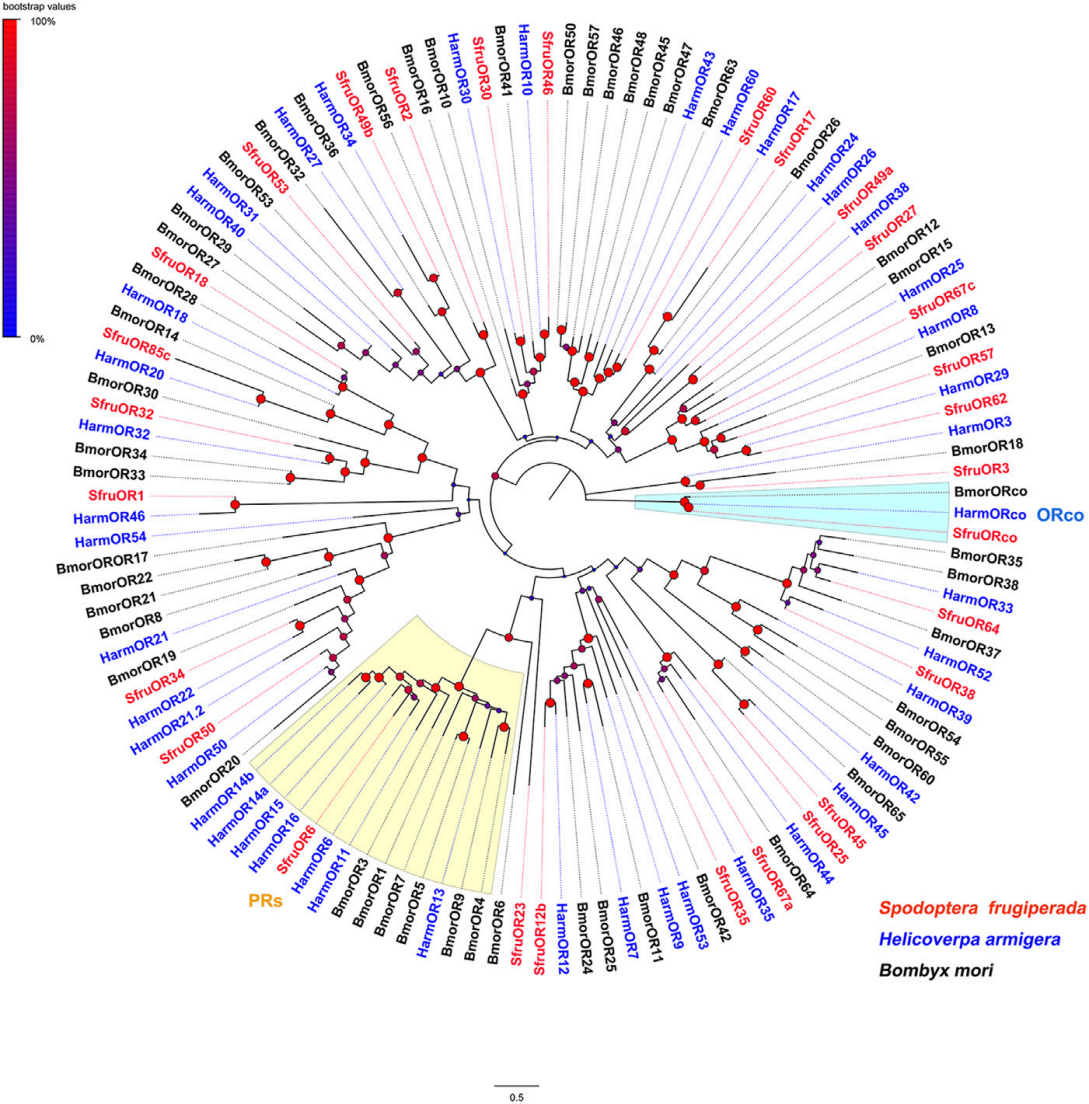
Neighbor-joining tree constructed with candidate ORs from *Spodoptera frugiperda* and homologs from *H*. *armigera* and *B*. *mori*. The tree was rooted by the ORco orthologs. Node support was estimated with 1000 bootstrap replicates, and bootstrap values were displayed with circles based on the scale indicated at the top left. The scale bar at the bottom indicates the branch length in proportion to amino acid substitutions per site.

To reason the putative functions of candidate chemoreceptors, phylogenetic relationships were analyzed based on alignments with homologs from other insect species. According to the neighbor-joining tree of ORs from *S. frugiperda* (this study), *H. armigera*, and *Bombyx mori*, SfruORco was clustered in the ORco branch. Notably, we identified one putative “classic lepidopteran PR” (SfruOR6) in the transcriptome of *S. frugiperda* mouthpart. The other 27 SfruORs were scattered in various “ordinary OR” branches (Figure 1).

**FIGURE 2:**
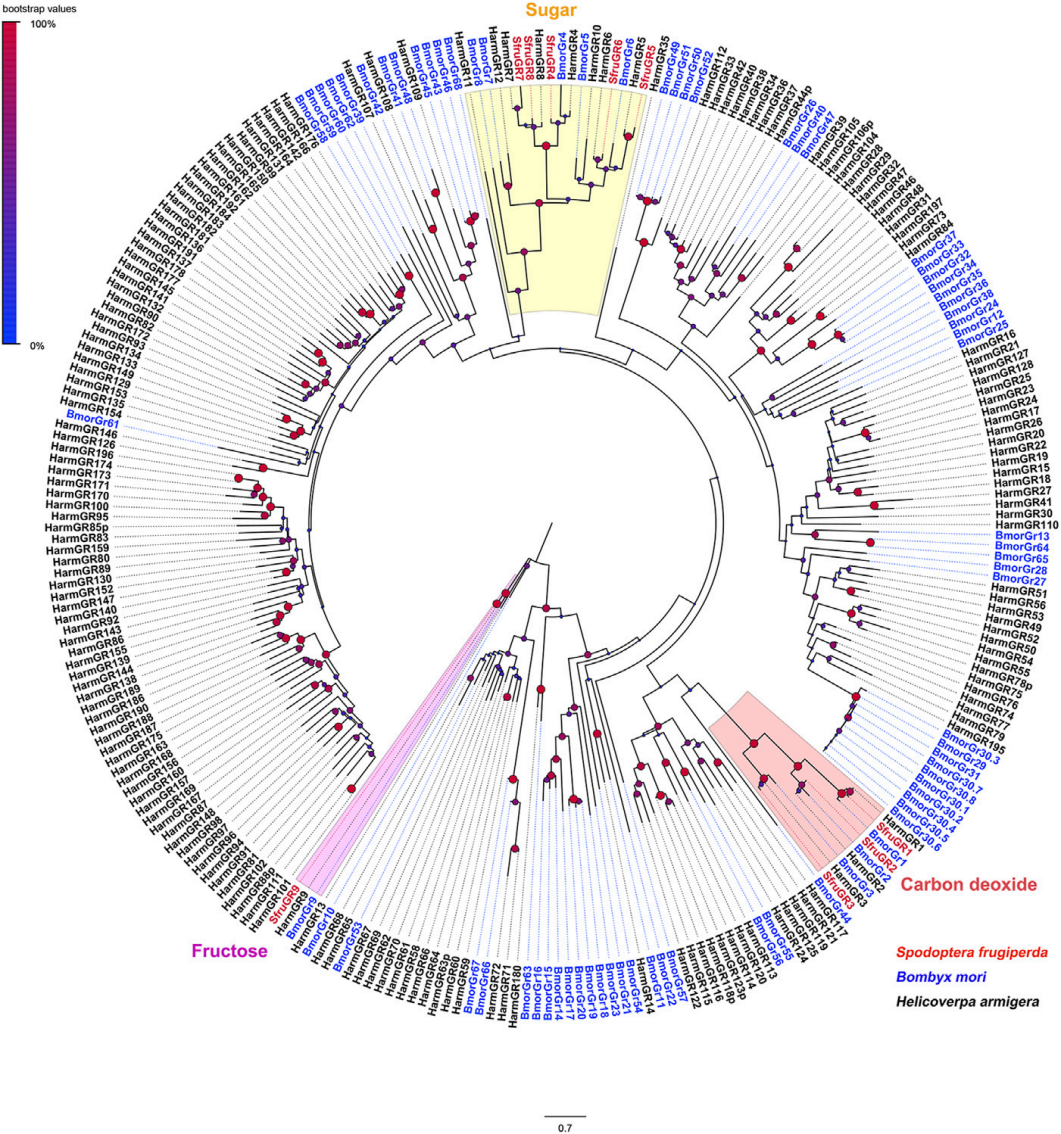
Neighbor-joining tree constructed with candidate GRs from *S. frugiperda* and homologs from *H*. *armigera* and *B*. *mori*. The tree was rooted by the GR9 orthologs. Node support was estimated with 1000 bootstrap replicates, and bootstrap values were displayed with circles based on the scale indicated at the top left. The scale bar at the bottom indicates the branch length in proportion to amino acid substitutions per site.

A phylogenetic tree built with GRs from *S. frugiperda*, *B*. *mori*, and *H*. *armigera* showed that SfruGR1/2/3 were grouped with the CO_2_ receptors BmorGR1/2/3 and HarmGR1/2/3. And SfruGR9 clustered with the fructose receptors BmorGR9 and HarmGR9. Other five SfruGRs (SfruGR4/5/6/7/8) clustered in the “sugar-taste receptors” clade (Figure 2).

**FIGURE 3:**
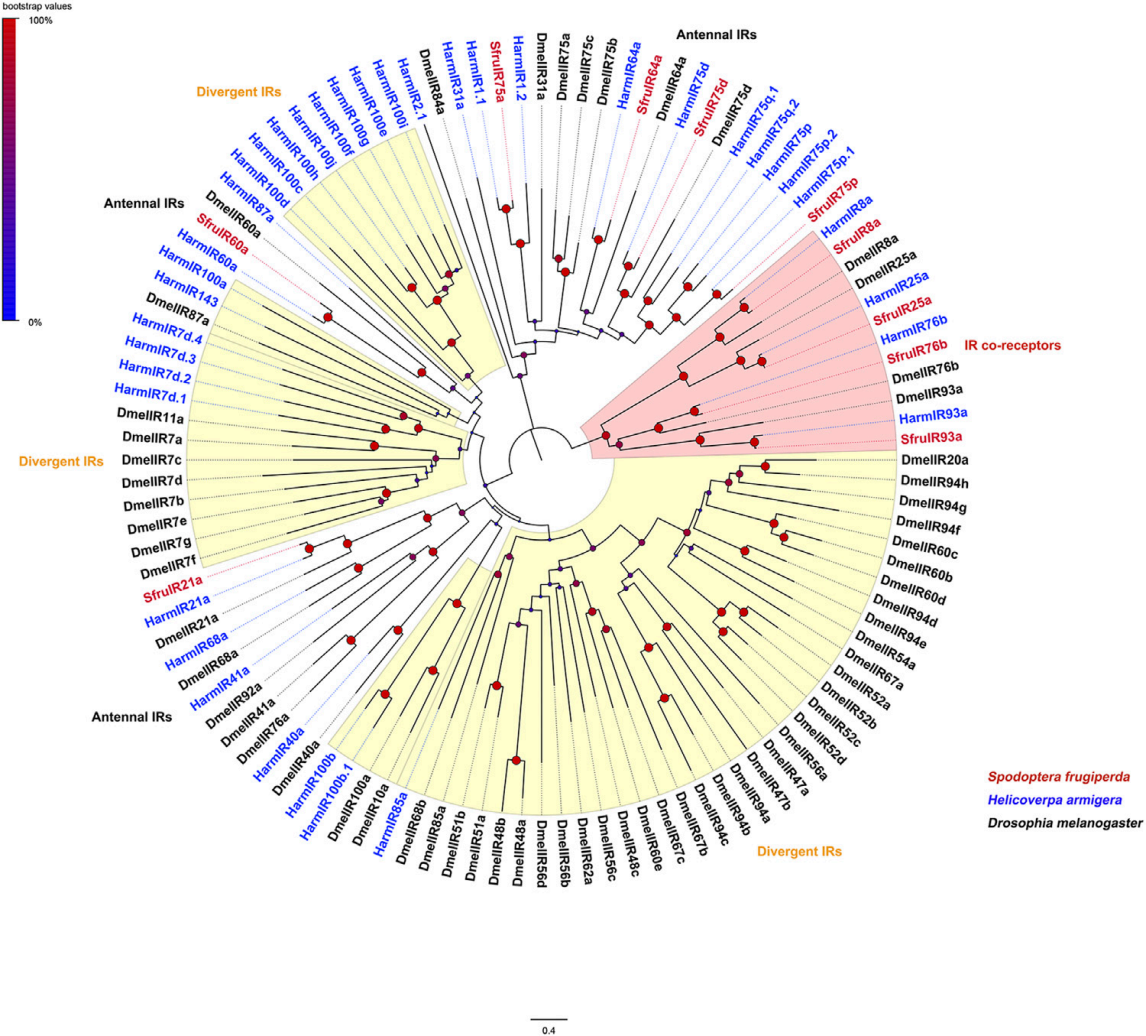
Neighbor-joining tree constructed with candidate IRs from *S. frugiperda* and homologs from *B*. *mori* and *D*. *melanogaster*. The tree was rooted by the IR-co receptor orthologs. Node support was estimated with 1000 bootstrap replicates, and bootstrap values were displayed with circles based on the scale indicated at the top left. The scale bar at the bottom indicates the branch length in proportion to amino acid substitutions per site.

Evolutionary relationships between the candidate IRs and IRs from *D*. *melanogaster* and *H*. *armigera* showed that four putative IR co-receptors (SfruIR8a/25a/76b/93a) clustered in the co-receptor lineages of IR8a, IR25a, IR76b, and IR93a, respectively. The other 6 IRs (SfruIR21a/60a/64a/75a/75d/75p) belong to the “antennal IR” clades (Figure 3).

### TPM analysis

**FIGURE 4:**
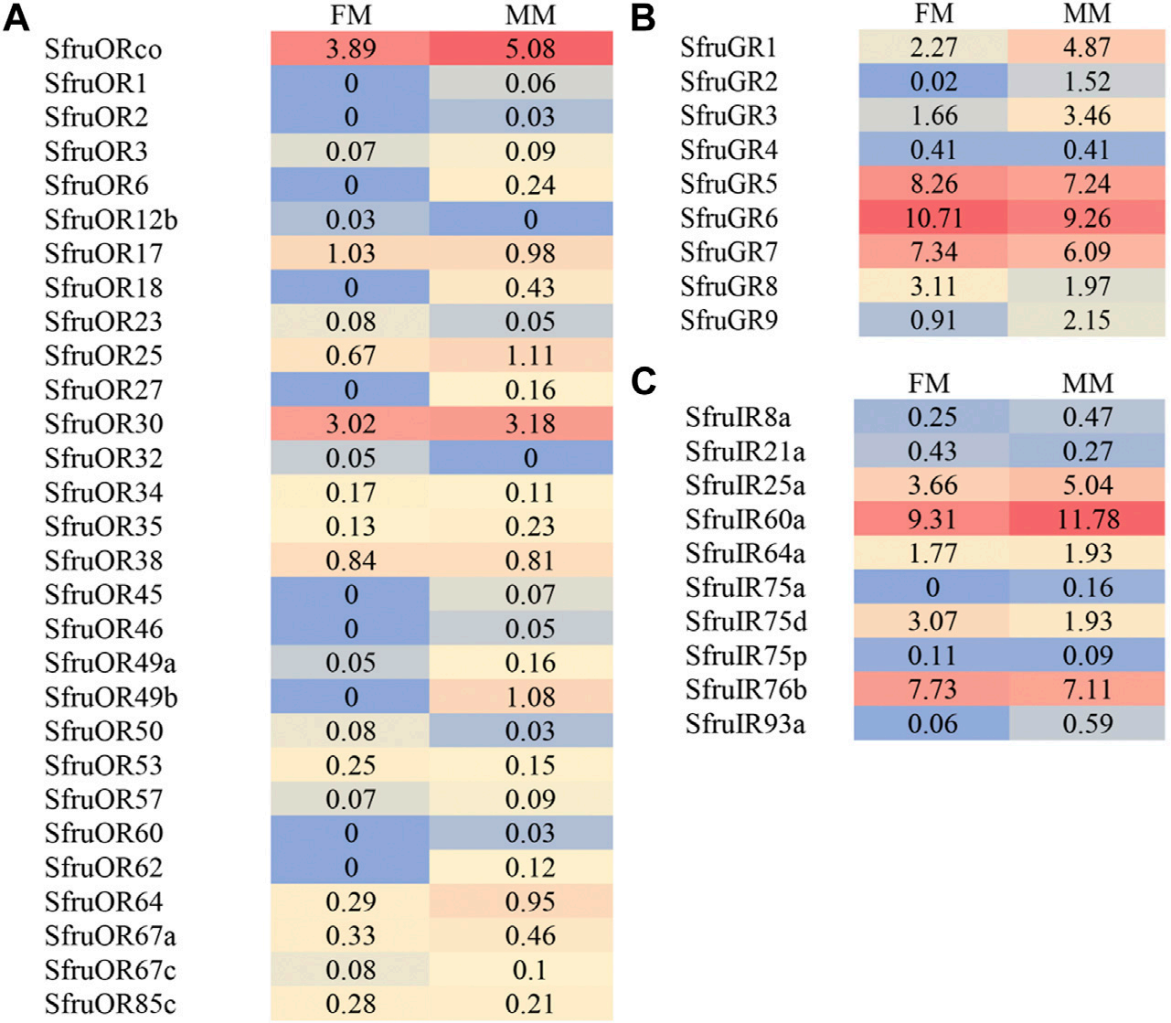
Heat-plot of TPM values for candidate chemoreceptors in female mouthpart (FM) and male mouthparts (MM). **(A)**. *SfruORs*, **(B)**. *SfruGRs*, **(C)**. *SfruGRs*. The TPM value of each OR gene is indicated in each box.

The expression abundance of candidate chemoreceptors in male and female mouthpart was then normalized across the transcriptome using the TPM method. For ORs, despite the inconsistency of expression levels between males and females, *SfruORco* was the highest transcribed gene (3.89/5.08 TPM, female/male, same below) among all the annotated *ORs*. Another candidate *OR*, *SfruOR30*, showed the second highest levels as that indicated by the TPM values (3.02/3.18). The other 27 *ORs* showed relative low expression levels in male and female mouthpart (the mean TPM value ≤ 1) (Figure 4A).

Based on the TPM values, transcript levels of *SfruGR6* in the mouthpart were the highest among all the annotated *GRs*, with its TPM values slightly higher in female mouthparts (10.71 TPM) than in the male ones (9.26 TPM). Other two sugar-taste receptors, *SfruGR5* and *SfruGR7*, were also highly expressed in the mouthpart of *S*. *frugiperda*, with their TPM values of 8.26/7.24 and 7.34/6.09, respectively. In comparison, the three putative CO_2_ receptors had modest TPM values (2.27/4.87 for GR1, 0.02/1.52 for GR2, and 1.66/3.46 for GR3) in the mouthpart of *S*. *frugiperda* (Figure 4B).

TPM analysis showed that *SfruIR60a* had higher transcript levels (9.31/11.18 TPM) in the mouthpart than the other *SfruIRs*. *SfruIR76b* was also abundantly transcribed in the mouthpart (7.73/7.11 TPM). In contrast, *SfruIR75a* and *SfruIR93a* showed low TPM values of 0/0.16 (for *SfruIR75a*) and 0.06/0.59 (for *SfruIR93a*) (Figure 4C).

### Expression patterns of candidate chemoreceptors

We performed RT-qPCR to analyze expression patterns of candidate chemoreceptors in different chemosensory tissues of adult *S*. *frugiperda*. Expression levels of the 48 chemoreceptors in female and male mouthparts were basically consistent with their TPM values in these two tissues.

**FIGURE 5:**
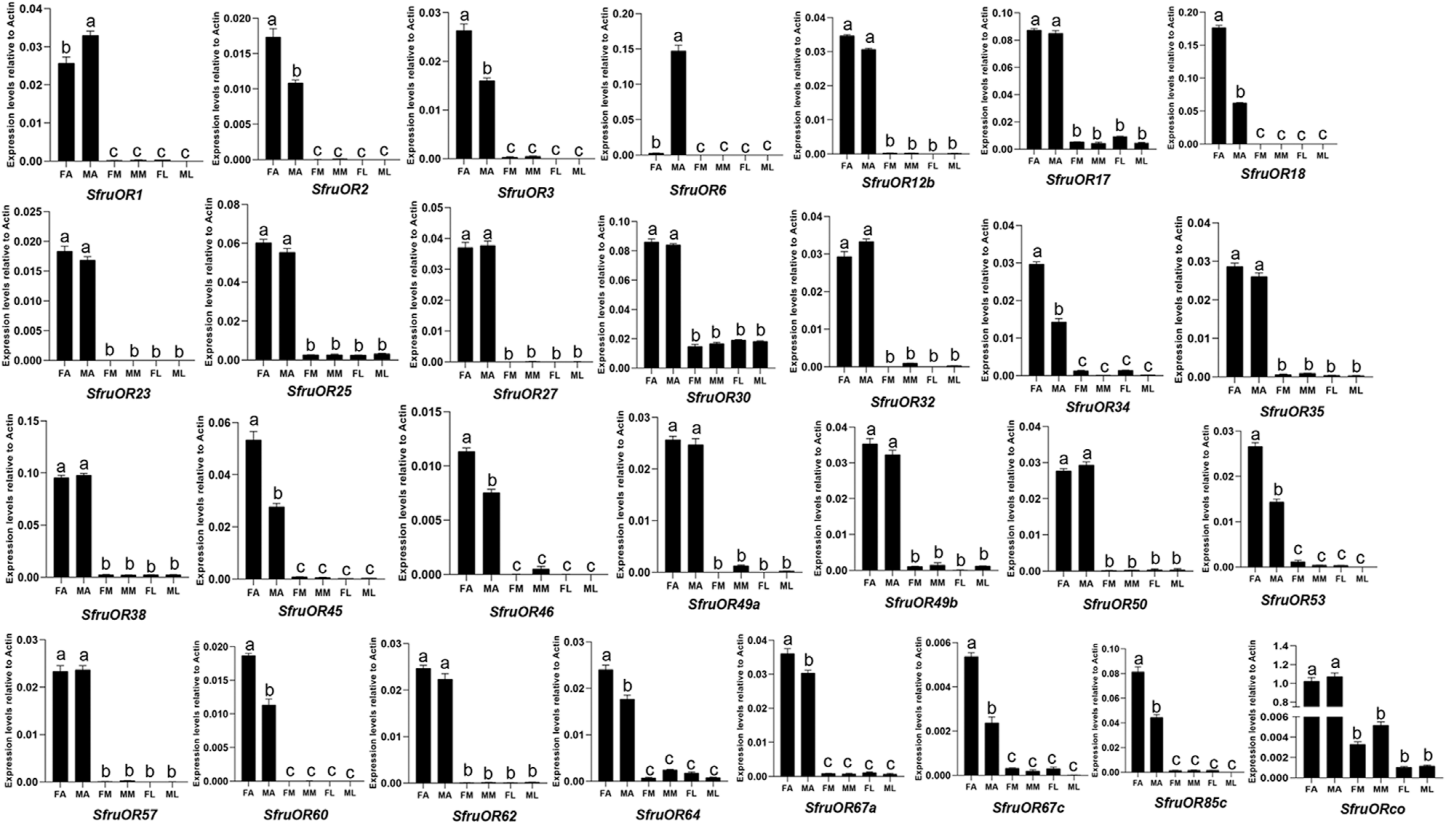
Expression patterns of candidate *ORs* in different chemosensory tissues of adult *S. frugiperda*. RT-qPCR analysis was conducted for *SfruOR* genes in female antennae (FA), male antennae (MA), female mouthparts (FM), male mouthparts (MM), female legs (FL), and male legs (ML). Different letters indicate significant difference based on a one-way ANOVA followed by Tukey’s multiple comparison test. Error bars show the standard errors of the means (+SE), *p* < 0.05, *n* = 3.

As shown in Figure 5, all of the *OR* genes were mainly expressed in antennae. Among which, 10 *ORs* (*SfruOR3*/*18*/*34*/*45*/*46*/*53*/*64*/*67a*/*67c*/*85c*) were more expressed in female antennae than in male ones, whereas 2 *ORs* (*SfruOR1*/*6*) were more expressed in male antennae than in female ones, especially the putative pheromone receptor *SfruOR6* which was predominantly detected in the male antennae of *S*. *frugiperda*.

**FIGURE 6:**
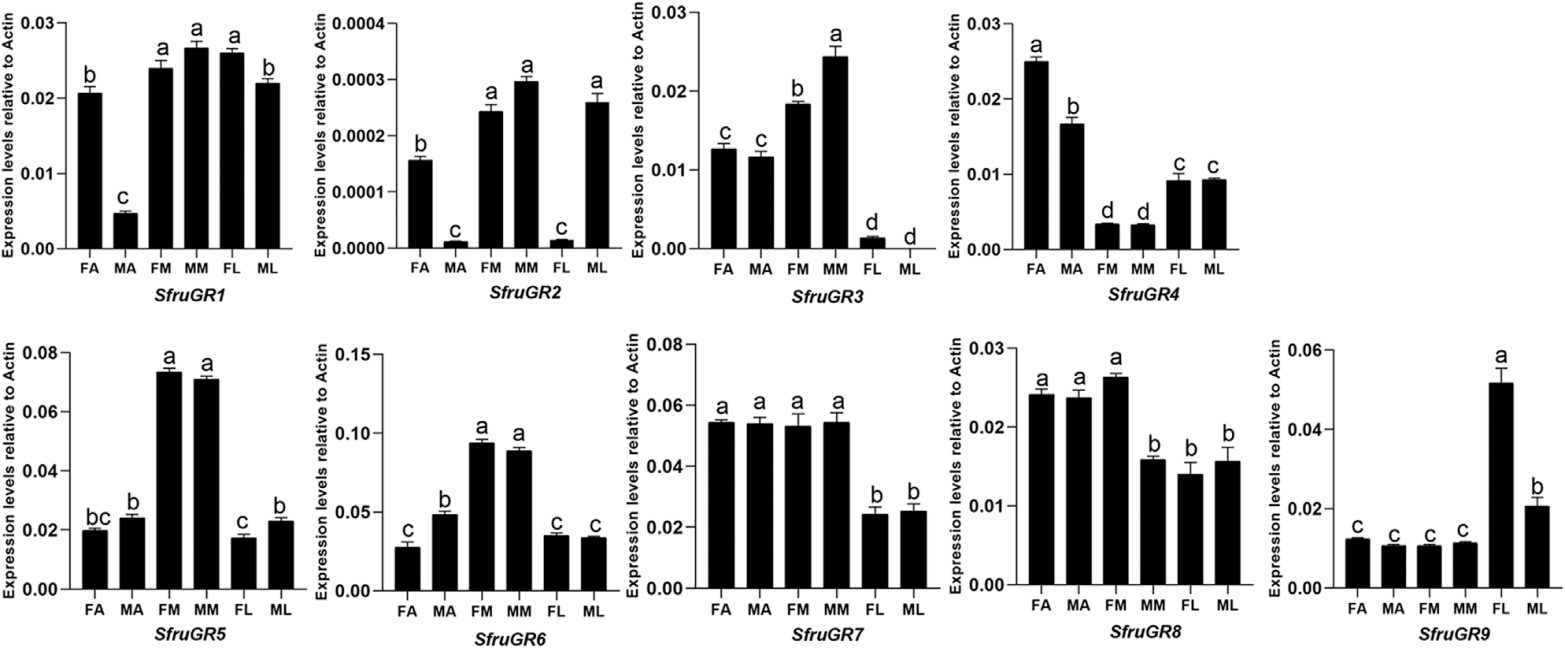
Expression patterns of candidate *GRs* in different chemosensory tissues of adult *S. frugiperda*. RT-qPCR analysis was conducted for *SfruGR* genes in female antennae (FA), male antennae (MA), female mouthparts (FM), male mouthparts (MM), female legs (FL), and male legs (ML). Different letters indicate significant difference based on a one-way ANOVA followed by Tukey’s multiple comparison test. Error bars show the standard errors of the means (+SE), *p* < 0.05, *n* = 3.

Based on RT-qPCR results, *SfruGR1* was mainly expressed in mouthparts and legs. *SfruGR7* and *SfruGR8* were mainly expressed in antennae and mouthparts. In comparison, *SfruGR4* was more expressed in antennae than in mouthparts and legs, and with its levels higher in female antennae than in male ones. Notably, although *SfruGR*9 was highly expressed in all of the tested tissues, its levels in female legs were significantly higher than that in the other tissues. The other 4 *GR*s, *SfruGR2*/*3*/*5*/*6*, were mainly expressed in the mouthparts. Among which, *SfruGR3* was more expressed in male mouthparts than in female ones (Figure 6).

**FIGURE 7:**
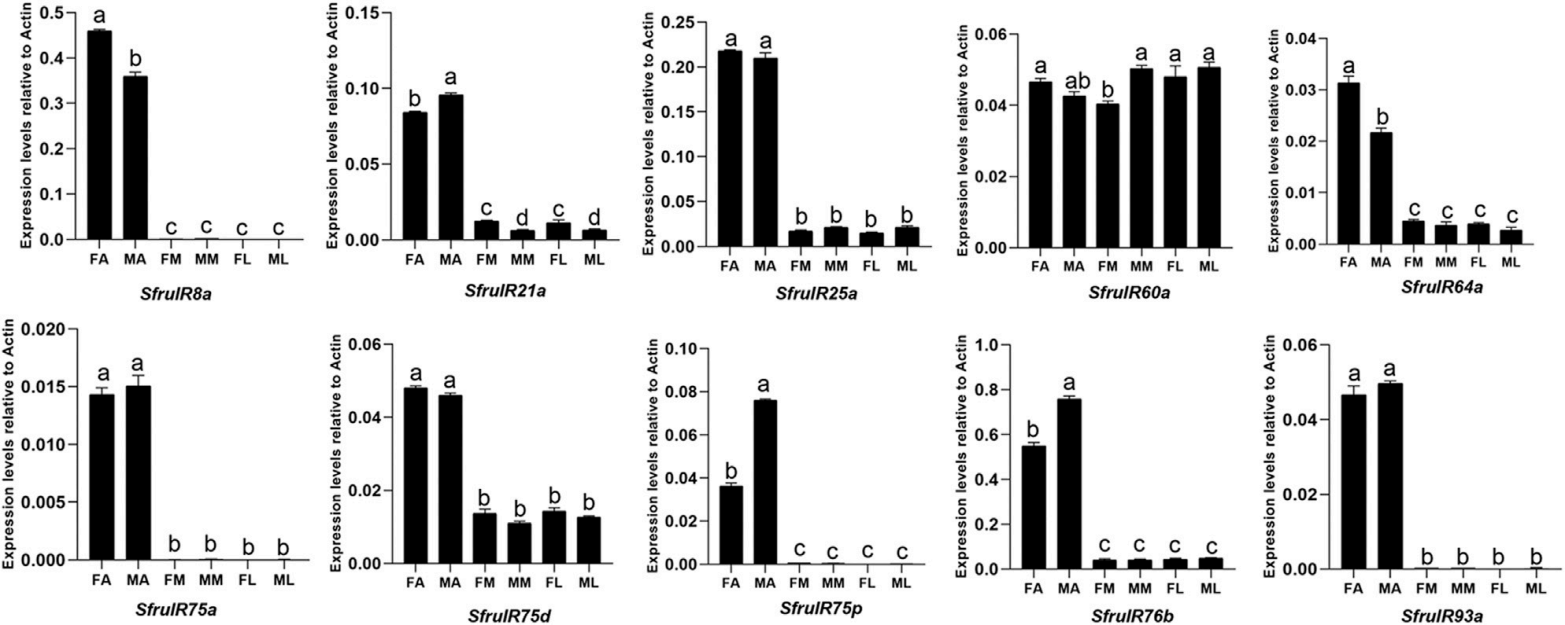
Expression patterns of candidate *IRs* in different chemosensory tissues of adult *S. frugiperda*. RT-qPCR analysis was conducted for *SfruIR* genes in female antennae (FA), male antennae (MA), female mouthparts (FM), male mouthparts (MM), female legs (FL), and male legs (ML). Different letters indicate significant difference based on a one-way ANOVA followed by Tukey’s multiple comparison test. Error bars show the standard errors of the means (+SE), *p* < 0.05, *n* = 3.

According to the expression profiling, all of the candidate *IR* genes were mainly expressed in the antennae, especially *SfruIR8a*, *SfruIR75a, SfruIR75p*, and *SfruIR93a*, which were almost exclusively expressed in the antennae. Moreover, the expression of *SfruIR60a* was also detected in the mouthparts, with comparable levels to that in the antennae and the legs (Figure 7).

### Expression comparison of specific chemoreceptors between proboscises and labial palps

**FIGURE 8:**
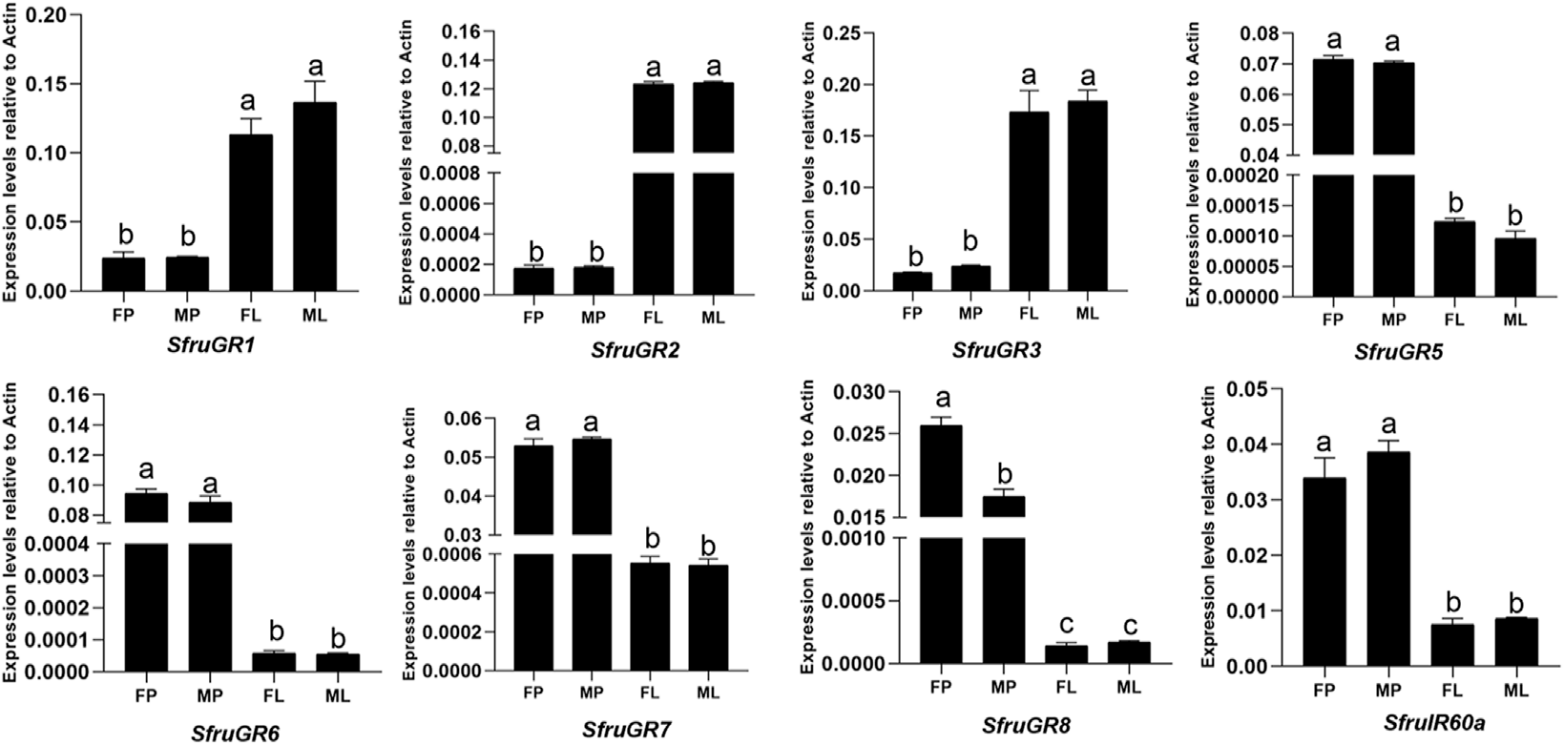
Comparison of the genes displaying high expression in the mouthpart among female proboscises (FP), male proboscises (MP), female labial palps (FL), and male labial palps (ML). Different letters indicate significant difference based on a one-way ANOVA followed by Tukey’s multiple comparison test. Error bars show the standard errors of the means (+SE), *p* < 0.05, *n* = 3.

Subsequently, we compared the expression levels of mouthpart-biased chemoreceptors between proboscises and labial palps of *S. frugiperda*. In total, 7 *GR*s (*SfruGR1*/*2*/*3*/*5*/*6*/*7*/*8*) and 1 *IR* (*SfruIR60a*) were selected and subjected to RT-qPCR. According to the results, 3 putative CO_2_ receptors (*SfruGR1*/*2*/*3*) were predominantly expressed in labial palps, and no significant differences between sexes were noted. In contrast, the 4 putative sugar-taste receptors (*SfruGR5*/*6*/*7*/*8*/*9*) and *SfruIR60a* were mainly expressed in proboscises. Among which, the expression of *SfruGR8* was significantly higher in female proboscises than in male ones (*p* < 0.05). The other measured genes exhibited similar expression levels between the proboscises of both sexes (Figure 8).

## Discussion

It has long been reported that many chemosensilla are distributed on the mouthpart of moths and that the mouthpart functions in chemosensation in moths (Kent et al., 1986; Liu et al., 2014; Hu et al., 2021). However, the identity of the chemoreceptor genes in the mouthpart and their functions in feeding are still unclear for most moth species. In this study, through analyzation of the transcriptome data, we identified 29 ORs, 9 GRs, and 10 IRs in the mouthpart of *S. frugiperda* adults. This number is bigger than the reported 4 ORs, 7 GRs, and 6 IRs in the transcriptome of the *H*. *armigera* mouthpart (Guo et al., 2018). The high quality of the transcriptome data provides us confidence for the next step researches. Moreover, we found that the number of chemoreceptors in the adult mouthparts (29 ORs, 9 GRs, and 10 IRs) is larger than that in the larval (6th instar) ones (11 ORs, 4 GRs, and 6 IRs) (Sun et al., 2022). The quantity variation of chemoreceptors may relate to their living environment. The habitats of the larvae are relatively simple and concealed. In contrast, the adults live in open environments and their habitats are relatively complicated. Correspondingly, the *S. frugiperda* adults have a larger number of chemoreceptors than that in the larvae. Though such inference needs to be validated with more instances.

Insect functional ORs are dimeric complexes of specifically tuned ORs and a highly conserved OR co-receptor, ORco (Leal, 2013). Although all of the annotated *SfruORs* are mainly expressed in the antennae, *SfruORco* also shows high expression in the mouthpart of *S*. *frugiperda*. This finding indicates olfaction roles of the *S. frugiperda* mouthpart. Furthermore, according to the RT-qPCR results, the expression of *SfruORco* is significantly higher in the antennae than in the mouthparts. Such expression profile verifies the previous inference that *ORco* is mainly expressed in the antennae of insects (Jones, et al., 2005). Another interesting finding relates to the detection of the putative pheromone receptor SfruOR6 in the *S. frugiperda* mouthpart (although the expression level is very weak). A series of functional studies in heterologous system reported the best ligand of OR6 (ortholog of SfurOR6) in *H*. *armigera* and *Helicoverpa assulta* is *Z*9-16: OH (Chang et al., 2016; Yang et al., 2017). The participation of *S. frugiperda* mouthpart (whether or not use SfruOR6 to detect sex pheromones) in sex pheromone detection remains to be determined.

The sensing of CO_2_ has been long documented in insects. The neuron cells involved in the detection of CO_2_ are located in maxillary palps (for mosquitoes) and labial palps (for Lepidoptera adults) (Kellogg, 1970; Bogner et al., 1986; Grant et al., 1995). The mechanism underlying the detection of CO_2_ stimuli at the molecular level was first unraveled in *D*. *melanogaster* (Jones et al., 2007; Kwon et al., 2007). Subsequently, the molecular mechanism concerning CO_2_ sensing was uncovered in several moth species, and 3 GR genes, GR1/GR2/GR3, are determined to be responsible for the CO_2_ sensing (Xu and Anderson, 2015; Ning et al., 2016). In this study, three putative CO_2_ receptors (SfruGR1/2/3) have been annotated in the mouthpart of *S. frugiperda*. RT-qPCR demonstrated they are predominantly expressed in the labial palps, which is in accordance with the findings reported in other moth species (Xu and Anderson, 2015; Ning et al., 2016). Further work is required to validate the function of SfruGR1/2/3 so as to elucidate the molecular mechanism of CO_2_ detection in *S*. *frugiperda*.

Sugars are vital in insect life as valuable food resources. The detection of sugars is always utilized by insects to evaluate the nutritional values of foods (Slone et al., 2007; Kent and Robertson, 2009). In the silkmoth *B*. *mori*, 5 sugar-taste receptors (BmorGR4–8) were identified (Wanner and Robertson, 2008). BmorGR8 responds to myo-inositol and epi-inositol (Zhang et al., 2011), and BmorGR9 selectively responds to *D*-fructose (Sato et al., 2011). Nine putative sugar-taste receptors (HarmGR4−12) were identified in *H*. *armigera* (Xu et al., 2017). Heterologous expression in sf9 cells combined with calcium imaging found that HarmGR9 responds to *D*-galactose, *D*-maltose, and *D*-fructose (Xu et al., 2012). While *Xenopus* oocytes expression and two-electrode voltage clamping reported that HarmGR9 responds specifically to *D*-fructose (Jiang et al., 2015). In our study, a total of 6 putative sugar-taste receptors (SfruGR4/5/6/7/8/9) were identified in the mouthpart of *S*. *frugiperda* adults. Expression analysis showed that *SfruGR5*/*6*/*7*/*8* are predominantly expressed in the proboscis of *S*. *frugiperda*, corroborating the vital roles of adult proboscises in the feeding of sweet substances. Moreover, the expression of *SfruGR8* was documented to be significantly higher in female proboscises than in male ones, indicating its roles in female-associated behaviors of *S*. *frugiperda*. According to the RT-qPCR results, *SfruGR4* and *SfruGR9* displayed the highest expression in female antennae and female legs, respectively. We speculate that these GRs are involved in the gustatory process in the antennae and legs of female *S*. *frugiperda*. Specific ligands of the six putative GRs in the detection of sugar tastants in *S*. *frugiperda* will be one focus of our research in the future. We did not identify members that belonged to the “bitter-taste receptors” in the transcriptome of *S*. *frugiperda* mouthpart. This may echo the previous inference that most bitter-taste receptors have low transcript levels in chemosensory tissues (Xu et al., 2016).

Although the function of insect IRs was initially limited to olfaction, recent findings extended their roles to the sensing of taste, humidity, temperature, and sound (Rytz et al., 2013; Ni et al., 2016; Pitts et al., 2017; Hou et al., 2022). In this study, we annotated a total of 10 IRs in the mouthpart of *S*. *frugiperda*. Phylogenetic analysis showed 6 (SfruIR21a/60a/64a/75a/75d/75p) of them were classified into the “antennal IRs”. Most of the annotated “antennal IRs” were highly or specifically expressed in the antennae of *S*. *frugiperda*, consistent with that reported in other species (Liu et al., 2018; Zhu et al., 2018), However, *SfruIR60a* was also highly expressed in proboscises and legs. Similar findings had been reported for *HarmIR60a* in *H*. *armigera* (Liu et al., 2018). Thus, we suggest that SfruIR60a may be involved in both olfaction and gustation in the mouthpart of *S*. *frugiperda*. Like insect ORs, a heteromeric complex is needed for functional IRs, with at least one specific IR and an IR co-receptor within a single CSN (Abuin et al., 2011; Silbering et al., 2011). In our study, 4 putative IR co-receptors (*SfruIR8a*/*25a*/*76b*/*IR93a*) were identified in the mouthpart of *S*. *frugiperda*. According to the TPM values, *SfruIR76b* is the highest expressed co-receptor among the four co-receptors in the mouthpart. In *D*. *melanogaster*, DmelIR76b had been demonstrated to function as the co-receptor of specific IRs that tune to amino acids (Ganguly et al., 2017). The role of SfruIR76b (whether or not sense amino acids) in *S. frugiperda* mouthpart remains to be elucidated. Although “divergent IRs” were reported to be the largest group in *D*. *melanogaster* (Croset et al., 2010), we did not identify putative “divergent IRs” in the current study. This may be due to the relative small number or/and low expression levels of “divergent IRs” in the mouthpart of *S. frugiperda* which need to be experimentally investigated in the future.

## Conclusion

By analyzing the mouthpart transcriptome of adult *S. frugiperda*, we annotated 48 chemoreceptors. Expression pattern investigation revealed several chemoreceptors being highly expressed in the mouthpart (labial palps or proboscises). We suggest that these genes could be important in the chemosensation in *S. frugiperda* mouthpart. These findings give us useful information for further investigation of chemosensory mechanism in the mouthpart of *S. frugiperda* as well as of other moth species.

## Data Availability

The datasets presented in this study can be found in online repositories. The names of the repository/repositories and accession number(s) can be found in the article/Supplementary Material.
