# Supplementary material for: A mouthpart transcriptome for Spodoptera frugiperda adults: identification of candidate chemoreceptors and investigation of expression patterns
Source: Front Physiol. 2023 Apr 25;14:1193085. doi: 10.3389/fphys.2023.1193085 (PMC10166800; doi:10.3389/fphys.2023.1193085)
Supplement: Supplementary file 1 [file DataSheet1.ZIP › Supplementary data/Table S1 (tree).docx]

Table S1. Amino acid sequences of ORs, GRs, and IRs used in phylogenetic analyses.

**ORs:**

>BmorORco

MMTKVKTQGLVTDLMPCIRLLQAAGHFLFNYHADTSGMNMLLRKIYSSAHAVLIVVHYICMGINMAQYKDEVNELTANTITVLFFAHSIIKLAFFAFNSKSFYRTLAVWNQSNSHPLFTESDARYHQISLSKMRRLLYFICGMTVFSVISWVTLTFFGESVRMIASKETNETLTEPAPRLPLKAWYPFKTMSGGGYVFAFIYQIYFLLFSMALANLLDVIFCSWLIFACEQLQHLKAIMKPLMELSAALDTYRPNTAELFRVSSTDKTEKVPDAVDMDIRGIYSTQQDFGMTLRGAGGKLQNFNAENNPNGLTAKQEMLARSAIKYWVERHKHVVRLVASIGDTYGTALLFHMLVSTITLTLLAYQATKINGINVYAFSTIGYLVYTLGQVFHFCIFGNRLIEESSSVMEAAYSCQWYDGSEEAKTFVQIVCQQCQKAMTISGAKFFNVSLDLFASVLGAVVTYFMVLIQLK

>BmorOR1

MLLSFKDDSRSPDIQKPQNFQYMKILRFNLKIICAWPEKQLNEIRSLGHSIHRVILPIQSVVCLACGILYIHFHFNEIPFFILASTFITVMMNLATCSRTALVMLFERYLVLTGRFITVMHLFNFQKNSDYAYKLCTFVNRMSHFYTLYVLFSMFMGLGLFNLLPLYNNYVSGAFSDPYGPNVTFFHSVYFAFPFDYSHNFRGYIIMALFNSYVSVTCSIGLVMFDLLMCLMVMHVWGHLKILSHNLINFPRPKASHVITTPNGPTNVETYTEEESKEVFARLRECIKHYGTVDDFANDMSETFGVILLVYYGFHQVSLCMLLLECSDLSTKAMLRYGPLTLIMIQQLIQISIIFELLGSVADRIPDAVYQLPWECMDVKNRRVVYGFLRRTQNPVRFKAMGMLDVGVQTMASILKTSIS

YFVMLRTVAT

>BmorOR3

MIFVDDAVIGIKDPREYRHLRVLRTSLRLLGAWPGHYLGEETGSKYECAPMFLLMFIKIACLYLTIVYLRNNADVLGFFELGHVYLTIFMTFVTLSRGFSLTWNPNYHKVVKKFITEMHLLYFKDNSEYAMKTHRRVHKISHFYTVFLKVQMIAGLTLFNVIPMYNNYRQGNYASDRPANITYDLSIYYETFDILNTPNGYIFICVFNWFASYICCSFFCSFDLILSLMISTVSGHFRILIHNLLTFPLPEAITASKKFVDKHRCNGNRSEFVLEEAKLYSPAEMWQVTDRLRQCIDYHRKLVEFTGDISEAFGPMLFVYYLFHQVSGCLLLLECSQLNTAALVRYGVLTVVLYQQLIQLSVIVESVGTVTGRLKDAVYEVPWEYMDTSNRKTVAIFLMNVQEPLHVNALGLAKVGVQSMAAILKTSFSYFTFLRTVSE

>BmorOR4

MFKIIKNIIVENDALKQVEKPQEFQYMKWVQYHLKYIDGWPNMDMNKKNVSKIRFHKRHLLVVEQTITFLSQMFYIVKNYGKLSFFEIGHSYITALMTIVIFSRSVVTALGRYRKIARYFVSSLHLYHYKDISEYALQTHLLVHRLSHYYTVYLISLVVTGMLLFNITPLYNNISSGVFNSPRPENMTFQHAVYLGLPFDYTTDIKGYFVVFILNWHLSHIAASYFCTFDLFLSLLILHLWGHLRIILNNLKTFPKPYTNNSMYTEEENQVVLLKLQECIRYHNFIISFTVMMSNVYDVVIIVYYLFHQVTGCLLLLQCSTLDWESLSRYGPLTLIIFQQLIQVSMIFEILGFLSDKLPNAVYSIPWEAMNVTNRKLVQVLLQKSQKPIQFKAMNMMSVGVQTMASIIKTSISYFIMLRTIARD

>BmorOR5

MLLYYPNTQVKEKVNNVEEFTYIKFLKSFCKIMDFWPEREEKNSKTRIFRLRYILVLQFCFTLVAGVLYLTNSVGKQTFYDLGHTIITVLMNVVSLSRLILRCFKKYDVVGQQFINKIHLYHYRNDSEYAMKIHTVVHKISHNMTYIFSFCIIFGTVTFNLTPIFNNIGSDAYKNPRPDNVTLQQCVYYALPFDYTGNFKWYLLVAIFNVQKTFFCTSLFILFELSLSLMIICLWGHLRIFIHNLNHIPAPRNSFEYTKEERQEVDDTLKKCIQHHTLIIGFVRIMSETYGLAVLIYYAFQQVVGCLLLLQCSQMELKTVTRFGFLTLVLNQQLIQISVIFELLGYMSDKLQDAVYCVPWEYMDTSHRKMVYMMFRQSQIPLQLKAMNMLSIGVKTMVSILKTSVTYYLILKTVTTD

>BmorOR6

MKEEYYLQHPRTQLFYKVLAHVSTIESTIDLTWWGYTFPKYVGWFYHLQCNVVRLFGKCVVVSQILFIILNYQTIDKSVFIIAITITPLGALVGIKAESAKAECYVNLMKNFMDKVHIHSIYRKNENNEFVKKKVIQIERVSRFTAYFLVILIAINCLSWMLKPTLHNIKHFEEIMNKSMEFQYYIYFWTPLDYKYNLRDYIIIHTLCIYLGATAVTVIVTFDIFNFIAVFHVVAHIQILKNNVKSNWSDDFNESEKKGYLVSILEYHAYIIRIFGEVQSAFGLNVASNYLQNLIEDGLFLYQIMNGEKENVLMYGLMIILYLGGLIFLSIVLEEIRRQNYDLCEYVYALPWEGMSLENQKIFVVFLQRTQPDLEFETVCGMKAGVKPAFSIVKSMFSYYVMINSRF

>BmorOR7

MLLYHPNTQVEEKVNNVEEFTYMKFLKSFCKIMDFWPEREEKNSKTRIFRLRYILVLQFCFTLVAGVLYLKNNFGKKTFYDLGHTIITVVMNVVSVSRLILRCFKKYDVVGQQFINKIHLYHFRNDSEYSMKTYKAVHKISNNMTYIFSFSIFVCVVTFNLNPVFNNIGSGAYKNPRPDNVTLQQCVYYALPFDYTGDFKWYMLVAIFNVQKTFFCTSLFILFDLLLSMMIIHLWGHIRIFIHNLNHIPAPRNSLEYTREERQEVDNTLKKCIQHHTLIIGFVRIMSETYGLAVLIYYAFQQVVGCLLLLQCSRLDLKTITRFGFLTTMVNQQLIQISVIFELLGYMNDKLQEAVYCVPWEYMDTSHRKMVYMMFRQSQIPLQLKAMNMLSIGVKTMASILKTSVTYYLMLKTITANEA

>BmorOROR8

MSLSTRCLLKDFCKYVYYAGAGNFWYEDIYKETVPYKMYVVISFFTYTVMIFLENLAALFGKLPEVEKNSAVMFAAIHNIVLTKMFLLLYHKRSISKLNCEMAAVGENLEEASIMRRQFRKMRLGTALYFISVYLSLVAYGVESARRTIVEGAPFYTVVTYLPDYDNTTVLASFLRIFFYITWLYMMLPMMSADCMPIAHLITMTYKFVTLCRHFDQIREKFQINVKIMAKTEATEILKLGFIEGIKMHQKLMYLADEIHRVFGIIMALQVCESSAVAVLLLLRLALSPHLDLTNAFMTYTFVCSLFLLLALNLWNAGELTYQASLLSNAMFYSGWYFCDFEKDWCRDIRRLVLIGCAQAQKPLILKAFGVLDLSYETFVSVARMTYSVFAVFYKRGD

>BmorOR9

NVDNVEDFKYVKWLRNHLKTVDAWPVHSKSKRKIQKRYVLPIFSAACFISQTVYLKNGIGTLSFVVLVHSYICFLINGSCLCRGILIATERYKRLATCYLKTVHLFHHKNRSEHAMKIHVIVHRLSHYYTIYLISLVFVGMVLFNFMPIYNNINSGAFKSPRPENVTFQHAMYLALPFDYTTNIKGYFVVFILNWYISLVTTSHFCTFDLFISLMIIHLWGHIKILMCSLEDIEGFVPGSSFKFTIEQNRKIYLILQECIRHHQFTIDFTNEMSSTFGLVILFYYFFYQVSGCLLFACMLTNESLSRFGPMTFILFQQLIQLSIVFELISSLSENLPNAVYNVPWEFMDKNNRKMIQVLLLQSQKLIQFKATSMMNVGVQAMATILKTSVSYFIMLRTMYQEH

>BmorOR10

MRTNAKSFLFVPSKVLTLCGVWPVEKTSIFSLIYRSIMLSSQFCFLVFNGIYIGLMWGDLKAVSDALYMFFTQTTCCSKAIGFYFNFMKIKRIVASMDDVLFTAMSIEDQATIFSHSRTVNKLYKGVLGFTGFTLVQWTVLSLIGSGRTLPFNEMWVPTDISKSPNYEITFVVELWMMVISAALFMSVDTITVATMMFSCAQLDIIMKKTQQIQEIPLSPDLSSRNRSELHEKNNGILIDCIKQHQAIVRFSELCEGTFQVHSFFHLGGIVFMICVIGFRMAGESPVSAQFWAALSYLVIILGQLYLYCWCANELTTKSEQLRDKLYLTPWYDQDVKFKRNLCIAMECMAKALTFRAGSYIPLSRAMFVSILRSSYSYFAFLNQANEQ

>BmorOR11

MDEHSHFETSLNKIKVLFKYSGMNLENTVTNTYEFLNHRWVYILNHAWTLAAVTFICIGISNGQNFIEMTCIAPCVAMTVLAVSKSFFHYINENAVKSLLENLIELERTDFERTKSVQRTEIVATEKQLLNMVINVLYVLNCSMILVFDMTPLIIIAIKYWTTNKFVRLLPYLDIFVFVPYKFEYWVMAYILQIWAECIVLLFIGAADCLFFTCCTYIRIHFRLLQYDFERLTSSRRESDGLRDDEDFRETYTNLVKRHQGLIESSSILEMIYSKSTLSNFVLSSLVICLSAFNVTVIVVNDVTIVMTYLIFLAMSLMQVYFLCFFDMLMSASEEVGNAVYNCSWYTEKASTGKDLLFTITRS

>BmorOR12

MTRITDVFSLNFIFWKFLGLWGKSAPSKYNMAYTVFYLFASLFVYDIFLTLNLIHTPRKLETLVRETMFYFNHLVAVTKILMMFIMRKKILVIFDLLDCEEFKPNDENSQEIMKRKTDFYYIYWRIVAVTSNLSCFMLVIGPLIKMLIWKIELGLPVCKFYFMSDELRNKYFVIWYIYQSFGIYNQMVNNLNLDTFNCGMLWMAVGQLQILKTKFVNLKLNDFENGLDLKSRDDMQIERLRKYLTHYEIILKYCATVQDILNITIFVQLGMSSIVICVGLCGFVAMPSNTETAIFMSSYLITMTMQIFVPSWMGTQISFECGELMSAAYCCEWIPRSKLFKRSLILFVERAKTPVRITGLKIFTLSLDTFTSIMKTTYSFFTLIRQLQVDEVN

>BmorOR13

TSRPYKFFNILRLCTMAPKQIDCFEINWKFWKFLGIWSENKPHRYYKYYSKIFITFFVILYDVLYTINFYFVPRQLDLIIGEMLFYLTELSVLSKVFTFIIMRHKLKIIFEILESDAFQTDTEEELKILHRAKVFIKRYWKIVALVSITANLTHISSPLLKNLIFKVELVLPVCSYSFLSESFLKTFEYPLYFYQIVGIHFHMLYNLNIDTYFLGLMILIIAQLDILNVKFRNLKSGKDHTQLNESIMGLNKNLDHYNEIERCVLKHDYYYNPIPFYFNKQQTSRICLICILQSVPVEYYIFLATYMFIMIIQIMVPCCFGSRIMDKSILLSSAIYNCDWTSNSKDFKINMRLFVERANKPLSITGGKMFSLSLATFTS

>BmorOR14

MSNYIFKPFHETYRIITFTMIAAMIYPNPATEKRRLIYIGLMLLSVIPLAFMIVTEMYEFFMASDLNNTIRHSTVIGPFIGGFVKSPFFEIMFVYTFFSSFIYIINYVGYDGFFGLCINHACLKMKLYCRALEDAMRSDSRRHEKIVAVIEEQRRTYEYIALIQDTFNIWLGLIYVATMIQMCTCMYHIVQSFNIDVRYIIFVISIIHIYLPCRYAANLKCMAAETPTLIYCCGWESVSDLRIKRMMPFMVARSQVIVEITAFNMFAFDMELFVWIMKTSYSMFTLMRS

>BmorOR15

MMTLVYQTDIFKPNVFFWKMFGIWADRKSSKTYKYYSFVFLFITLIMYNSLLAINLLYTPLKIELLIREVIFCFTEITVTTKVLMILFKRNKILDAFDLLNKNEFRGNSEESSAIIQKNNSAYKTYWKLYAILSNFAYSSQVLGPLIVKLIWKTKLELPICNYYFLNEELRHDFFSGWYIYQSFGMYGHMMYNVNIDTFISGLLMMAVTQLKIIQTKLLSLKLNPRERKMDRGLMNITEVLKLNEILKHYELVLKYCSTVQSILDVAMFVQFGVASAIICVAMCGLIMVRSSTETLLFMVTYLFAMTLQIFVPAWMGTQLHFQSQELVFAAYNSEWIPRCQSFKRSIIIFVERAKIPITITGLKMFPLSLATFTSIMKTAYSFFTLIRNMQTLQEE

>BmorOR16

MPVSPERSPHYHLGYSFQLVTICMSAYMYFGVDSVAFSSVIFGCAQIGVIKDKIMSIKPLGIYRNHKTYTNISRYNRKTLIECVKHHQAVISFTELVEDTYNSYLLFQLVGSVGIICGLAQCPITIPIAILCYLSVMISQLFVCCWCGHELSATSEELHTILYNCAWYDQDVKFKRDLNFMMARARRPILLRAGYYISLSRQSFVSILRMSYSYFAVLNQTNK

>BmorOROR17

MREDKMEINNSQKFYTKMIFRYLYSVGLGDWWYQHEDRSDSHRKLYCLWAVISNAYIFLNICNELLANFRKDLTDVEKNDAIQFSFAHPLIFAKIASFFFNRKKIREVFGRLLEENRSVYSCGELEKESMKQIKRYSLAFIGVSYMTLVMSTIDGLRAHFKEGIPIRTEVTYYPSPSNSGVIVNILRFLVEFHWWYIVSVMVAIDSLAVASFVFVTFKFKLLQRYFKDMGLTVRRDQSNMTDEALADKFRRDFIVGVKLHENALWCAENVQKAFGWVYSVQVFETVALLVMCLVKLVTTNHNMIFLLANFAFMLCVIILNGSYMMPAGDVTYEASEVPTSIFLCGWELVRQTDLRFLVVVAIQRSQVPVIMKAFGIMTLSYSNFIAVSLFKFYVQFQINLF

>BmorOR18

HFFDFNIKYLFYVGLWPSNEAKRIEKIAYKIYEYQLHVLSLIFLVTTGIGTYKNHKDIIALLTNLDKTLVAYNFVFKVIVFVYKREELRKLIEQIVQSGDQITEDRKALMAKLVIVLTGISTVIITAFSCLALFEGEMTIDAWMPFDPMKSKMNLFAASQILAATFVVPCGYRAFAMLGIVCSLILYLRDQLVDLQNKIRDLRFATGNVEKLRDDFKLIVKKHVRL

>BmorOR19

MHEFVINVQNETTKLYDQLNIILYILGLQGIWVDEIKLSRRFHVFFKVVTFILHIMCGMFAGLQFFAIFTQNSLNSQQKSDVIVIGISNPMAYIFCINFIRNRNEIKDLFYHLAVVLKIYYNDVEIEKSMVNKIKSYLSTYVFASITILVSNGIIAFFQTINSDEPFLGIITAWPDKTDTSKTASYARIGFYLFWCIHFFRISTVFAVIVCILISIKYQYKFLCSYFESLNKIFDDETSSHEVKEAEFENAFCNGIKIHTQIIWCVRRCQIMCRTVFSANIMLDTFVLVILMLAMVNSENDFYGLCSQMSSVLVTVVLMAFFMWTAGDINVQASQLPDAIYGSGWYNCRGKSSARIRSLVTISMNKAQQPILMWALGFVELSHKNFVAIIKSAYSVFSVFY

>BmorOR20

MIQASKYPNSKTKELFRKIAHIAYICGLPNFWIEELNLPKSFIRVYDKIVRIFNVATYFFLGIEIAAHFTQHHLTNKQKFDLLLYSISHPILNGYGVIVSRQVGNVKKVLLDLIVNLKVKYNDPVIEEAMIKISMTYSVSFITNCVLSMLTYTFDALLMVYKKGVTFNVIITAWPDVEDTTTEASIGRIGFHIFWWLFVTRPFAVYVLVINLTTCLSHQYMNLQSYFFHLEDIFKENLSQNEKEAKYEAEYKIGVMLHANTLRCTRRCHMVWNGVMSGQIIFNISLIVIIMAQMMPFLSAPVSSCGMLVTSQFRSVHRFYASRLATAMYCSGWQNCRGKSSVSIRNMVMNTIAVAQ

>BmorOR21

MNKNMNKNHYILKTYCDKIFLVGSGNFWYQKTESRNDKTLLYKIYSCVLFFTYGFMTVLEIMAAMMGDFPEDEKRDSVTFATSHTVVMIKFISIIKNKELLKTLNRKMMMICEAHEEQTLMDEMYRTVKINVVAYCVAVYGSATFYVFEGLRKFYNGSHFVTIVTYYPSNDDDTLAATIVRIATTLVLLMMLLTMIISVDTYTMAYLIMYKYKFITLRHYFKRLRENVDELVAAGKARLAAEKLAQGLVEGIKMHNELLSLSKDIDKAFGTVMALQLCQSSGSAVSLLLQIAVTMYLLLALFLCNAGEITYQASLLSDEIFYCGWHKCNSPVLSTQRNIRDIVLIAILRAQSPLVMKAFKMVVRSTYSVFALFYAQNK

>BmorOR22

MNKNMNKNHYILKTYCDKIFLVGSGNFWYQKTESRNDKTLLYKIYSCVLFFTYGFMTVLEIMAATMGDFPDDEKRDSVTFASSHTLIMIKFISIIKNKELLKTLNRKMMMICEAHEEQTLMDEMYRIVKINVVAYCVAVYGSVTFFVFEGLRKFYDGSHFVTIVTYYPSKDDDTMLASIFRIATTLVLLVMMLSMIISVDTYTMAYLIMYKYKFITLRHYFKRLRENVDELVAAGKARLAAEKLAQGLVEGIKMHNELLSLSKDIHKAFGTVMALQLCQSSGSAVSLLLQIAVTMYLLLALFLCNAGEITYQ

>BmorOR24

MPEELFLDRSIKKIESYFRWMGINIRSGDNNNKKDVFKIRCIYFINFVLLNTDVLGAIFWFRSGLEQGKTFTEVTYNAPCLTFSFLANFKMLSLIFYEKTVHELIAALQKLEIKHFLRQNCAEELKMLKDEKNFLHAVFKGSKIVNYASILTFGCSPLVLIASNYYKTGRMDYLLPLIVLYPFDVDNITVWPIIYVRQIWSVITAVIGVCATDYLFYTFCVYISTQFRLLGHSIERVVPNNGLSVRTRLNGNLRMKFVENLKWHQELIRAASLLEQIYTKSTLYNFVTSSVIICLTGFNVAVVEDFAVILSFLFFLFMSLLQIILLCFFGDKLMKSSTNISDAVYNSKWYLTEKNVGKVLLMVQIRSQRACRLTAYGFAEVNLRAFMKILSTAWSYFALLQSLYSSHE

>BmorOR25

MFEKALRSANFYMRVIGIPTDIRDGNRTLMERLRNRWFYCINFLWLNTDVAGEITWFVKGLLNGSSTLIENTYLIPCLTLCILGNVKTFFTIKYANHIIDLVAILKDLEIKNNAARKNETEIVKERLKFLTTSNKFLLFVIGTGIIAFGIGPLMLTASIYFSSGDMKLKLPFLIWYPFDSSDIRYWPFVYVHQVWSACIACCAVYGPDCFYFTSCTFIHIHFIHLQNDITNVIVESSRARKNGLYRGCHQAFLELTNRHKDLIRCVNLLEIIYSKSTLVNVVSSSLLICVTGFNVMVTFCWFAAPFASFLALGLVQTYLLCYYGDTIMCSSTEVSDAVYNSTWYGTNISQMRDYLFVMKRAQKPCKLTAYGFSDVNLRTFSRILSTAWSYFALLITIYRGNGQQ

>BmorOR26

MSTGSAAGDSVAPHLRRLRQVGFCQLDPTSQSRRPILALMHRVYHRLVLAATVLYIFEQLTYAYQARNDMERLSRVLFLMLCHLTCIAKQFVFHSDADKINQLVVGLDDALCNQPVETHRLLLLETSRRAARLLMLYSGCAVSTCILWAVFPLLDQLRGRTVEFAFWIPIDYRHNAFQFAVVLAYAFYSTSLVAVANTTMDAFIATVLYQCTTQLRILRMNFESLPERAYALSRKTRQDYHTVTHELLVDCLLHYKKITETCNLLEQIFGKAILVQFGVGGWILCMAAYQIVDLSVLSIEFASMILFISCILTELFLYCYYGNEVSTESERLVTSIYSMEWVGARLGFQRGLLVLLERARRPVRPAAGLVIPLSLQTFLKIIKSSYTFYAVLRQTK

>BmorOR27

MPSSFFLPNLENPDYPSLGPTLKGLKYWGMWQSGGIKRILYNSIHAFATFFVITQYVELWIIRNNVELALRNLSVTMLSTVCVVKAGTFVCWQKYWSGIIGFVSNLEKEQLSKNDAATQAAIVKYIKYSRRVTYFYWSLVTATVFTVILAPLVGFLSSPERELIANGTLPYPEIMSSWVPFDRSRGFGYWVTALVHTLICFYGGGVVANYDSNAVVLMSFFAGQMKLLSINCSRLFDDGNEVISNNEAMKRIKECHYHHVYLVRL

>BmorOR28

MHTLALVFALLYPSNCNIIKRAIGITLIIALSGGQLFWCMTYTFKCVYELSIFNFARNNSSNNLFFFCSSVIFKTMKYCVILVKISRHFLKGNNDLGEDYKIVYKEYNKTHDFKRLMVHEMKLKYFEDIQSVGPPFHCMFAYNFLQVCVLILNYSGFDGSFCIASIRLCMKLKLVVYKVQKAFAESKSVSELKHQLNDAIKDNLDALKFHEQIQNVFFIALSFDVKFSSLCVFGTLLVFLPCHYASNLTK

>BmorOR29

MFDFLQNLEDSERPLLGPNFWLINKTGLLLPKTNFGKLAYILVHEIVTFFVVTQYVELYVIRSDLDLVLTNLKISMLSIVCIVKVNTFVFWQTSWREVLEYVNEADKFERNQTDETRGKIIETYTKYCRRLTYFYWSLVFTTFLTTTNTPLMRYWSSPIFREHLRNGTEDFPHIFSSWMPFDKNHSPGSYCTIVWHVLLCAYGAAIMAAYDTCIVVIMVFFGEKLNLLRERCKKMLANDLYNHAFVIGQLHDIHVQLI

>BmorOR30

MSVSNLKFEVLFKPTTMSLHMNRSHPSIKRNKIWLLQFISLMTLTVFCATGLITSLLFHDLKFGKYMEASKNGTIAMLSFTTTFKYSLLLYLQKSLNRLIAKIDMDYEIAKGLTPQEKAIVLNYAKKGVIVSKFWLFTAFAITFCFPLKAFIIMGYRFFIKNEFRLEPMFDMTYPEPIESYKTSFPVYFILFVVFFLFGCYASSLFVAFDPLVPIFVLHACGQLDLLSLRITKLFSDTKNPRIIAKELKVIISKLQELYGFVNFIKVNFSILYEYNMKITTISMPLSAFQVVESLRRGEFNIEFTYFFFGCILHFFMPCYYSNLLMERSENFRFAIYSCGWENHNDKNIRQMLLFMLTRATEPLGIATVFTNISLDTFAEMCRQSYTIFNLMNAAWA

>BmorOR32

FRKSLRGSQEEPRANGKSENVRFLINSHILHCGLRFNETNCHTHYIAKVAIFCFIVTYMLQVMELYWSKGDQEKLFECFSILSFCGMGVMKLVILRVYHQRWRFLLNQVSILENRHLDPGPLSYDSDNDNDDNEIVTFITKYTDKFKRTSSILIKMYASTLVIYVLSPFVEYIFRQFRGDLNIAYPHILPAWTPLDEFSVTGYLIMVSFETVACIYCVFVHVAFDLTCVGLMIFACGQFYLLRYRSERIGGKGRICRLLKSTEVRAHYRIVFCHGIHVLLV

>BmorOR33

MIYYRKCKMELNFDKIFKIAIISQKFSGTYPYTKRDKKWATHFILMHGELTIICMLFIYNIIEFDLKAADYSQMCRNMCLSFVYMVITLLYINMLYYQSKLKMLIETMKAEYELAKTMSEEEQNVILEYAKKGRWLCRAWAILTTCGMAQFFLKSIVCTIYSAIQGNFRIVQYYEVICPEVIERHRNNPVIFITLYFCTFFYSLYTSALYTSVLPLGPIFLLHGCAKLEIVRLNIKNLFDNDDYVVQERLKKTVLQMQDIYCYSHEINECFQILYEFLLKATSLVLPITIFAVIQALGRGQFIPEFFAFIFGAFMVGTTPCYYSNMLMEKSEDVRMTLYSCGWETRFDLNTRKCIILMLCRALRPVSIRTIFRSVSLTTLTDVFQQAYALFNLLNAVWN

>BmorOR34

MIYYRKSKMELNFDKIFRIAIISQKFSGTYPYTKRDKKWATHFILMHGELTIICMLFIYNIIEFDLKAADYSQMCRNMCLSFVYLVITLLYINMLYYQSKLKMLIETMKAEYEIAKTMSEEEQNVILEYAKKGRWLCRAWAILTTCGMAQFFLKSIIVCTIYSAIQGNFRIVQYYEVIYPEVIERHRNNPVIFITMYFCTFFYSLYTSALYTSVLPLGPIFLLHGCAKLEIVRLNIKNLFDNDDYVVQERLKKTVLQMQEIYCYSNEINECFQVIYEFLLKSSSLVLPITIFAVIQALGRGQFIPEFFAFIFGAFVVGTTPCYYSNMLMEKSEDVCMTLYSCGWETRFDLNTRKCIILMLCRALRPVSIRTIFRSVSLTTLTGVFQQAYALFNLLNAVWN

>BmorOR35

MKLWQSIREFGLEYCDLPTTLQNVASLLRAITLNIDSRHTARIPFICYVMTVVITLSYFYVFLVSMAWFVFVRSAETRDYLAAMVVLSLGISSEIGTLKFFYTFIYIKKVQRIVREYLECDHMVVPESRFADNVLKTMRNVKKRAILYWVVVIGNGVVYVTKPLFMSGRHHMEDRYIVYGLEPMFESPNYEVAYFLMMFGLCFICYPPANVTVFLIVVVGYTEAQMIALGEEMLRIWEDAVAHYNNKYHTVGALTNSSEKNKIINQYVKFRLTEIIKMHTTNIQLLRQVEFVFRSAIAMGYVFLVLGLIAELLGGLENTYLQIPFALIQVLVDCYTGQKVMDASSLFEQAVYDCKWENFDKSNMKTVLLILQNSQKSMRLSVGGITVLGFSCMMSVMKSIYSAYATLRTTMS

>BmorOR36

MVFNSKKNIISLFSLLEDSRHPSVGPHLRLLSLTGIWYPNSKTNITLLKRACFYVIVLFFVSQYLKCIIKFKIDSLQLILEYAPFHMGIVKTCFFQKDYNVWQDLVSFISKTERDQIAKKDPKSIKTIQSYISRNRKITYSFWALAFIANIGVFSKPYQNNQSDVNGTVTYNHLFDGYTPFSEEPPGYYFSMGIETILGHVVSFYVLGWDTLVVSIMIFFAGQMQMSRLQCSRMINGSPERTHKNIIKCHKFHTDLIKYQKQFNSLISPVMFVYLFVSSINLSVCIVQIAEIEDDFATVLSSFIFLLACLIQLLLFYWHSNEVTVQSELVSYSTFESNWTSTQNKLQKEVALLGLTTSKTLVFTAGSFNHMTLATFISVSLTFELFELID

>BmorOR37

MELGCSRHLKLPCSLHPIGISKHGNTLSELLIYFPAIPKITYAILAVLLTVYYYIYLCSITWFVFVRCPQTGDLAAASIVFSLGVSSEIGAIKLFIIAKLRDITGEYLQCEADMAPGRLRARVGRSLRTVRRRAFVYWLVLVVNAFAYDLMPAFLPGRHLSEDVFVIYGFEPMFESPNFEIASTLMGVSVVFICYTAGSISAFLIVIVGYSEATMLALSDEISCVWDDACASECQQPNDFIRARLGKIVAIHTKQIRLIREVEVVFRGALAGGFACVAFGLIAALLGGLENTFLQLPFCVIQISVDCFVGQRLRDANVAFETAVYNCKWEYFDKSNMKTVLLILQNSQKTMGLTAGGVAALDFTSLMTIFKSVYSGVHHSQTDD

>BmorOR38

MNLSQSVNEQANEYVKMRLERISKIHSPMLPFEDIQDFRELCCIPLAVYAVTGSITASYVYAFLISLLWFLFARCTDPEDFQVAMVVFSLGISSEIGSTKFFNSIIYIKELRKLFKDYLLYDATCPAQGRLRLHLLTTLRYVKRRAIIYWLVIIGNGFIFAIKPLLVEGRHLAQDDLVLIGLEPMRQSPNYEIAYAIMTMGVCFICYPPAHVTMFLIIIVGYTEAQMLALSEELKHLWNDAIEHYEKHSRTEREADAAMKSKILNSFVNFRLVQIIKSHSTNVNLIGRVENVFRGSLAVGYVFLIVGLIAELLGGLENTYLQVPFALIQVAIDCFIGQRVNDANIDFEKAVYDCKWENFDKRNMKIVLLLLQNAQKTVSLSAGGIAKLNFSCFMSVIKSIYSAYTTLRTTMK

>BmorOR41

MMGNSTDLFLDRTKSILNFFAMWRSFEKPIPLKVYMAFIMTTQYLFLIFEIIYIVNVWGDMAEVSEASILLFTQASVCYKITSFISKTNNFVILLGLIESEIFSAQTELHEKILILKARKIKRLCMFFLVNAVTTCSLWAVIPLLDISSKMLPFKIWMPASTGESPHYELGYLYQMITIYISAFLFIGVDSVPLSMIMFGCAQLEIIMDKIGKVKSRPLDQQPMQRQAVLNSNYELLVECVRRYQSVVRFIELTEKTYHANIFFQLSGSVLIICNIGFRIAIVDSNSLQFYSMLTYLVTMLSQLFQYCWCGHELTIRGEELRETLYQSPWHEQDIRFRKVLIITMERMKRPIIFKAGHYIPLSRPTFVAILRCSYSYFAVLNRVRNE

>BmorOR42

MDIPKFEELLKQIQMNFWLMGIPFDNPKIQIRYYVLLLTLSLMLIDEIAFFGSRMSSENFLELTQLAPCICIGVLSVLKILALTAKRQKIYELTQNLECLHKIILNDTRKTELVRKNLVLIKFITKYFFVLNAVLIFVYNFSSPVIIAYNYIVSNEVQFVLPYAVLLPFKTDSWIPWLIVYVYSIFCGFTCVLYYATVDVLYCVMTSLVCNNFSLISFKLQKVNRNTAHLLKEVVKEQQYVLKLAEDLENIFTAPNLFNVLIGSVEICALGFNLMIGDLTQIPGCILFLSSVLLQILIMSVFGENLISESSRIAEAAFLCKWYEMDQKSKKTILTIMIRSHKPKKLTAYKFSIISYGSFSKIISTSWSYFTILRTMYTPPGTKFQDDL

>BmorOR45

MKVLDNVNHAVKVTMNCCRLYGLFVSDDLTKRQLIIMRAFSLMLYLFFVGFFITTQSALIITMWGDLNLMTNVGLVLGTHLTLSAKVFTLHYKEKEITNVIYKNEVRLRAETREQGKYIISEMNRETTLFMRLFIPFGMGTVTAWLLCTPKGELYTPAWYPCNTTKSPAHEIILAHQGIAVILTATLEIAIVLLMTSIVAVCRCRLKLVGLSFETICDDLPSNIMNKLTADEQVIVAKRVRENVIEHQAVLECINDIQDCFSSAMLVHIAISTMIICATAYQLAVEKSLDLTQRMTMASFLGGMSTEIFLFCYQGGHLSIDSMEVATAVYSCPWYTFPTSLKRSLLVIMIRAQQPALLTAGGFAPLLLDTFVSIMKASYSFFTVLQNASE

>BmorOR46

AATYVQIADLIDIWGDLDLMAETSLLLFMELAVISKILTLIFKYDKIMEIINGTEDILCSENRLEGQKIIASIDKETTRFFQYYTSSVIFTTFFWFLGEHSSTFFIRAKYPFNELKSPGYEFALIHQCMMMVFTGYFEFNINIFFASVVAGCRCRLKLVALSLRNICINIPVNKKNLITPEEEKLITERLHCAISQHKYALDAAEDVKHCLSKVLLVQLTVSIVIICTTAYQMAVVRILYWWQEKINYYASLTMAGYLFGTSLEVFLFCYQGEFLRESSEEIADAAYECPWYTLTRPLKKTLLIIMTRAQRPATLTAGGFVTLDITEYMAIMKASYSFFTVLQQVSE

>BmorOR47

MKLVFDNFIFALKVTLNWCRYFGIFIPDELTGRRQKLLVQAYSVFMFMLFIGFFIITQIILFILVWGDLSLMTDVGLVLGTNLALSAKIAVFFFKREELASILKKNDDTLRFETREEGKKIISEIDRETNAFMKVFFCFGVGTVIAWFLSTPKGELHIATWYPCDTKRSPAYEIIMIHQLAITADLLMLSMIAVCRCRVKLVGLYLQTICDDLPCNVKNKLTSDEEVIVAKRIREYVIEHQAILDCISELQNHFSPALLVQLLTSVVIICVTAYQLAVEKSSDLLRKFTMASFLFAMSTEMFTFGYQGGHLSHDSMEVATAAYSCPWYTFPTSLKRSLLVIMIRAQQPALLTAGGFTTLSLETFVTIMKASYSFFTVLQEATD

>BmorOR48

AAACVQIADIIDIWGDINLMAETALLLFMEFAVISKILTLLLRYDRIMEIINGTEEILYFENGLEGQRIIASVDKETTRFLQFNSAFVVLSTTFWFTGEHSSTFFIRAKYPFNELKSPGYEFALIHQCMMMVFTGYTVFNINIFFASVVAGCRCRLKLVALSIRNICINIPVNKKNLITPEEEKIVKERLHCAISQHKCALNAAKDIKNCISEFLLVQFTVSIIIICTTAYQLAVVCLFQNKAIGNIQKTSMFGYILGASLEVFLFCFQGEFLRNAVRDCEEIADAAYECPWYTLTQPLKRTLLIIMMRAQSPVILTAGGFIDLSIREFMGILKASYSFFTVLQQVSE

>BmorOR50

MPSLLKTESLALTLTLNTLSWAGLILRDDYTKTQRIIMKVYGGLVFLYLFVFTAYVQIADLVVIWGNIDFMTETSLILFMQLAVSAKVLTLMLKSKKIMEVTNEADAILNSEKKVEGQRIIASIDKNTTLFLKYYGFFVAFTIICWFMGENTSTFFIRSKYPFNELKSPGREFAFVHQCIVVIFTGSFDFNVDIIIISLVAVCRCRLKLVALSLRNLCLDIPMNKRNLITSDEEKVITERLRNIISQHKRALDAAEAIKHYLSGALLVQLMVSIVVICTTAYQLAVKKSTTMQSLTMAGYLFGTSLEVFLFCYQGEFLRESSEEIADAAYECPWYTLTRPLKKTLLIIMTRAQRPATLTAGGFVTLDITEYMAIMKASYSFFTVLQQVSE

>BmorOR53

MALKKMLALTKGLEDPTHPLLGPTLKALSVFGLWQTGSQKSTVIYNTFHFLTFLFVITEYIDLYTVRKELSKMLNNLSVTVLSTICMIKTLSYVCRQSHLKVLVREISELELELMKTTDKNIVKRLRQYTVYTRAVTYVYWFLVVGINVVLLTSPLLKYASSEIYRSEIKNGTEPPPLILCSWFPFDSARMPGYFWATMVHIIMSIQGCGVVATYDMNAVAVMSYLKGQTSILKDKCKAIFDETASSRDVLNRIRDCHRHHNILLRHYYMFNSLLSPIMFVYMLICSFTICCSIIQLDSSETTISQRIWIIQYSIGQISQLFLYCWHSNEFAAKVKKKHFPLFPINLF

>BmorOR54

MGLNTIKEFFVNVKRRFQDVSIDSLLWIVNIVPSLAGFSIRSDRVSAPFWIVHWSLLVYVYAVGNAVYQWKFANEAIDYITSFINVSLLILIGNNSWWFLANRRLLKSVLHKIEVNDELSRRSEQSRLKHKKLLKIIKRIVLVFYMSNYVNASFIYLPNRVDVLNNYAMTPCVGMEPLTVSPNRELCLTILCMQEFSIMTVVLNFQALLLCFIAHTAVMFQILADEIMALNNYENLEEHQAYVKEMLPIFVKRHSLTLSAVDNYKSLYSVPLGVNFGSNALTILLILYLPVLEWFKFIPIFVFCFMLFFLYCFLCQKLVNASEAFETAIYCCGWENFALREMKMIYVMLHQAQKPVELLAADIVPVNMNTFATTLQAMYKFVTVVKF

>BmorOR55

MCFLKIKQQIIDIQKHFKDYSLNGSLWIVNLLPRLMGFNLRADKVGVFFWTIYILLLVYVFGIGIFVYLWKHVDTMSGLMKSYLNLSLILVIVNNSCWFLSKRSLLNKVLKKIHLIEDLSCESEHALAKYRRVFKIVTHLLLASYVLFYFTEIYFMFLFRNYDLLEDYSLAPCVGLEPLSSSPNSEICLIIVLIHEFISTTVMMSFAALFLVLIAHTAVMFLVLAEDMTKLTDLINLADHRKMIRESLRSLIHRHSLLLQIVYELRLLYSVPLGINFISNAMSILVLLCLPIHEWPSFLHIIGYCFFAFFLYCFLGQNVINASEKFIDAIYCCGWEHFGVAEKKLVHVMLRQAQKPVEIIALGMISVNMNTYVEALQLIYKFVTVLKI

>BmorOR56

MKLLEKLEDPDRPLLGPNVKALKFWGLLLPESRSKKYFYLFMHFAVTVFTATEYIDVWFVKSDLALLLNNLKITMLATVSVLKVTTFLLWQNAWRDLIGYVSRADLEQRATSDSRKLALINGFTGYCRKITYYYWFLMYTTVAIVTVQPIFKFFSSAAYRLDVQSGNGTYLQVVSSWIPWDKNTLPGYLLASIYQTYAAIYGGGWITSFDTNAIVIMVFFRAELELLRIDCAALFDDEKSFGDMAFMRRLKECHRRHTELVKHSRLFDSCLSPIMLLYMFVCSVMLCVTAYQITIETNPMERFLMTEYLVFGVAQLFMYCWHSNDVLYASQDLSRGPYESAWWSRDVKYRKNLYILVAQFNKVIVFSAGPFTKLTVATFIRILKGAYSYYTLLSQSQMNKT

>BmorOR57

MPSLIKNRIFGLTLTLNTLSWAGLILRDDYTKTQRIIMKVYGGLVFLYLFVFTAYVQIADLVVIWGNIDFMTETSLILFMQLAVSAKVLTLMLKSKKIMEVTNEADAILISEKKVEGQRIIASIDKNTTLFLKYYGFFVAFTIICWFMGENTSTFFIRSKYPFNELKSPGREFAFVHQCIVVIFTGSFDFNVDIIIISLVAVCRCRLKLVALSLRNLCLDIPMNKRNLITSDEEKVITERLRNIISQHKRALDAAEAIKHYLSGALLVQLMVSIVVICTTAYQLAVKKSTTMQSLTMAGYLFGTSLEVFLFCYQGEFLRESSEEIADAAYECPWYTLTRPLKKTLLIIMTRAQRPATLTAGGFVTLDITEYMAVSLISNT

>BmorOR60

MVRPCRYFAIHFILLRFLGLGWWHHPHENETRNYPGLYLYYSILTQLVWVVGLVGLETIDPFVGEKDMDRFMFSLSFVITHDLTLIKLYIFYFRNVEIQDIVRTIEIDLYRYYQNDDKIRATIRISRIFTAAFLFFGWVTIGNANIYGIVQDLRWKDIVKNLNETTSKPLRTLPQPIFIPWPYQEDKHYILTFILETMGLLWTGHIVMTIDTFIASVILHMSTQFAILREAIVTAYDRTMIALSEGALQSGVLCENSNGNEENNQIFLESFYSKEHIESVLESTLLSCIRQHQLLIGCVEKFSKTYSYGFMTQLLSSMAGICVVMVQVSQGASSFKSVRLVTSLAFFFAMVIQLAIQCFTGNELTIQAERIADAVMESKWEKMPVRLRRLLLVTMMRAQRPLHLTAAGFAYIDNTCFLSILKAAYSYYAVLSQKQG

>BmorOR63

MKLWIRNANFTISLSLTLLRCLGFWSPDGLAGNKRLLYNCYSFVFFMFLLGIYILIQVVDMIKIWGDLPLMTGTAFLLFTNFAHATKVINIVIRKNRIQRVIQQANAVLMGVQSEEARRIVKSCDFETSIQLCLYFLLTFVTTVGWATSAEKHQLPLRAWYPYDTSKSPAYELTYIHQVAALLIAAYINVAKDSLVSSLIAQCRCRLRLVGLALASLGQDLKIDYQSQLSPAQENILNLRLKTCVLEHQTVLAAVTELQACFSKPTFAQFTVSLIIICVTAFQLVSQTGNLVRLLSMGTYLMNMIFQVFIYCYQGNKLSVESSEIAGSVYFSPWYLGSVKLRRALLIVMVRSRRVAKLTAGGFTTLSLASFMAIIKASYSLFTLLQQVKQKK

>BmorOR64

MGVSNGRGTVKPFLYPLVDELDYNLIVGVHLPFEYKTPSRYPLAYITVVIAFIYVSYFVMVTDLIMQAHLLHLLCQFNVLADCFENMLNDCVKGFEGPLVSLHEYIHPLIDEFEYNLMVGLRLPFSFDTPLRYLFTYVIVLIAFNYTAHYVMVTDLIMQSYLIPLICQYAVLADCFENILIDCSNDYGDHARRNDIVYSRSMELRAILSRPMLGQLASSGLLICFVGYQATTSISVNIVKCLMSLFYLGYNMFTLFVVCRWCEEITNKSLNIGNAVYCSGWESGMTVVPTVRSTILLVILRANKPIVFTAGGMYNLSLTSYTSLVKGSYSALTFLLRIQHE

>BmorOR65

MRLGFEVSISEYLYRNIFYIYTLFHILLHFYYILHMIKLDLEAIFDDIDESVALLPHRDTRRIEVQKILNGRMKRVVTWHISVFKAVEAVSSIYGPPLAYQVMFTSIAICLIAIQITQKLENGILDIRFTMLGVAACLQMWIPCYLGTLLRNKAFGVGEACWNSGWHQTPLGRMIRQDIIIVLLRAQQPVTIKFPGLQSIQLETFSSVIFNLYGYYYFLLLRWVDELTAHLVLSGYWSP

>HarmOR3

MTLSVLDRFYLIDDGFFSFNLKYLFFVGLWPEKTLTRNQKILYKMYEHFISFLTTTFIVLAGIGTYQHKDDLVVVFCNIDRCLVVYNFFFKTIIFFIKRNQLRDLIDEIEMSGDEVTEERKKLMANYVMFITGVTAAVIGAFSLLALFEGTMSIEAWLPSDPMESLMNQILSLEILAFCVFPGLCRAFAMQGLVCSMIMYLCDQLIHLQKELRDLTYVKETEMVMRTKFKNAIRKHIRLMGYSGRMENIFKEYFLVQNLAVTVELCLNAVMMTVVGVQQITLLITFLAYLMLALVNAYIYCYLGNELIIQSQGIALAAYESTWTSWPVDLQKDLLIVILAAQRPLKLSAGGMALLCIQTFSQALYNGYSIFAVLNDAVN

>HarmOR6

MSFRKFLFENEAVDGIKSPSDYLYIKILRFTLDVIRSWPRKELGEPESASFTVFMKYFYLVLTIATVVGSILYVVVHVSELSFLEAGLMYLIILMSFLDALTVMSLTFSAKYRVLAKDFLTKIHLFYYKDRSKAAMEIHKKVHLISHLFSLWLLFQMLSGLSLFNLTPMYSNLAAGKYRRGGLGNTTFEHSLYYLYPFNTSTDVFGYIVACILHWIISYLCSTWFCMFNLFISLMVFNLWGHFKILIITLEEFPRPKSIGTSESAYKYSQEELVEVAERLKDCINYHREIKNFTNRMSDVFGPMLFVYYSFHQASGCLLLLECSQMTAQALMRYLPLTIILTQQLIQLSVVFELVGSESEKLKDAVYSVPWECMDTKNRKMVRFFLMNVQEPIHVKAMGLANVGVTTMAAILKTSMSYFTFLRSM

>HarmOR7

MKIKMSKPLIFDQSIEKLGVLFRFSGMNIKNKIVTPLDTIKYRWLYTLNFLVVFSAIIGSVYYVILGIKQGKNFIEVTSVAPCLTFSILSMIKSLYHLMYEEHIQELIDLLTEHEIRENNREKCIEKEEIIANETGFLNKVINVLYVLNCSMIVVFDMTPIVMIAVKYYKTNEFEMLLPYLDVFSFIPYELKYWPFAYIHQIWSECVVLLDMAAADYLFFTCCTYIRVQFKLLQYDFERMIPDRSISKGLFFEENELRNKFTELLKWHQDIIYSSTILEIIYSKSTLFNFLSSSLVICLTGFNVTIVDDIVIIITFLTFLSMALMQVFFLCFFADLMMTASLEITNSVYNCKWYSANIKVGKQILFVQTRAQEPCKLTAAGFADVNLNAFMRVLSSAWSYFALLRTVYGAK

>HarmOR8

MILFAMNFSFLPKQLDIFVEDLLFYFTDCAAISGILTIVFMREKVCELLEMLESDIFQPDDVEGLAIVEGAGKFIKLYWNIFASVSFTSSAVHLSPIIVHFVIGTELKLPVCSYRFLSENFGQMFVVPLYLYQGSGNMFHMMYNVSIDTFFAGLMVLTIAQLDVLDKKLRRVTDKDEHEDADGETFRQRHDKHREAVRKINQCIIHYEEINKFRRLVQDVFSISLFVQFGMGSCIICICLMRFTMPAPLSYFFFLATYMFLMVIQIMVPCWFGQRIIDKSNFLAFSAYNCEWTSETRQFKSNMRFFVERANKPLSITGGKMFRLSLVTFTSIMNSAYSFFTLLQNVKSRK

>HarmOR9

MLDQFDRCLKSVNLYLKFLGLYLESKDTDKTFVERTRSHRLYFAHLFSLNLEVVAQVLWVLEAVITGKSFVEITRLIPCLILCLISNFKTLSLLYYGRHNNEFIVTMRSLLLNQMQVEEKEHRFRKNLIDKHVLILTSISKKISYVIVLDLLMFALAHAFIIIPHYFKTDEVKLEMPFIAYYPFNEFDLRVYPWVYFHQVYSAVIAMIMVYGPDCFFFTCCTFIHIQFSLLNNDMERIVTEETPRYDKTKFKKLAVRHIELMRCVNLLEKIFSKSILFNALTSSVIICVTGFNVLVVDNIVMMASFTAFLIFGLMQIFLYCYYGDTIMRSSMEVSTSIYNSLWYNIPAADRKGFLIVIIRAQKPCALTADGFFKMNLSAFASILSKSWSYFALLKTMYHPE

>HarmOR10

MAVKNTSLFLGRPKKILSAHGVWPHPNNFVILRKLYMLFVMWTQYSFLLFEIIYIADVWGDIDAVSEASYLLFTQASLCYKSTAFMVNKQSLLELLEIMDCEIFEPKSAEHEKILAAQARKIKRLCLFFLTSATTTCTLWAMIPLFDAASKRSFPFRIWMPVTPLKSPDYELGYLYQMVSIYISAFLFISVDSVAVSMIMFGCAQLEIIMDKIQKIKYVFESADSEEGRRNIIKTNNEFLVECVKQHQTVERFIQLCEDTYHANIFFQLTGTVAIICNIGLRISIVEPNSVQFFSMLNYMVTMLSQLFLYCWCGHELTIRSENLREWLYQCPWYEQDTEFKRALFIAMERMKKPIIFKAGHYISLSRPTFVAILRCSYSYFAVLNRVNTE

>HarmOR11

MHLAGNAVTGITGPMDYKYMKVLRFVLRIISGWPGKALGEKTLRIEGMGHAYYNTILSLVYLALGIAYLKKNFHRFDFLELGQLYIVLLMNMLSTSRAFTLCLSQKYREVAKIFIQKIHLFYFKEKSDFAMKIHITVHKISFISAVYLSVLLFIAACMFNLIPMYNNYSAGRFASFDNLENTTYEQAISCLYPWNFETNFNGYLAATLSGWYGTILCGSSVSMFDLFLCLMIFNLWGHFKILIYNLEHFPRPASEVVDAEGEERSGRTVGSEMYSQSELEEVAVLLRDCIQYHMLIYNFTNNMSDAFGMALFIYYSFHQITGCLLLLECSQMTAAALTRYLPLTIIMFGELVLLSIIFETIGTMSEKLKDAVYKVPWEYMDTKNRRTVLIFLIKVQEPIHVKAGGLVDVGVTTMASILKTSFSYFAFLRTF

>HarmOR12

MEDEPLLIDKTVKNIEFLFRCTGINIKSGTKTRKDMIKSRTVYIINFLWLNIDLAGAVMWFFTGIANSKSFTELTYVAPCITLSFLGNLKSLFLILREKHVDKLIQVLRDLEINEKARPKSEETDAIIKYEHNFVTTVISVLNVLYFVLLVAFALSPVSLVALKYFTTNELELLLPFLIVYPFDPYDIRYWPWVYLRQIWSEVVVIIDICTADYLFYTFCTYIRMQFRLLKHYIERVIPEDDGGGRLTNIEQVRAEFVLLIKWHQDLISSANMLETVYTRSTLFNFVSSSVLICLTGFNVMAISDVAFVATFLSFLFMSLLQIFFLCFFGDLLMTSSTEISEAVYNCRWYLADTSLGKDLLLVQTRAQTPCKLTASDFSEVNLKAFMKILSTAWSYFALLQTLYGAPT

>HarmOR13

MKILSDGSDLEGVEKVEDIFYINLARKSMWILDSWPRTPNESVTYRYFVLALNVATLVGGAVYLRNNTGVLSSFELGHTYITVFMNCITCSRCIMILSREYNEVMLSFVNKIHLFHHRHKSEYAYKTHIFIHKISHFYTVYLLGLALNGLLLFNMIPFYNCYSRGMFRDVIPANATYDHSVFYSVPFDYTTKFKGYIAMTSFNCFISYTCTSYFCVVDLTVSLVIFHLWGHMRPLTYHLANFKKPASVLESNENTDAIKDHSYTQEELKEVFGKLREYIRHHNLILKFSSEMSNAFGPALLAYMVFHQVSGCILLLECSQLDMKTLVRYGPLTVVILQQLIQISVIFELLGSSNDKLIDAVYLVPWEYMDTKNRKLVFVMLRQSQRSIDLKMMSMLTVGVQTMTAILKTSFSYFVMLKTVAEEE

>HarmOR14a

MGGIRDFIFNLEAKEGITKPTDYPYMILCRHLLTVITCWPKEPKEGLDTRAKLKARIWVTFQKIFHLNGCFITTIGMAMYIALHKNSMSFFELGHLYISLLMTVVIFSRVTTLCWNPEYQAVATDFLTKIHLFYYKDDSDFSMQTHKQVHKISHLFTLLLTGQMVAGMSLFNLTPMHNNFSTGKYKKGGLKNSTFEHSLYFSYPFNASSDVRGYILSNIFHWIISYLCSTWFCTLDLFLSIVVFHVWGHFKILIHDLNHFPRSLNTISFRLDQSNITLTTEMYSSRELVQVSERLNKSVEYHRRIVSFTDKMSEVFGPMLFVYYGFHQTSGCLLLLECSQMTVEALVRYLPLTIILFQQLIQLSIIFELVGSVSDKLKDAVYGLPWEDMDTKNRKTVAFFLMNVQEPVHVKALGLADVGVTSMTAILKTSMSYFTFLRSM

>HarmOR14b

MAGLRDFFFNYEANEAITTPKNYPYLIIMRISLSLIKCWPKKTTENLAAGAKMKAKVWGMVQNVLHLAFCVLTIVGTATYVMIHKKNMTFFELGHLYITLMLSCVVFSRLATLTFNEEYQVVANEFLNKIHLFYYKDNSEFSMQTHKQIHRVSHLFTLYVTGQMLGGLSLFNLTPMYNNYSAGKYSKGGLKNSTFDHSLYYSYPFDVSTGVRGYIFSNILHWFFSYIVSTWICTLDLFLSVIVFHIWGHFKILLHDIDNFPKPSKMVSFKLKNTNVTISNENYSTEELEQLADKLKKCIDYHREIISFTNKISEVFGPMLLAYYGFHQASGCLLLLECSQMTPEALARYLPLTLILFQQLIQLSIVFELVGTVSSKLNDAVYGLPWEDMDVKNRKTVAFFLLNVQEPVHVKALGPADVGVTSMTKILKTSMSFFTFLRSM

>HarmOR15

MTFYELGHLYISLLMIVCTFSRITTLCLNDEYRVIAKDFVTKIHLFFYKDRSDYSMETHKKVHMISHIFTLYLSGQMMLGLFLFNVTPIYNNYSAGKYTSGGLKNSTYEHALYFSWPFNASTDFRGYVVSNILHWLLSFSCSSWFCVVDFFLSLMVFHVWGHFKILLHDLDHFPRPANKISFILEDSYVTITDEIYSRNELNQVFDRLNKCIDYHRDIVSFTDKMSEVFGPMLLAYYGFHQASGCLLLLECSQMTVAALVRYLPLTIILFQQLIQLSIIFELVGSVSDKLKDAVYGLPWEAMDTKNRRIVAFFLMNVQEPVHVKALGLADVGVTSMTAILKTSMSYFAFLRSK

>HarmOR16

MGLRQFLFENEAVEGINSASDYLYIKVLRFMLLIVNSWPRKEIGEPESPKLSAFVKYFYLVVTVLASAGFILYLVKHNSELTFLETGHMYIVLLMSFNDVSRVATLTMSTTYREVARDFLTKIHLFYYKDRSKQAMETHRAVHKIAHLFTLWLVSQMLSGLSLFNLIPMYSNYAAGRFSGEVSKNSTFEHSMYYPYPFDTSTDIRGYSIACITHWIISYLCATWFCMFDLFLSLMVFHLWGHFKILNYTLNDFPRPSSKVEAAKYSDEELVEVAARLKDCILYHREIILFTDRMSNVFGPMLFLYYMFHQASGCLLLLECSQMTAQALIRYGPLTIILTQQLIQLSVIFELVGSESDKLKHAVYGVPWECMDVKNRRSVVIFLANTQEPVHVKAMGVANVGVTSMAAILKTSMSYFTFLRSL

>HarmOR17

MFLRSECARSVAPHVRVLRCVGFLRGAALSSRGRAERLALRSYHALALAATSLYVLQQAVYAYQERGDMDKPSQVMFLMLCHVTCVVKQIAFHVDADRIDRLIASLDEPLLNQCAGERGALLRGTARGAARLLRTYAGCAVATCVLWIVFPVINRIQGISFEFPFWTGFSYDHNAVFTLVLLQSFYCTNLVAIGNTSMDAFMATILDQCKTQLRILRINFESLPERARALHVESGENYDTILDKLFVDCLVHYNKITEMCTELHDVFAVPLLVQFGVGGWIPCMAAYKIVSLDVLSIEFASITLFITCILIELFIFCYYGNEVTVESERVSQSLYSMEWRRARLTFRRSLVLVMERAKRPLRPAAGRVIPLSLDTFVKILKSSYSFYAVLRQTK

>HarmOR18

MEMKVDVLPEKKYKGFNETFKLCAFSLAFAFLYPNRTTALRRCITITLIVTFCGGQLFWFITYTFKCLYTLDIYNFARNMTLAVVLVLFFIKTYYVIYATSKFAPLLDKISDDLLEANNLEEEFQVLYDDHIKIAKVGEISWLLIPTIMSALFPIYAGALMTIESIQTDDYERRMVHDMELLFVEDIQSETPFFQCMFAYNCVQCVVLVPNYCGFDGSFCIATTHLRLKLKLMTLKVNKAFKYSKSRQELRMRLYDSIKDHQDALDFYVQLQNVYGPWLFAVFLLTSFMISFNLYQIYLLQRIDPKYTSFGVVGVLHIYLPCRYASDLTRVSEEIPDDLYLAQWEAWADPSITKLLMFMITRAQKEMIVTGMGLVVFNMEMFKSILQTSYSFFTLITA

>HarmOR20

MDEELEFKPFHETYRLITFSLCIAMIYPNPRTEKWRLFSIPILIATVAPVAIMIFLDMYKCWKNGDIVNIIRHSTVVGPFLGGFFKMILMYHKRVQAKQILDEFDRDHLMFNTVAETYKDIARASIRNCQIYSERLWACLVTTCVMTFPVMAIVLNIYNFMFKSEPTKYMIHDLEKPFSKEPEERFESPYFELLFVYMFYAAILYVVNFTGYDGFFGLCVNHARLKMELYCKALEEAMMADREEVYGRVIAVIREQCRMFRYVDLIQDTFNIWLGIIFIATMIQICTCLYHITEGYGFDIRYMIFVYGAVVHIYLPCRYAAKLKAMSMETSNRFYCSGWERVDDERVRKMIVFMIARAQVPNEITAFNMMAFDMELFLSILQTSYSMFTLLRS

>HarmOR21

MIKRSKFFSWALILNCVLSLLMYTVEAVMRVIRAGATYTTVITAYPDVEDRSALSHVVRVIAYIIWCIYLTRIFAVYSLVISLTIAMSYQFKNLTSYFCNLSKIFEDERMTQTEKEQEYERAFRVGIKIHSETLKCTEDIQAICRDVFSGQIIFNILMLIVLMHQMVNSARNLTNAVTLVMAALTILLSTGFFMWNAGDITVEAQLLPTAMFSSGWENCGRDSSVRVRKLIVIAMMQAQEPVVLTGLGIIALSYQSYVSVSTFTK

>HarmOR21.2

MTKFLDELNTIFFLVGLTDLWISEVKFSKRFIQIYKKINYVMDFLCLFFVVFLLGSYFTQKDLTEKLANDRLMFSIILPGNLVFYYISVYYKEEIRNLLYHHRVLKEQHNDTRLEREMIRNIRVFSITLNSIAFVVDTSYGFGALYEVVTKGENFNTIVPVWPDVHDNSSLAGAMRVFFYFCWLNPIATRVLTTFSLLLTEMVAVCYQFRNLQSYFYSLDDIFSDDTLSQKEKEIKYEEGFKIGIRMHIMTLWCKKLHQHVNKEILAIEMVLFFAMLMSELTTLLGGERNASQLCMMFLISVSTCISLGFFMWNGGDITIEASKISEAMYSSGWQHCRGHSSVRMRKLVTFAIKQAQDPVVYKTLGVVDLSHTSYVTLVKMPYSAVSVLY

>HarmOR22

MMAIHVIMVIFCFSCTLSLWTQKNLSESQQSDRLAYGASAPIITIFYHFVILCYKDDVRKVLYKLVVVLKVDHNDKQAEREMMEQSRLHNGLFFSSCVCNMVFVGLYNCYLAVTTDATFITCISAWPDIEERSLPAGLTRVVVYFVWFAHVVRNMGVFLIIHTVLLCLTQQYKNLQSYFEDLNKIFDETKLSQEEKELKYEIKFKRGIEQHALTLWCVDETQRVFKITFSSHVLLWCGLLISILPDVMNNDDHTLKMLVSNAPRVAAALVGLGYFMWPAGDMSVEASNLPQAMYGSGWQCCYHRSSRVRKLVVLAMMQAQRQIEMKAFGHLTFSYE

>HarmOR24

MDKLARVMFLLLCHITSIAKQLVFHLKAERIDEMLAGLEDPLYNQPEEAHRRLLGATAASASRFVRAYSGCAVVTCTLWITFPVMYRLQGLPVEFPFWITVDYNRPTMFILVLAYSYYVTTLVGIANTTMDAFMATVLNQCKTQLRLLRMNFECLPERAAALSRQLGGSYDAALFALFRECLVHYEKITETAKMLQNIFGTAILIQFGIGGWILCMAAYKIVSLNMLSVEFASMALFISCILTELFLYCYYGNEVTDESERVSQSLYSMEWRRARLTFRRSLVLVMERAKRPLRPAAGRVIPLSLDTFVKIIKSSYTFYAVLRQTK

>HarmOR25

MPSDQSRMFDPPLTVLKIFGVWEGRTPSKYYKTFSFLFLFVSWFFYNFLLTLSLVYTPRSVELFLRELMFYFTEISITSKFLTVLLLRNKILEVFSVIDSDEFVGDYENKDGILYRTNKGYSLCWKVYNVLANIDYTCVIIMPVVIDLIQGTKSVLPICNYYFLSEDFRDSHFVILYLYQSIGMYGHMMYNLNMDSLAWGLLAVGIAQIKVLNKNFTDLKLSAEESKLPLEIQDNIQKTRLFKLLRHYEAILNYCDAIQNLLSVTFFFQFSFGALTTCVIMCSLLMPGTMVYRIFLVIYLFAMAGQIAVPGFFGTLLTHESQELVTAAYNCEWIERSQSFKRTLILFRERAGTPIIISGMKMFPLSLVTFVAIMKTTYSFFTLIRNA

>HarmOR26

MDCTIVAFYAQAKIQIQMLRHNLEQLVEFDDSAKINTQFNKTGLYSTSYKDEQQERVAIQERLKKCVQHYYQILRFAKEVESIFGEAMVVQFFVMAWVICMTMYKIVGLSIYSAEFVSMAVYLGCMLAQLFIYCYFGTQLKVESELVNQSIYCCDWMKLSPRFRRQLLVMMQCCGRPIAPRTAYVIPMSLDTYIAVLRSSYTLFTFLNR

>HarmOR27

MGKDDPDRLFECFSVLSFCAMGMLKLLSLRKNHRKWRKLLTQITILENTQLSNRSISCVEYQSDSEDSDNFSEHISIYTKKFRGTSIVLTRIYSFTAFLFILSPFAERIICEIRGVECVGYPHVFPGWTPLDDFSIFGYLVTVLCEVFSAVYCVCVHMAFDLTVIGIMIFVCGQFSLLRDYSSRIGGKGRQCNLSMRRDERARFRIIRCHDINLLLVNSITELDMLLKNIIGVYFFVATLTLCSVAVRLKSEDMGVMQLVSLIQYMCGTLTQLFLFCRYGDAVLHESTMGMGEGPFAAASWCLSPRVRRDLSMLSAGMMSQRHLRAGPFSFIDLPSFIQVVRAAYSYYAVLGKKE

>HarmOR29

MGYQQIDCFDIHLKILRILGVWPHDNPSIYYIYFSRIFVFTFTVLYVVIYTMNFYFLPQQLEVFADELIFYFTNVGALSKALAFIFLRDKVKKMLFMLESEIFQSDDPEEIKLIKEGKEKSNFYWKITAGLSVSANTVNVCLPLLVHIIFSVELEFPVCRYSFIPEKYEAMFAYPAYFYQSIGITTHMLYNVNIDTFLLGVMFLAMTQLDILDRKLRKVTDVCINPDAARGSVDKFIDDQNAVLEIIKCIKHYDAICEYCKLIQDAFSEILFVLFSSGSCKICMCLFRFTMPATTGYFVFLYTYVTVMTLQVMVPCWFGSRLMDKSSQITIAAYDCDWTPRCRRFKSNLRLLVERANRPITIIGGKMFLLSLGTFTAIMNSSYSFFTLMRHMQSR

>HarmOR30

MVSSQITCGLWAMKPLFDDADRKFPFDMWMPVSPEKAVQYYIGYAFQLGTICISAYMYFGVDSVVFSSVIFGCAQIDIIKEKLMSITTVDRKQGTKEALAQNYNKLVDCIKHHQAIVTFTELVENAYHPYLLFQLVGSVGIICMSALRILVVDWRSMQFFSILTYVSVMISQLFVCCWCGHELTATSEDLHTVLSNASGTSRT

>HarmOR31

MNSILQNLEDPNRPFLGPNYWIIKNMGLLLPKNFLAKILYIILHEIVAFFVITQYMELYVIRTDLDLVLTNMKISMLSVVCIVKVHSFIFWQKHWHDVLDYVTAADKFERQSDDPIKSRIVETYTRYCRRLTYFYWVLVFTTFLTTTGTPLMRYLSSSTFRQNMRNGTEPFPHIFSSWMPIDKYHSPGCWITVLWHTLLCAYGAAIMAAYDTCIVVIMVFFGGKLDLLRERCKQMFGPSTISDRQCEEVVRQLHGIHVMMLKYSRLFNSLLSPVMFFYMVMCSLMLCASAYQLTSAQNAAQKLLMAEYLIFGIAQLFVFCWHSNDVLIKNENMTSGPFESNWFLANYRQRKDVLTLSGQLCIKNIFTAGPFANLTLPTFINILKGAYSYYTLLRK

>HarmOR32

MNKDFQTSKDLPPKERDSIKKYINQGLWVCKQWLFLTISGCAIFLFKNLGLMLYYYCMNEFRLVPFYEVVLYPPIMEENRDNIFVYLLMYAIMLLFSAYSALMYAAFVPLGPIFILHACGQLVLVKLRIDDLFVECDDEVIRKKLKGIILHLQYVHSFVDRIQQVFKIGYELTLKFTALILPITIYAVLEGFYRGEVNVEFVTFIVGGVMISGSPCYYSDLLMEKGEDVRMSLYTCGWEQHYDRRTRTTLQLMLQNALKPIAIQTVFTVMCLDALTDLFQQSYAIFNLMNCMWN

>HarmOR33

MWRYIRKFGLEYCDLPTMLWNVSVLLKVLTVNIYGKNRKAIPLIFYIIVTVGLLTYFYVYVVSMIWFVFSRCPVTGDVLAALIVFSLGVASEISTVKFLYMRIHIKDVRKMVADCLDSYSKVVPGTRFSNNLLRTLREAKRRAMLFWMVIIGNGLMYVVKPLLLPGRHFMDDVVLLYGLEPMFETPNYQISFVLMGSSCVLICYLCANISAFLIIITGYVQAQMLALSEELTHLWEDAEENYRGNELEDITDDGDQNDRNKDAILNDYVTVHLKDIAKSHAENINLLGQIEGTFRGAIAIEFCLLVVALIAELLGGLQNTYMEVPFALMQVGMDCLIGQRVMDAGAVFEDAVYDCKWERFNKKNMKTAMVLLLNAQRPMTISA

>HarmOR34

MSAKFSIMQIFKFLEDPAYPSVGPHLKLLGFTGLWHPNRHTLVGRFKHILFYITISFFFSQYIKCFINFNASSLKLILQYAPFHMGIVKSCFFQKDYKTWQQVIDYMSSVELAQLSKSNKEQYKIIYDYIKRNRKVSYFFWALAFFSNFSIFTEPYQKNQINVNGTSIYLNIFDGYTPFQKEPPGYYISMLIQTVLGHIVSAYVVGWDTLVVSIMIFFAGQLKITCLRCKMMIDVTNPMKSHLKIAECHRFHTTLVEYTRIFNALISP

>HarmOR35

MCLYLPFDLVIVIMTSNVSALLRLLQVDLKNAIQLRDEQHKTKSHLNVSDTQSYEELKRIVDIHQRLLRIADQLSSIFGLVIFIHVACAALEICFFGFLTMVYGGLAETIANMLTVLNAVFTIFLLSLSGQFLCDTSSEVADAAYESYWYESDHKVKKLTLSIIIRAQRPSYLSALGFSQLTLKSFSKIMSSAWTYFSLLIQMYEET

>HarmOR38

MIILSENIKQKLAFLSPYLPYGVIESWEDLNPRLYHAVHIYWLKFYGMWFNNYSPNNIKFWLHMVYTLTVLWLACFFPGIGEVVYLLKQRENIGDIADGLYLFLSEMYTYVKIAVFWMNRDKVISLLEYLHCKEFKPKEPEHRDIITKSIKSARFVMTYYSTMCVGAVSVGIIMPLTENFDILPTNVEYPFFNVYRSPAYEAVYIHHIYYKPATCIIDGVMDTILAAFVASAIGQIEILAFNLRNFNLVAERQRRRDLAQNKYIEEYPAQHYVRSVLKECIRHHNCIIRYVSMIESAFSLASALQFMLSVMVLCLIGIQFLSIENPSAHPMQIAWMGIYLTCMLIEVFILCWFGDELIWKSMDLAKAAFEGPWMNSDRQTNMFIIILLERCKRPLRLSAGKIFTLSLDTYTVLINWSYKA

FAVMRNMKK

>HarmOR39

MHRKRKEFNDLLEVAQKNDDLIIETGRFLHVHEKMLRSIKIIILFCYVFHFINEVVVYIPFRILRMEDFSIASCVGFGPLNVSPNREVCMGLMTAHILISIMVICCYDISLLFLFSHTTAVFQILFEEMMSINDITQTCQNSDEDYAVIVARLKNVIVRHVLALQTVGKVEDIFSVSIGICFGLDAISLCLILCVATRSLYAFCSDFTIAFSIFFLYCCQGQRLTTASEKFEMAVYCCGWENLRVKERKQVLLMLKRAQKPVIVYAAKVIPIRIYTFASTMQAIYKFVTIFKV

>HarmOR40

MAMRNLSLTMLSTVCVFKACNLMLWQNSWKELIDYVSELERSQLSKNDPVVNKIISDYVKYARRVTYLYWALVTATVVTVILAPLFIYLSSPNYQESIKNGSAPYPEIMSSWTPFDRSRGLGFCGATLYQMLACFYGGTVVANFDSTAVVIMTFFTGQLKVLSVNCERLFGDGNELVDYDEAVKRITECHLHHYYMVKFSSVLNSLLSPVLFLYVIICSLMICASAVQLTTEGTGNMQRIWIAEYL

>HarmOR42

MTNSRPRHYFGFHYRILRFLGLGWWHHPEEGKTTNFPGWYLYYSIVTQVVWVAGFVGLETIDPFVGEKEMDRFMFSLSFVITHNLTLIKLYIFFFKNVDIQEIVRTLEIELYDYYQNIEKNRKTVKISKIITGSFIFFGWLTIGNGNVYGTIQDLHWKSLVATLNDTSQIPVRTLPQPIYIPWNYQKDKSYIPTFVLETVGLLWTGHIVMTIDTFIASVILHMGAQFEILNEAITTAYDRTMTSLREGIRPEDSGHQGQQSSILSVEDSNERIVHAFIPKEEIDAALQTTFRNCFRQHQVLINCVEKFSRTYSYGFMTQLLSSMAAICVVMVQVSQDASSFKSVRLITSVAFFFAMITQLGMQCFTGNELTLQAERISDAVMQCKWERIPTRQRRLLLMMMMRAQRPLRLTAAGFTNMDNACFLAIMKAAYSYYAVLSQRQE

>HarmOR43

MVKNENRSLQYCLTVLKVAGFLHPLGDGRIPRLTRRLYCFGVFMFLVGCIIMAQTGAMFEIWGDLALMTSASFLLFTNLAFATKIINVVVRCREIQEIIDEGDADLLAEDRYLGIEVIKSCNVETSLSMGLYTLLSGVTVFGWAASAEKNQLPLRAWYPYDTSKSPAYELTYIDQSSAVTLAALVNVCLDTLVTSLIAVCRCRLRLVALSLRTLCDGIPLPDKQLISPTEERIVLTRLSQCIIKHEAALKAARQIQRCFSLPILAQFAVSVVIICVTAYQLAMELNNRNWFRCIPMVAYLLCMALEVFLYCYQGNELLEESSEIAGAAYECPWYHCSVRMRRTLLIVMVRTRRALRLTAGGITTLSLACFTSIIKGSYTFFTVLQQAEDRNPK

>HarmOR44

MGYMIYSSSARMLSALVVICEIWHALGNNMSLDELISSVNVIFIHLITLWKLMIMVSNKKVFKKLARALESPSFDISTENRQAIVNHWVLTHKKYLKVLLCLAYLTLAVWVLHPLVDDMDFNLMVDVKLPFAYDSPLRYVISYLFVGTMFSYASSMVIMSEVIMQAHLIPLVCQFNVLANCFENVFEECASEFPDINKHELVKHNMFVEKYRKRLGDLVKQHREILDQTTDLKTILSAPMLGQLACSGLLICFVGYQATATIAENLGKFVMSLFYLGYNMFTLYIICRWCEEITIQSQRIGQSAYFSGWESGVSHVPGARATIILVIARSNKPLVFLAGGMYTLSLTSYTSLVKASYSALNILLTTKHE

>HarmOR45

MRLQILEDFLLKKTFDFDRPDINLYNFHPQLRILLAVKGVFFTNRRSLLRFIWPCICIQLSIVAMTLEEIFIWRGVTVKDYSFATECFCYWVILGCIPMVYVSIVVNTNKIYDIVVTMNEDFIYVCSLGDRYRKPFLEGQLLIWQLCYAWFIFVCFVGGLYVIIPLVGLLYQSLFATIDENTVRPLQFPMWLPNDDPYRTPNYEIFLVIESTLIFCFVQTFCVYIYTLLHILLHYYTIMNMIIIDFSVIFEGLDESVALLPRHDPRRRETQLILNARIAKIVRWHLSVFKAVSTVSSVYGPPLVYQVSFSSLAICLIAYQIAEKLDNGKVDILFCLLGIAACLQLWIPCHLGTMIRNKAFEVGDAGWTCGWHETPLGLMIRNDILIIILRAQKPVTIKFTGLPSVQLETFSSTMSSAYSYFNMLRQYSK

>HarmOR46

MSSLGTIADSLPLLVSLIIVAYYAMYRQDLYDLMEYMERNFKYHSAGGLTNMTMEESCKTAQRFARIYTACTMFSVTMYATMPVIIHLWTKEPIQSWMYMDITRSPFYEFVFLVSCLAQMFVGLAMGQFGVFFASNSILICGQLDLLCCSLRNARYTALLQHGVKHAALRVSHATIQDDEKHNYIYNVSEMKESVYHYDKKVSNLYAEAKTQFDIYSSEFDDATVNALRDCASLCQVINRYKEMFENFVSPLLALRVVQVTLYLCTLLYAATLKFDMITVE

>HarmOR50

METTTYTRSKTTEFFYKMNFAIYIFGLPNFWIEDLKLSKRFVKIYDKISLFNDLLVYLLLVMEFGAFFTQHNLTDKQKFNLMVFAISHPLLCSFCVMVSKLKKKVRLVMYSQAVALKRDYNDPEVEKQMIARSLTYVLAFMSSCTITMIMFAIEAIWDVIRHGATFTTLITAYPDVQDRSILADVVRVLAFVTWWIFLTKMVAVYMLVIPLTISLRYQFKNLQSYFLSLAELFERSDLSQKEKEEKYEAGLKLGIKLHSETLSCAEDTQDVCRGVFSGQIIFNILLLIVLMAQMVTSERTFVNMFGTVATSCTVITSTGFFMWNAGDVTVEASYLPTAIYFSGWQHCQRDSSMRVRRLVVTCMSHAQQPVIFKGLGYIELSYQSFITIVKSSYSVFSVLY

>HarmOR52

MRTLREIGQEIRKFGLEYCDLPTMFENVAILLRLLTLNIDIKYKGGITFYSYIITIVSGACYYYVFFFSMTWYVFWRSRELGEDIGAMIILSLGITSEIGPLKLFYMSYNKDKTQKIANDFLECDANTIKSTRFYANLLKHCRTVKKRAMLYWIVVAGNGVIYLLKPITMKGRNLPENYFLIFGLEPIFETPNYQIAYCMMVSALFFVCYVPACVTAFLIVVTGYAESQMLALSEEMIQLWPDAIKRAEERTQLDPSKVLDVYNLEVKTIMNQFVEKRLKEIIKRHALVINLLNQVEIVFRQAIAMGFVLLIVGLLAELLGKLENTFLQMPFAFMQVSIDCFAGQRVMDASLVFEKAVYDCRWENFDKANMKLVLVMLQSSQKTLALSAGGISTLSFTALMSIYRGLYSSYTALRSTVK

>HarmOR53

MSEKEFDKTLKLTNYALIMSGIKTSENDMNKALEYFINHYLFYCNAIALHTVIFGEVYWIVDGIRTNHPFVELSLVSPCATISILSTIKCGFIFSNKGILMRVVHKLKEIHTSFDDNELSKESSARTKIVTDSLKLLQFVQISFATIYIFVFFSFCFIPVILAEYNYYRTGEFVVTYPFFVKYPFDFDVHHCPVWQLIYFHQVWATAIVIMSMFGCDSLFYGLCVYIKTHFQLLGLRFENIVGATKSETQRNLAKAVVRHQELIDLVNQMEMLYSKSSLVNIITSSILICLSAFNITVVDKLNVILAFVTFLVMSLSQISLVCYFADLLMAASMEISGSVYRSPWYEADNHSKKILLLVIMRSQKACKLTAWKFADLNLGAFTTILSRSWSYFALLKTVYK

>HarmOR54

MLTASSRLKTVFEMSHHGLSLIPEGTASKKIMLATLQQARYFSWLVVANLAVTHVTYLLMPFLFTVLGNDRYLPTTPGETYGLSPKYETPFFEITFVLTSVATAFSAINQTGYIVLFVTLICHELGHFYAITEALHEIHTILTKEERSRNNSEQNEQKSVDKLLIFCVKHHQFLMNFHGKIRDMYKVIFGAHFLSMTVVLVTTLQTMNVWDYRNTILTGMSGIMPLFLYCFGGEKLISAGLQMSSAAYSCGWEMMEAKQAKVVLLMLCLVQRPLYLTAADIFIMNRETFGDVAQVVYKIYAVFN

>HarmOR60

MGVLVRNATMSVSISLTALQFVGFWAPEYLGKTQKQLYTCLSVFSFMFLLGTYLIIQVVDLFLIWGDIAMMTSTAFLLFTNMAQAAKIVNIVYRKERIQRIVNDCDAVLSRAQSLEEKEIVKSCNREMIVLQILYFSLTLITSLGWATSAEPHQLPLRAWYPYDTTKSPAYELTYVHQVGALLIAAYLNVAKDTLVAALIAQCRCRLRLLGYALRTLDKGMGNEAYTFTSEQEKTLNLRLGSCVMQHQKALDVGKELQECFSEPTFAQLTVSLIIICATAFQLSMGHSDNMVRLLSMGTYLLNMTFQVFIYCYQGNQLSEESSEIAGAAYECPWYKCSVRVRRGLLIVMVRTRRALRLTAGGFTTLSLACFTSIIKGSYTFFTVLQQAEDRNPK

>HarmORco

MMTKVKAQGLVSDLMPNIKLMQMAGHFLFNYHSENAGMSNLLRKIYASTHAILIFIHYACMGINMAKYSDEVNELTANTITVLFFAHTIIKLAFFALNSKSFYRTLAVWNQSNSHPLFTESDARYHQIALTKMRRLLYFICGMTVLSVISWVTLTFFGESVRMVTNKETNETLTEVVPRLPLKAWYPFNAMSGTMYIVAFAFQVYWLLFSMAIANLMDVMFCSWLIFACEQLQHLKAIMKPLMELSASLDTYRPNTAELFRASSTEKSEKIPDTVDMDIRGIYSTQQDFGMTLRGAGGRLQNFGQQNPNPNGLTPKQEMLARSAIKYWVERHKHVVRLVASIGDTYGTALLFHMLVSTITLTLLAYQATKINGINVYAFSTIGYLSYTLGQVFHFCIFGNRLIEESSSVMEAAYSCQWYDGSEEAKTFVQIVCQQCQKAMSISGAKFFTVSLDLFASVLGAVVTYFMVLIQLK

>SfruORco

MMTKVKAQGLVSDLMPNIKLMQAAGHFLFNYHSENGGMTGLLRKIYASTHAILITIHFACMGINMAQYSDEVNELTANTITVLFFTHTIIKLGFFALNSKSFYRTLAVWNQSNSHPLFTESDARYHQIALTKMRRLLYFICGMTVLSVVSWVTLTFFGESVRLITSKETNETLTEIAPRLPLKAWYPFNAMSGTMYIIAFAFQVYWLLFSMAIANLMDVMFCSWLIFACEQLQHLKAIMKPLMELSASLDTYRPNTAELFRASSTEKSEKIPDTVDMDIRGIYSTQQDFGMTLRGAGGRLQTFGQQNNNPNGLTPKQEMLARSAIKYWVERHKHVVRLVASIGDTYGTALLFHMLVSTITLTLLAYQATKINGINVYAFSTIGYLSYTLGQVFHFCIFGNRLIEESSSVMEAAYSCQWYDGSEEAKTFVQIVCQQCQKAMSISGAKFFTVSLDLFASVLGAVVTYFMVLVQLK

>SfruOR50

METTTTSYTHSKTTNFFYKINFIVYIFGLPNFWIEDLKLSKRFVKFYDKFSMFNNTLIFLLIIFELCSYFTQSDDQLTEQQQSNRLIYAISHPMLFMFRVMMTSIKERVRLVMYSLNVGLKRVHNDLEVEKQMIACTFMYLSALLLSCLMSMLMYAAQGFGEVFRRGKTFTTIITAYPSVEDDSDMANVVRAICFIIWWIFLTRIYAVYMLVISLTTCLSYQYKNLQSYFVSLNDIFERSDLSQTEKEEQYEAGFIVGIKLHADTLRCTQLTQSVCRGVFSGQIIFNILLLVVLMAQMANSERTLVNLCSAGFTACAVLISTGFYMWNAGDVTVEASHLGTAVYFSGWYHCQGPSSVRIRKLVVFTMSQAQRPVVLKGLGYIDLSYQSYIRIVKSSYSVFSVLF

>SfruOR53

MFLKKVIKYTKRLEDPKNPLLGPTLKGLYLFGLWQTGGKFRTVVYNLIHLTTFFFVTSQFVDLYFVRHDINKVLNNMSLTVLSVICLAKCFSYVFWQSEWRKLAQSISEEELKEIKNGDPIILKHMEGYTKYTRIITYMFWTMVLITNFLLILTPLLKYVSSHSYREEIRMGTEPLPQILCSWFPFDNERMPGYLISVIVHIIMGSQGSGVLAVYDMNAVAIMSYLKGQMIILREKCNSLFDDVTSTQDVLDRIKECHRHHNVLLKHSSVFNSLLSPTMFVYVLMCSITICGSVVQFSSKEATASQKLWVFQYTSGLISQLFLYCWHSNEVTLHSKLVDRGIYSSDWWKSNVRVRKQLLLLAGKLNHPLILDAGPYTTLSIPTFIEIMKGSYSFFTLFSQMQEN

>SfruOR25

MGLIKNLCLKLSYTKAIDRSSGRLETMFFENYYRIAYMTGMSTADDNIGYLVYSNAVKLMIVLLVLGVVWYGFMETTSFDEFAGNLNVSLLQFITFYRYRNMLAHEKFYNELASSMESPYFDISTEQRKKLVEFWSRTNVKYLKLLMGLGNCTLLAWFIFPLVDDIEYNLIVGLYLPFFYKSPSMYPLAYMLSVIFFFYISQFVMVTDLKMQTHLIHVLCQFSVLADCFENMIPDCTAGLEGVPRNHLMYNNHFAAKYTERLGNLVKQHKILLGHAMNLRDTLSGPLLGQLAASGVLICFIGYQATATIGQSVVACMTSFLFLAYNLFDFYMICRWCQEITNQSANVGEAIYCSGWECGVSKLPGVRSTIMFVIARANKPLVLTAGGMYDLSLTSYTSLVKTSYSALTVLLRFRHD

>SfruOR30

MVSSEDLFLNRAKFVMKYLGVWVLPENASCFLKAYRGFMMTLQYLFLIFQMIYIVQVWGDLDAVSQASYLLFTQACLCLKVTVFQINMPILKELLRLMDAEVFKPENDVHEKLLELQAARIKRLLLAFMVSSQITCGMWALKPLFDDADRKFPFDMWMPVSPEDAMQYYIGYAFQLGTICISAYMYFGVDSVTFSSVIFGCAQIDIIKEKLMSITSIKERRGTKEVDEALADNYNKLVDCIKHHQAIVTFTELVENAYHSYLLFQLIGSVGIICMSALRILVVDWRSMQFFSILTYLSVMISQLFVCCWCGHELTIRSENLREWLYQCPWYEQDTKFRRSLFIAMERMKKPIIFKAGHYISLSRPTFIAILRCSYSYFAVLNRVNTE

>SfruOR32

MTNHKELNFESTFKITTMALHISGAHPGVPKDLKWVLKFIILHGIFTFTFSIVIYSIINHDLKEKNFIQICKNGVMFVVFCVISFQYFVLVIHQKNLVELIKNVNADYEELQNLSEKEKRLMYKYVDQGIKVCRQWFILTFAGCMIFIVKSIGLMLYYHLINDFQYVPLYDIKYPALIEDRKNDNLFVFLGTYLLLLSFACYSSLNYTSYVPLGPIFMLHASGQLELVRNRIEDLFLECDAEAIRVKLKGIIMKLQYIYSAVDDMKKVFKFGYEITLKGTAVILPITFYAVLETAKNGEISLEFISFIVGGIMISGAPCYYSDLLMEKGEALRMSLYTCGWEQHYDRRTRTTLQLMLQHALRPIAIQTIFRTLCLDALTDLYQQSYAIFNLMNATWN

>SfruOR35

MTSLYRRYFFKKKSKQRSNERIYDKSDYDTSYAPTKKVLGWVAIRMTHNISEKTTMLWDMFYWFEMANLFLVGPSELVSMLTTAYEAKTFRDSIKVFRTMPCFGCVVLSMFKSIKMVVHRPVYENLANELREMWPEGEVSEEEHHIISSALKQLNFIVKGYYWCNNALLISFLSPPYFITIARYFGYDSPMGLHFLYWLPFDPYQPVYYEITLVLQTWHALVVIWFNVAWDMLFCLFLCHITTQFDLLARRVRRLFYVQVDKQLVSSYPMASVSKEFLETEGDRVNSYGAQYWEARYQKEITEIVLRHHSLIRLTNDVENMFSLALLINFMNSSIIICFCGFCCVLIEKWNEVAYKSFLVTALSQTWLLCWYGQKLIDSSQRLSDALYGCGWYNSSKRARSAVLIMLHRAQKGIYVTTHGFSVISLASYSTIIKTAWSYFTLLLNFFKEKSVN

>SfruOR45

MRLQIIKDFLLKQYFDFDRPDINLYNFHPQLRIFLAVKGVFFTNRGSRLRLIWPSICIQLSIIAMTFEEMFIYRGVTIKDYSFATECFCYWVLLGCIPVVYVSILVHTNKIYDIVVKMNEEFIYVCSLGPVYRKPFLEGQLLIWQLCYAWFGFVSFVGGLYVVFPLAGLIYQSLFATLDENTTRPLQFPMWLPHDDPYRTPNYELFLLIQSTLCFCFVQTFCVYVYTLFHILLHYYIIMDMIIIDFSVIFADLEESVALLPRYDTRRMETQRILNARIEKIVKWHLSVFRAVKTVSSIYGPPLVYQVSFSSIAICLIAYQIAEKLDQGSLDILFSLLSICACLQLWIPCHLGTMIRNKAFEVGDAGWKCGWHETPLGLMIRTDIIIIILRAQQPVTIKYTGLPEIQLETFSSCMSSSYSYFNMLRQYSK

>SfruOR64

METLRRFGPQYCDVETMLWNVSVMLRGLTLNIDRRNKKPIPIIVYIISISICLGYFYVYLVSMSWFVFHRCQQTGDLLAAIIVFSLGVSSEIGTVKLIFMFLHIGKVRRIVSECLACDALVVAGSRFSANLLSTLTLVKKRALVFWVVIIGNGVVYVVKPIMMPGRHFTEDKFILYGLEPMVENPNYQIATILSMAGVIFTCYLPANITAFLIVVTGYIEAQMLSLTEELLHLWEDAESHYYITHQTNAVLEESIENSIIRNKVINDYIESHLKDIIKTHGRNINLLHQVESVFSGAIALEFVILGVGLIAELLGGLENTYLEIPFALAQVGMDCFTGQRVMDASVKFERAVYDCKWENYSISNMKIVLMMLQSSQKTMKLSAGGIIMLSFSCLMQVFRSIYSAYTTLRSTMK

>SfruOR17

MSVCASSVAPHVRCLRRVGFCRWGAAAPANRLLATAARFYHVFALAATSTYVLQQLIYAYQERNDMDKLSQVMFVLLCHVTCVAKQVAFHVDAGRIDQLIARLDEPLLNQCGGPRGALLHGTARRAARLLRVYSGCAVATCVLWIVFPVLYRIRGIAFEFPFWAGISYDHNLVFGAVLLYSFYTTNLVAIGNTTMDAFMATILDQCKTQLRILRMNFETLPERARALQRAGGGAYDASLQRLFVDCLLHYNIITE

>SfruOR57

MNFHQIDCFNINMRFFKFLAIWPGNNPCRYYNYYSKAFIMFFVIIFYLLFSINFYFLPRQLDIFIEEMIFYFTDLAVTSKVLTFVLMHDQIIEILDVLECEVFQPDDDEGSIILEKAKKFNKSYWKIVTSVSYLSSLVHILSPLISHLILNVELLLPVCRYAFISDEYRLMLIYPIYLYQSASIHFHMLYNVNVDSFFLGLMVLAIAQLDILDTKLRRVTDDYEVEEAGIDLSRPLTNRRNIEDVMKINQCIIHFIKVCRFCELVKDVFSVTLFVQFGAASCIICVCLLRFTMPAPMGYYIFLGTYMSVMILQIMAPCWLGTRIMVKSQLLAFSVYNCDWTSRSRQFKSNMRFFVDRANKPLSITGGKMFKLSLDTFTSIINSAYSFFTLLQHFQKE-

>SfruOR62

MGYNQIDCFKIHLTILRVLGVWPEENPSICYIYFSRIFVFIFTVLYVIIYTMNFYFLPQQLEIFAEELIFYFTNVGALSKALAFIFLRDKVKKMLDMLESEMFQTDNPEEVKLIEKAKEKSLFYWKITFGLSVSANTVNVFLPFVLHLIFSIKLEFPVCRYSFIPEQYEAIFLYPAYLYQSIGITSHMLYNVNIDTFLLGVMFLAMAQLDILDRKLRKVTDVCVNVDAPRGSIDKMIDDQNAVLEINKCIKHYDAVCEYCKLIQEAFSEILFVLFSSGSCKICMCLFRFTMPAETQYFVFLTLYIVVMTLQVMVPCWFGSRLIEKSSQITFAVYDCDWTPRCRRFKSNLRLLVERANRPIIIKGGKMFLLS

>SfruOR34

MNFFKNPEVPTELYKETDTTRMIDKYNKFWFLCCCPDFWVKKVDYSDTFTRIYRPSMLVNHLVMLIFCSSCFLSLWTQHDLTQSQNSDRLAYAASTPVIIILYHFVILYYRDDVKQVLYKLAVVLKVDHNDKQAEEDMIKQSKLHNGIFFSSCVCNMVFVGLNNFYRAVTTDATFITCISAWPDIEDRSTLAGLTRVVVYFVWFSHVVRNMGVFLIIHTVLLLLSQQYKNLQSYFEDLNKIFLENELTQEEQELKFEVRFKKGIEQHALTLWCVDETQRIFQITFSSHVLLWCGLLISILPDVLNNDTHSLTMLVSNAPRVCAALVGLGYYMWPAGDITVEASNVPHAMYGSGWQCCHDRSPRIRKLVVLAMMQAQRSIELKAFGHFTFSYETYVAIVKMSYSLFSVLY

>SfruOR85c

MKEQAEFTTFHETYKLITFAMSVGMIYPNPKTEMWRLASIPLLVATITPLATMIFIDMYKSWMATDIVNIIRHSTVVGPFLGGFFKMILMYHKRIQAKQILDEINRDYKNINNYSEVYKDITRASVKNCQVYSERGWAITVVTCVMTFPVMAISLNTYNYAFVSEPVKYMIHDLQKPFADDPEDRFASPYFEIIFLYMFYCSILYVVNFTGYDGFFGLAINHACLKMDLYCKALEEAFKADAHEVCGRVIGVIKEQCRMFQFVELIQDTFNIWLGIIFLATMIQICTCLYHITEGYGFDLRYMIFVTGAVIHIYLPCRYAAKLKAMSLDTANRFYSSGWEQIDDQRVRKMILFMVTRAQVPNEIVAFNMLSFDMELFVSILQTSYSMFTLLRS

>SfruOR12b

MEEEPLLINKTIKKIEVWLRATGTNVKSTPKTRMDTMKSRAIYIINFFWLNVDLGGAVVWFISGIANKKSFTELTYVSPCITLSFLANFKCFFLFLNEDCVDKLLEKLRELEMNERSRPRHEDKNAIMVREHKFLTNLISFLNVFFCGLIVAFALGPVALTIFIYVTTNEIDLQLPFLIIYPFDAFQLKYWPWVYLHQIWSEVVVIVGLGAADYLFYTFCSNISVQFQLLKYNIENLIPDDETCGSLVNAEEIRAEFVDLIKWHQDLISSVHLLETIYTRSTLLNFVSSSALICLTGFNVLAGSDFVYVITFVSFLFLSSLQIFFLCFFGDLLLTSSMEVSDAVYNCRWYLAGTSLGKDLLLVQTRSQTPCKLTAWDFSEVNLKSFMK

>SfruOR38

MLSFKEIIHEIRKFGLEYCDLPTMLENVSILLRVLTVNIDSKYKKGITVLSYIVTAVTAACFYYVFLFSMTWFVFWRARVTGELVGAMVVLSLGISSEIGPFKLFYMCYYMDKTHKIADGFLECDANTIKGTRFHTNLMKCLRNVKKRAMLYWVVVAGNGVLYVMKPVAMRGRNLPENYFLIYGLEPMFETPNYQIAYFMMIASVFFVCYVPASVTAFLIVLTGYAEAQMLALSEEMLQLWSDATKYAKTQIEDTTEELDVHNPKVKVIINQFVERRLREIIGRHANVINLLNQVEIVFRQAIAIGFVLLIGGLLSELLGKLENTFLQLPFALMQVSMDCFAGQRVMDASAMFEASVYDCKWENFDKSNMKLVLVILQNAQKTMTLSAGGVRTLSFSALMSVFRGIYSAYTALRSTMK

>SfruOR67c

MPSDQSKMFDNSFRIAKIFGIWPGFKPSRYYKFYSFIYLFVTFVCYNLLLTLNLLYTPRKIELLLREVIFYFTEITVATKILTILFMRDKIIEALNLIDCDEFVGDYENKEGILFKTNMGFKLGWKSYLVLSNIAYSSQVVAPIFLDILRGTKSELPICKYYFLSDEDRETHFLFWFMYQSFGMYGHMMYNVNIDSIIAGLLLIAIAQLKLLSNNLTNFKLSDEESKLPKDSQDKIQIRKLNKLLRHYEVIVNYCETVQDTLSVTLFFQFSVASIIICVVMCGLLLPSSTETRVFLVMYLLTMTLQIFVPGYLGTQLTYGSERLVTAAYSSEWLPRSESFKSSLKLFRERAGRPMVISGLKMFPLSLITFTSIMKTAYSFFTLIRNVQES

>SfruOR1

MDTNTDKTTLNSEKPDHYIHYIEKPLKLVACWDLFPNSANEKRKVFNDIYLGIVLFVLTHIPMVLTVHLYTEWQDIMSSLGTIADALPLLVSLVIVAYYAIHRRDLYELLNYLDKNFKYHSARGLTNMTMQQSCMTARRFGRIYTACTMFSVTMYATLPVIVHLWTKEPIQSWIYMDVTQSPFFEFVFLLSCLAQMYVGLAMGQFGVFFASNSILICGQLDLVCCSLRNARYTALLQEGVKHAALVATHSDIQRDEDHNYIYNFSEIKESLYHYDEKVTHNYIDVKTNFDIYSPEYDDATMEALRDCASLCQVVNKYKEMFESFVSPLLALRVVQVTLYLCTLLYAATLKFDMITVEYLAAVALDIFVYCYYGNQIILQADRVSTAAYQSMWHTMGIRPRKVLLNILLANRRPVVVRAGKFLPMDLHTFVVIIKTSFSYYTLLVNVNEK

>SfruOR49a

MKSAAIDLSRHISLQIGALKLFEIWFYIPYNMKDVRFWVRIWTRIVLGIIVFVIPTGSQLIYAVRLIMSGNAEIQEVAGIINLILTELLVSLRLLDLSLRRHRVSQLAEQLKCEEFQFHSPAQKRILEKAVESSRRLFWILLCSCSSDVLVHVVVVPALHNFQTLPLKMDLVYLDVNKESYFNYLCAYQILYKPMMLATFAAVQTLPWATMTCAISQLDVLIYNLENIQDLVKATVAEKQCTENEAFRDIFKGCVLHHCSIIKFVNTIQNAFGGQLSATLCLSTGILGTTAVQMLSIESPLKNLTEVIWVLAYLSIFICNLFIDCYFGNTITVKSMHVSTVIFSCPWIELPTTLKKNLVIFIAKTQRPLVITAAKLVPVSLDTFTKVMNWTYKAFAVMNQMKN

>SfruOR67a

MRVLSYVWKSISQSKALELAGPLETAFFASVYRLSFFVGLSMSDDYLLYTLYSSFIRIICSLVVWFEIWHVLGNTDVSLDQIISSVNVIFIHLVTIWKLVTMVKNKSIFKRLAKALESSSFDMSSPRIQCVINNWILISNQYFKMLLRLGLLTLTVWESYPMIDEIKFNLMVDVKLPFEYQSLLTYIVTYIAVAIMFAYASLMVIISELIMQAHLIRLICQFDVLADCFENIFEECAEEFPDLTKHELVKDKTFVDKYVKRLGDLVAQHREILDQTNDLRTILSAPLLGQLTCSGLLICFVGYQATATIAENLGKFVMSLLYLGYNMFTWYLMCRWCEEITIKSQRIGEAAYFSGWESGISLAPGARATITLVIARANKPLVFVAGGMYTLSLSSYTTLVKASYSALNILLTMSHE

>SfruOR49b

MSDKCSIQNVLKFLEDPEYPSIGPHLKLLAFTGLWHPNRQTLIGRFKQILFYVTITFFFSQYIKCFIKFNADSLKLILQYAPFHMGIIKSCFFQKDYKNWELVIDYMSSVERNQLAKNDKKHNDIIHEYIKRNRKVSYFFWALAFFSNFSIFTEPYQKNQINVNGTSIYLKIFDGYTPFDNEPPGYYFSMLIQTVLGHIVSAYVVGWDTLVVSIMIFFTGQLKITCMYCRLAIDALDSTKSHENIAKCHRFHTTLVEYTHLFNSLISPVMFMYLVVISINLGVCIIQIVEIHDDIATLVSSILFVVACLIQLLLFYWFANEVTVESTFVSYSTFESDWPQANKKLQKEIALLGLTTKKILVFRAGPFNQMSLTTFIAILRASYSFYTLLNSTN

>SfruOR27

MIILNENMKNKLAFLSPVVPYGVFESWEDLNPRLYHAVHIYWLKFYGMWFNDSSPQSIKFWVQMFYTLIVLWLVCFFPGIGEVVYLLRRRENIGDVAESLYLFLSEMYTYFKVAVFWLNRNKVIDLLKYLHCEEFKPKEPEHRDIIIKSIKSARFVMTYYSTMCVGAVSVGIIMPLTENFDILPTNVEYPFFNVYQTPAYEAVYVHHIYYKPATCIIDGVMDTILAAFVASAIGQIEILAFNLRNFDILAERQRKRDVAENKFIEEYKPPYYVRSVLKECIVHHNCIIRYVSMIESAFSLASALQFMLSVMVLCLIGIQFLSIENPSSHPMQIAWMGIYLTCMLIEVFILCWFGDELIWKSMDLAKAAFEGPWMNSDRKSNMFIIIFLERCKRPMRLTAGKIFTLSLDTYTVLINWSYKAFAVMRNMKK

>SfruOR60

MGIFVKNATLSVSISLTALQTVGFWAPEGLTETQRCLYRWFGVFSFMFMLGSYIFIQVVDLFMIWGDLPLMTGAAFLLFTNLAQATKIVNIMVRKQRIQNIVNDSDKVLTEVQTFEEKEIVKSCNREMIVLQVVYFSLTLITGLGWATSAEKHQLPLRAWYPYDTSKSPAYELTYVHQIGALLIAAFLNVGKDTLVAALTAQCRCRLRLLGLSLRNLDKGLDTEDKFMLTPDQEKTVSSRLRLCVVQHQETLQAAKELQECFSEPTFAQLTVSLIIICVTAFQLSMTQPDNMVRLLSMGTYLLNMTFQVFIYCYQGNQLTEESFEIAGAAYECLWYKFSLRLRRALLIVMVRTRRALRLTAGGFTTLSLTSFMAIIKASYSLFTLLQQVNEE

>SfruOR3

MALTVLERFYLIDDGFFSFNIKYLFFVGLWPEKTLTRNQKILYKIYEIFIHTLTIIFLIMAGIGTYQHNDDLLIVLSNMDKSLVVYNFFLKTIIFLIKRDQLKDLIDEIEASGDEVTKERKKLMANYVMVITGITAAVVSAFSILALLEGAMSIEAWMPFDPMESSMNLVLSLQIIAVCVFPALCRAFAMQGLVCSMIRYLCDQLIHLQKELRSLDYVKDTEMITRMKFKMIIRKHIRLMSYSMKMESIFKEYFLVQNLAVTVELCLNAVMMTTVGVQQITLLFSFLVYLVLALINAYIYCYLGNELIIQSEGIALAAYESTWTSWPVDLQKDLLIVINASQRPLKLSAGGMALLSIQTFSQALYNGYSIFAVLNDAVN

>SfruOR6

MGLKHFLFENECVKGITAPTDYLYVKLLRMTLRVIATWPQREMGEKEPVYLNNFLKYFYLVVTIGCQFGSYLYLRAYNDELTMMEAGHNYLMIMMTFIDISRIISLTFSTEYRKICKEFFTKMHLFYFKNVSEYAMETHKRVHLLSHLFTLWLLFQMVFGVIFFNLIPMYYNYTAGRYKPGGTQNSTFEHSLYYKYPFDTLTEVKGYIVANIINWILSCLCVTWFCMCDLILSLMVFNIWGHLKMLTHTLHNFPRPSLETSITIDGGLTVTSAKYSVEESVQILDKLKECVNYHRRIVEFNSKVSDVFGPMLICYYLYHQTSGCLLLLECSQMTAPVLMRYLPLTIVCTQQLIQLSVIFELIGTESEKLPDAVYSVPWECMDTSNRKFVIFFLMNVKEPIAVKPLGIANVGVTTMAAILRTSLSYFTFLRSI

>SfruOR18

MEIKTDIPTDKKYKTFDKTFKLCAFSLAFAFLYPNRKTTLKRCVTITLIITFCGGQLFWFITYTFKCLYTLDLYNFARNMTLAVVLILFFIKTYYAIYATPKFATLLEKITEDLLEANNLGEQQQKLYDEHIKIAKVGEISWLVIPVLMSAIFPLYAGTLMSIESIETDDYQRRMVHDMELLYVEDIQSETPFFQCMFAYNCVQCVVLVPNYCAFDGSFCIATTHIQLKLKLMALKVHDAFKYSKNKYELRMKMNEAIRDHQDTLDFYVQTQKLFGPWLFFVFLLTSFMITFNLYQMYLLQRIDPKYTSFGLVGVMHIYLPCQYASSLTKVSEDIPNDLYVVDWEVWADPNITKQLIFMITKAQKEMIMTGMGLVVYNMELFKSIMQTSYSFFTLITA

>SfruOR46

MEVEKNTTLFLGRPKNILTAHGVWPHPNNYIILRKLYMLFVMWTQYSFLLFEIIYIVNVWGDIDEVSEASYLLFTQASLCYKSTTFMINKKSLLELLDIMDSDIFKPKSAEHEKILAAQALKIKRLCLFFLTSATTTCTLWAMIPLFDDASKRSFPFRIWMPVTPLKSPDYELGYLYQMVSIYISAFLFISVDSVAVCMIMFGCAELEIIMDKIQKIQSIFDTDQSNEDRKKLIKMNNELLDECVKQHQTVERLIQLCEDTYHANIFFQLSGTVAIICNIGLRISIVEKNSVQFFSMLNYMVTMLSQLFLYCWCGHELTIRSENLREWLYQCPWYEQDTKFR

>SfruOR23

MDEAFLAFHRVLSFAGISIFAKQNWDSNRWLGYQIFNFIIGVLCFIFTTGFVVTNVSDFLLCIQGACIWTTGVIMTITLGVCLVFRNEFRFFLEEMAFRDHMLEMPLIDHILLVSPRGAKIEELRNLVTGSQEKLFKYVTILLKSYVASVFLCATLYLCSPVYGMLVREDQSLRLLAFDMWFPWSLEDYTVYIISFIFHAYAGYLCCIAYPGLQCIIILLLGQLIRQTRILTFILLHMNELVLEVTAVQDERWQDICTLVLSQCVDHYVKIKRFSNRLNVICRPFYLTLILVAIMLVCMCSVKIAVSNKLSLDTFKYYIHEFCFILVVLMFCLIGQQVENECEALERAVTEKWYIFNHNHKSNIRIFKMALGQRMPIYIFGTITLSLPTFTWFIKTGMSFFTLVMSVLEEET

>SfruOR2

MLKNILQKLENPKRPLLGPNVKALQFWGLLLPKNVFMKYFYLLMHVLVTIFTATEYIDVWFVKSDLNLLLNNLKITMLATISVIKVSTFLSWQKYWISIIEYVTRADLDQRLTEDKKKLKLLKAFTKYCRKITYLYWSLMYTTVIIVMVQPIFKYVSSETYRMNVKNGTETYLQVVSSWVPFNKNTMLGYLAASVYQSYAAIYGGGWITSFDTNAMVIMVFFRAELELLRIDCRDIFGSESVPVERDVALKRLKDCHRRHVELVKYARLFDSCLSPIMLLYMFVCSVMLCVTAYQITIETSPMQRFLTTEYLVFGIAQLFIYCWHSNDVLYASADLMLGPYESTWWSRSVQYRKDLYLLVAQFNKSIVFSAGPFSKLTVATFISILKGAYSYYTLLSQSQMK

**GRs:**

>HarmGR1

MNKEEHGFRVYNTNTVHKNETRKREMFQRIDEKDGIKEYDAKDLYGPEITDKDGALLDAHDSFYITTKSLLVLFQIMGVMPIMRVPKNAQTTKRTTFNWISKATLWAYLVWSLECIIVVKVGRERLANFQSSANKRFDEVIYNIIFLSILIPHFLLPIASWRHGPQVAIFKNMWTHYQLKYLKITGTPIVFPNLYSLTWGLCVFSWGLSFAVILSQHYLQDDFELWHSFAYYHIIAMLDGFCSLWYINCNAFGTASRGLAMNLHKALEAEHPALKVAQYRHLWVDLSHMMQQLGRAYSNMYGIYCMVIFFTTTISLYGALSEILEHGLSYKEMGLFVIVGYCMTLLFIICNEAYHASRKVGLEFQVRLLNVNLGAVDRSTQREVEMFLVAISKNPPIMNLDGFTNINRELFTANVSFMSTYLIVLMQFKLTLLRQSARKTLKTIVRAVFNTTTTILDDDFTDDVDEE

>HarmGR2

MTIPDHLFDEGINNTLLQHDMRHVQQNRIVYEKTQREYEQEQRDMLSSQDGDTCEIHDQFYRDHKLLLVLFRALAVMPITRSRPGTITFSWRSTATMYAVCFYIAATAVVMIVGYERIMILRSIRRFDEYIYAILFVIFLVPHFWIPFVGWGVAHQVAIYKTNWGKFQVRYYRVTGENLKFPNLKTTIVMISVGCLLLAVCFLLSLCILMDGFLLRHTTAYYHIITMINMNCALWYINCKGIKIASQSLSECFRRDVEAECSAKLISRYRYLWLNLSELLQSLGNAYARTYSTYCLFMFANITIAVYGALSEIVDHGFGFSFKEMGLFVDAAYCSTLLFIFVDCSHNSTLTVAAGVQETLLSIDVLSVDRPTQKEIDHFIQAIEMNPAVVSLKGYAHVNRELLTSAISMIAIYLIVLLQFKISLPRDPQIVAT

>HarmGR3

MTVPIPNGFPVQINSKPKNKIIFLDVTPVSTPIKPHSPNVVAPMRNNLVAPHISNDIIYENIKPVFTLLRIMGVLPITRPSACVNQFQIASSSMLYAILVFLSLVSYVLYLSLHKVQILRTAEGKFEEAVIEYLFTVYLFPMIAVPLLWYETRKIANVLNGWVDFEMVYKQLSGRTLPVKLYKKALAMAVIIPILSTTTVIVTHVTMVHFKPMQLVPYVFLEILTYMLGGYWYLLCETLSICANILAEDFQNALRHIGPAGKVAEYRALWLRLSKLSRDTGIANCYTFTFVNLYLFLIITLSIYGLLSQISDGFGIKDIGLALTAFCSISLLFFICDEAHYASHNVRTNFQKKLLMVELSWMNTDAQTEVNMFLRATEMNPSQISLGGFFNVNRTLFKSLLATMVTYLVVLLQFQISIPDESQNRDEEEEVPYNITSATTEAMTTSTTTIMTTVLTTLAKKKKKN

>HarmGR4

MEIKLCKLFVVLTEGIEYMKGNSVKNLNEKKRDDFLPTLNNVFLKARFFGISGYGLTISFFWSLILFSMLVVMESVAIWRVVTLLGEWLVSASNNGLIGRLSGAIFYGNALISLFLSSKFVHSWRSLSNYWLRMETSTALDFPADVRIRKRTIYITAFVVSVAVVEHILSMISATGVGFPPEEFLYRYVTLSHGFILKAQDYTIWKAIPIFVLSKLATALWNFQDLIIILISMGLSSRYNRLNLYVRHVVSVEKQFESKQRFGTELYLQIQVWRRLREAYVRQSTLVRMVDRNLGSLVLLSNINNLYFICLQIYLGIHKSSGSTISRCYFLFSLGWLIFRACSVVLAASDVHLHSQRALKSLHACPSAAYNVEIKRLQYQLAHDFVALTGMGFFSLRRELLLEVAAAILKYELVLIQYDK

>HarmGR5

MQNGWNNVISNISVGSVNTVNYLFRTWERLAPNRNMDLYSLEKFKKYKNDWNYPVHVRYQDQVMAEKEKPRMTFQTAMKVTLTIGQCFGLNPVQGIREKDASKLRFKLLSGRCLFTFFSLIGQFIMAFVLFLSLFKETSSTVDTASNFGFTTTILFFRIATNWPKLCMHIAKVESVDPNTDNKLGKKFNIACISILFLALMEHLFSELHGISIALDCFPDTPVYESFMKLSFQWLFGFIPYSDFAGGMAHFSNLQCTFNWNFADVFVICMSMYLTARLEQVNQRIIAAKDKNSPSSFWRTMREDYNRSVHLVRQVDKIIGGVVFMSFASNLFFVCSQLLHTLAGGIKASQRCKPEIGADRRFFYGYEHSIYFVFSFSFLVIRSLAVSLTASKVHAASLEPAYSLYDVSSANYCVEVERFLDQIHGDTVALSGLQFFHVKRGLVLTIAGTIVTYELVLMQFTGITPTTSPESVSGVIK

>HarmGR6

MGQTSFRRNMSFWIPVKKNKVDVAKPKVKNITSFQDALRVTVIIGQVFSLLPFVGVFTNVASNVKFVKTSWKCVYSLLSLVGQMFMAVLCINKLAKTTVSLNGTSPVIFYVTTCVTMMLFFQVARRWPALVQHISKAEDMDPNFDCSLTRKCNITCAVVLILALCEHILSLLSAFAGASACYSGMDTYEGFVTHFYPWVFSYLPYSIVLGVITQFLHFQSTFIWNFSDLFVICMSYYLTSRLEQVNRKLLAAQGKYLPEIFWRATREDYCRATQIVRKVDEVISGVVFISFANNLFFICLQLFNTLEDGLKGTGECTPKLKKIVVSKSGPLGGHEAAAYFLFSLVYLLSRSVAVSLIASQVNSASSVPAPVLYDVPSPVYCVEVQRFLDQVNGDKVALSGLQFFSVTRGLLLTVAGTIVTYELVMFQFNSSTPTLNITSPTVVTHTITTLAT

>HarmGR7

MSSRGFGQFLRDGNLILPEQPNHDDFLTVMEKVFKWSCLIGVLGSKRHINYAWSGFILLVLLFMESQAIWKVIKALAGWAIDTAGQRSVTARLAGTIFYTIAILSLVLSSRLYRSWGQLSALWARVERIMAVKAPPDKTLKRRMYFFLGFMTVCSLLEHIMSVVSAIGLDCPPALIIKRYVLISHGFMILRHEYSDWYALPLIFMSTLASLLWNFQDVLIVLISMGLTSRYSRLNQCLAKICALERKQMDSDKKNEATKVYAWRKLREAYVKQAMLVRKVDDAIGSIIILSCFCNFYFICLQLFLGITQSKASEPIKTAYYFLSLGWICFRVLCVVLAASDINVHSRLGLKYIYTHDSHSYNIEMGRLQDQLSKDYVALSGKGFFYLSKSILLQMAGAIITYELMLIQFDDQGTDDVQLNLTKNAIGV

>HarmGR8

MSSKEFKQFLRQNKLLLPQQPFHDDFLDVIEKVFHWSCFYGVFGSKRSISLIWSTLILGSLVIIEVLAIWKVIRALAGVARDMSGHRSVTARLAGTIFYSISILSLVLISKLYYNWRTNIAGLWGKVERSVGVKIPVDKTLKCRMSFVAGLMTFCSFFEHALSILASVGFDCPPSLILKRYVLVSHGFIFMGQDYSEWFAMPLVIISTIATLLWNFQDQLIVLISMGLTSRYRRLNECLAKFCELEKQHMDSDKKVEAVKVYTWRKIREAYVKQAMLVRKIDVALGGIIILSCSCNFYFICLQMFLGITQGMSTDFLTGVYYMVSLAWLCIRVLSVVLAASGVNTHSKLALNHLYTYETHCYNVEVERLQDQLTKDYIALSGMGFFYLNKTILLQMAGAIITYELVLIQFDDQGNDGIALNATNI

>HarmGR9

MGVESAKVEEVTAAPVPSESGARPSRPTHCVVGGAHAFILRISSFFGLAPLRFESRSNGFTVSISGAMCVYSYILVTVLVICTIFGLVAEINVGVELSVRMSSRMSQVVSTCDVLVVVATAGAGVYGAPRRMRNMLKFMENIASVDTSIGGQYSLVTERKLCGIILAILIFFSILIADDFTFYALQAKKLDREWDVVTNYLGFYLLWFVVLILELQFAFTALSVRARFSAVNDALALTARQVSIPVEKPKSSSPLNIYAIRVAPVDSQRSANVSLLVDTMTGREHVVIIKRTASGEPRLVVSPCDAVRRLAALHGTLCDVVNSIDDSYGLPLVVILISTLLHLIVTPYFLIMEIIVSTNRIHFLVLQFLWCVTHMLRMIVVVEPGHYTIAEGKRTEGLVCRLMTSAPSTGVLPSRLEIFSRQLMLQSVSYAPMGMCTLHRPLIASVIGAVTTYLVILIQFQRYDN

>HarmGR10

MEYGLDAKISKELESINFRVLQETQLDDEAKKRSTKPWIEEDNKLVGKRRIDVKDQFTAFQKAMKVLLVWGQTIGLNPVTGILQKDPSKMRFTVYTWKFLFSLTVAVAQTIGTTLCIYKLFREPTSISALGFVTFFTSTCFTTFLFILIASKWPTLMQDIVRSKLDEYVDKKIITKCRITCCIFIGMALMEHFLSILSRVARVIECSQNETDHGEVFVKVTSPWLYDLNVPYVVAVAVMVQYVNLITTANWNYSYIFIVCVSMYLSSILNQINKRIALEAQKTHVPAKIWINLREDYTRATHLVKRFDDVISGIVLVTYANDLFFICLQLYNVLSNMSKAAQLVNKLCPDQDGTFRAYSYPAYLIYSVLYLLVRFLTVSIVASGVNTASLLPAPILYGIPTTAYTKEVERFQNQVNGDVVALSGLHFFYITRDLVLTLQN

>HarmGR11

MPSKLFLKTFNMATRHRLDKREICGLHSTVRGTLFCSRVMGLLPVSGLTCPTSRRLRFTFRSPYTVLYVASLFGQLLMFVMTLCWLMMNGISLANITNAVFYTSSLISSLILLHIGRCWPALVGSVETLERELPPFHRNVASISNVTTIFILTAAIVEHLLSVFYGLKVACACDSNNVAENYFRFNMPWIFDYTPFTIWKGALSELFNIQSTFVWSLNDLLIMVISIYLTEHLLIHNELLKKAAEQEHFSCLEFRTQYLKIVRLVKLINGQFGIYILTSFGSNLYWICTQLFYSLSRTQTGHFITCTFKDSPTKVPPANEYENPLCPWMLGEKGALNGVEHSIYFTYSFSFLLLRTLLVLLLAARIHSNSVAPLYVLYGIPSSRFHIEVERFIAQINNLKVAMSGLDFFYVTRTMILTLLGTIVTYELVLLQFNR

>HarmGR12

MKVHQLTMRRSNKRCGLHVCLRHAMRLARWTGFFPLQGLGQAYADGTRYKILSLYFIYNFTTLLGQLVMSCFAILLFFQTEVTLNSISNVIFYVTSLISAVLFLKLAKQWPRLMARATETEQGLTELKLPNKVIVKCCVIAYVAMALALVEHILCTSFNLTFVMHCLKEAGITTNVMENYVVHRMPYVFNYVPYSLFWAFLFEYLCLQSTFVWSFNDVLITCFSIYITAYFRSLNQVVTANSKKDKDNMIPWSTLRVHYSKLVRLVKEIDNHISSFILLAFFTDLFYICLQLFNSLHRNYASFKFCNELQTKQALTSPSYLLYYLYSFIFLVLRATMLSLFASNVHCAALEPVHAVYDVPSTLYDNEVRRFQLQLHHTKVGLTGKFFYVTRNMVLKVIGTIITYEIVLLQYTITPNPYYNGTKVILNISSHSYS

>HarmGR13

MSAFRDTECVVTGTLSMMLRMSQIAGVAPLSFRRTHGGWYIRTSRAANCYGKALSFCLWFLSSFTIAIDILIQPERSFRTRTNSTRIVWLADVATVAIVVCAAAFTGISRMRCLTVYALKLEEINLRLSLFHEEPSNEANRRLIAVSSMIFVVSTILVDYSIFIYQVITEHGKIVTSCMYIFYNISTIVQQVILVTFSETVTSVLTSLQMLNNCLKNLLQEILDSSELTNCALNYDSYINMNPNRSAIPNKSINSVVDTMAVYKGYRKNSIKAVPSTIRRLALLYCSICDVIRLVNDSHGLILVALMLCLLLHLVITPYHVITNIFNKERSDRSSPLLQLNWAVLHFVNLLLIVEPCHRTHEEMEQTRHLISQMIRYTPSEHGVLLTELQMFYQHLILNEVSYAPLKMFSLNRSLIVTVI

>HarmGR14

MRKLNLTPLKYLLIIENLTCVFRNYLCLRKCTRCLVTIWVVFETGHMFFNVYYNIVHGQNAELKAQRIYFFSSTAFSIYVMTSSLYFSKRFYKLLSNFDEFYNIFEDDVYNKKMKRAQKVMIWMVVCFIGIKFIVFYIMQVKTEDLENGLVSNTVSEYSVTVNDFRYIFQYFILDCILLIVAEQLRAISRSIDSELSAMMENQRNVGHVEGLPRVVLNYEKINKWAQAYESINESTHLCNSMFSVQLTIMLLIVTAYYIILLYSIAIITVEGVHTVKTLVMNLFSMSVFLLALLVISRAGQKIQNSSQQLRQRLCELCVHTLGNEEYYKLAKDLLRCVRTRPVRIHVFGTLDVNMSMLPSIVVLFTSYTVIALQFNNVL

>HarmGR15

MELVKLEMFIKAIMIFRIVCGLYCKITSNKIIAAIIKIYCALVIVIVFVISLSYLRALKNSASSAFAFGMPASKYFVNSVVHFCFDGDNFYEFFHSLKNLQITQDSELKYDIPITIFLFISITGARLKNYSRYYWLGMSDGHFRFDRFMSFCFGVLISYIGSITCVMRFMMFELLWRRMAMLRKRLEQDLLNARRFENEEGVLRKQLRACLSIYKGILDSTRKNDAPMKLLVTHLYIIFLNNVFQY

>HarmGR16

MAFVKSETFIKVILIYRILSGYYWKISSNKLVSVLLRIYCLFIATAFLCSMYQFYLFVSIPGKIHMCCICYLFSINVATNLIFNGDNFVYFLQEIRNVNIPRHIGCGDKIPITTVLIILTIFLRIGSRIQFDDHTHIDIMQGVFSIQNFSLTFDFLYYSSYLTRIMMFELLWQRITMLRKCLEQDLSIARRFEDGEQLLRNNLRACLKIFRSLVNTTRVCDAPMKLLVRDSVLY

>HarmGR17

MALVKPVALIKLVMFIRLISGFYCKISSNSKINFLVKTYCILIATLIIFLTITVAFVRVNIESKCHIGFMSTLYIISVVTSICLNGDNFEDFLTKIRGITTLPNVESADKITFSIFLFFLSLCSRIVVNVKFTIDNVQSISDPLFYVSLVSLTLATLQYSASFTRIMMFELLCRRMVILRKRVESDLSIPMTYQAKEMVREKIRKCLHTYKSLLDTIRGTDMPMKFLVSFLLKNLIEINRMQRVYKPGVHAPQIPSV

>HarmGR18

MFIKAVMIIQIFCGLYNKISSNRIICAITKIYCALVIAAIVIIAVIHIFFFNIFIVAKSSVVISVTTYVLYAMVHFYFNGDHFNEFFYSLKHLQTTHGSESKYDISITIFLLISISSARLYGYLKFTFMHISILPIDEFTGLFLDLLVCNISHTTCVIRYLMFELLWRRMAILRKRLEQDLLNARRFENEEGVLRRQLRSCLSIYNEILDTTRKNGGPMKLLVIHLYIIFLKIVPLKLTNITVVLLVPQTLNHMFGFLLKNAIGSELTVVDMYILFFETLSPALLAEMVQSEIECMKLSIVKQLLVCKDERTLNAIQDATTYLEQNPFKYTIWRVFAVDMSLILNMIALLTTYTVAMVQFAHFYD

>HarmGR19

MASVKLETFIKSVIFLRLFCGLYYKLSSNKITVALTKIYCTFVAKIIITIFVLFIKNLKVPFQIYLSFGLMCSGYLTNVVFSAYFDGDNFMKYFSALKEIRNPQDFPSFSDIKISIILLFFFASSRIFNYAAYSVSAMFTFQNPFPYNIYTTTAFIGSIAVLHSLVNSTRMMTFELLWRRMAILRKRLEQDLLKARRFENEGDILKNNLKQFLNTYKSILDTIRISEKPMKLGVTVVTNNKSSYLNTNHIAVNILR

>HarmGR20

MVFLKQEILIQILLVYRLICGFYFRISANKIVAPLFKVYCIFLASIVIAKTVWLIVNNKLDTLGTFHMCSACSLYVSHVAINLFFNGDHFIDFLEKIKNINEEDTCAPRDKILISAFILFITTLVRFYSYVQLEYDFFKRFFGSLDSVFTFSLLVDVFQYSSTITRTMMFELLWRRMGNLRKRLEQDLSIARRFDEGDEVMKEKLRLSLHAYKNLMKITFETCTPMKLEVIYMRFVIKNLKLSFILKRLYRRF

>HarmGR21

MEATSVFGFHRSVLLSANSITMAFVKPETLFQVIMVFRAILGIYHKISPNKLINAFLRVYCVIVAIVVHLGTIYIMQFFNLTEVHLIILNLFYSVDAVMGLCLDIDYLKGFLQKVRNMDTEHFEMKLKAPVTVFFIFYILIARFYCHTKFRYDNGESFFSPLFSEASLGLIFDLVRYSSIWIRILMFELLWHQMAMLRKHFKTELSVARRFEEGDHMREKLRSCMTSYKNLLNASHKFDPVVKTQVRV

>HarmGR22

MADIKPNQMIKAVMILKIICGFFCKISSNKIVITLARVYCLFFGLVCMSMLIILANHAKSFSALLQVFFGVYVYLIYFLLNFYFEGENFMGFLHKIRKVVTSQHMKPCEGVQISIRLFTISLAANILLRLKITLDQSHYTFSSTYSFLAFVSTINLLEFSPILTRLMTFELLWRRMAMVRRNLERELSTHQGYENSECILKEKLRRCLNVYKCLLEVTRVNETPMKIAVYNQKLKYARRSQRLSLIS

>HarmGR23

MVARLMVGFYWKTSSNKLVNALVKTYCVTIATVFNIMSITYITNEDEHIPTANTIYMAFVICLYDIIVVVNLFYTGEYLEDFFDKMENPAIAQDMEAGDKLIVTTAVIIFSLTTKFITGSIYIYEKFTLTNTSKNFILLIMQIINPISNFTRIMVFELLWRRMAMLRKSLQQDLTSARMFEGEEIFKTKLRTSLIKYQLILDTLKKIKHPIQFLVIVSFMNYVKSSYHMPDKKKLFSVVILFSDFVKYSSLYSTCTASYVWFYCIWRS

>HarmGR24

MVARLMVGFYWKTSSNKLVNASVKTYCVIIAIVLNILSINFITSEDEHIPTINLINMAFVICLYDIIVIVNLCYAGEYLEDFFDKLENPAVPQDMEAGDKLIVTTAVIIFCLTTRFISCSKYIYETYIALNNTAIDIHCLLLIMQIINPISNFTRIMVFELLWRRMAMLRKSLQQDLTTARIFREEEILKMKLRTFLMKYQLILDTVNKIKHPIQFLVIVSLMNYVKFSIHMRDKKKLLSVVYLIVFRFFQVQ

>HarmGR25

MVARLMAGFYWKTSSNKLVTALVKTYCVTIATAFNIMSITFITSEDEHIPLLNQFYLAFVLCLYDIIVVVNLRYAGEYIEDFLDKMENPAVPQDTEAGDKFVVSIAVIMFCLTTRFMFYSKLLYKSLIDPSDLSISRNFILLIMQMIHPVSNFTQIMMFELIFRRMAMLRQSLQQDVTNTRIFGGEEILKMKLRTYLMKYQLILDTINKTKQPVKYLVIV

>HarmGR26

MDVQATPASIKTIMFIRLLCGFYCDISANKIVTVLVRVYCVAIITLVMAFGIYLWNGIIGISSKIHFLFITTPYITSMVTNICFHGEYFSEFLNKMENFNLTHGFLSSIKIPISSLFFVFVFLQRFLFQMKFTFDAIGLPFRGVLTHASFILILCLMNYTAEFSIHIMFELLWHRMGMLRKRLEQDISTARILRDGEESIRENIRTCMRRYQHLLETARVTDGPVKFLVDTYIFITAR

>HarmGR27

MYSVNAIIIYRFIMGFYTEFSSNKIINFISKIVCVLLNIFITTKLFSLIDYNIVSSFIAFAGYSIIYLTNALASLFNSKWIQTYVNDLKSIGNAMGTGADIDIPIIRIILLCLHGLSSTTQIVTCEHEFCSGFYQKINGFVYSLATAMATILHMVVFELLFHKVRANRKYLENQLTIFKRHQNIMDLKNNLRLCMSNHVKLIDSLDKTDKSLKTTFFVADLAVIPIIMSRVFEIVKSPDRSALVNILLIGKGVVLRSMPGILADLTSREIDKMKHIIGQNLLVYEDDDVKYLLKDTHLFFEHQPFQYTVWRLFSINTAMNVVAFKMVVSYTLAMIQFAHFFG

>HarmGR28

MPSFRESGLESFFTLKGILIMRSIFGYYYKFSNSVPISVLLKLYCIACCIIIWCHIFVWSDVNSRAAFYDVTLLIEITINMLISLFVEDEFSTLNDLIASLPTKQNLRLTYAVIICTIIEQVTAFTYGRSLFTAEDAALYMFQYISCTYGRLSLIYQAHNNEKAIKTLCDSLKDKIEDMNMDAAEKRGHVEKFIDTFKQIINFSFDAKCQVMKFKVAVLGYVCDFFRVQFMVYYIYEFHLKIQVTLMLPWYCGVAYSIFIICLPSMLGELANYHLDEIKVVLVDEVIRNEGTYYFVIILGLRFD

>HarmGR29

MPSFRESRLESFFTLKGILIMRSIFGYYYKFSNSVPISLLLKLYCIACCIIIWCHIFVWSEVNSRAVFYDVTLLIEITINMLISLFVEDEFSTLNDLIASLPTKQNLRLTYAVIICTIIEQVTAFTYGRSLFTAEDAALYMFQYISCTYSRLSLIYQAHNNEKAIKTLCDSLKDKIEDMNMDAAEKRGHVEKFIDTFKQIINFSFDAKCQVMKFKVIQFISTSHIFVKFKIMQ

>HarmGR30

MAYLQKNLHIVKFLEPLMSVKTIMLIRISGGFYHKISSNKLISCITALYCLAITAFLSVNLLTLNYPSYFILVKNYVGFLIYYLLVGICLLTDSGYFQNFLNEIKKIDHMLGASSNIKVPISSFLLLGIITGVTGMDVLYVIYEVENNRQEKGFFITLFCMSLLVQTCAIANYTNVMAFELLWYRMRIFKQFLENTLKREFRSQDEEIKINAVRNCMVLYQRILDTVNKNNLPMKLLTFVLVTKFIPKTVATLYDIVTNAIPEVSYMLIHEFFVDFMVLCAPAIFADLICGEISSIKSMFKKQLLTCQGTFTNCYLVLAKKRSQ

>HarmGR31

MDKSLYKLLLFRFFFGHYFKLSSSKRICFIAKIFCLFTVIYISVIFVKFFLLFGSVISSAINLWILLIEAILSIFLSLHTEEAYVLKFSAKIKNYVSSSSTCRVTNVLAFLIIPINLFYLLAAYQYEFSITNNLLYNITFTVCYCSYLTSLYITEMFASAINNLTSDTVIKLKDVDITDEEKRFCIENFLDNYLKLMNIYNVTATVSRIKVSV

>HarmGR32

MPWLKGTEIESFFTVKGILLFRLIFGFYFKLSNSIIIDLLIKSYCFLCMILMWLHIIFVPNPENPAARVYDTLLAIEITLHIFLSLSKNEFGLDLIHSFDSAILQTKPRCLISYSMIFPVVFLEIYVAAFIINWNETKFINITMFYYQFVACYASRLAAIFQSENYAHATKTLYVQMKENFESNMNSAGKCEHVQQFNDKITITVDFFDKHRNVTRFKVKHLCISIFFFYIKK

>HarmGR33

MTDDTLVCDIKNVLFIRFALGFLQNFNGTSKTRWLSYLYTICFLLLFAVLSLFPNEVIYVSYRILALIEYFFLFMISFLSKEEYIYESYKLIYGLDTIPGAKLIFQNLEYCLKVYFFVSVFTGSFFTGISICFRIQEACSITNSFAVILTILDRTARDIGAYSLIMFIGLLYSRVKLLRNYLDTKSANTAWDRYSVKQYINMYESLTNTIDDSAVPVKVTVCFSCTLLL

>HarmGR34

MTDDTLVCDIKNVLFIRFAFGFRQNFNGSSKIKRLSYLYTIFFSLLFTALTLFSNDLSYHSLSYLILALTEYFVLFTVSFLTKDEYIQRNFKLIYGLDTLPGAKKIFQNLEYFLKVSFVLGLANILFFATMICFRISGLCSIANLLSFFYILLHRLACDLGDYVLIMFIGLLYSRVKLLRNYLVTKSANTAWDRYSVKQFINMYESLANTIHDSAAPVKVTVCFSMYSSSLELSIN

>HarmGR35

MMIDSLVSSLQYIMFLRFIFGFRLDCNSSPQMRLFTKLYPVLFFIVLNFAVWSSENVLNPTLRYSTVIEYCAQFFVSFLAKQEFVNKNYQFIYGIDSLPGAHKNFKRLRVFIKFFCLYYILLRLLAILQLVVYVGPLLKNVTADIIYFHVCDIGRLNIFLVFCILFCRVKTFEMNFQTITDVVVLDKYSVKKYINMYQTLIDYVESMDMLFKLMVIFFIRYNDRFR

>HarmGR36

MIGDSLVCNIKYVIWLRFAFGYLPNFHGSPKMRAFSYFYTIFLFISFTTIVIAPFYKFPWFFRVLALLEYTTHFLLAFVTKDDYLYQSFRFIYGIDTNANVRKLYRNLEVFFKFIILYFLANKILVVMMLCYRLPSICLFSNTLDFSVNIIIRLACDMGRFTVILSIGLLYVRSKILKMNFLTQSPNTICGRHSVRNFINMYESLINTFDKIKTPTNITVCFVITY

>HarmGR37

MIKNNLVVNIKPIMIFRSLFGYRQKFNSPLKSNFFDVFSLISFFMWSLSFFFKTYYFPLVYVMSDNIEYCICFFVAFFTKEEYIYFFYKRQSSIDNLPGAEKLFSRLNLILRAFVVYCFLTKIVVIIILRVFAPEIFQLGLYLDIVMGLSQSMSCDMGRFTIILMVGLMYCRMKIIKDNMDMIGSDLRNRFVARNFIQMYQSLVCSLQRIDVPLKVSVSPLFSINQK

>HarmGR38

MTDDTLVCDIKIVLFIRFAFGFLQNFNGSSKIRRLSYIYSIFFLLLLTALLLAHNELVALSYRIMALIEYLILFMISFLTKEEYIHQYYKLIHGLDTYPGAKKIFQNLENFLKVSFVLGLTNNLLCASFICFRYPKTCSIATPFFFVPIILHRLACDVGGYTLIMFISLLYSRVKLLRTYFDTKPANTAWDRYSVKQYINMYESLTNTIDISAVPVKVTVCFSMCSPSLVLSIN

>HarmGR39

MNSNKKVCNLHHILYFRLIFGYYFKQPTLKLRILTKLYIALFISGLTWLFITKVNGMLYYLQYCDILEYITFIIYSLITEDSSLLRSYEEKIKIDSLPVAKQYFRQLEKFLYLLLFLICGLRLFASSLFCIYAFEICKMAPFGVSVTNTFLTAMDWRHLNTVIWFSLLQTRVKILKNTLEIQGFDRGPMQRFSPRMYIKMYEELVGFSEFNGYAMKSIVRITSH

>HarmGR40

MTDDALVCDIKNVLFIRFAFGFLQNFNGSSKTRRLSYLYTICFLVFFAVLSLFPNEVIYLSYRVLALIEYFILFMISFLTKEEYIHQSYKLIYGLDTIPGAKKIFQNLEYFLKVYFFVSVLTGSFVIGIVIYFRIQDTSSITNSLIITILDRMTRDIGAYSLIMFIGLLYSRVKLLRTYLDTKSTNTAWDRYSIKQYINMYERLSNTIDSAVPVKVTVCFLMHYPSLELSIN

>HarmGR41

MILKYLYKINSLLKIIYSVNGIIITRFTLGFYTEFCSNKKIILLSKIYVVLTILNVSVNHIYTKEYQNISATLSLSFFAIIYFMNSTASLIIDSKWIQKYVNDLKSIRQSMVADKCFDIPISRIIMIIYQAFTLMMILVYCHYDICRHHIYLRVIEGYYAVTSVTTIVPTLVVLELMFYAIRENRKCLEKNLSKFNTSGSVDDLKKNFRNCMSNYTKLRDCLKSTDAPVKTMVSMNEV

>HarmGR42

MTDDTLVCDIKNVLFIRFAFGFLQNFNGSKIKRLSYLYTICFFILFAVFSLFPNELIFLSFRILALIEYFILFMISFVTKEEYIRQSYKVIYGLDTIPGAKKIFQNLEHFLKVSFVLSWTNNLCFSSLICFKIPEACSISYLYSVSPLLLHRLTCDAGRYTLIMFLGLLHSRAKLLRNYLDTKSANTAWDRYSVKQYINMYESFTNTIDISAVPVKLTVSYSMFSPSLALSITLSR

>HarmGR43

MCILYGFSYIESEDPLYTIIFTFVDYMSCDLGRFSLFIIFGLFYCRTKGFRLNMEGEGTDIPWDSYTVKKYMNSYQALYNSLFIAYKIIITVVRFKAYLFVARLFLQVG

>HarmGR44p

MIGGNLVCNIRSIMLFRFAFGYRQKFNGSFKLRVFSYIYPITFLIICNLDIFIISTLSLYXIANILEYSIYFFVAFLTKDEYVFKCYEFINGIDNLPEIQKYLNYLNKIIKLGPWLFVLKNSIMCIIYAFYYSESEDNPLYIIFFTFVNDMSRDLGRFSLYLIFGLFYCRTKGLRLNMEGEGTDIPWDSYTVKKYMNSYQALHNNLFIAYKIFIKVVSYKVYLFVFFVLQVGHRKIFIA

>HarmGR45p

MKSMIFFYCINKALLMYSLCILFPNICHGPLKLVFIADHIYGMGADLGRFGFLLVLGILICRLKTVSMSFKNIATEVSRDKYAVKKYMNMYETLINYVEEIDTPIKFTV

>HarmGR46

MVKQFINKLKTQFQYYFGLNAVLFFKLIFGSYYDFSSSVILRRFAKAYCIFVIIAYWFVIYFIWTVNSKISLAFFLIVMTVDAAANILFSFITEEKYVIEFSSMVLFESGLNDRNIYFYLQIFHLVIVISSYLKRYYSIYAYAVAFVNISAYNNRIISFYVMNTFKDAVRSLRLSLSKHFKNKNLTSDQKLLQINKFLNAYMKLLRIIDKVFKIIRFKVSFCTICDIIFLPASNFLKCFVCPVLDCLCIDSRLLQNIRYIIPWISICISGKCYFALFIYLYAYR

>HarmGR47

MFQQIMIKIKNKIRSFYSLNSILFVRLLFGLYYKLSESIIIRLFAKIYCILYLILYLIFVKIYNTTDTDNFTFEFYGSVILIETVTNILFSLFSGELYVMKFLSSLPESDLYSFGVPLNLIICHSIKVILEYLFVPIPVFIGWVYLSVHMTIYNSRMTTVCVMDMLRQAYKSVSGTLIRNIMRRNVTDEEKITEIKIFVKGVMKLGHNMSIDINITRFKVRF

>HarmGR48

MLQQLLINLKSQFRSYPGLNAILIYRFIFGLYYDLSSSAILCLFARLYCILILIATVYTINFVKAISPLMSVTFYAMTKIINAIVNMLLSLITAEKHEIEFSSKVLSETGLRHRNFYRNLIFCYLAVNVSVCIEHSEAPTTYPISFLNITALNSRATSGYILQIFKDTVRSYRLSLINNFKNKQLTSQEKKYELNKFLNSYMKLIINLERVLKIARFKVRQLNGNFFITGLGK

>HarmGR49

MIAKIKYILKQRSPWILKLVTFLCLFYGQYLKISESKIICGFLRVYCIFISSVIIWGFYISYTKNVIYYGFAIIEYVFTIVIYFILYNDSIFKFYNNLEIYDRIMGFKEIPHFACYMIMFLIVNAIFRPITGFFRSTYIFSTWIQRISVGGALCIIEMNMFGIAHFFSLFHQRMQLMRKFLESNSVPVNITGMDEVAVSVRNVKKSLYYYDKLLDCLQSLDIQVQIMVIVFIIFVF

>HarmGR50

MEMLSKLKLLLSSCVDHFLPILRLGFRCYSRISDSKHVCYIAKLYTVIIFIFSTSGFLYWHRSTVISILLATQYCVHAIYSFVTSDKQAFRFHRSVKTSDAIMGFKNLPYISKPVLVPIYITVLLWMFYVYYFWIDDLHLSTYIMTICSDLNTVTVGILLIGVFSRMPIMKIALENNFVPVNIVGKDQLQKNVKIVRKCVGYYSNLLDSFDVIHTQFQFTVSDFMKCASCHYIVQ

>HarmGR51

MQEVKKLTKTQISAMWLVIFFTSARAFLGCYKKISESKLICFLFRIYCIFISFTLVAYYFRDNIDTGSSSHMLVIIEYIIIVIFHMFTGDTYMRTFVNAIKMNDRIMGFKGIPTFTKYVYLLMFLGTVSKTVAGSFRYMYSLATLLRCLTIGAILVSVDFNQLTIIISFSMLHYRMKVLRRFIDSNSVPVNITGRDKVAISIRNTKMSLFYYNNLLDSLQLINKELQFLVKNKFCY

>HarmGR52

MITKIKYILKQRSTSKWIIKLATYLCLLFGLYSEISESKIICGFLRAYCIFISSLTVWAFCVTNTTNFISYVFVIVKYVSAVVVFIILHNDSFIKFNNNLEINDRIMGFKEMPYFSIHGFMFFMCNSIIRTATGFSRNTYIFSSWFQKLSIGGALFIIEINMFGFYYFFSIFYERIKLLRKFLGSNVVSVNITGRDEVAVSIRNVKKGLYYYDKLLDGLQCINMQIQMMVNAFNIFVFDCTLCYYLYYTIAIIAT

>HarmGR53

MISAIKKYVKNDKLFMWLTKSYIFMRLLLGVYVKISESKLICVFIRFYCICIFFVFASIFTYNPENYSVLFEAAEYVFLVIYHFICENNSFVFIDVIKINDRVMGFKETPLVNKYLSFYMVLNSVLRYTTGQLRYTYHFVSWNEHISLGIALISVDISMWGSIAILTMIHDRIKILRKFIQSNKVSVNITGRDEVFISLRNIKKSLHYYDKLLDSLDLVNNQLQFQVNVLFRIVHIKYIC

>HarmGR54

MPQYSPTAILITIQYSVTVIYGYITSEKHAIRFFTHVITSDAIMGFKNLPFVRNAVLVPLYIFYLIWGFALYYRWVDLIRLPTIIMLLATDFNALCTGILFFGLYERMQLIQRTLENNFVPVNIVGQDQLQKNVKIVRKCVGYYSNLLDSFDAVETQLQVMVSGLMKVIEQIRCSSLHKIATYEGVVLFLKIYSVIFLGSICVALPRAVLVAGI

>HarmGR55

MDASKKLKFILEVSIDIFLPLTRNLLGYYTKLVDSKYVCYFCKFYCVFVNFYLVTQTYYLHGFFSVRDLIVFQYSVGAIISFLKADVCGVVFFKHLKTSDAIMGFENVSFGKRSVIAPLYFLYIARQIMVYIIWFDAMSFPVYFMCAASDLNSLLISIYLISIFGRMKLLKKTLENNLVPVNIVGKEQLDKNVNIVRKCVGYYTNMIDAWNIIDNDMQIMLAVALLTNTPIWIMNFYAVVMLFLQPTGQQEAVKLVQGNILSLLMATSPTIVSELISNEIDAIKATLVTQLVRCSDPSLSSELEVALHYIHIPPFKFVICRAVPVDINMPITIIGFCITYVIVFMQFLHFSNL

>HarmGR56

MIVKRNNFIQNRQPSLWLYKFITILRCLLGNYREVSKWKPICYLFKLYCIFICSVIVVSFYLSHYSSNFTNYIVILEYVAIVFFHFVCGDNYIMSFFSDVKINDRIMGFEEIRLPNYVCYVGFAGLLLRSTVSIIRIPIVGNSLRYYSITSAILSTDINQLTVIVIFSIIQDRIKTVSTFIASNSVPVNITGRDEVAISMRNVKKSMLYYNNLLDSLQHINKQLQFLVTTFLTILKIFGNLVGFIPCN

>HarmGR58

MKVNPATVKKQESKPNMEQLEESEYNDKIAKVMRSMKTITILEYSYGLFKFQFTNGQLQPINILIKMTAFLSIAAYIFMLYINVCLTNGTFFLGSYTVIQFVPSMVVLLQYIISTFKNFSISESLLNIRIFTTIAKLDSLLRVEVLNDFYEILRSKTNLIMLIFVILHAANFVLEFLTSNKTLWLIFISYHLHMTQEIELLAFFNCVSMITYRIQLINKFLTFFNNGQEQRDLTVFIVREKNNEAQEKLNFVGRVSETNVKLRDLATMYDIIGKICLMINKAYNLKLFMILVTAFTFILVTIWQALSFYQSPQYCTQDFVKLSLWCFSTVCNLSALAFVCERLLRARNKTRILVNKIIMNYDLPKTMRVQAKAFMELVEAWPLRIYIYDMFSLDITLLLKFISVATTYLIVIIQIFHFI

>HarmGR59

MEAEYDNKNKYFTRMTLTKSHEKQIIETLNIFIIFEYFFGIFRFERVNEELREPNWKKKVLSFCITSTCAISFVAYSVITLNIVMRATMREAIYINLTCITTMLLQFCSSALSSTFFFNFTNIRIITTLANLDVMLQIEDYKNFYKKCLFTTYKYVTVVFITQVTLSIIDGFTMYLGWAVPAAFLDFSQRLTILTFCKYVDLLRRRLKIINNYLKAFTDECEKETITVFTLRSRTNKTTQAINFIGHASDNNTKIRDLSRMYGMIGQACSMVNKIFNFLILTILFNSFIFIITIMWISLVMYRNSSDNLGLYINVVLSFFCWISYLLFITITCQRLVSLRNKTKILLNKIVMNYDLPTTMRDQAKAFMQLVEAFPLRIHVYDMFSIDISLMLKFISVATTYLIVVIQIFNFF

>HarmGR60

MKEKIKGKLNSSPEKFNIRSVKRIVDTINVIIKAEYFLGIFRFTIVHGSLREPNWKLKILSALIVLICSTLFLLLSESYYGLSSHLYRNFTDSTVINLSYFVMMAMQYSIHAITITFCFNASNICIINMLANMDTMLKAKILNNFYKKSLFQSYIYVLLVVSTQFSITTLACCTLNTSWSLIAGILDIVQRLEIVIFCRYIDLLRQRLGVINNYIKKFVSEQEKPSALVFTIRNRTIETTETINFIGEASESNDKIRDLAKIYGTIGHTSSMVNKMFNFQILTVIMSTFIFIIAIMWTCLKFYRNNYSNTGYLLNLILSTMFWISYIALMSITCERLILLRKETKSLVNKIVMNYDLPTTMRDQAKAFMQLVEAWPLRIHIYDMFSVDITLILKFISVATTYLIVVIQIIKFF

>HarmGR61

MQRFVMKDNNEVKAKEPSESNMEKQEENKYNDKLAKIMYTKKPVTILEYCFGVIKFYFRNGQMHAPNKIIKGTYVFFVVLYTFVLFEYFYEPIQTNDLNSKIVLAALPGIIVFIQYIASSIKNSSNSYPKLNIEIITTFAKLDTLLQVEDINNYYNISRSKVNLMVFLFIVFHSVNLLLEIMTYTYQIWPPIIWFHLFVTQKMEIVNFLHIVYMSTYRLDVINKMLRTFILENRQKDVTVFIIRKKIKNTQTKNFIGSASEDNEKIRDLSAIYNIIGNNCSLTNKVYNFNLLMSLVTAFVFILVAIWQTLSLYQSSSQYDMKDIMKVTLWCCNTVFNLAALAVVCEKLLRTRNKTRILVNKIIMNYDMPTTMRDQAKAFMELVEAWPLRFFVYDMFSIDITLILNFISVATTYLIVIIQISHFI

>HarmGR62

MENNMTEEKNQENKQNMEYSEENECKIKIQKVMYTVKPVTVLEYCFAMFKYNFCDGQLQPTKTGMKIYSSLCIIVYALVFFRFFFMPAVGYPYITLVPPICAFIHYVISSFIAFFLSGSKAYICIFTTIAKLDQLLQVDIVKDFYKKSRSRTNILVFIAVSIHALNCFLEIIGDLKEYVMTLTSFHLFFTQRIELASFFNCVAMVTDRLNVVNKFLDTFVTEQDKKDITVFIVKERKKESKETLNFIGRASENNVKIRDLAAIYDILGRTCMMINKAYNFSLLMILTNSLAFILITFWQALSFYQSQEMDSSDLIYMAVWCMSTISNLIALAFGCERLLRARNKTRILVNKIVMDYDLPRSMRVQAKAFMELVEVWPLHIYIYDMFSVDITLILKFISVATTYLIVIIQISHLI

>HarmGR63p

MEFQLEEKIPSYRNTNKRRGEEQIFNTLKIIIKIEYFVGIFRFTLLNEKLSRPNWRMKSISIFIITISVVWFFSFAAYNLELPSVDDVTSYKFMNLICIIFMFLQFFASASTTFTFNTSNICIISMLAKVDTMLKVEILSNFYKKCSLNTYIYLTFVIATQILISIIDILTVRISWAITAGILDFVQRLEIAAFCSYVDLLKCRLNIINRLLKTFVDDQEKKATAALTIEFRSGIIENFSFIGQFRENNTKIRDLAKIYVMIGQICSKVNEIFNMQILTILIPLYL

>HarmGR64

MEFQLEEKIPSYRNRNKRRGEEQIFNTLKIIIKIEYFVGIFRFALLNEKLGRPNWRMKSISIFIITINVVWFFSFAAYDLELPSVDNVTSYKFMNLICIIFMFLQFFVSASTTFTFNTSNICIISMLAKVDTMLKVENLSNFYKKSSLDTNIYLALVITTQILVNITDFLTVHISWAITAGFLDFVQRLEIAAFCIYVGLLKGRLNMVNHYLKTFVDDQGKKAATALTIEFRRGIIENFTFIGQVRENNTKIRDLAKIYVMIGQICSKVNEIFNMQILTILMNTFITMIDMMWTCLVMYRAPTSPKLGVLINVTLSCCTWISFIAMMSIRCERFLYVRNETKILVNKIIMNYDLPTTMRDQAKAFMQLVEAWPLSIHIYDMFSVDISLTLSFISVATTYLIVLIQVIKFF

>HarmGR65

MEFGKDQDNESNIDQDYSNQRKKIVNIYNNIKRELIFEYFTGIYRFQLIDGELRTPNWKLKALGIVIVSIYTAAFIWFIIPDPSDCMTGLHVFLNNIDDFPCIVVLIQYIASMITCNFLMNSKNIRSITLLGEVDTILQVEKIEDFYKKIESKLNKYLILLILTHFIHGVLDVFSSDDIVWEMTILPLYLNQKIVVLIFCSYVIMLNSRLRLINSYLREFIQEQDKRSVPVFTVRGTKTKNEKTLNYIGRPSIRNTKIRDLATTYDIMGEICFMVNEIFNFQIFISLVTTFTFIVITIWTSLNVYRKPDYQSSQLINVLIWCMNMICNVAAMSFACERLLVLRNETRILVNKIIMNYNLPKTVRVQAKAFMELVEAWPLRIYVYDMFSIDITLMLKFISVATTYLIVLIQISHFI

>HarmGR66

MLDALLLGMVWAFVAGIITFVQRLEIITFCKYIDLLRRRLQIINQYLKTFANEQDSKYVTVFTMESETIKTKETVNFIGNASESNTKIRDLAQMYGMIGHTCSMVGKIFSLQILTILMSVFILLITIMYSKQSHRLPNFNGCLWSNGKCSSFIFLLDFLLGFHVIQM

>HarmGR67

MANVKKVEPNPKVENKKCDQKRTMLAIINTIKPMLFVEYLYGIHRFYFIKGQLRPPNWIMRAYAVIHTGSFLVLFFAFLNFPAVFSGSMKIVEIMDEFPPIVVLIEIMSSTIIATFVVNTVNISIFIKLAAIDAKLQAESLSDFYKKSRFETYVLLLALSVSHFINSVIDFVTADDITIKGMIVLPLYFVQKLETFTFCKYMYMVRRRLTIVNDYLRAFVEEQEKNSANIFTVTNNKVEKKREVNFIGRASDTNTKIRDLATMYDIIGKICHMINKVFNFQIFMTLVSTFTYVVITIWTSLYYYRKPGSNLGELINTAVWCCSAIYTVGIMSVSCERLLLVRNETRVLVNKVIMNYDLPKTMRVQAKAFMELVEAWPLRIYIYDMFSVDITLMLKFISVATTYLIVIIQISHFV

>HarmGR68

MDDDKEQDNESNTVKDDVKTDENPKKNMVEIINGMKTELIFEYCCGIYRYQLVDGELRRANWKRKALGTLIILIYTIVFFWFLFMDPDDDDLHFFMSTIEELPSVVVLFQYVSSVIANNFIFNSKSIRVITLLAEVDTMLQVEKFADFYKKISFRLNKVVIFLIISHIVNTAMNFCSVQDVAWGITVLPLYFIQRVEIFIFCGAVYMLKCRVKIINNYLKEFIQKQDKKSVTVFTVGKAKPKPDTTLNYIGRPSIRNAKIRDLATAYDNIGEICSMITDVYNFQVFLTLVSTFSYIVITIWTSLNFYRKRDYRIRQLINVMIWCFNMIFNVAAMSFTCERLLVARTETRTLVNKVIMNYDLPKTMRVQAKAFMELVEAWPLRIYVYDMFSIDITLMLKFISVATTYLIVIIQISHFL

>HarmGR69

MEDTTNEDTNYAKEKEKLISESKKQILDTMHLINVIECINGIFRFSFVNNELLPPNRIMKMLTVFCILIYVIIFVFCFISLSPLSDDEFDVVALVIQFSITLDFLQYAACTVTATFLVNSNYIRIIDSLACLDTELEIGKLSNFYKLSRFETYKYIFLVVFTQGLRAIADWFSDASTILYTLNFLLSFIQNVEVMTFCKYNDMLRRRLKVINQYVQVFADEQEQDTATVFIVKPKNEQEKEELHFIGRPSDDNTKIRDLAKMYNAIGQICTMVNEVFNFLILAFLATTFTYIILNMWTCLHYYRIGLNDLGMLINITSSFFFWILYVVVISITCERLLLVRNDTKIQVNKIVMNYDLPKTMREQAKTFMELIEAWPLRIHVFDMFSVDISLMLKFISVATTYLIVVIQMFNLM

>HarmGR70

MEPIDKTEIRNRAAKILHTLKPITIFENVYGIFKFNLVNGDLRPPNWKLKTVAVIYMSIFCYLYVVKHYSDQFVESTTKWSREYIMKQVPAWFVLIQYVVSCLKASFPDKSKSDIHIIKTFAELDCLLHFETLNRFYAKSRSRTNILVLILLIYHLLNFVLDIFSDYDYPRVFLEAHIYLVQKLKIVGFFRLMYMVTHRLDIINGCLNKFILEQERANTTVFSITKRQKRMKDMFNFIGHPSESNVQIRNLALMYNIIGKQCCMINELHNFKFFMILLAAFGYVVVTIWTALSYYQTQQFNATGTVIIAIWCLSTICNLTAMAWACEALRRERRKTKISVNMIVMDYSLPKTMRVQAKAFMELIEAWPLRICIYDIFSIDITLLLKFISVSTTYLIVLIQIYHLI

>HarmGR71

MFVTIKKKPFNILGNNHNIIEALSRTNFFRRFSGISVFTLKLSADNRVIRGFSKFGFTCFLTWLSLYIYCTYRAHAEDQTVLRVLFSTKVQRYGDDYERISSTIYVIFAFWKIPFRLNINNGFMGMVVKVDKALEELGATVNYNFDALLALSMSISQTFLCVTRLLSVWLTLRHLGVPVPSEKMFQVILSDSLALIATAHFSFFLTVLRCRFRHMNKILQDIKNHKSWEHKLFIRGSMFSNPQKAVSLQDKFICEKIKACANIHAMLYQMTDLTNKVFGSILMVTILIYQTYTIWFMFSFMEATAAGLFHDVERYVVFCINVFWEIGYATFITFMVIYVSERAVYEVSVTHRRA

>HarmGR72

MSATMKVPVKNKYFSYPFKLDGCKNIVEALSRSNFFRRLGGISVFVLKVGSDNRVTREISTYGLLFYIVWYAIYVYCTYKAHYEDQTILRIIYSTKLQRYGDDYERISSTAFIIFAYYKIPFGVNINKVFISMTVDLDKALENLGENVDYRLDALLALVISISQIALSTTRFFSIWLTIYQLDVPIPLERMHQVICSDTLALIATAHYCFYVKVIKCRFQHMNKILEDIKNHKSWEYKLFTRGSMVANVQKAKGLEDKYICEKIKACGKIHSMLYKMIEANNKMFGSMIMLTVLVYISYITLYMFYFMEATAAGLFHHPDKYAVLFVYVLWEIGYASVTIYVIVYISEYAAYEVSRK

>HarmGR73

MKFDHNVSLKQLMWLKIIFFQFCNWSSSVYVSVFAKLYCISNYLFIFIYGSIKMSHFNQYSASIRLNYITSIVELNVDTLFSLIHGEEYVRRFVKMLEKDFPGLQKFYKYYVTIYLSLIGLVGFLIFFTMDIRGGDNILKPCLFFNRSVACYFCRISVIYVIEGYRNTVSILRKQLNTQLNMNNITEAQKTVFIKEFTHSFIKLTEHFDSAMTIIRPLTIFRFVIDFCKILNIIYYISFFDISSFVISWTFEVIIEMLSLCCPMLILESAADDLDEIRKIIAKELLTYEDYYLRSVIYESMEFVDGYTTGLNVWNQYPMNKDMVLAFIGLITSYVIALLQFSY

>HarmGR74

MNITESKTICYFMKLYCICFSAVVIANFYFIEVHEKNIYSLIFVSIVEYALISSLSLIYGFNLFLNFFTAIKTNDRIIGFERMSLITRYSYLIIFVNSVMRYVITVSFGSTYIHSLLEHFFVFLAAIALDLYYVTIIILFALIQNRMKCLRLFLASNSVPICITARNNVANSIRNVRKSLVYYNNLLDYLEGLGQQLQYMVHFLFIYFFVLLELVKDTISYLSLFFLLQIFVNWVCYTSRVIILLYSITVFYSTGVS

>HarmGR75

MITFQSFLSKDLTLRMLQIIAFLRLFYGNYVTISESRAICYLVKLYCIGVCSFIALFGFYAITQLYSFTIISFTLIDYFMTALIFVVFSDQPILHFFSGVQTQDRIIGFKKMSYINKYMYFILFCTSFMRLAFFIHRIITSYRSNLEFILVVYILTATDLNYLLLIIIFSVLHNRMNRLRLFLESNSIPINITGQNRIAISIQNVKKGLIYYDRLLDSLQSLDKILQCQLMVYSITQFLRISMMSYRLIQQFMVEYILLRSTINVLELIPTALFVFAPLIFVEATTYEVERIKTILTAQILRSSDECLRFELQTALQYVRLRPFRYTLCRAVPLDINLIFTTASLCITYVIVACQLVYFSK

>HarmGR76

MKPIQNFLFRDNATLSELLLRTIAYLRLFLGNYVHVTKSKGIRYFLKLYCICFFMSVMSIYVYTSVPFQFTVFSFTVFEYILTAIVFVVSDDKHIHDYFINIITNDRIMGFKKMPYMTTYVYLILFSSTITRFCLCIIRIHTNIHTVITFCAILISITAMDLHYLLVIFLFSILHTRMNMLSLYIESNSVSVNIIAHNEIAKSIRAVRKSLSYYDKLLDSFENLAKILQYELIIYWVTEFLRASIITYNGLQGLISSKGEHLVLQHSNSVMTLNLMEVIISVALISFPAFIVEATVYEVNRIKKTLTAQILKCSDECLRFELQTALQYVRLRPFRYTLCRAVPLDINLLFTTAALCITYVIVALQLTHFAA

>HarmGR77

MIPERQLPNQQIVSEWVLKIIAACRLLLGSYMNITESKPICYFMKLYCICFSAVVISNYFTEVYENHTFSVFYVFIVEYICMSFLSLIYDYKLFLDFFTTIKTNDRIIGFERMSLITHYTYMIIFVNSVMRFGITVAHGSTTTKSDLGHLCILLTFTALDLQYLTNIIIFALLQNRMKCLRLFLASNSVPICITARNNVANSIRNVRKSLVYYNNLLDYLEGLGQQIQSMHYTDLFLGTLTIGVVHSKVMIYSPALIVEVTIREVEKIKAILTTQILRTSDECLRFELQTALQYVRLRPFRYTLCRAVPLDINLLFTATAFCITYVIVALQLNHFAE

>HarmGR78p

MAIQYSVHAIYSFVTSEKQVIRLYTSVKTSDAIMGFKNLPYVSKAVLVPIHITLVLWMLFIYLFWIDSLYLPRYIIAIASDVNLLTVSLLLFGLYSRMRVIQNALENNFVPVNIVGKDQLQKNSNPMHGFTLAIINMASIIVSTSPAIISELISNEIETIKATLVTQLVRCSDPSLSSELEVALHYIHIRPFKFVICRAVPVDINMPNTIISFCVTYVIVIMQFIHFSSVY

>HarmGR79

MNPKLLFQFKSPTLCLIQIIALLRLLLGNYINLGHSKLTRFLTKLYCVCVCSSIVSMYILSLSERALFPVYSFIIIDYTCAVILSIIFGYDRYPAFLAANMTNDRIIGFKINLSLTNYAILSIYGSAFLRICLIIAHFLSHPSSILAFCSITCAIAATDVNYIAVIIIFTMLHRRMKNLRTFLETNSIPINITGQDEIAISITNVRKSLLYYNNLLDNFQSLDELLQYAVSKYTIWRMFQITINWITNFSRTCVVVYFFVQHIIAQLYPSLVLISSPAIIVETIMYEVDRIKETLTTQILRCSDPKCYTSIGISLSSVCHQTSVSGSSCRRRCSTCACVRSDTRSAARCRSTSTCCSPPPHFALLMSL

>HarmGR80

MDRASTFRSKLNVKRGTKLNPIFTKNLHSEVLLDNFIEKDLQSLLKPLNIMYSVFICAKYSIRDNFITCNNFLYNFIGVFTTGLFLCISVYRICSTLSSKRIQYVIFEWNTIFNFMSYSLGLIMNYIINIRFSDKNIQLVVKIQSVLRVLKMNRKDLTCLLVYNWSGVLAINALIVGSIAYMMYIFFPHVDILDVIYFYSTIVFDVNIMYSLFVANFSRKALCSWINDVLQSGDDSDFYWNRMFNVYLNMLDIYTTLEVVFQYSVRFHYLEIFLNY

>HarmGR81

MHNLQPKKMLKMKKIIQKREKSFDQPNRIGFDYQLDKELHQIVNSFNFALNLCFSSKYYVQSNHIEARGMKYRLLTSCYTIIMGLLCIYRIVTADIRDALMSYSENCFLRFLSGLYYTDYLLGFILCYILDIVHSHNHIILVAIFKVIHRSIDCSKIVSRFIIWNWITLCTTICIDLFIYVMYYGFFARFSLIENISDCLCDVMFITFYINYIIAIRVIILLKVYLDEWILNIRNLSNGLDRDEVCLKLIRVYNDIMKAYDLYKSIYQLLVSSLMNHLFQKFSVAFLRECFIFADSNSRTGHIFAKFNVLLSDHHKPQEECTGRR

>HarmGR82

MSSITRYDVGTGNSCHSVNSRCPVNKVDKDLQSIFLPLNLLQILVLNPKFYIRKNLVKPNDCLQKLILMCGLVIFLSGYVYRVTEIILDDNLKRYGSINFLYYASYFDFVFYSSGFIMNVIIHCKQSSKMVTFVLIFQKIHVFLNTGSIKRSVIRNWTNVTVIFVFYILVMLFSSLSIYNQASWNFALNLLYLASLDSNIIYAISLMRLLVDKLELWSVRVLMTSTDGNDVTYRYQMFEAYKQILKCYDLCKDVFQQQVSQVVNLNAYSETYIDSVES

>HarmGR83

MYVRSVEDIPTLPINRLHINNMVDNDLQALLRPLNLVPRLLFCASYRIRHNFICPNSFLYNVLVVFYFVSFRCLALYTVIYLCIYIVDFHGGSKIFFTLFDCLDFTIFSIGFLINTYVCFKESDNNILLILKIQYVLRNLNVSRLCLKSLIASSWWSVILIHAFFIVSGTYYCYYFSELRITDVLTQYPTILFDVNVIYASLLIKLLEKTLRVWIEAVQKQNIDNSERERHLEMLFDVYFNIQGAYKIIDKTFQIQVNIYFHVLLIIKNVMTMLLIKNYESIKNIKYFVLVFISQTWLLKSIVVFIYLSVECERFYAAMRDVHDTSIMLMNSEQFAELDMRVYKNIHRARRSLFSKLDGCRLFQVDAELPLQLSRLISSYIIVCLQFAFL

>HarmGR84

MKFDHNVSLKQFMWLKIICFQFCNWSSSVYVSVFAKLYCISNYLFILIYGSMLMVYLGQYSASVYINYVTANTELNVDTLFSLIHGEEYVRRFVKMLEKDFPGLQKSYKHYVTIYLTLIMFIEISIFFIMDIGERQFLQSAMFFNKHVACYFCRISVIYIMEGYCNTVSILKKQLTTQLDTKNVTEAQKTVFIKEFTRSFMKLTEHLDSAMKIIRPLTIFRFIIDFCKILNIIYHVCFFDFSRFILSWCFEVIIEMFALCCPMLILEWAANDLDEIKRIIAKELLTYEDYNLRSAIYESMEFVDGYTMGLNVWNQYPMNKDMVLAFIGLITSYVIALLQFSY

>HarmGR85p

MFSLLNFKRKQQSDPVVLTKAPNIPYSEGLLDNRVEKDLQSVLRPLNLTLGIFICSKYSIRYNFITPNGIYYNICGFVCVVIYCSYSIYTTVSSSIELVQLKNNFNPRLHWSSIFEVFFNAFGLMLHYINNVKHRCNNTAIVLKIQFAIKVLEFNSNELKSLKFFNWLSIIILNGICLVSIAFFSYFFFENLRFFGILSFYCSVIFDVSLLYAACVIKLLRKLLAAWNDHVVRSKNILDSQRESYWNTMYDVYISILEAYMIVEKSFGALFTFLAIIAKAWLCKSVVLGIILSLECEKFYSAMKKVQITVTPLIQSTRYLESQLPFCKNIHRVQLADYEKMSACGLFTVDAMMSLRLFDFITTYTIVIM

>HarmGR86

MPFYTIFNSRIAARRRSLTTANQRHEIETNEGLGRENVVEKYLQRIFQPLDIMQAIFLSTKYKIRDNIITPIGRIYSFISISGEFGLMIVYYFLYIHTDIWTDNINHFIFGVVDFILYFCGVLLNPLVNVIQKLNNVLLVLKLQNIHRVLNMNENYFKHIIISNWIFSFTVSNFQLLWLCGYYYAFYNIGVDNILTVYICIRFDMNVVYATRTIKLLCKSLEKWTEDLWRSGYFEDSDDQYWDRMFEAFLDTIKAFHIIETIYQQTVGFLNSNHSDLKYGFLTFLSLSWLMKNLTLQTLLSVETERLYAAMREVQSSCILIPTLKQPSVYQRRFYKNIQRVQEISFKKMSMCRLVTVDAELPLRVLHTITTFTIVILQFEFL

>HarmGR87

MIFTNNKISAKTREPEVLLDNRLGKDIQRMLYPVNLILSLFLSSKYTIKDDYITPKGKKFYIATFFFILLLYGLGINRVFFEDIEDTMGTDNRDIVTIIFSFAFVFYSIGFTLIFVLNIIHSDCSISLILTLQKVFNSLDFSDKIVAITRWNWFAICIAFGTNVFLYMLYYVTYHDFNPVDLVMDIMFITFDINLVYGILVITWLRKILEKWIEDVLAFEDGDEEFYSEYFQVYRNILNAYNCYKTLFQLLVRIFSIFCFSKNLMYYFFLPVVYFLQVLFHTADTFFRCLCYFAIILQILQMPDSTVYEQMVQYTVVKVVAAVWQIKDVLLVVMQCLECEKFYMAVEDVETTCIQRLKKKHHLAAEERLCSSVLQANRTSYCKMSACGLFDIDATLPLDLIGLLTNYIVIMLQSFFL

>HarmGR88p

MSEKRKIVPYPSPMLPNTEIDKEMQGIVKSFNFVLHLFFSSKYCLRRNNVYPRGTKYRFMTVLHTSFLNGLNVFRAYRDSSIGVTVNDFMRIMNCCYDFVQMYAFILLFILDFVHKQNHVLLILAIQTINRSFDLSKSIRSFIIWNWIVLFITLCLESYMHIVYYAIYAHSRFSDVIPDCICDFMFISFNVNYIFATRIIILLKIYLDEWVKFILILNEREENNEYCLKLLEIYENIMQAYNLAKTVFKNLISVSHTLDLFFWVTKTMLLSLVHCAYCERFYISVEEAECACIQLIKNINCPKSHKYLCKAVIRINRSFSKMTACGLFYIDASLAICFFGAVTNYAIVMLQFTF

>HarmGR89

MLAYFKKKPKSKVNDVIIKTLPTVQYKEVLLNNVIEKDLQSVLKPLDLMQRLFICAKYCIQDNFITSNSRSYNILGITLAITLRSLLFYNLLRIVSSEDQNNYVLAFSNIFDDVFFSIGFILNYYSNIIQCNNHVLLVLKIQEVHRILRVNGKQLKCLIIINWVYVIVLNVVYILGALFYCLIVSFARILDPVSNYCSIAFDINIVYTAFVMSLLRKTLSIWIEDIRTSKHVAIESYWTVMFDVYSNILEAFKIFEK

>HarmGR90

MTESGIPLTNKLSKEVQQIFQPLYLMQMLVLNPRYQLSNNFIHPNMWLNKFILLTSLGLYVSFSLYRIFDIYLYFDLTMFLLGNYLQFASYFNLVFYCVGFIINFTNLFIHSKRYVAFVLIFQRIHTCIGNKASFKSAVNISRIYVSIFFGAYFLMSCFVITIFKQTSWVIVFNTAIMLTIDANMIYAITLIHLLTNEVKLWNSHISMFSKKGSDEDQHKIMFETYLHIAKSHRICRTVFQYMVRYFIILSRKCSNGIGLNSSVFLHVPRSYRIHLHFHGDLCLDSKSRGHTNSAGPQFRSLLPSYRRCTRFLRSHHDMVKQSYARTYCVSTVPVSAG

>HarmGR91

MITKITQMVKQPITEPDSSFRVLKDDRLDKELQHIVRPINFALHLFFSSKFDVRYNHIYPIGTKYRLLTFCYTMIMIIFCIYEIFTFDNLVEAAFDYQKILVKFLHIVYFTLYVVQFLTWFVLDNVQTQNIVSFILMIQVIHRGIGCSTEFRSFVIWNWISLFSVLCVNLIIHIIYYIFLDDLSNMTTFIAFLLDILYISLDVNYAVAVSFIRLIEKYLEIWTKEVLKMNAEKENGEQRRKLLKIYHNIMDVYDLYKTIFQYLVCLK

>HarmGR92

MLHIVRKLQTAIKKVKCASSYMNTDNLAVNVIEKYLQATLLPLNIMQELFFCAKYQIQNDFIYTNGLKYDIVSAIGTILYVSLSFYLISSSFNKIHNLLLDLVVFAIGSILNYFVNIMQKNNNVLLICSIQNAHRMLDINGMVFKRSLIFNWIYVIALNSFHIFWLFYYCYTFDDISIRDIFTSYLHICFDVNVVYAAKLLEINRKTAQIWIERIQQSVGNIVHCDSNNLFKAYLEILKSYQLIEKTFQYLVSLFVACN

>HarmGR93

MTVESCVKKNSITRPDQPVKALPGDFIDNDVKRMLRPLRLIHFFSFCPYYRLKGNLILPNSLCSKLLSFCVTMFFMFLFAYRCYDHRYIRQQRRNVAFHTINSYVLVLISCFGVLSNFISALQSKLNVKFVLKIQDVHAFLNDSKVFKRFIYRNWIFVISIICYEVFGWISVNTLLKLSYMDVLCGAASMSFNVNIVNATRLIVLLQDKLNLWNDRVLQLEGMESNSDTEDYCQKLYQKYIDIMECYDIHKLSFQMKVS

>HarmGR94

MLLENRLDKEVERIAFSFNLPLNLLLYSKYRLKYNRIYPNGIKYDIYALFCTLFLGVLCFYRIFTLDMTNASMSYMERAILTVVPILFFTIYFIGFVIVFVSDIVYKDNNVLLILTIQTIHRSISFSKTNHSFNMWTHISFATVILVNLITRGTFYLTCRYSHVSEEISDIVRDFSFVTTDVNMVIATRIIILLKQYIDLWIKAILTTNVAQATDLYCQKLFDVYMNILKAYKIYRKVFQALVSFDFYIFHLFI

>HarmGR95

MLSHLNKFWKPNKSATTLEHGTRESTENEPNSFVIEDDLQSILKPLNLMLGYFFCSKYSIRDKLITYNSYIYEYIRVLVVIIIYSWNFYNTILLNKKMLDQHWHGLICLGLGSMSGFFLSLIGDTIITWSNIIQKQCNILLVIKIQQILRVLQIYGNELRNVINWSWICVIVLNIFSMSYFICYCVTIEEINVIGIIISFASVTYDINVVFAFLLLKLCEKILRVWLEKIKLLKDNGDEASDEFWNRMLKVYLSLLDIYLMIESTFKHMASIS

>HarmGR96

MLTTKTMRQTQNNAEAVSCQILPKNRLEKEIQKIVCSFNFALRLFLVSNYCIKNNHINPNGKIYHTFAFFWMLFMSVLCVYRMFTLEEAVEHLEAAILVNLLFLYYAVYWLGFTVIFIQNLVNRNRIVSLILKIQTIFRSLGFLKDIQSYIIWNYVSLASIVCANIFVHVTFYISWSHIKFIDQIIDNVTNALFVSIHIYTIIAIRVIILLRKFLEEWMSVVNTMNVEHDNNELCLKLFESYKNILEAFNLFKEVFNLLVSNRNVYCL

>HarmGR97

MSQTQINVATVSFKILKNNRLDNEIQKIAYSFDFAVQLFLFSLYNIKNNHIDPNGKIYHTSGIIFMFLLNSLCFYRMHSIAGLRGKIEQFDLIIFLIFFYFGWYCVVFTMMYIQNILHKNYIVELILKIQAIHRNIGYKQSLHSFVIINWISIATIIISNIMCLITFCASGNYNDVIELVSDNICHFTYVAHHINVIVATRIII

>HarmGR98

MYQPNSRSDISHCKNLYNRLDDEILKIVYPFRCVLYLFFTIKYRIRNNRIYPSGKLYRIFAFCWMLFLNSLCILRISNVEVRNNGKTKQLEYSILLVLCTGFFVTYFIEFTLMFIFDMINEENNILLILRIQNIHRSIGSRKTIQRYITWNWISVAIIIFSDFAIRVLYYISSYYPHFMHSVYDAIIDAMFIALDVNIVITMRILVLLRVYLNEWINNIKTMKADDEEYRKMFQDYKNILLAYDLFKTVYQAFVGSVSTYLFNCCEY

>HarmGR99

MLKSQLVNSLNNFIDKDLQSMLLPLSLMQNFTFCPKFRIKNNRITPISYFHKFVAFVGTVMFIYFYVLRVYIQSFEKIFKNDFFELYTCCYCSFGIAINFIDSIIQTKLYVNFVLLIQKVHRLLNDEHHFRFYVISNWLRIIAVYGFFIIILIEVSVWIQMPYYYIASCYPMVAFDLNLVYAISLITLLKDKIILWDIHVSNLQAMQEENEPKKMYQTYVNILKCYEIYATCFERNVSGFFLVISLISLFLVLLPKLF

>HarmGR100

MLSSIKKTNLNKIALMVVKRPTVSSERRNKIEEDLESILKPLNIMQSLFICAKYSIRNRIITHNSKLYNALRVFCTCIYSCIYLYRMISQDHQTINKSWRKFWFGMAWIAGFILYMVGDLINTISNIKHSHKNILLVLKIQHVLSILRISGTDLRIFVCYSWASVIILNIFSISYVICYCFTTNIIIFEIISAYTSIAHDINIAYAIILMKLNEKMVRVFMEELESSKCDDSKIEEYWNRMLNMYLNILEIYNIIWKTFQQMLVLLPLMAQIWNAKNMVVLMCLSTECERFYTAMKDVETACTKIFKSRNCSVHLTRVCKNIQRGQNTYFKKMNACGFFYIDVHLPVMLSSFITSYTIVLLQFVYLD

>HarmGR101

MSPARFQKISNNRLERDMYRTAYSFNLPFIMLLASKYQFKYDRIYPNGKKYLLFNFVYMLFMNGLCIYQCYSVEMNSDRINFLGKIAIEISINFYIVTYFIGFTMMFITDFVFKNKYVLFILKIQSIYVEIGSRSIITSFITWNWIYLLLTIAINILLHGLFYSNYNERAFDVSIYIIRDFHLIVLDINVVLAIRIIVLLRKFIESWIQYISVKNDEADNALYCRELFGIYVKILNAYSLYEKLFQLMKPIIAILKVLLLVKDLGLIIVHSEQCEKFYMAVTKSESVCIQLIKNGHFTKYQKRLYKNVIRRNRVFSKMSACGLFDIDATLPIRFTEALTHYVIVLLQFNYL

>HarmGR102

MTETNDDSQTNNQLNMRPKYPKSKPCQVLPNNRLDKEVQMIANSYNIALFVFVSSKYYVKDNHIYSRERTFLLFKIFHILFTNTLCIYRMFTININTLGSMGHYEDECMKILNVVFYVTYFICYTFIFINDVVQQNNYVVLILKIQEIHGYIDCSEKIRSYVMFTYFGVIFTICIDSLIIVAFYMFFDTLYWFDIITTCYCDIMILSFNINYVISMRILDLLKLYLEEWTIEVLRNVGKNDDEQCIKLLKMYQTIIEAYNLHATIFEKLVG

>HarmGR103p

MSIIKTDIFTKMIIGLQLICGFYCKISTNKVVNALVRAYCVSIAATNIVLILCELFITIYRSGIGIVVFVATLYFLHLIIDLCSNCENFLKFVNNIRQPISQDLQSDVSTPITAIVL

>HarmGR104

MIGYHQFYRLRFFIYFRIFFGLGNFMFSPFQNFVLKLYSIVVAIIITAAGAIFIQYYGTDTFHRVVVLVEYFAYSLISFVAKGEDVRSFFQYLPSLDSFPGANREYRKMMNSVIFVLSFSIAWRITVTVIVLYLYSASLNTLVGMEIFFFIVITVAIDLGRASTFIYFSILYFRLKIFKTMLRSTDFNSTRNISVVYKFIQIYELLADKFRKIQKVLKLQVSIARQIV

>HarmGR105

MFEPQTVCNIEPLMYYRFILGFCQNFQHSRLTRILSKIYPIVLVVALIVKTFIFNDTATLYNKYFIGLEYSFNIVVSLVTTDKYINMYFKYYHTIDSISGAKKIYKNLEKLAIISVFYASSFRSWAFIVISRYNEAFFKGFDRSNIIELAVMYYVNDLSKITTLLCFTLLYFRTKVMKMALEATEFNDALRDRFAVNRLIQMYETIIDTFEIVAQPLKFTVRSIIMNRYYLGVQGRFRPRWLFHRKRSASWAGHIIVHEHVRRHKCTPYIPSLS

>HarmGR106p

MRKMELFARLCVIYSVFFRLYTFHVFCQNAPPVSANSFETFDIAVLVFLNIANDMRRIMTLFCFVLLYCRTRVMKIALDAIDFNDSIRDRFAVNRLIQMYEALVDTLKYIGYPFKVAVCIYPESVNRQKKTVILEIFLFSGYILFDLFVIKNNLRIDCKIITY

>HarmGR107

MNADLLKCFAPIHNVLLFLGSSRLKIKNNMIAPSTRYQKMYALCCIFIVTLSFSYIQLYYYLTYYHEDTTIYVCYAIGIMVQNVSYLSHTIFARFLDVESSVKLCQNLQKVDNILRLKQFKRYNEQQYYWNVVVLVFIITSFECGFLVHIWYTVEYPILAFFAGIGLLNVYMELVLAASLIVYLAIRLKFLNKIAHHNFKIKGYYNNTRAACAVDEHLLINSDVKDANIDLGNFLICMKEILKMYQHITQVFSFPVSRCTTRAKIIKVRASWRALSSRLLSF

>HarmGR108

MPRNIEELSEDLFTDDFIRIFQPILFVLRALGLARVSIKYRYPTGTSKWYLLYSNVFWLLNALSAVYFFFHCGESFDSKYADSTLKFGVLNSGINGILVVFRNNLERNNKFGAMYVKLQKIERHLNMEDTKSINKQLRSQSTIVMVVAFFVTIFWIVLFKYLFMKSMCIPLIVNVVTSIGLQTEMAQIYFIIKFIITRVNYINDMLRQVSLLSIEPLTKPMDDGILFVVSNTLKHQDGEVPGELVSGMHCIFETLSDFTGLFQFSLFYFICQILAWNLVTIHYLVTSMKEQGAADTDLLLCVMPVLVALQFIILTLCLKAQDLSTKLEEARKLCIDIISSPLINGKSREHAKQLTLLVEGRRSVSIYNICTFGTRLPLHLLAITASYTVVLLQLALL

>HarmGR109

MKNLKENIKIEYLSKDILDEEFMKSFSLLYYTQRLIGSTRVQIKHRFVTTPSFLQKFHTLISVVLLLGLDYLVIQKYDKILFDRETIYYLSICVTGLQTITFLCNIINVRFINGDANVELLVNLQQIDRRMNINRNKSITTLLVKTNVISLVVVLIMFITLLGVASAKGTAAFWPYTGIAYSQFSFVIELISCSNMFMYFYVRARFINSIIKNYIDQKGTQEILYSKERFLSSYFASKVFMRRLAAGSHNFVSSDTDVYLKQLLEGFFKFQDIYKFQVFMFCCKLVASALLTFEFLLYAVQNDTVGLWDSLTPSFFTVIDLVMAILLGVRCEVFIREVKETKRLVITVMSRHYDGRLREKSKRMLKLVEETPPHFSVYDMWQLDANVLLQMFMLVTGLIVTQMQFAFL

>HarmGR110

MISLKSVITIKTLMAIRLGISGLYFPITSNKIISLLLKIYCAIFTITVMYYILTCTYGLPFRYNLTSYSILTMYFANVIHTVFHNGDGEYLKNFFVAINKIDLAIGERPDDEIKISRLIFIVVFLVMRTTGMVIYCQSEYKKYCSLLRSLLLAGLFVSIAGQWCHTSYIMMFESVYHRMRLLRKRFENRLSASRQFEADEKVMENQLRQCLDIYKNLLGVTGLYGARIKIMVITQLFIYVHIFLAKMYILKKNLLIDLKIELIT

>HarmGR111

MSPARFQKISNNRLERDMYRTAYSFNLPFIMLLASKYQFKYDRIYPNGKKYLLFNFVYMLFMNGLCIYQCYSVEMNSDRINFLGKIAIEISINFYIVTYFIGFTMMFITDFVFKNKYVLFILKIQSIYVEIGSRSIITSFITWNWIYLLLTIAINILLHGLFYSNYNERAFDVSIYIIRDFHLIVLDINVVLAIRIIVLLRKFIESWIQYISVKNDEADNALYCRELFGIYVKILNAYSLYEKLFQLMKPIIAILKVLLLVKDLGLIIVHSEQCEKFYMAVTKSESVCIQLIKNGHFTKYQKRLYKNVIRRNRVFSKMSACGLFDIDATLPIRFTEALTHYVIVLLQFNYL

>HarmGR112

MLFNGLPNTNVSVNRSSKVVPITVKPTQRNNYVPSKTVSAIQTIKYQVCTALVFGINRLYLFKPNTFVLLLSYVYTIFLPILVWDIMFNADDLSATYFVFKYTCCIEYVLLISISVFTSRSKLVNLLRDLDKFDSLLNIRKDLKVIDSGYISVFWFCGCFIYSLCEYICCYFYLTVFIDRSVYCLYVMMLAHDCEQILFFVLLRTIYTRLRVIKAHVLKVFSAENRTNNYRRKLDKVEALSNNAQLDISSLHRVYDLLHKCAEQLNSIMSLSVSFEELFAN

>HarmGR113

MNLFYVLIFCENFMCVYRNYFDINKYQRIVAITRIIVELTLSVVITAHNVLLTKAVNYESTEKDVLLTMLFQILTLFKSIVIIIGGIMNSESFKQFYENLRKLYHCFENDVDYKMFEKKLRIKSLVGFSIFTFMSLVQMFGKIFQYYFLGTYQLTEIIVLVLYELWVDMRYSLEHVVVFCAISCISDFLKCLNISVNKVLMRFSEQPSLEPNSNEENVNLPEKVNNWTEKYQKIMACCKNISLCYQELVSNHHLPSLFMTRLGSASSLTACS

>HarmGR114

MSEIKLLTYLLHIENLLGIYRITNHHKNVKKYLITIQIFLISLLYTSVVVVEIDLNIRRVDGDDHEIDDIYLIFSSSWYINFLTSLASSVYYRSAFESYYESINRVYDWFRREKSNVTSMTKFQWCTLMFSSFTMFFNLFQPVEALFKYDFTYPLFVAYILLTVSFLKITLLFEHFVLFSIIILIVRVLKCLNHLVNAAEKRLRSQISDSECEIATKQIQEWASLYTDLANC

>HarmGR115

MTPIKLLNYLLFVENILGVHRKYNIQNKIKKYLIIFQIVIQTTFHAITLISEIYLLFKEEKWKNYIDINVIKSCYAVTAHINAISAVLTGICYSQSFLSYLDSITRVSDSFQDDRKLAKSLKKMYYLSISLMFLSISFVMYRVKEYLKRFSYMHPLIVVPVVVSQFFIRSTLIVQPLILFTIIMIVAHLFRSFNYFISVVNKRARSVDSLSEENDITREEIQNWVELYRDLENSCENVAIFFGHQYFFTLTMSISNSIMMVYHVGCSVLLKAITPDLRRMVIGLLNYVAWSMLPVISGQVVRNQAVKCHREFARLYNTVVIDPSEEKGKLIKDFIRVIKKKPLDIKLLSKLPIGMYMLPAMLTMGVNYAIMVLQINHII

>HarmGR116

MSSAKLFKYLLVFENILGIYRNYGNKNRKVKCLIIFQILVQTIYHIINVSGELYYLLQKRLSTQTFIELGFVLSANINASVTLVSGFLYSREFQKFHRTISLISERFKHEKSLKRSLKTLFYVTGIITGFLITSVILRAREVYVRHYSFSDALLVNFMFLPQLFTRLTLTYQLIITYVYVMVVVNLVKCFNSLISDGQRKVSRNTSVLVNCGCDVTKEQIQDWVELYQEFSNCCEDVTICHGWQASLFCICY

>HarmGR117

MADHLINLLTKTETIIGIYRNYDSLSKKQKILCKLRIFFEILFVFSIATYNVLVLENFLSTRIFHYMMMYHISNFLGGPIVAISGILCSNTYKNFIDNFMTMDMHYQKKSAYVKCLKKMKILFVVTCIISCLSIVFFLITKITARFFIHHHYVNIGFVLMLVVAVFVQQRFFLEHTLMYIFIRMIQNVLRCLNDCMLDAQVGYNDMTRSGQSDSREWRPLLTAEQVQLWAEHYKCLLICSKNLSICFRSQVI

>HarmGR118p

MTKIQWCTLMFSLFTMFFNVFKPVEAIFKYGYGFARPLFMAYVLFSVSFLKITMLFEHFVLFSIIILIVRVVKCLNQLVNAAEESLRYQTFDSESEITTKQIQEWASLYTDLTNCCKEVTQCFGGLVIFFQLLYSSVGANSHNVDSLTSLVLAPFWQA

>HarmGR119

MIEDKVIKTFVISENIMGVCRNFAALTTSQKIFSIIRIVVEISAYIIIYSLFFLDKCNHVLIEGGHFLSLMMIYHPVNFVCGSMILLCPVYNPCGNKLFIKEFTMVQCEFRHTPFYAQSMKRVKSYIITCITFFSIIVAIVLYTKVLLVFEWSTFGPQSLYIGLITIEIIFEIRQTVESVTIFSYITLLQYHLKTINSCIASVVAQYDPLEARSDSQTNNDHLTVDRVQYWADTYEKISNCSKLLSQCFSTQVNFGLNSILLFIY

>HarmGR120

MSLLNNLIKSENYMCIYRNFMLMKNYQKIIVLIRIFLELCATIIIITHKIFFRDLGNVVSSLLFVPYCYQALVLLKNLVIVVGSVLNSKSFLIVNEKTRVLHNRFRNEPSYSKSVKILNLKCSVISIAFLILVFIVIIIRIYIISFNKDRFNKGRVVIIILFEAWVDVRFMLEHLVIYTVITLIYDFLKCLNNYVYEDLKKYNIDTKDEEVHLDEQFNETADKLTVWTEVYQDILSCTKNTSICFNELVIKYEFI

>HarmGR121

MLTKIETILGVNRNYPSTSKTRKILFEIRIVLEVLYIIGFIYTYRTKIKFKDGQFYLMEIFHLANYLSGPIIMINGILTSQQYKRYLENFIPVHAYYIKESKYAEKMKKIKTIFIIVTSISFAISCAGFLVKYYNRYVQNQEITFLVTIFLLSALLVHYRFMLENGVMFTHMAMLRNLLKCLNDCILDAQVGYSYFVQSGRSDRNEWRPLLSEEKVQLWASQYMSLLNCSKNLSVCFRAQVREPQIQ

>HarmGR122

MSSVKLIKNLLFGENMLAIYRTYDCQNRLMKCFIIIHILILILLNTFIVSLELYYLFDLGEPDQMSVVFCGFSIASYVNTLSSIMSGIYFSSGFLSFMKSITFISESFKNDTIVIKSEKRLRWFSLLILTFPFGLFLIRLQEVLKKFKDLNLMILIPIVVSQTFTRMTLLYQPIVFFIVISTVVIYFKCLNRLISIATDSLKICRLRSGLHGECDLGRDQIEGWVELYRDLANCCEKVSICFGRQFSFSLVLTMSNYILLLYQICYMNTYHIPNDFEFKKIILVIISYIIMTMLPIFAGQLICNQELKCHRVLSRLYNTMLIYSNEAEVKLVKDFIRLMKKYPLDIKLMNKLPAGMYMVPAILSFAVNYTIVMLQFHHVI

>HarmGR123p

MTKQQWRTLMFLIFTMFFNVLKPVEAMFRYEFTHPLFVGYILLSVSFLKITMLFEHFVLFSIIILIVRVVKCLNHMVNAAEESLRNQIFDNECEIVTKQIQEWASLYTDLTNCCKHVTQSFNCLLSFSIMLSISHFVLLTYEIIYTIYRKGIIITDDLLQISFVILADTAVMTMPMIAGQLLLNQGVKLHRLLARLYNVLIIRPEDSEAKLIKDFLRLLKKKPLEVRFGSNYQAGMSLLPVMLMMSVNYLVILLQFNHVV

>HarmGR124

MIKTFLLAENIMCVYRNYVSFSGYKKVLILIRITLEIIWMLITILIFVWQVSEQFSTRNHFLYLMGVYHLTNALCGIALVICGVQYSESYKLFLNNFEVIRHEFQNSPAYTKNIKKLKRNLTILIVFFAFVSIIDFFFKLRANMITWRQNHIKITFLTLLFTLVYIEIRKILVNSIHYANITILRSTLESLTDRVSEIREKLSKPGMSDRSYKLTVAIVDEWAVNYQKVLISSKLLSECAGCQVICTFFLICVLFYLYLCCLKSICFYFGYCLLLFFYPVHNVV

>HarmGR125

MVLYHSSNLLCGSMIILCAICCAGSHKLFINNLKIVQREFWYTPTYIKGMKKLKIYIAVTTTFFSLAVASVLYSKCRNATIWMTPDIILCYIALLVIEIYVEIRQILESIIIFSYISMLQYSLKCLNVDVLGTRKQYNRLGIFLYVELKSSNFLNVQVEEWAAKYQNILVCSKLLSDCFSNQVSWYNSIKITLQTKQLADW

>HarmGR126

MLFTSKFSIAYNCIRPHRFFYYVFSFIGVLTFILYHLQRLFNGNFATYNYLSLAATLNVFFVVIPFPIFYTLNVLQREDNVEIILKIQNALKIINYKRYMIRTYWNWFYIIRHLIAYLIIVCITRNSQIAIYYYTLNYVDVNVIYGVIMIELIRDGVIIWVSEVEHYSKLCLDLNEETYNKTMKKLFHAYISLMEAFDIFKGLFQYSVSFYVISWQICFTRDHLAPVGLHSLSEG

>HarmGR127

MSGAESSVVIRIIIAVRLLCGFYTKVSSNKIVDALVRAYCVIFSMTVFSILCTVFSRVPLIKVTFMITFSLIYACNVILDHCFYGDKFFEFVRKMRHISISQDSRLRHVKLPITMFLTILLFCSRLFSHLKYMLENKYVIHMISMQSLGLAYDLIQLVSVLPRILMFELIWRQMIILRKQVQQDLSSIRRFEAGEELLKNKLKVFSNAYKNLLNSTREIDSASKLLVRNAILIEIAIKFVVIISPALLAEMVNSEIDKMKLCIVKQLLVCHGDSTRDAIEDVMLYLKQNPFKYTIWRLFTLDMTMLYLKQNPFKYTIWRLFTLDMTLILSFIGVFTTYTVALVQFTHFFV

>HarmGR128

MSITKTDIFTKIIIAIRLICGFYYKISTNKVVNALVRAYCVSIATTVIVIIIYEWFISLDRSAVKILVFSSTLYSLNIFVDFCSNRENFMLFVKYIRQPISQDMQLVVNTPITAILLISTLCLRVFSHIKYQYDNGIWFAFISEETLMLVLDILQYVSISQKMMMFELLWRKMAALRNHLERDLSSARRYETREDLLKNKLKACVDIYNNILNSTREIDAQTKFLVIN

>HarmGR129

MIQKNYVDKDLQSMLLPLNLMQNIMFYPKYSIFNNCIVPNSVLSKVVALCSTMAFVLIHLYRSYNLYYNQMIREFVNILYITSYFDIILSCIGFAINFIVSIYQTENNVLFILKFQKVHRFLNDKNLFNRFVFMNWLILVLLFSFVIFIMSSFFLYMEVPLPDFFCGLAAFCFDINIIYALRLIKLLEDKVKLWNFEAQHLLQIYHSNIESHCQRMFEAYFNILECYNLYKYSFQLMVCMYGPESLFLRYFLFHHLRC

>HarmGR130

MLPSLENSLESPENVHTIESISPEASNEETVNNKIDKDLQSVLRPLNLMQAIVICSKYRISDNKIKPHSRLYNWLGFTLVIAFRILSIHKLLTSNYPSNVSRLVAFLYMINIFDFVFNAIGFFLNSYVNIIHRYNNVWLVLKLQHIHRILNINSKNLQMLIIYNWISAISIYIVFIMYMCYISLFFPVFSVFGTIVTFSTISFDINVIHALLLIKLLRQTLRMWILKLLDLKNTDTVSNDESLWTQMFDAYKNILDVYKMIEKLFKLMVSIFL

>HarmGR131

MKFYRKTETKTNVHLVFNNLLDKDVQSIFFPLNLMHYIVFCPKYTIKNNFIIPTSFIVKLISILGTLVFISVTLYRNYYLFFYQESVTISPFMYYSSYYDALFYSFGFSMNCLFGIFKSELIIRSIMTFQNIHRYLNNESNTRRNIILNWTYVIVTFVGYFSIYTYFYSQLSNSYNLTNAFFLVSFDINAVLAIRSLNLLEDKISLWNVSICKNQELENVNDRNYAKKMYQAYVNVLECYETLKTLSRSFVST

>HarmGR132

MRSHTRQTFRQHGIFFPFESILNNQLDKDVQSILFPLNLLQFIVLNPKCHIKNSFINPNNSFNKVILFFGMIIYVSAYIYRVLEITLDVNLRAYGTLSFLYIASYFDFAFYSTGFILNSIINFCKTKDMVNLILMYQDVNRFLKDKSNFRWSVIRSWIYVALIIGFYVFTMLFMSVAPFHIVFNLIILISLDSNIIYTIILLKLLTEKVVVWNSAILKVHKNGCSTSYCTKMFDVYVDIFSCYNLIKDMFQQPVSLLKFFSTTFLIYYYEASKIFRLCYFVFRFYTKL

>HarmGR133

MLLPLNLMQTIALYPKYSISNNVITPNSAISNVLSLCATMAATVTHFYEGFKLCYDADVVFKYIASNIQYFASFFDIFLTCIGFIFYFFICIFHSKNNVLFVLTFQKIHRFLNDEKISKRFICWNWITMAVVFIFDIVVLTYFNLRLHLPLYTFVCCLFAITFDVSIYYALRLMKLLSDTIKVWNMEAQNLVRLRHSNINQPNCQKMFKAYSQILECYNLFERSFQQIVCILMFYFFVLEPSLL

>HarmGR134

MAEKHYVDKDVQSMLLPLNLMQTIALYPKYSIWNNVITPNSAISNVLSLCATMAATIVHIYEVFELCYDADVVFKYIVSSIQYFASFFDIFLTCIGFNFYYFICIFHSKNNVSFVLKFQKVHRFLTDSKRKRFIFWNWITMASLIIFDVAVLIYIHVKLHFPLYNLFCCLMSISFDVSMNYALRLMKLLSDKIEVWNMEAQNLRLLHHSNSDMHCQNMFKAYVQILECYNLFKCSFQQIVCITDVFFCIF

>HarmGR135

MESARQKTTNKTKSCSVSIATIKTIDKDMQRMLLPLNLFHNILLCPKYRIKNNFINPNSFLSIILGLFGLILSIFSFCYRVYKYYRINPKKYVMNVMYVTSYIDFVIFSIGTIINFKINVLETVRNVSLVLKIQDIHRFLPGQNYFKCFTIWNWISIFSGVGFYTYMLIFTVLTFEMHIDGMLFGFTLLFIDVNIIYCIRLIKLINNKVDIWNRRALKMHQMDPIDNEDYCEKMFEAYMNILKCYDFYKDSFQLMVRNNLFHSSARYF

>HarmGR136

MCGKRYEMRNTRKRIICRKIRVHNYKNNQIDQDIKWMLFPLNLMQMITFFPKYSIRSNIIKPNSLILKFVSLTATILFISAFIHRHFTLLSRSNIHSSSLIYTYAATVAFCLGCIINFIYSVIKTRDQIIFVLTIQRIHRFLNNRNVYKHFVIWNWIYVIGLFIFYISAVTYFTIMLNLPIYSTYSSVILICHDVNLICAIRLMRLLQDKAVLWNDKIWFQENENGHHNNVCRRTFKVYVDILKSFNVYRTVFQIPVSKFMLNS

>HarmGR137

MEPKGRIKRIFRRYIDKDIQKMILPLQMMQTICLNPKFSLKNNFIKPNNIANNLLAVVGVIFFVSLLIYRICDMMLDENLRRYQTVNFLYFATCVDSFFYGCGFIMNFILHFVHTMNNVNIILIFQEIHRHINDKASSNMAVFRNYVIVSMVFAFQTAASIYVYIVYMHPPWYVVCYVLVLISLDSNIAYSVCFMKLIADKLVLWNAKLLWSLQHGSHVMRCKKMARAYVQILNCFDVYKNIFELPVSIR

>HarmGR138

MAINIEDISTQNNVVEKEVLSLFKPFRMMHALFVTAKYKIKDDVISANTLLYTCMSGFTSIVILVFYFFSIFQTAFVFKWEGLNLAKQVCNIFIYAIYLMGSMMNFCSDIINKDFNVLLVYKIQSICETLKIKGKSLKNFIMINWIYVITLNVYHMWWIVFFSYAFSSSYLYYEVVTNYFYIIFDMNVLYGMRVMKIIRQPLQIWLEEVRNLNSVIDEDYEYFWNKMFKIYEETLETYQIFAKIFRSVVSKVNWRLLIILAYEICLKLILRNYLSCSLIRTSTIYFRCSL

>HarmGR139

MVTVEVIPVNKNKQIIEKDVQSLFKPFNIMFALFFCSKYRIRNDVIHTNSLFYKVVSGICCLAIFIGYCISVFIKIFTIHLEGINYSKFCYNITVCALFFSGYTLIYYTHVIESNRNVLLMYKIQNIYKIVKTRGVFVKNFIKYNWIGVAVVALYQLLWILFFTIAFASNYEYYEVIANYVYVIFDLTGLYCVRIMRIIREPLRLWLDDVKNVQHVDHEGKASFWNKMLRIYLETLDAYQVAARTIQPGVSLIFNY

>HarmGR140

MKLKVVAMEASIVAKNNIIDEDLQSVLKPLNFMQAVFFLSKYSIRNNLIKPNSLIYDLISVTCLLMFRIVSIYRIIIFSFASKWTPLLQFLYVSQILDSIFYSVGFLLNNYINIVYSKINIGLVLKLQFVHKVLNINRHKLRPLIIYNWIFAISVYSYFIIFNLYMWLKFPNPSYYALILVFSALAFDINIVYALLLIKLLTQMLRIWLVEIQELTNVGMSGSDESYWNKMFDVYKNILEAYKTVEELFKLLVRFYFCE

>HarmGR141

MRSTSRKIWNRIRIILSSEEFSNNKLDKDVQSIFFPLNLMQLVVLGQKCRIKNNRINPNHCFNKVILFCGMVTYLMTYMNRFLEIMLDENFRTYVKNPFLFLSTYFDHFFYMSGFILNFIISVTKTKDVVNLILTYQKVNRILKDESSFKGTVTRSWIYVGAIFAFYLYTLFFSLLASFNVLFNTVVVITLDANLIYTMVVIKLLTEKVKLWNARIITDSENDCNNKMFDVYVDLLKCYDLLKNVFQQSVSQATPF

>HarmGR142

MKIIIINNYKKINFVDEDIRSMLLPLNLAMLCPKYSIKGNLIAPNTFRNNCVSIVITLVLISAMCYRTYGLSFYQDGFSNIVYYYSYYDVCYYSFGYIMNYIISVYQTEQNISLVLTLQKLHRLFNDAAAFKRFIIFNWIFVITALVTHLLLVTSACLDMLYHSKVNLIGYLLVLFDIYIIYCFRLMKFMEDKVHLWKSKLESSEEFDVCKFCEIMFESYVDILKCYDMIKDCFQRFVSMILFFNVSNIENSCWSA

>HarmGR143

MAVTIEEIQNIIDKDLQSLLRPLNLMYILFGCAKYKIHDNKISPNSVIYNTISSITAIFIFCISFYFMIGTFSLNFNGYIYINHLGKIYTYILLIVGCLSDLYTNIFQKSNYISFVMNIQNIYRSLNISGIFRSYIFPNWVSVIALNCFHFTWMFYTFYAFQSLDHSFVFASYYCIVFDMNIVYAIRIMRLINKSLKYWLEDVEMSGRFVTESYWNKMFETYIEILKTYQIIESTFQRTVCLSV

>HarmGR144

MVKIDSKPSFEKSELSSNNVIEKDVQALLKPFNVIFALFISSKYRIQNDVIYQNTLLYKILSGISYTFLIAGYFYSIFRTAFIYKWEGINFTKQWCNVFIYAIYFLCCVINYHTNIVCSKINVVLVFKIQNISEILKVKGISLNDFIKFNWVYFTILNVYHVFWIVFFTIAFSDTYEYYEFLTNYVYILFDMSVLHGTRFLKVLRQPLKLWIREMRNSDSVLDEDNEYFWNNMFRIYEEILDTYQILTKTIQPVVSY

>HarmGR145

MVLSRRNSMSLIETNQGDVEILSNNFIDKKLERLFFPLNLMQNLVLNPKYIIKQNRIKPNDVFNKFKIFLSMVIFLAVFAYRLCEVIFDENLRRYGSVKFLYFEIYSECFVYCTRSVVNCIVNLVQSKNFVAFVLTYQEIHRILTYEHMIKFYIIRNWVYFSIVFGYYIIVLVLIPLIFERWAFHFDINVFTYIILDANLIYTIALLKHLNDKVKQWNIEVVRSPHRICSERMFQVYVQIFECYEIYKNVVQENVS

>HarmGR146

MELITNNYLDRGFQNMLLPFRCLNHLVFISRFSIEYNCIRPHSRSYYIISFMGVLCYIIFHSLKFFDANLTAIPNQFIQFFLKVNIIMLLIPYAGFFILNVLHRNKHVQILLKMQKAFRIINYKRYKLAILWNWFGVFRHIGGFIITTAYIRLLSVAEYFYTLIFFDVHITYAISLITLIRDGVITWIAELERHSQNLEVDKDKHDERMKKLFQAYINLMEAYEIFKKLFQVAVRILSF

>HarmGR147

MFPCFKHEGQSDPSSEDVVHEDLQSVLKPLNLMQAPFFLSKYTIRNNCIKPNSIIYNLMAVISMLIFRIVNVYKIVVFPFVTKVNSSVTLFLYVSQILDTVFYTVGFVLNNYLNIVYSRINIGLVLKLQFVHRVLNINRRNLKSLIIYNWIFVVSLYSYFIFIGIFSWITYPFITLYSYILVVSALSFDMNIVYALRLIKLLTQLLRFWLMEIQELRNLGVCRSDESYWMKMFDVFKNIVEAYKTIQDLFSLTV

>HarmGR148

MPKKNRIVEVIVRKDGIKNPLIKDIKRIFLPFNLALNLFLNSKYEIRNNNIYPNGPKYNIFASFFLILMNALCVYRMFTFDVADNSSIEEDLTKAILGFLGTSFYFVTLIGFTITFISNTLHRENVVLLILMIQTIYRSIDISKSINSYIIRNWICLVIVIISDFTERLMYHVTCHYHVLFEQAFDIITDIMPLVLDINIMLFNRILVLLRIYLEEWIKIVETTNDDDEEQWVRFYKIYTNILNAFNLNAKVFEWLVRLIPYLMQNSIIFLSEYLYMS

>HarmGR149

MIDNTNSNYIDKNLQTMLFPLNLMQNMMFFPKYSIYNNNIRSNSLLSNFVSLCTTIAVISLHLYRSYKLYSDNIIREFINILYITSYFDIILTCIGFTINFIVSVYQSKNNVLFILKLQKVHTFLNGEQQFKRFMYRNWMFLVFEFLYFTFGLCFFCVKLNLPKYDYFCSLTALCFDVNLVYAIRMIKLLSDKVELWNIEAQKLLQLNYVDIESHCQKMFDAYVHILECYDLFQCSYQQLVRIFFSLSYLFNKVFFVLKIFTIFYRSCSTAFNSFFTSSFISKLRLTF

>HarmGR150

MNTVDKDVQSMLLPLNLMQYLTFCPKYRIKDNFIIPNSRVSYFISAIASLIFMFILETFYYQILKSPDFDEEPAYLIACTTYDTLFYGFGYTLNIMDSVIRSKKNIQFILTFQRVHRLVNIEKCFKNFVVWNWIIITLFLTLQTLLITVFCLLSDFFDATVGYFYVLAIFDLSIVYAMRVLKLLENTTVLWIQVLNSHQFGNLYDCKKLFQAYVDILQCYDMFKSCFQHFVSFYQAKCFFHVMF

>HarmGR151p

MVFRLLCGFYFKISSNKIITVLVRAYCLFIGATIVLGTIVYISQLEAILISILNVTNIINVVTDCFFYGENFTAFLQKITKLDISGDLEGQIDTPVTMFILLFTLFVRLCNHIRYLIIIRVYFSVEFCFGIGSTALCFYHTKNNDVRATVAPDEKTP

>HarmGR152

MVLTFKTVCDKRNKKIKVKPRINMPTEAKLNNEIDKDLQSVLKPLNLIQGLFIMAKYKISDNRIQKDTLLYNLLSIICLIIYRLVNFYKITISSLNRDWEGTRFFIYMSNINDTIFYTFGFVLNNCINIFYSDSNILLVLKLQQVHRILKINSKHLNDLISFYWRFIISFFISHLLFEMYFIFQFPVYTMYGVLLSFAILTTDINIIYAYFLMKLLNKTLRVWIEEIQKLRNFVTFSINDSYWIEMFNAFEYILKVYNFIRKVFKLMVSYCFIIIMTETFIIIVLYSFFTFTIVEIMSFPCHF

>HarmGR153

MTPDTSVSLRNDKINHSFAIHNFVDKDVQTMLWPLNLIENILLCPKYCIKNNIIKFNSLTCILVSVIGFIICESLRLYRIYNLHFDYFTRNFNNIKYIMAYVDFVLFSFGFFIVYFVNIFHMKYNVLFVLKFQNIHRFLNEKKYFMRYIIFYRISVVIIPIFFTGVILYSFLRHSVTVMDCICAISLICFDTNYVYAIRMMKLLKSKVDLWNIQIGQLQKLDQDEKVICCNKMLEAYKNILDCLDLYKTVFQPLVSLNFFCILYHY

>HarmGR154

MKMKIICNTIKKYFSKPVMKKLIDKDLQSMLLPINLMQNILLSPQYRIKDNLIKTNTLTAILVSFCGVMISIFAFLLRICLTSEAIKQYYSSLYIVSKIELVLYSTGFIINYISVLRSNKNVLFILKVQDIHRFVNDGIYLKRLIVCNWISVILIFSFDFTIIIYAWVKLELRFYNLICGVSVICFDINFIYAIIFLKLLRNQAELWNIRLKNFSGQSNGESVCRSMFEAYDNILKCYEMYKDYFQQNVCNFNHQRVIFVY

>HarmGR155

MSVVEDGISNSNDRNVIGKDAQAWLKPWNLMDALFICSKFKIKDNVISSNSLFYNIMSITSCLVLVLIYFYCIFKDCFHIAWEGLLLAKYVHYCFEYLVYVIGVIIAYFFNIKNRHSNVVFALKIQRICEIFKIHGKSLKSLIVLNWVLVIVLNSYQVFWVFFFYYAFSRYGFPIEELVPNYFNIQFDVNAVHTSRIMKLMWQTLRTWLEGLQNVYIVEDEDVEHYWRKIIAVYKEIIEAYDIFRKSFQVLVRE

>HarmGR156

MSNTLRSKVFQNHLHKDVQKMFYPFYYFFLLLLSPKYCIKDNYITPNSLKRNLVSFLGAFYVFVTSIVYACEEGYKDYYNESNVHSLAMMSFIYSLDISSFALGIMLVFVQNIIYSRKNILIILMFERIRQSIDISKSIRSMVIWNWVFGTLYFSIHAIVFIELHVLSQVNFLMQIGGFICNYMYAIFDVNSVYGLRIMKILTTYLNRWTEMVLKLNEGEENLISCVKLFDIYTNILKAFELFKDVFQVLVRSVDLIGSNYSACLFELERIIYFTGIIYNNQRVYS

>HarmGR157

MLKPCIFLRKTRSYPVEVLLNNRLDKEVESIFYPFHVILTLLCASKYCIRDHFITPNEYKFYTVNFISLSYVVASFAYQMYNNQLVHIHRSDNNIVVSFLSVFLPISRCICHILYFVLNIMHCQNNVFIIVLINIIYKSLNSFQKVRCHIINSWILLALILLIHAWLTITYIVIYEIYFDVIHHVSEVLLYIFDIDFVYNVRVLLMLTNYLNSWIENIKLFDDGQEYDKIHYIKMFQTYLNILRAYDVYKTVSQVLVRWQKSFTQIKCMSNLCFILGLPPSLTKCS

>HarmGR159

MCSVLNMYKYIRNKLRCAREAVLIDGVSPAAYKEVLFDNIIEEDLQSLLKPFNLMTALWIGKKYTIRDNFITFNSNVYNYIGITLCVAFLIARVSYRLIVEGIDLDSFAFSLLRTNAMLDLIIFLFGILLTYSTNIIHSYHNILLVLNVQNALRILKINRKDLRRLTVYNWTTIACIWAFFFVPHVFYSMGTKRFDIFENLGILFKISFDVNMMYIIFLVNLLEKILHEWIRNFEKSMFKTSDELYWKKMLYLYLNVQESYQIVETVFRTHVSLQYHYSKFLHTESV

>HarmGR160

MTPYSREVLLNNRLDKDVQRILFPFNFFLTMFLSSKYCIRDNYITPSKRKYYVFGLFGICIITAANVHQMYGQIANMDLNKRGLLILIFLHVTQIFNFALSIVLNIIDCHKNVLLIVIIQAIHRSFDFSKSIRNLVFYSWMILLIGLCINVYTIAYGYAILQSWHILSFIHDVLMVVLDIDLIYKIRLLILLTTYLNEWIKNICLKKDDWQQDQANCVNLFATYQNILKAYDVSNELSEIIVSYEVYL

>HarmGR161

MRSFKNIVNIYPKIINNKVDNDVQEMLYPLDFMQCLIFISKYHIRNNLIAPIGVITTFISMIATMAFVVVHIYQTLFATSETNSIASITTENITRYFSCVFYCSVFTINFIMCVIQTNKSIKFVLTYQKVHRFLKNSGSYSYNNNYIIWNWVFVITAVIWHTSTIVYCVVSIGYNFFVATCYMYPIMLFDINMVYAMRIIKLLENEINIWNDVIKSRLHTQDENYCRDLLNIYVEILECYEIFKDCFQQSVSISTNRLD

>HarmGR162

MKLKICRYCSVSTKKTSLTMNSVLDKDVQSMLLPLNLMQYLTFCPKYRTKDNFIISTSRVSNVISVIGTLVLILSLELYYYKIVFIDNISEKQYYRFMYSLIFDSIYLSFGFILNFMDGILGSKNNINFVLRLQGVHRFLTKGNNDKKRFKYFIICSWIIVISFVSVCFFSVPLTFIHTEFYIHIIYYLIILIFDINLIYATRIIKLLEHQVATWIQVFSCNELDYLCEETLLKKLFRAYVDILQCYDIYKLCFQHFVSIGRKSDYIF

>HarmGR163

MFQSLNNVKHKQKIPRNILNFPPEVCLNNYLEKEIQNIFRPFNYAFILLLSSKYTMQDNYITPNGKLRTFLSCMSASYVSGVGFYYMISNKYLEYNSSVYSITVVTIVVQLIIIGYCFGVILIMVNNFVFSQKNILLIVTIQTIGKNINLSKVVKNFIVGNWIAILIPSAIYAGIQGSFYIFYYIIDYTLVLATLCTIAFLAFELELVYALRVIVLLRKYLQGWVQMVSKLNYDQDDGYDCVKLFKIYQNILQAFELYKAVSQFLVIGLHLLSSQKSFLKRTLIL

>HarmGR164

MHILFKNILDKDVQCMLWPLNLMQYMMLCPKYQIKNNLITPNSLISNIISIIATVGFISSSFYRTYEIIYYSVLKSSFFMSFVLYYDCIYYVVGFIMNCAMGILQTKNMVKFVLIFQKIHRFLNDGSLFTRYVIMNWIYFIAALGFFFIILMLFVMLFENWIFIIYGYELIFFDLNVVYLIRIIKLLEDKVLLYNKYLLNCQNLTHDESYRQKMVQVYVDLLECYSILKKSFQQFVSNAFIFCFSD

>HarmGR165

MNTVNKNVQSMLLPLNLMQYLKFCPKYCIKNNFITPNSNVSKLISAIATLALILFLELCYYKLVFHDHYDKEKHYYLLASYTFDSIYFSFGLITNCLDGIMCSKNNIQFVLIVQRIHTFLNIKRNFNHFTAYNWLTVISYISLYFTLITIYCIHLNLASSTIFYYMFIIFYFNLIYAIRIIKLLENQVVLWIQGLNCTQLENTYDKNCYKNLFQAYVDILQCYDIFKSCFQHFVSILICKFSTDFNICIIIYFFGLQYLLYVTFQILYYISEVFIYSLINAEEAIILLKIGWVYLHQVRSFLESDMKVFIYNCICRNVKSLSYFCYRPKHGSYHYS

>HarmGR166

MHNIQILKAVKNNNNIVDKDIQSMLLPLNLMHYIMCCPRYHIKNNLIIPNGLISHCVSIIGTIVFIALLCYRTYVLSSEYTAMFDVLVYYYSYYDIVYYTFGLTMGCTLSIIQTKKNVEFVLIFQKVHRFLNDETSFKNLIVFNWIFFVAAIVCHFFIVSGFFSLLTYYSKFVWTAYLLVFLDFYIINVIRAIKLIEDKARLWSLNLLNKNIENMDVQNYCKRMFESYFNILKCYDIIKVCFQQFVSMIITWLYDNLIVAEL

>HarmGR167

MFNSHVAVTSCYPVEVLLNNHLDKEVQAIFYPFNFLLTISMSSKYCIRDNYITPTQRKFHILRFVCTVALLVIYDSVTFKVFAGENIIFYIARFLSVIRNLTFLQNVAVSILCNQDNVLLVVLIQMIHRSIDLSNKIRSFIAWNWIIIATISAFNIMVATAFSSGKDGFNFIGCFTDVIYTSFDVDFVYSIRVLNLLNKYLYEWIKSVRIMNEGKENDKMNCTKLLKTYENILRAYDVYKKVTQYLVSILRKGYIILCFSITKQKTM

>HarmGR168

MHNIFKIFQKKRNQTIILNPVHHDTRLDNEVLRIIYPFNFAFFLLLSSKYSIQDDRIMPIGMMRKCLSLFNVFYAGALSLFFIYFYIVTNDFSKSSIIMSIIHVSGTVTFSMGLTLIIVVNIVFENYNIQLIKMIQFINRGIDFSRSVKSFIIYNWVFVIFVFSIDLFTYIFFMVTYYMDVLGIVTLWARLMFISYDINRVYAIRIITLLRKYLDEWNKNVSQVNNEDGTRFMKLLEVYENILESFKLYKTIFQELVSIFNELNVYLL

>HarmGR169

MRSNCPPAVLLNNHLDEEVQRILQPFHTLLTVFLSSKYRIRDNHINPNGFIFEFSGFSGLCFAFGATVYRLLKNENSGFDSNVMTNIIHYFLPAMRLLGFVTNFVLTIIHRYNNVILVLHIQRIYRSIDFSKSVDSYVLGNRITVAIILVTNGVIFTVFITMYNGFDVLNVLFDIYFVTLDVDFIYAIRILILLVRFLEEWIKSNKLIEEQAIDGEYSSKLRESHRYILLAYEMYKTTTQLMVSQEGPQKVDR

>HarmGR170

MCSYFKEYFESCQELIIIKDISPEADFGNNIVDEDLQSILRPLNLMQSLFLSAKYCIRDKNITSTTRFYTFLRIIFVLVHRCFQAYQIIVWNTNMFNENSTSPNYYSMLYLCIASTVGFFIYFIGDVISIISNIVFRRSNILLVLKIQHVFSFLRLNRNEIRGFIVSSWVLVIVSNVMSLSFVVYYVFTFAEVHFITMFTAYASISFEINVLYAFILLKLTENMLREWIKKFEATRNIDDSEKLFQVFGIYWDTLEIYMIIEKTFQHMVWFDSTC

>HarmGR171

MKHNIIRYTLCITDMYPSLTMYLKQFRNFAKVENISAVTSEDRFQNNKVEEDLQSIFKPFNFMMNLFLCAKYSVRGKCFTPNTRFYNWFRLICVIVNRCFNLRQFIIWHYTIKKTHFVFSYYYGHLCINIGSAIIYILYLTGDLIISVSTITQSDYNIFLVIKMQEVLKSLKINGSEIKGFLSFSWWSVIISNILSIGYIILYCFTLADMVSIVDVISAYASISYEIHVLYALLLLKLTNKMLTVWIKEFRNSRNLGDSTNEEYINRMFFIYWDVQDIYMTIEKTFHHTVRNSIYCFIWIVAVVQYIYIFQILFYIIFTISISLWEIFSSLVFKTAGHQSVSIKVTWLILSGIL

>HarmGR172

MTRRTRIQQNNNNIFQVEMLLNNYVDEDIQSMLLPLNMLHFIRLCPKFIIRDNFITPNSSKFNSVFFIATIVWLFALFYDIHTEFWEELVNFNITDSVSFSVFFSSGLIVNLSLSVFRTRDYVSFVLKFQNVHRFLNNESDFKVFTFINWFIVILFVIIYGGFVIILILITDTLTIQLFCAFVLLSFDADIIYIIRFMKLLKDKFNLWNEQALQVRNMRDGNKEEYCQKLYQAYIDIMECYRLIKTFSRLVVSKKQASLPVSLESFLVNYFIAKTSCYCSISSTSRNYYPQ

>HarmGR173

MWSCFKQYWLPHHRSVIIENTSSFSPDDIIEYNRIEEDLQAILRPLNFMENIFLCAKYSIRRQYITSNSRIYNFFRVFCIILNRCFHTNQIIDWNITVWNERNTTLHFYSTLCVSISGFIGYMLYLTGDSVSTVSNIVLSRYNILLALKIQCALSSLRINRSQVTGIIICSWCCVIISNIYSISWVIFYCCYYGNINIVTIVTAYASISYEISVAYAFILIKLTDKLLQQWINECRALSLEDSENVENVDKLFIVYCDIQEIYMIIEKTFQHLVSFEPS

>HarmGR174

MFSYLKEYFKLYKMSTVENISRVPSDNICENNRIEEDLQLILRPLNFMQGLFFCAKYSIRGKSITCTTRGYHLLRVICVIVAHGYNAYLFIVNSIAFWNNPIASSFFYSSLCLWVSSFIAYILYMIGDSLNSIVNISMSHLNIVLVLKIQHVLSFLRLRRSDVKGSIICSWACVIIANILSFGWIAYFCATAPEINYIPIITSYASITYEINVIYAFTLLNLTTKMLNVCINEFRTSSCLKASENVKYLHELFHTYCNILEIYMIIEKTFQHMVSI

>HarmGR175

MWDSVRNAQIFIPSSQPVALSKNRLNKEVYRIIYPFHLIFLVLCSSKYTVKYNFILKDGVLRKIVSFLSVCFVVIVSSCYMFLEKYTAYINNVQSIKIIFFIFHGVLILYCIANIMLFSMNIALSKQNIELILKIQLVHTNIDFSKSSKNFIIWNWIYLIIFLVIDLAISSSYYVTYYEQDVVDALGYLCNYLFAVFDMNVIYACRLITLLRKYLNKWSKVILKLSDGVNNRNCGQLFEIYDNIIRAFQLYKTVFQVVVSSLLTIAVAIDSFVHTFKQISCWCHVPLQILSTTVNIFSRNLGFIESRLQIITDTAESVNEVIFVLLSLKFLLSLFYL

>HarmGR176

MTTVRKIVSIKPIYNFTIDKDIQSMLLPLNLTQYMMFCHKYRIKNNLITPNGLRTKCITIIGTIIFIFSIAYRTFSLSFNQNSAAFSPLIYYYSYYDTIYYGFGLILSCVLSIRNTKKHVRFILIFQKVHRFLNDKTVFKQSVVFNWLFVITCLVIHFTTVISVALMLIYYIKYVWNGFVLVVFDLNVVHTVRFIKLLEDKVEVWRTRLLNSPDLEITDLPSYSKGMFQAFFFFF

>HarmGR177

METLPLSNAPKSSFSSNVIDKDVQAIFYPLNVMSVLLLHPKYVIKNNKITPLSNVIKIFSACVTTLYLCQHAHKFFSVVLDDNIRRIQPVSYLYYATGSDLLFLTWGFIMNCAGNIIYTKKYVAFILKFQESHRFLGSACFKRFIIVNWLSIISMFGYFISSCIYTYFTFFHPPWGTIFHMMVLANLDADAVYACRLILLISEQFIQWNERALLLKENGEDKDYCRKMFETYGQILKCYKIYRNTMQFMVSRILGFLMIISCL

>HarmGR178

MSIKMKPTAHPKEFTCTYIDRDVQKMYQPLNLMQQLTLNPKYQIKAGFIKPNNVKSILLSFCGLISYVGVSIYRVWELATDENMQRYTPIKFLNFATLIDASFNSTGYIMNFILPIIEVKQNITFILTFQEIHRFIHKTDNCKIIVNNWISVITYFSFYIFACIYIYVYYMPNWYVMYYVFILCTRDTNPIYAIGVIKLLTEKVFLWNSELLMSSKAGHREMRCDRMFRAYGHILSCYDVYKNIFQIPVSIIFGKLRNYNCVYIKSSFLFLN

>HarmGR180

MQVSDIIKPGDFNNKSYNSMIPIIRILKVFAINGNVAPNKTSMLIKSICACSIFGCLSSYCLYYKTKYVYNRLDISIRVTDMTQMICDFFQYTVDLFFVYKFGRSLYIEYFRQFEIIDVCLETSCYAEMKRRLLKTMTFFLVIWFISSFTDLGAWVITYGWMIPVVHSLSYLYLLIKILATLDLIANIIQVEVRLRIINNFIKNCYNCASACPVGILADCIRNKNWLHGEDGSPDQSLKARSIDSHEIKRLSKCYLMLTEQVMFINKMYGFRILLNTTSLLFDMVKILNLAIRIIVGSQRTLYNSAGYNFLPGVSGFVRFLTCAAILITLVNRCEQAYRQRERILNVIDHLLINKNPDLTLRSAIQDLQSLLQDRPICFNMAGFFTLNFSLLVSIASVVVTYTIILLQSVN

>HarmGR181

MAQTVMNFVVGVSQTHKSIRFVLTFQKVHRFLKNDPHFDNLIIWNWIVVIIAATFYNSAFVYFTNYLGLPMYFIYVSVILSAFDFNIVYAYRLMTCLTHKLEVWNIKVLSSGETNCDIFSKNMFQAYLDILECYDLVEACFQHYFFFVFQILFYIGEVFIHYLDLMSIAGMAIFSTVIWLMKNLAWQIMLSQQCEKFYSTVQSAQDNCMFVLKSNCTESVQRLCKNVRRLHRSRFSKLRVCALFRADAALQLSLMALLTDYIVVVLQFAFL

>HarmGR182

MSLMCTVATALRSSLNTKKALDIGRLQNINVMPFPSNVVDKDVQSILLPLNLLQLLTFCPKYRIKNNVVYPNSLIAISIIVIATSIFVLSFVYRSYYLLSANVLPWFSDFSVYFDVIFYSIGFIMNCFFIIVQSKQNVQFVLIFQSLHSFLKETNLKNLVIWNWVFVILVLLVFHIIFIYLLVILKLPFHFLYYSILLNSLDFHIVYASRLMKLLEHKLILWNIQVLSCQGNIDESYGKRLFQAYIDMLQCYELVKVFFQQFVKVLVHSLFYIKASLDLFSMTVKLGQMHRIAMAALSISSIVIWLVKNLLWQIQLTVQCERFYSVILHSQDTCAIVLNSNGSEAEKRLCKNVRRVTRARFSKLRVCALFYVDASLQLSLMALLTDYIVVLLQFAFLDP

>HarmGR183

MSINLVDKDVQSMLLPLNLMQYIAFCPKYCIKNNFISINSLFSNFISFCGLLIFLSSFLYRNTLIAQSLGHSFTVFMYITAYFDFVYYCCGYVMNFIVGIMQTKNSVNFVLTFQNIHRFLNNETYSRNFKVLNWIIVILTVVGQTTIFAYFNITVGLSHYFIYISFIITVFDFNIIYATRLLGILENKLLLWSNNVLDLREIGEIYDENYCRNMYLAYVDILKCYELHKVCFQEYICFYITETFLHSLICIQVSIEMCKMAASRGNISTIGTAILSTMSVLLWILKDLFWQLLFCRQCEKFYSSMENVPDYCTLILKTSCSESVRRLCKNVRRVHRARFSKLRVCSLFDAGAALQLSLMVLLADYTIVLLQFAFL

>HarmGR184

MEIFVNSRQINSNNIVDSDVQSMLLPLNLLQNVFFCPKYRIKNNYITPTNLMSNLISSIATLVFIIMYAYRNYLIGLFKTSQFSTAWKYSSYFNGFCYSLGFIMNLVIGIIQSQNSVQFVLTFQNVHRFLKNENGFRSFIIWNWVAVCLTLVYYVFFFIYQYTRGTIGKIHACVGFLLSSFDFNVVYATRLLRLLEYQLVLWNNRFFKLRETSDIRDKDIIQKLFNAYANILECYDIIKISFQHYVSFTLQLIIDLLKYAANNDCQKTVIIAIRVSIAVLLWLIKNLMWQMTFSHQCERLYLANERTLDHCAFILTSYCSGMEKRLCKNVLRMSRVRFSKLRVCGLFYAGAALQLSLIALLADYTIVLLQLAFL

>HarmGR186

MWKKSIHAFKILPNNRLEKEVQRIVTPFNVILTAVCSPKFRIRNGYITPSSKKIHILLFFGITACNVWSCYGITKSQNEYTTTSIVSYFFSLTIFFYYIDFILFTSCNVLHSRRQVSLILKIQELYRNIDITKKVKNYIIWTWIWFLATFSVFLVNFLSFLMNLDRIFFIHLSTYFANLQFDLNFIYSVRIMTLLVIYLKEWTKSVVDLNEEEQNKEYFVKKFKTYQNILRAFKIFTLCFKDIVSTSSYI

>HarmGR187

MADSSRRLFELVFRNKLDEDTLMIIKPFNIFLRIFFSSKFKIRNGYITPRDKTYYILPFIFVSLFKVWTVYYVYIYNSSILNNTFRHIYFWHIFISYCIYYSLLVYCNIVNSQNNVVLILRIQEIFRSIHLKNGIRSYVIWNWITFVVLASLECFCTTIYARTMNLLSSLNSFDILLSICYDFNVACSIRLIKSLTLNLVEWSNTDK

>HarmGR188

MWYSHRPSQLLLFTNKLDKDVQRILKPFNIILTIFFSSKFKIRNGYITPCDKKLHIILFICVIFLNAWSVYEMRVYISGKASIIINSQIIFSFLLIICFFTYFILIFSNIAYCQSNILLIYTIQDIHRAMRNSSSFKNYITWNWITILICICFDILIMSSYCMLLSKIHIFSVSTLYLNMASEINVFYCIRVLAFLIMSLEEWIENVLVVKSDDYCEEYCGKLFKVYQDIIKAHKLLQNCFRLLVRSLIFILIKIF

>HarmGR189

MFQSKRSVILLTTNKLDEDILRIVRPFNVVLTAIASSKFKIKNRHITPCVKTFHLLICFSIIALKLWSSFMFVVGRGDFKHKKIVDNFFCLTVFFYCVNYTLLTYNNIMHSCHNISLFMKIQDINRNINISVQSYVVWTWISFLITLILFISDISWFLLKSGIVGTVHVSENILVFQFDINFVYGIRLVTLLVMYLKEWARSIIIMTEERPNEYFVRKLETYVNILEAFKLFTICFKAMVSWFYKLDEMLS

>HarmGR190

MKVTVVHPFVLSPSNKLDKDILRIVKPFNIILTAVCSSKYKIRHGYITPCGLNYYILTYAGIGVFLVWSTYNMISINIDELNQRADLAAYLYCTMFLFYISYGQFIIYNIIHRQDHISLILKIQEIYRSVDISKCVQNVVLWNWLSFFLMFCCMIPNISLLYLSQNFVSYTHITNHISNIMFDFNFIYGIRIIALLVVFLKKWSESILDKEFNLEKKLKAYQNILEAFQLFAICFRAIVSSS

>HarmGR191

MEGKTREKKFSCNYVDKDVRTIFRPLKIMQTCSFNPKYQFKNNFIYPANTISDVIAYFGVITFLTIMIWRITDILFDENLRRYQTLNFLYFASYADSLFYSFGYIMNLILHFLHSKDSVKMILIFQEIHRFIRKSASLKNIVCRNWVALALMGGFQSIVVIYLYIVYMHPPWYIIFYVFYLLSPDYNTVYAICFMKLLLDKAVLWNVNLLLSLQGQRKVLCRQMTRTYGQILDCFDVYKNVFELPVSTYKIFIC

>HarmGR192

MNMNQEFAPVFTVDKDVQTMLMPLNLMQNVFFNKKYRIKNNTILPNNLTSNILSFVASLTCTLIFLYRVYLMSALEDSRFTSILYYSSFFDCLYYCVGYVINFVSGVIFTKKSIEFVFIFQKIHRFISNETDFRLFVIWNWVSVSVAASGYVLICIFFVTNADLSVFNGFPCLFLCIFDFNIVYAMRLIQLLRSKLVLWNLSLETTSI

>HarmGR194p

MSIIKTDIFTKMIIGLQLICGFYCKISTNKVVNALVRAYCVSIAATNIVLILCELFITIYRSGIGIVVFVATLYFLHLIIDLCSNCENFLKFVNNIRQPISQDLQSDVSTPITAIVL

>HarmGR195

MEIKSSSFFKSFQKCMGPLYFYKVLILLQVLLGRYFSLSKSKLTRFFTKLYCVFMYIHMIYKWNDVVLVSHKFVLPPFIMSEYTGYFVISIILSEDYFFNFCDNLLTNDRVMGFKNIPHVPPNVIGFMLITVISRVAFVLTRHFTVSLPSVHLIYVTVLLISLDLSHIYTCVIFCMIQLRMKVLRCFLENIHIPINIVSGNEVEMSIKNVRKSLYYYNNLLDSMAAIDKHTQCMVSKLYLHQ

>HarmGR196

MFSYLKEYFKLYKMSTVENISRVPSDNICENNRIEEDLQLILRPLNFMQGLFFCAKYSIRGKSITCTTRGYHLLRVICVIVAHGYNAYLFIVNSIAFWNNPIASSFFYSSLCLWVSSFIAYILYMIGDSLNSIVNISMSHLNIVLVLKIQHVLSFLRLRRSDVKGSIICSWACVIIANILSFGWIAYFCATAPEINYIPIITSYASITYEINVIYAFTLLNLTTKMLNVCINEFRTSSCLKASENVKYLHELFHTYCNILEIYMIIEKTFQHMVSI

>HarmGR197

MDNSVYKLLSLRLLFGHYFKLSSSKWICYIAKIFCFSMLIVNFAINCILLLDFTSFDISQISLWMLLWIMLVESSSSILISLYTDETYLLKFSAKIKNYVSSPTPCRATYVMASFIIPLNFSSVLIAYLYEFGVAVNIFYNISYTTCYCSYLTSLYITEMYAKAINNLTSAIVNRLKDINISDEEKRVCIENFLDNYLKLLKIYNATMTVSRINVSVCKSFE

>BmorGR1

MNRHDHRFSIYNPKRNEAMWKRELFVNNEGKDIKDFQIKDIYGPEITDKDGALLDKHDSFYLNTKSLLVLFQIMGVMPIMRVPKSAQTTRRTTYNWISKATLWAYLVWGLECIIVVKVGQERLANFQIGSNKRFDEVIYNIIFLSILIPHFLLPIASWRHGPQVAIFKNMWTHYQLKYLKITGKPIVFPNLYILTWGLCIFSWVLSFAVVLSQHYLQDDFELWHSFAYYHIIAMLDGFCSLWYINCNAFGTASRGLAINLHKALEAEHPALKLAQYRHLWVDLSHMMQQLGRAYSNMYGIYCMVIFFTTTISLYGALSEILEHGLSYKEMGLFVIVAYCMTLLFIICNEAYHASRKVGHEFQDRLLNVNLGAIDRSTQREVEMFLVAIAKNPPIMNLDGFTNINRELFTANISFMSTYLIVLMQFKLTLLRQGARKTVTAIVRAIFNTTITDNG

AGGSDEDQE

>BmorGR2

MIPDHLFEEGINNTFLDYDMRHVQRNRNIQEKTQKDYEQEQRDLLSSQDGDTCEIHDQFYRDHKLLLVLFRALAVMPITRSRPGTITFSWKSTATIYAVCFYIAATAVVLIVGYERIQILQSIKRFDDYIYAILFIVFLVPHFWIPFVGWGVAHQVAIYKTNWGKFQVRYYRVTGENLKFPNLKTLIVIISVGCLLLAVCFLLSLCALLDGFLLKHTSAYYHIITMINMNCALWYINCKAIKIASQSLSECFQRVAAGVQDTLLSIDVLAVDRPTQKEIDHFIQAIEMNPAFVSLKGYAHVNRELLTSVRFTTIIEADLLMIY

>BmorGR3

MSFEIKNNFFRTSVPIPNGFPVQTEAKSKNKPIFLDVSPAPTPKVNSPNAIIPMKNNLIDPFINKDIIYENIKPVFMVLRIMGVLPLTRTTSGVTYKQLSNRILPVKLYKKSLLIAIIIPILSTTSVIVTHVTMVHFKTSQIIPYVFLEILTYMLGGYWYLLCEILSLCANVLADDFQQALRHVGPAGKVAKYRALWLRLSKLARNTGVANCYTFTFVNLYLFLIITLSIYGLLSKISEGFGTKDIGLALTALCSVFLLFFICDEAHYASHNVRTNFQKKLLMVELSWMNTDAQTEVNMFLRATEMNPSQISLGGFFDVNRTLFKSLLATMVTYLVVLLQFQISIPDATQPEIPTNIDDHVQNITDTTTEASSPISTLMSAFAKRKND

>BmorGR4

MSRIFSMTRYFGVSTCKPSIAFGWTVILLLMLLAIEVGAIWKIVRLLGGWAVHSTDSRGFTARLSGCIFYGNALLSLILSIKFVSSWEQLSERWSRTETDPGLRLPSDSRIKRRTVLVSAFVMTCACVEHMLSMMSATGFDCPPEEYTERYILSSHGFLVQNDEYNLWLAIPIFIMSKLATALWNFQDLIIILISMGFTSRYNRLNTYVHRVVMLERNLKEGAQVSSENYMRFQIWRRIRQAYVRQAALVRLVDDQLGALVLLSNVNNLYFICLQLFLGINSKDRGSFINRLYYFISLGWLMFRACGVVLAAADVYIHSKKALISLYLCPELAYNLEIKRLKYQLKNDEVALTGMGLFSLNRELLLEVAGTIVTYELVLLQFSNED

>BmorGR5

MYACYKIIVALSLNRQYNHTVTRVGNRKHIKSTTRGNFRERILRKVQNRISPEPIQEDSKIPLTCQFQLFQTAMKHLLISGQFMGLNPVSRISDHSPTKIRFTVLSWKFVYGVTIGIAQACATVLCFCKLLKDSVNIVALDFVKIVSAYFAFYLSTGCNTFIFLRVASKWPTLIKHVYETQLDSYIDVKVKNKCFAAYIIFFSMSMTEHMLSLLSKFVITMDCLPKGSDLFESYIIRNFPWLFEFDVPYYLPIGVILQFLTLVSTINWSYSDLFIVCMSIYLTSILKQINKKIEMAGNSNHLPIPFWRTLREDYTRATRLVRSFDDTISSVIFLSFASNLFFICLQLYNILSNGVTSKYNLLKEMCPNYPSGPLGGYEQIMYLLFSLSFLLGRSLVVSLVAAKVHSASMVPASALYNIPRNMYCSEIQRFLDQVHGDKVALSGLRFFYVTRSLVLSVAGTIVTYELVLLQFSNED

>BmorGR6

MLLRNYKQNLSFWTSAKKSKIHKIQSQETVTFQGSLKLVLFIGQLFSLFPVCGLLSNDANKVKFVPISWKCGYSMLSMIGQLFIIVMCILYVAHFETTLNGTTPIIFYGVTFISMIAFIRASRRWPELIQHISKSEELDPSFDFRLKKKCNITLLLVLVLAILEHIFSIRSAYSASQICYPHTGFYEGFVRYLYPWVFDFLPYSAALGMVTQFLNIQSHFIWNFTDLFVICMSYYLTSRLDLVNKKLLPAQGKYLPEIFWRTTRETYCRATKLVRKVDEIINGILFISFANNLFFVCVQLFNTFDDSVDMVGLCYNYSERRTKPVGREPVIYLLFSLGFLISRSITVSLIASQVNLASTVPAPILYDVPSAVYCVEVQRFLEQVNGDNVALTGLQFFSVTRGLLLSVAGTIVTYELVMVQFNQAPASDSFTEKLVENNISTIETFYNYS

>BmorGR7

MVLEAHTQIQYCTAKANYCEFHAGLRHLMRLARWAGFFPVQGLSQTNPDDVRFEFRSLYALYHAITVIGQTVMTFLAFYSFVDSNVSLSVVSNFLFYFTNYVTLVLLWRLSKNWSALISKTLEFEQSVTEIRTTRNLVSRTNTLTYVVLIFAMIEHALSKVFNIRSVMCCLGETSLNHTVINNYFKFKWKFVFDYFSTSTTYSYFVGFIAEFLCMQATFLWSFTDVLIMCFSIYLSSFFEDFNSTVSSFMKKASKTVPWSTLRVQYSQIVLIVKQMDEQLDYFVLISYFTNLFFICFQLYNSLNRIYDANDVCNENMDIIATASVTYLTYYVFSFLFLVTRALLLSIMAANVHSCAQVPQLALYEVPTADYSLDVQRFQLQLRYTTVGLSGVCFNVTRGMILRVIGTIVTYELVLIQLTKKNLDNDTSIRDYYLPKHLI

>BmorGR8

MAPRSVRSMVGTSKKDMLKGGFYETVRIPLYIYRLIGILPISGLWHRSSKYNRFSLKSFYTIIYAPTIVMQTFLLLVHIYDLFAFFFGHQRLGRLIYHMNFYTITILIFMGSRKWKNVIKEIETIELTLPRLRNSKKALALTKSFVFAFFVFSLAEVVLILQFTLRLTKQRHVLPGDSGLYLRSYFVYIFPYLYDHFPFSYVMGFIVQIIKVQGIITLNMVNCSVVILSIYLTNRLKHYNRIVFAKGSKTNNTRLKWVELNLLYTRISNLVKIIDKNLNPFVFISFTANLSYICAQLFYILNKLTSSRTVKITSFLEDKRSDWETVLYISISFALVVLKVLLVSITAAEVHTTSREPLRLLYTLPTAEYTIETQRLMTQVYYSNLSLSGLNFFHITRGMLLGMVATLLTYEIVLLQI

>BmorGR9

MPPSPDLRADEPKTPCLVGGAHAFILKISSFCGLAPLRFEPRSQEYAVTISKGKCFYSYILVTFLVICTIYGLVAEIGVGVEKSVRMSSRMSQVVSACDILVVAVTAGVGVYGAPARMRTMLSYMENIVAVDRELGRHHSAATERKLCALLLLILLSFTILLVDDFCFYAMQAGKTGRQWEIVTNYAGFYFLWYIVMVLELQFAFTALSLRARLKLFNEALNVTASQVCKPVKKPKNSQLSVYATSVRPVSCKRENVIVETIRVRDKDDAFVMMKTADGVPCLQVPPCEAVGRLSRMRCTLCEVTRHIADGYGLPLVIILMSTLLHLIVTPYFLIMEIIVSTHRLHFLVLQFLWCTTHLIRMLVVVEPCHYTIREGKRTEDILCRLMTLAPHGGVLSSRLEVLSRLLMLQNISYSPLGMCTLDRPLMVTVLGAVTTYLVILIQFQRYDS

>BmorGR10

MTMSIKPRLQCMVPPSLALALRVSRLAGIAPLKFVAKQSNIMIRLSTSLCVYSYLLVTALNVCTLIAVMIDFSVPVKLSIRMQTETKRFVWIADVVIMGILSGVGVYTAPIQMRRLIAYLHRIHKINSDLGTYSSSLTDKMLHRLTIGMLLITSVIIVTDFTFVMYLADLNHRQLLIAIMYWCYYCSYFIAHLLEMQFVLIAALALSSLKLVNNGLRTLLHQSGIESLTEIPNSNEQHTANAILPQPPKKSVNNSIDTLAFVVTKRSVRFPTAGWMDQRTIRRLALSYGSICEVVRQIDNNNGIIVLLLLASFLLHLVVTPYYLIISFVTESPHTGFEKVLNPILQTVWCLYHTFGLVMIIEPCHRTHEEMETTRELVSRVMCSADPRDPISIELEMFFRQLVLNKASYAPLKVCTLTRSLVATILGSITTYLIVIVQLEIKNMQ

>BmorGR11

MKPFRFFLFVENVICVYRNYSFHKRYARAIILSRVMFEVSLIILTLHSCRNFGAVKYKTEIIFTYLATASSTILILLALYKTNRFTELFLNFKAFYRNRNLDVDHLEKWNRKQKMATVIIVLFCVIKFSTLIYTDLIGEYSTPCRGYFTEYLFYTNLFMCNARYLFEFSTACVVLHLVSEQLDYIAISMDCTMFLYIDISKKNIMSSAKKRKLKYFDIFKQFEKWTDAYMNVKRSANLCDTVFRAQLAIMITTITLYYIILLYGITSFNIERGKFSVVKSLSYLISLFGFLIALLLLSKAGQRIQKSAENLRRKLSKFLLHSLEDPEFHRAATNLLRLVCTHHIKMRCFGFIDIDMTLLPSCLMFVTSYT

VIALQFNNVV

>BmorGR12

MKNLKLCRTTFYFKIIMCSRFISGLYFKATSKKWISYLYKVICVLYIICITRLFYAKEDTFKPLVFXXQFIGNSIESLRTGEGHVLKCYSTIFSLKLIRNYLPDSNNHIPISSITNFLVIIWKVFDQVYIVLMHYFYTTDIIIHLRILSILTTIGVNLSLMPIIVIFELMWRAVKALRKSLGEHLKGPVLIEGRERLKAQQILRCLNVYKDLNATLKFNSTPMKTMILISTLATFIRLTLFLYQAILGHNEGLHLPRKILAIIYYALPVCLLGVLMELVARECDKLKTLMTKELLVCKDDSYCTVIVDAVSYIELNPLKFSILRAFNVNSTLILGLTNLCTTYLIAVIQFTYSCEDINGLSHSHSH

>BmorGR13

MEDSFNRLLSIRNMIIFQNVCGFYHMCTEKLYISRIIKMYCVALAIVLSVFCFQNPDITYLSWDVVWVTFGYTLNVIICLRYNGNYFFQYWNGLHEIDIKMNLTSIDKEKVPISRAVFTVFLILRSTAFAMTIFVFGYLETGILSNTIISIYSINLTEFYRNMSNIPMILMFETFYVRIKILKEQLCSELSTVLGCNNDARQLKLILKYLRNYRSLVRHLMDTTLPFKILILVILVGSFLRSLLIGYAFVYNSDQIILLSLPVMFSTKILSEVVEIKLICTKELLKNKNEGLVLLDLDSKKPTFLTSKACGEQLQDALSFLNNRSYSYTLLQVIEFDCSLAFVFTSFCITHLIVVVQFTHVLD

>BmorGR14

MNLHKNIIPIRNNLFANKVTAIALPKTLSVLFKLIHIFFLLDLGVYEYKTFKIKCIVKFLTISGSLTISVVCFSFMVSNLSEHTFVGWYGFFISTYIFVVLFFNLSNRMTFVEFYKTLLRFDANYGIDSNEYKFNFKIIFVNILFIANRMVLSFVYCSYYPQNCIRPRYAQILFMLPWLTLDVLLTTNMFLFYATYCRIAKFPMLIKNSMNIVALRNSYKLIVDSLEKTQTSFDIVFIIALVFSVPEIMMSIYSTLLEVISKHFLEVASILSLNYVAIAQSLLLTLAPSLCAGVLPWKTNNIKIILHEKLFTEKDKASAREIELFIKYIESRPLKLRACNLVPLDFSLTIIVLNICVTYLIVIIQFTHLY

>BmorGR15

MISSSDINHKRNKVFAYNVPGIALSKTLTVLFKLLHYVLLLDVGIYEYKTFKNKCIVKFLTIATGVSVSIVYFCLIATVLRKNAFFYWFYVLFISQYMIIVFIFTLSNGMSFTDYYKMLLRFDAKYQINSNNYYFNIKIILVIIISILNRIGMAIIYCSYYTKNCYEMSFSQIIFVLPWLTRDVILIMNVFLFYVTYCRITKFPALLENTKNVGSLRNSYKLIVDSLEKTQKPFDFVFTISLVFNIPEIMLSIYFTLLQVIHSHFLEVAPTLSISYFSITHSVVLILAPSLCAGVLPWKTNTIKIVLHDKLFLEKDKNSARNIKLFIKYIEARPLKLRACNLVPLDFSLPVIVLNLCVTYLIVIVQFSHLS

>BmorGR16

MIMNLTTDRISKRNKVFAYNVPEVTLPTTLKVLFKLIQFTLSLDFGVYKYKTFKMKCVAKVLTLAGCLAASAACVSLIISNIFENQLFFGWYTLFVCQYTIVIFMFTFSNGMTFIDYKMMLLRFDAKYQIDSNVYHFNIKIVLVVVISVTSRLFLCAVYCIYSTENCIKPWYNQLLFFPWLSLDIVLIMNMFLFYATYCRLAKFPSLFENPKNVVPLRNSYKLIVDSLEKTKKSFDAVLIAALIFNIPEIMMSIYYTLFQVMNKHFQEVAPVLSLSYFTIILSVLLILAPSLCAGVLPWKTRHMRLILLEKLFAEKDKNSAREIELFIKYIEARPLQLRACNLVPLDFNLPVIVLNLCITYLIVIIQFT

HLF

>BmorGR17

MGFSLGTTALSMFFFEKPVVFTIIQITMIIVKPAKYKLSDPFRPKDTSKLSESIIMYFKLFHIFLGIDLGGFRYQNRQVKYAVRLISLIQPLAIYGLCIYALLKIIANTEFLWYTISFTEYVAMSVAITLFSNEMTYCNFMINLKFIDTKLKIGDESFRIGVKLISSTILIGVTRCFTTTTYCLLGFCAKPTAAQILFQIPWLTIDLMLLQYMFIFYACYCRLVKILRILKKRNTDIEEMRRIYKTLVDVLDRARAPFDLAYLLGLLFSIPDVLYSIYESIIKVGEINTAKALSMSIIYITNIQSLALMFAPALTAGFLPSLTMKMRIILHDKLLEEQDKKTYRHIVLFIKYIETCPLKLKACQIIPLDFSFPIIILNIV

VTYLIVAIQLTHFL

>BmorGR18

MRRSTKVISMVNQSDKGEIKTCSRFMKIYFFVIYILTGFNFGFYTGRGLNFLRVIQASVLLLRFIIASNCIYIAFHFRLLEAIWYSLTFSESLAIVVCFMLSRSALSCKNLFEYLYSVDQELKKSVGPSIEVKLALYTVVVSVLRLTVYVFCAIAYYETLHEGFCVELVYNTPCYCSDLYLVIHFTIFHSVYCRLKALRISMNEKFDVYKGTLIYKSLIDNLEEIKKSLDVPFFVILLNAVAIAMINILVTLEISYGQTMKFIRTAPRYLETVLLFSSAFAPVLAADMMASEAQKIKVTLNNILQRDDSLLEDDRRKVKQFAGYVSARPFRLRACRVLSLDCTLPVTVLSICVTYLIVVVQFTHLY

>BmorGR19

MRRSTKVISLVNQSDKGEIKTCSRFMKIYFFVIYILTGFNFGFYTGCGLNFLRVIQASVLLLRLSVASYSMYIARYSPLLEVIWCCLTASENLAVVVCFMLSRSALSCKNLFEYLYSVDQELKKSVGPSIEVKLALYTVVVSVLRLIIYVFCATAYYRKLFDGLRLELLYHTPCYSLDLYLVVHFTIFHSVYCRLKALRISLNEKFDVYKGTLIYKSLIDNLEEIKKSLDVPLFVILLNAVAIAMINILVTLHISYGKTGCRIDYKSVAEMACSQLVIRTSRSETANGSSEAKRKKLVVGPLMKLITAAPRYLETVLLFSAAFAPVLAADMMASEAQKIKVTLNNILQRDDSLLEDDRRKVKQFAGYVSARPFRLRACRVLSLDCTLPVTVLSICVTYLIVVVQFMHLY

>BmorGR20

MRRSTKVISLVKQSDKGEIKTCSRFMKIYFFVIYILTGFNFGFYTGCGLNFLRVIQASVLLLRSIIASYSIYVAIHFRVLEAIWYCLTFSESLMVVVCFMLSRSALSCKSLFEYLYSVDQELKKSVGPSIEVKLVLYTVVVSVLRLTVYVFCAIAYYESLHEGFSVELIYNTPCYCSDLYLVVHFTIFHSVYCRLKALRISMNEKFDVYKGTLIYKSLIDNLEEIKKSLDVPMKFIRTASRYLETVLLFSSAFAPVLAADMMASEAQKIKVTLNNILQTDDSLRKSYGATVFSFALLVTMIVEATMVGVEDNNIIGPYIDVEREARLTGLYVMAIILTLMFLAKFIFDLVFVYGVVMERAGIVKAYFIMWAVFFFLSVSVFFLNCLDFNTSTIVLEVFYIGLNIYAILLSHSFYKQLNTREDV

>BmorGR21

MAQRTNSINLFRSRPPDIRAGVGEPRIFSKFICGTMFTQKSLVNFDLGKTPRGGDQEHSKFFKIYFLAVHSVTVLDFGFDRNAKKLTKILISMFSISVRMGLAAVSFMSLWGRPNALALGWAPGTLLCENILVAVTYSASRSTFKCGDLFADLSTIDELFGSACDYRIESKMLLFTATMTVLRVVIYSTSRLVRADGFDFVDVLEVLNNLETMCMYLFLTVYFFVLFSIYCRFKKLRELMKNDFEIRRANLIYIALKDCTDKIKQSLDVPFLVVLVFTVLVVMVDVFITLEMIISNKYNMAVYVVRYLEITLDFLMLFAPVLLADMMAVQVDGLKITLHDRLCLNNGVGHDDLSDAMWMSFVDMKRYSSLAEFIGYVEARGCRLRACRVVPLDLTLPVTVFNVCVTYLIVMIQFADLY

>BmorGR22

MDFGFSLGVYKRMKVLENISLVLRVMVAIMCAAMVMKQDILDSAWADITLTESLLVIVSFKLSKPKLSYRELLENLSIVDETQGAPPAGYKVERKLITYIAGVTALRLTVLCLYCVAHTEQYSIDNFIEFLYNVPCYCLDLYLIVHFIIFHSIYCRLRTLRKALSNNFDVYRAHLIYKTLIDCTEEIKKCLDIPVSRSDRHRHSLHKPMGTFLKSYRTPRVMFQLVVILIATILVVMVNVLVTLRMLFKGEVTQRGSVHVQVVLPENLPLSYFNIVHCISLQSIISAFLLRYIEVILSLALLKLLDELKPDAFKSHWKKIWRVLIVGNDGQFARNQTSTDNIVQAANQWNMTRWIRADRTSDIREVAGSQDHLTETDLEEMLTTRSCVTAYWIFDAGSEHVWYVAAAEERREVRQFAHYVGTRPFRLRACHVLALDSSLPITVVSVCVTYLIVIVQFTHLY

>BmorGR23

MAQFQIPSMAGSGLNVAPFSRGRRHEGPKHSNFTKKYFLLVHLVTCLDFGFHRDNDTKTYKWFHAANIGVRLVLSAYVCSVSLSQDLSFASAAWTILNNSKHLLVVAIFTIFKPKSSCAEILKDLLMIDEALKIHRGCDVKGQITVCIVLVTAARLLIAAASSLSLHEAFSASVGAAEVLYSFQSYCLDFYILANFFIFYSVYCRLKNLRRVLQNNFNIYRGNMIYKVLVEHMDDIKKFLDIPFVTSLLVTVIMAMINVLKTLQLIHDGENDVLTIVLRYLEMFLSFSLIFAPVILSDLMSIEADNINVVLHNYIYETDAADIAVAVHPSARPSPVSATEERTAAAAAASVSLAIFSSRLTSFNLCWRARGAGAETFVAVCVVRLGAAVPTPRVSGALAGLHAPRDRAQHLRHLPHRGRAVHASLLISLSLL

>BmorGR24

MCINKKIQSIIKSLVSIRTIMLVQSILGFYHKMSNNFFVSFLFLTYTTILISVLSFYSVNDVMAHKFAYTLSMILEYDINTILSLITAGRQYFNFFEEMKKIDFSIGFGELNIEDLPLSRTLFVTIFVTNILLSIMTAALILFFSTPFLIISSGSTYAMAIVFFGLSLNVLPRIIIFELIYKRIKYINLSLKRK

LKALALECDHTIARFEIINENLIIYNKLLQSLGNVNVSLKSSILLTTFTCFFRCSLICYYVITMNEDKVYIMQIIELTKQTLFLGVLIILAEYIKNEIENLKMTVSLQLFTCTDTQLYHQVSDXFEIHRITPIQLCGFKNMSVDTNLFLGLINVCSTYLIIITQFLNAYVN

>BmorGR25

MFVKCLKYVKKFKPMFSVMFIMNFRLICGLYYRIHSDAFVCFVFKVYCILCSMFLFFTSSDLAAPFSRSIPILATLFEYVANVLDCILTGQSYFFHLRMELMRIDPRLRGLDRPPASSIVFTAILSYKIFILAVYIHGKARTTYLQYEWFSSIGIHLLVLFSNLVHMNRMLIFEMVTFSLEAQKKTLGELLKSSLRRERVERKCEILNRFLKTYKRIIELFNNTMAATKLMTLISVVSCFIRILTYLYQVLTTQLSSSSAGSIFTTRHLTFIVTFVREHVLSVYFLKKVGICQRLQINFKIKCNVKTETLELISQDDEYTEKLEDALDFINSCSSKITILRAMTVDATLPLTFISLCTTYIIVVIQFSHIYD

>BmorGR26

MNKTKIYRKKLDKNERLVCSVQPAMFARLIVGLYYDIKVSNRVKWMIKSYCISLSSFICYLIIFRDDNFSLHPKLTSVMEYITYVTFSFLTCDKYLFRYLRFNPRTDGYPIFLYLCKKFEKFFKIIICLFVSFKILGVVLMMQSWPILSTPKYIWGTLALHFLWLASHMGRLVFILVYGILFCRMRTIRIIFENRGFQNTPQNRLTPKRYILMYEAVLNSIESVDFPVKFLIFTFICCFAPKLVVSLFEIMEEMKKGELSLTTFIWFLVELSPSYLFLLLSAIALDLVSEDVQELLSITIDRRLNCKNEKERSEIQEFFQYLRNNPFNYTLWQVVSLNLRTLLVATSFSIANVIAIMQIKNSKI

>BmorGR27

MVFKYKIMTKAPKSLPVLKILMLFRLVFGNYFRLSSNRYINFLVKSYCSTFTILLSVMCGKRLKNDSPYMLSLTEYILNKILNYATSEGYIFKYCNSIKTCDKIMGFKKLPIITIDVFIAIIITVITRTAITIYFGFLFPFDKYQVVLYVGCIVFSNDLNSLTIMNVFGLLNNRMNLLRKSLEAMTVPINIIGKNEVAPKVRLVRNAFRYYSNLLDNLDSVNHCVQYSLSVTLLLKFPKAVLLCYDSIKTYFVKIDNNFAMDIVDPTEIILSIVVMSFPAMLCEMITNEVEKIKAILTKHLIQCSDNSLRFELNITLLYICHRPFKYILWRAIPLDTSVPIGIVSLIITYVIVLIQLLHFST

>BmorGR28

MAHKIATVGPTNATATVKNNKRKLKISKRVTIFKVVRCLRFILGHYTELTSSKLKAFLIKCCSLLLAVIIIYAPLNYIKMAYVMGLIEYLLFVLLSLFTGDEYFYKFHNSIKSIDVLMGYKRGKIIDSNAIIFLLSVITIMRIVIIYCRSTVLAFRFTIIGVYLAIFSLRISYMLITVIFFAMYHRMKFLRKKFEIITIPVTIIGKQKVASKIRLIRKYLINYHHLLDCLRDINGGLQYFLAIMIACNLPKYIFFAYSAIKIQVLEHITIHSAVQNVEMFEGFLFVVVPAIFAELTTAEVERIIDVINRQLLRCTDEHMELELKVALEFIRRRPFDYVIWRTVPLNASLPIAIISLCITYVVIVIQLTQFHDNF

>BmorGR29

MYLRSKKSRFKLFSFERMIKILLMICGHYVQTDSSNVVSSIHRLFSIAITICLCPNFEFNPFYFHVIESVLYSILSQFTQYGFFFRFCSTIKTFDLLSGFKQIPLYTKRVCFFLLITLFMRLFTVLIHFLAYQSKFVTFCAFIIMLSANTGHILMTIMFSTLHTRMKSIQKLFANNPIPVNIVGKNENASHIKRVRKGLICYNNLLDTLKDAEKEIQFTLTVTCLCHVPKIICYVYFVITVIYKSKFSGYNLVPLFDMILACMAVTAPAVFAELTKNTVDKIKKILGSQLLRCSDESLRYELEITLEYVIQRPFSFSIWRAVSLDASLPVAMTSLCITYVIVILQLTQLRP

>BmorGR30.1

MYLRSKKSRFKLFSFERMIKILLMICGHYVQTDSSNVVSSIHRIFSIVITICLCPYFQFNPFFFHVIESVLYSILSQFTQYGFFFRYCSTIKTFDLLSGFKQIPLYTKRVCFFLLITLLVRLIIVLIHFSAHQTKLKTFCAFLIILSANTGHILMTIMFSILNTRMTLIQKLFANNPIPVNIVGKNQNASHI

KRVRKGLICYNNLLDTLKVAEKEIQFTLTVTYLCHVPKIICYVYFVITVIYKSKFSGYNLVPLFDMILACMAVTAPAVFAELTKNTVDKIKKILGSQLLRCSDESLRYELEITLEYVIQRPFSFSIWRAVSLDASLPVAMTSLCITYVIVILQLTQLRP

>BmorGR30.2

MYLRSKKSRFKLFSFERMIKILLMICGHYVQTDSSNVVSSIHRIFSIVITICLCPYFQFNPFFFHVIESVLYSILSQFTQYGFFFRYCSTIKTFDLLSGFKQIPLYTKRVCFFLLITLLVRLIIVLIHFSAHQTKLKTFCAFLIILSANTGHILMTIMFSILNTRMTLIQKLFANNPIPVNIVGKNQNASHIKRVRKGLICYNNLLDTLKVAEKEIQFTLTVTYLCHVPKIICYVYFVITVIYKSKFSGYNLVPLFDMILACMAVTAPAVFAELTKNTVDKIKKILGSQLLRCSDESLRYELEITLEYVIQRPFSFSIWRAVSLDASLPVAMTSLCITYVIVILQLTQLRP

>BmorGR30.3

MYLRSKKSRFKLFSFERMIKILLMICGHYVQTDSSNVVSSIHRIFSIVITICLCPYFQFNPFFFHVIESVLYSILSQFTQYGFFFRYCSTIKTFDLLSGFKQIPLYTKRVCFFLLITLLVRLIIVLIHFSAHQTKLKTFCAFLIILSANTGHILMTIMFSILNTRMTLIQKLFANNPIPVNIVGKNQNASHIKRVRKGLICYNNLLDTLKVAEKEIQFTLTVTYLCHVPKIICYVYFVITVIYKSKFSGYNLVPLFDMILACMAVTAPAVFAELTKNTVDKIKKILGSQLLRCSDYS

>BmorGR30.4

MYLRSKKSRFKLFSFERMIKILLMICGHYVQTDSSNVVSSIHRIFSIVITICLCPYFQFNPFFFHVIESVLYSILSQFTQYGFFFRYCSTIKTFDLLSGFKQIPLYTKRVCFFLLITLLVRLIIVLIHFSAHQTKLKTFCAFLIILSANTGHILMTIMFSILNTRMTLIQKLFANNPIPVNIVGKNQNASHIKRVRKGLICYNNLLDTLKVAEKEIQFTLTVTYLCHVPKIICYVYFVITVIYKSKFSGYNLVPLFDMILACMAVTAPAVFAELTKNTVDKIKKILGSQLLRCSDESLRYELEITLEYVIQRPFSFSIWRAVSLDASLPVAMTSLCITYVIVILQLTQLRP

>BmorGR30.5

MYLRSKKSRFKLFSFERMIKILLMICGHYVQTDSSNVVSSIHRIFSIVITICLCPYFQFNPFFFHVIESVLYSILSQFTQYGFFFRYCSTIKTFDLLSGFKQIPLYTKRVCFFLLITLLVRLIIVLIHFSAHQTKLKTFCAFLIILSANTGHILMTIMFSILNTRMTLIQKLFANNPIPVNIVGKNQNASHIKRVRKGLICYNNLLDTLKVAEKEIQFTLTVTYLCHVPKIICYVYFVITVIYKSKFSGYNLVPLFDMILACMAVTAPAVFAELTKNTVDKIKKILGSQLLRCSDESLRYELEITLEYVIQRPFSFSIWRAVSLDASLPVAMTSLCITYVIVILQLTQLRP

>BmorGR30.6

MYLRSKKSRFKLFSFERMIKILLMICGHYVQTDSSNVVSSIHRIFSIVITICLCPYFQFNPFFFHVIESVLYSILSQFTQYGFFFRYCSTIKTFDLLSGFKQIPLYTKRVCFFLLITLLVRLIIVLIHFSAHQTKLKTFCAFLIILSANTGHILMTIMFSILNTRMTLIQKLFANNPIPVNIVGKNQNASHIKRVRKGLICYNNLLDTLKVAEKEIQFTLTVTYLCHVPKIICYVYFVITVIYKSKFSGYNLVPLFDMILACMAVTAPAVFAELTKNTVDKIKKILGSQLLRCSDESLRYELEITLEYVIQRPFSFSIWRAVSLDASLPVAMTSLCITYVIVILQLTQLRP

>BmorGR30.7

MYLRSKKSRFKLFSFERMIKILLMICGHYVQTDSSNVVSSIHRIFSIVITICLCPYFQFNPFFFHVIESVLYSILSQFTQYGFFFRYCSTIKTFDLLSGFKQIPLYTKRVCFFLLITLLVRLIIVLIHFSAHQTKLKTFCAFLIILSANTGHILMTIMFSILNTRMTLIQKLFANNPIPVNIVGKNQNASHIKRVRKGLICYNNLLDTLKVAEKEIQFTLTVTYLCHVPKIICYVYFVITVIYKSKFSGYNLVPLFDMILACMAVTAPAVFAELTKNTVDKIKKILGSQLLRCSDESLRYELEITLEYVIQRPFSFSIWRAVSLDASLPVAMTSLCITYVIVILQLTQLRP

>BmorGR30.8

MYLRSKKSRFKLFSFERMIKILLMICGHYVQTDSSNVVSSIHRIFSIVITICLCPYFQFNPFFFHVIESVLYSILSQFTQYGFFFRYCSTIKTFDLLSGFKQIPLYTKRVCFFLLITLLVRLIIVLIHFSAHQTKLKTFCAFLIILSANTGHILMTIMFSILNTRMTLIQKLFANNPIPVNIVGKNQNASHIKRVRKGLICYNNLLDTLKVAEKEIQFTLTVTYLCHVPKIICYVYFVITVIYKSKFSGYNLVPLFDMILACMAVTAPAVFAELTKNTVDKIKKILGSQLLRCSDESLRYELEITLEYVIQRPFSFSIWRAVSLDASLPVAMTSLCITYVIVILQLTQLRP

>BmorGR31

MYLRSKKSRFKLFSFERMIKILLMICGHYVQTDSSNVVSSIHRIFSIVITICLCPYFQFNPFFFHVIESVLYSILSQFTQYGFFFRYCSTIKTFDLLSGFKQIPLYTKRVCFFLLITLLVRLIIVLIHFSAHQTKLKTFCAFLIILSANTGHILMTIMFSILNTRMTLIQKLFANNPIPVNIVGKNQNASHIKRVRKGLICYNNLLDTLKVAEKEIQFTLTVTYLCHVPKIICYVYFVITVIYKSKFSGYNLIPMLDMILACMAVTAPALFAELTKNTVDKIKKILGSQLLRCSDESLRYELEITLEYVIQRPFSFSIWRAVSLDASLPVAMTSLCITYVIVILQLTQLRP

>BmorGR32

MCYTNFVSRQVSKCINFFSTIRYVIYLRMFCGLYYNCSSSFKIRCIARLYCFIIYCLNLHYNLYIFTSSVSLTNFLHTFITLAEVSIHILFSLYTGESNFMSFCIEMNKLTSGPIDFVATKCVATHFIAFFVIGLHILSSTLICGAEVSCFTFSVVLASMTFLTTLLSRFTTIIMFDLVWIRMRSLRKILVNALESDLSEDEKVKSIESFLKAYKQIIASIRITKLATRNLVTFNFVSLFGKIMTLIYFCINCPGYLNTYLISSWIFGILLAGFVTCAPPVLVEMNVNELDEIKYALADQLVDYTDDKYRTAIYNALDYVEVHSIRYTLWKNFPMDLTMFFGFAGFCATYIIGLLQFTY

>BmorGR33

MCYTNFVSRQVSKCIHFFSTIRYIIYLRMFCGLYYNCSSSFKIRCIARLYCFIIYCLNLHYNSYIFTTNVSLTNFFHTFIILAEVSVHILFSLYTGESNFISFCIEMNKLTSDPNEFIATKCVTTHFIAYLVIVSHILSSTLICGARASCFTFSVILTSMTFLTTLLSRFTTIIMFDVVWIRMRSLRKILVNALESDLAENEKAKSIESFLNAYKQIIASTRITKLATRNLVIFNFVSMFGRIMTLIYFCINNPGYLDTYHMSLWIFGILLAGFVTCAPPVLVEMNVNELDEIKYALADQLVDYTDDNYRTAIYNALDYVEVHSIRYTLWKNFPMDLTMFFGFAGFCATYIIGLLQFTY

>BmorGR34

MCYTNFVSRQVSKCIHFFSTIRYVIYLRMFCGLYYDCSSSFKIRCIARLYCFIIYCLNLHYNLYIFTSGVSLTNFFYSFITFAEVSIHILLPLYTGESSFMSFCIEMNKLTSGPNEFIATKCVATHFIALLVIVSHILSSTLMCGARASCFTFSVILASMMFLTTLLSRFTTIIMFDVVWIRMRSLRKILVNALESDLAEDEKAKSIENFLNAYKKVIASIRITKLATRNLVTFNFVFMFGKIMTLIYFCINNPGYLNTYLISSWIFGILLAGFVTCAPPVLVEMNVNELDEIKYALADQLVDYTDDNYRTAIYNALDYVEVHSIRYTLWKNFPMDLTMFFGFAGFCATYIIGLLQFTY

>BmorGR35

MYSSLKLKDYIVNLESVMCSDQSITISFKNVFTVFFDYISSLDFMMVCRLCFGYYYEFNCSNLCKIMFKCFSISVCIFCVSMHLVQLISPPYLNHCVIMLESTVSIITSLVTEDKYFFEFCLDMKDINSMMNQPRNIKSFKIIYVIISGAICHVIRHISICREKALSFCFSTEYLTASFTIISGYWNYLNITMMFDLLYQRLVAVKQMLTNGLNICDTDEYKIKSVQKFIDVYKALTVSLSKTSNLIKHTVSLGIFCALWRIIFFVYYCISMDFQVESAQFITWVSSMCLSVFLVYIPALIVELCSNEVDAIKWILASELLEYRDKRLRTSLRDALDYIDVCPIDFEIWHCFPMNLSLCLGFIDISSSYIISIL

QFKY

>BmorGR36

MTVPYDKIKSALSKFVQLLFSINVVLFVRFLFGFYMKIGSRKYFHIATKIWIVTLTIFRVYFQCRNFMNYPSYALILHDFTCTVELVILCIISSLGGEQHFYTYCSEMAELIDNRKNKRASYFTTSTLLIGFIILIVPTSISCKKVSNALSMLFLNIFNYVACFMHHLTIIYVFELLWREIRKCRISLERLEIMSVDDKIMKIENFLDSYKRSLDSLNKANGVMIPTMALAYFAIIAKIVFFTYNILSLRGFNILVHGDSWLLSTSIAIIFICAPALLVELAANEVNKIQNLLAVELLKMKDDKF

>BmorGR37

MSAKINYQDKLCSIKSIMYLGLFCGLYFRSSTSRMMLLMTKVYCIVLLFLGISFHLNMLSSDLPTETTLHSSIIMLEFFIHIITSVSTGQAKFLHFCTEMMKINGHNSKGSLDFQLVITNIALIIIIITQTTSSLLYCIIRSKCLSHSYVFTVITSLCVLFSSFTMIIKYELIWNTVRSLKNTLVSNLDSFDLSEQEKVNSVYNFLSTYRDIKANVDLTIKGTRTTMIFNLFFLFVKILFLVYYCIINAENLDCLHASARVSAIVLSVAVTCIAPILVEINVYEFGRIKFALADQLLEYTDNNFRIALHDALDYIEVHSIRCSIWKNYPMDLNLCIGFISVCATYTISILQFSY

>BmorGR38

MKPKYVTNIKLVLFLRFLCGYYYEMEIPRRLKTVAKAYCIFFLFFYLVLHHLYCSFSNHTAKWSLYLEYSIYVFMSLYSKKMYLMDYYTSSRIIDFEPHSRIYKKLNIYLAILIPFLVVLKIVNMVTFCLSKSFNCWSWVSLLHNLLWNFTVLGRIPPVFVFALLFCRTRIIRRTLVSITVGPGSTVLKSLFKCTRYWLTVLKRPNTHLNSCYFFQLTVFLLCSTPKLILETFLMLNKIKESGPIVEKLAVYAIETFYTHLFFVVSSILFDLINVDLQRIKILIVEKRMKTKNTKHRIEVEKLFQFVKSQTIECTLWRVLSLNVRNILSFVSFAVTTIIAVLQIKNNNIY

>BmorGR39

MNVSPQYSVLKIFKPLFKVQTLFGSVRVKVGDNGITKTTKLQKFYSIFNILFATTGHFYTSFVYSVCVPCVGNSVAETSMALIQIYAGHLMNSFIVFSNTFLHYEKNVQMFKSLCSIDELMKIVRLEHRDLKLFIAIILLLSSTVIMNVYFLIYMVIILPISEKWVPFANIGIMNEDLEAITFVSVLYMLYDRVKYINQTALDPVNIAKLIKESDGQNDEETVSRILKAFKEISKAYKIVEKTFRVFFMFLVIHMFFFQMLTVEILIVLSDNLSWGTFMSKNLIGVKFIARIMLISVCVSYLERELQKTKGLCNLAVRNCENDIVRCHLKNIYRIIDTEIEPMTVFGLFYINNVPLDLISLTATYTVVLL

QFAFL

>BmorGR40

SMLVFCVLWSGLCYTGFIGICGMNFIWIATFMGRMVLPLIFGILLCRIRMFRLTLQKQGFDNLPYNRFSPRRYIMMYDSIVRGLEKTDFPAKNIMFVFTICIYSKILTGLFDLISVLKREGPKLMNVMLFTLEFLPSYVLLMIYSVTLDMVSTEMKEILKIVTEKRVFCKDKSHANIQELCQYIKNNQLKYTIWRLVSLNMQSLLRATSFCIVSTIAILQIKDWNG

>BmorGR41

MSENSLEEYIHMSFSPIYKYQKFLGSNRISLKAKNKITVANNWEKLYAFLWMLAASYSIHHFISFFYSYYYERSNIIFLACSLGISMHYLTYILTITYDKFLTREADIDLFIDIQKIDRLLKLDRCTVLFKKFRLINIFLLILVTVPFISGFLIHVFDYIDKPYKTFFLGLGVTITYVDVLVTAFFITKLTLRLAYINDRIAMYNKINIPHKKYSGIRRSILWIFGWRIFKIMPKIKKNGTREKKSTFIKYPSIIFNILKCYRSITEIYSLPVFLITATVSIWTFLVIGSLVAGSRSEIKIFPVVAMITVGLWNFYYIIQLTSLAFVNDLFLMEVKNTKQLCISVLLYTCDDSINKAANTILKNIECVPPIFSVYGIFVFDKSIILFLFGIITSNVMTVIQFSY

>BmorGR42

MNKTKKIERLSRDILDEDFIDVFKSIFIFQRIFGLLSVNITYKYITETSKLYKLFVMSLWTVNVLCLVDYILNYRTSFDVATDSMLKLVMSVNVTTNALIVWRNNFKLNTLKSQIYVKLQNLDRDLKTKDAVTMNKKLSALSIALMICGFIWCTIWLFVYNAIAMNTFCVPLTIILSANVGNWLEMVLLFIIFYFVNVRAEYVNKLLRRRLNQTECPDRVFLIQNAKPSDTVSREFICGMQSLLEIIGNIKDIYQFPIFLSTCQVMLCILVIVQNLIISVKEQMGGRAHSPPGFKWLLEPIDIYNTSTMVDSMLCMLPALLLMLTIFFSLCVIAEALTSKLDITKKLCAMGMHSFTDDISRRSSKQIVLLLEAKRSMSVFNIYTLGTRLPIHLLGVTASYTIVLLQFAVL

>BmorGR43

MKSPEYLSKDILDEDFVRVFRFPFLVQMALGSCRVHLKARFITIPTLGQKLYTVMSIIICSLLYFNITKLYISLYYQHSIVYYLFLAVAGLDQLSFFANLIHVRFLNGETNTGFCIMMQRIDRKMKIDHNNIFNKTVIRANILTITLIILLYMSLVISTIILKKYSLVTLFGLVHGQLILLVEMAYCSNLIIFFFIRVRFVNAIIKNHVHPENQNQPPKLVRYFVTNRIMRYLAAQTHDFIVNDTDVYLKQIFEGFSMFIDIYRFQASMPLLHKANCNEPANLRILLGWDTKKFFHTEFGSLINSPCDFAFFFSQEKIAGSPPARQVLASKNLANYYVIVHSVIGFITALYISGRCEFFFREIRETKRLAVAVLLKYQEGTILTIAYFPKLS

>BmorGR44

LGGSRVAICLFPFEHLALFLGVSCSYKISGMPVWTPLQIVVLSMTSGTRGASRCTTLVVLDVLRLRMNFLKRLLEKTMQSEISNQQKYQFLEQFAEHYRYLLEALDSTRPHSAIVVTTMLMTSFVKVLIIIYFVITNSIMTIDNSRLTYMMMTSFEVAAVSVAPALLEMANNEKDQIIMILADQLLKYTDSKLRRSVYDVVEYITVAPPTVTIWPEFTVDLGLYMDFIGLTVCYCIMILQFEY

>BmorGR45

MKSPEYLSKDILDEDFVRVFSFPFLVQMALGSCRVHLKARFITVPTLGQKLYTVMCIIICSLMYFNMTKLYLPLYYEHSIVYYIFVTVTGLDQLSFFANLIHLRFLNGETNTAFYIMMQRIDRNMKIDHNNIFNKTVTLANILTITLIILHYVGLVISTIILKEYSLLSLFGLLYGQLMLMVEMALCSNLIIFFFMRVRFVNAIIKNHVHPENQNQPPKLVRYFITNRITRYLAAQTHDFIVNDTDVYLKQIFEGFSMFIDIYRFQVCPLCIKLVVLTLLNFEFCLVAIQRNVLGPNHIGNYYIIVNSVMGFFTALYVSGRCELFFREIRETKRLSVAVLLQYQEGPLREKATRMLKIIEESTPQFSIYDMWQMDGYTFVKICSLVTNLIVTLLQFAYL

>BmorGR46

MITMKSPEYLSKDILDEDFVRVFRFPFLVQMALGSCRVHLKARFITIPTLGQKLYTVMSIIICSLLYFNITKLYLPLYYQHSIVYYLFLAVTGLDQLSFFANLIHVRFLNGETNTAFCIMMQRIDRNMKIDHNNILNKTVIRANIFTITFIILIYVVLVISTIMLNEYSLVTLFGLLYGQLIFMVEMAHCSNLILFFFTRVRFVNAIIKNHVHPENQNQPPKLVRYFVTNRITRYLAAQTHDFIVNDTDVYLKQIFEGFSMFTDIYRFQVCLFCIKIVVLSLLTFELCFVAVQRNLLETKNLTNYYIMTYSVIGFFTALYVSGRCELFFREIRETKRLAVAVLLQYQEGPLREKATRMLKIIEESTPQFSVYDMWNMDGYIFIRICSLVTNLIVTLLQFAYL

>BmorGR47

MIDFKTRNVCDINTIMKIRFLFGFYCDFPFNKRFQNILKFYCISVLVVLILGSWACSTGFRSDKKIVIYCEYIAYFLISLSTKDRYIFDYYKQQPLIDGSTTSKVLYKKLERLLKYFVTITIVLKMLNIFVFCGWNLTKCINELDGVLFINLLWIGLLLARLSLPVIYGLLYFRLRVLRMTLESKGFSNSPQNRFTPKKYITIYEKIMKDLLKMDYPLKYVFIIFLIGSVPKLLQNSWQFLNSLKNYGPEISKILEFTLECLHSYIVIILPIVVALDLSEDEIKKMKIITLNKRLACLNERQKMEIQQLFLLLKNNSLRYNLWRVVPVNLKSVLIFLSFGVTNAIAIMQAKNLN

>BmorGR48

MTVFMLKAQVDQDTILEKVVKPLNNTMFNNQEWSFQQDSAPGCSLGTSSQYLTYILTITYDKFLTREADIDLFINLQKIDRLLKLDRCTVLFKKIRLIYIFLLILVTVPFISGFLIHVFDYIDQPYKTFFLGLGLTIIYVDVLVTAFFIANLTLRLAYINDRIAMYYKRSLPLRKDSGIRRSRSWICGSWIFQIMPKIKNNGTRMKNYTFIKYQSLIFNILKCYRLITEIYSLPVFLITATVSIWTFLVIGSIVAGSRSEIKLFSIVAMITVGLWILFFIIQLTSLAFVNDLFLMEVKNTKQLCIRVLSYTRDDSINKAVNTILKDIEYAPPIFSVYGIFVFDKSIILFLIGIITGNIMTVIQFSY

>BmorGR49

MAGIRTISFKVKPLELPDVSENNFADDGLKIVQRFKFFIYIQVITGINRLYLLKCNKFVMLFSYLYAIFLISFVASVYWTKEPMKNSHLVIRLFSFIEYILLICISVFLKKKKMMKFFENLSMFDQILKIDKNVNSTFCMKRVFFWVTGSIVYNLIEFYALEFYDNTSKGLMTIICTYTIALTHDCEQIFFFTLQRVVYLRLLVVKRHIQEHFKVDEDSNRKKPNKYEMLSKNVQLNLTALHEVYGLLHNCAEKLNKIMSIPVLLMLFTSGLTTTILLKILVRVIQLADPSNPGPAAIGVCVYLIVHCIKYTLLVVIPCYYSSITATQVSLIRITLHDAINTIPLGKCKLQRRKVKAFYLMTKEYSFVYTLAGVIKLNMSLPLSYISLCTTYLVIIIQFSKFLD

>BmorGR50

MAGIRTISSKVKPLELPDVSENNFADDGLKIVQPFKFFIYIQAITGINRLYLLKCNKFVLMFSYLYAIFLISFVALVYWTTEPKKNSHLVIRLFTFFEYTLLACISVFLKKKKMIKFFENLSLLDKMLKINKNVNSTCCMKQVFFWVTGSIVYNLIEFYAMEFYDNTNKGLKTIICTYAIALAHDCEQIFFFTLQRVVYLRLLVVKRHIQEYFKVDEDSSRKKPNKYEMLSNNVQLNLTALHEVYALLHNCAEKLNTVMSIPVLLMLFTSGLSTTILLKFFVRVIQLTDPSNPGSAIGVCMYLIVRCIKYTLLVVISCYYSSITATQVSLIRITIHDAINTVPLGKLQRRKVKAFYLMTKEYSFVYALAGVIKLNMSLPLSYISLCTTYLVIIIQFSKFLD

>BmorGR51

MAMGIRTILSKVKPLELPDVSENNFADDGLKIVQRFKFFIYIQVLTGINRLYLLKCNKFVMLFSYLYAIFLISFVASVYWTKEPMKNSHLVIRLFSFIEYILLICISVFLKKKKMMKFFENLSMFDQILKIDKNVNSTFCMKRVFFWVTGSIVYNLIEFYALEFYDNTSKGLMTIICTYTIALTHDCEQIFFFTLQRVVYLRLLVVKRHIQEHFKVDEDSNRKKPNKYEMLSKNVQLNLTALHEVYGLLHNCAEKLNKIMSIPVLLMLFTSGLTTTILLRILVRVIQLADPSNPGSAIGLCVYLIVRCIKYTLLVVISCYYSSITATQVSLIRITINDAINTIAFGKLQRRKVKAFYLMTKEYSFVYTLAGVIKLNMSLPLSYISLCTTYLVIIIQFSKFFD

>BmorGR52

MAGIRTISSKVKPLELPDVSENNFADDGLKIVQRFKFFIYIQVITGINRLYLLKCNKFVMLFSYLYAMFLISFVVLVYWTTEAMKNSNLVIRNFTCLEYILLICIAMFLKKKKMIKFFENLSCLDKMLKIDKNVNSTCCMKRVSFWVAGSIVYNLIEFYAIEFYDNTNKGLVTIICTYTFALAHDCEQIFFFTLQRVVYLRLLVVKRHIQEYFKVDEDSSRKKPNKYEMLSNNVQLNLTALHEVYALLHNCAEKLNTVMSIPVLLILFTSGLSTTILLKILVRVIQFTDPSNPGQQSECACICIASNTRCLSRAITQASLQLKFLLFVSRFMMPSTLFHWVSKLQRRKVKAFYLMTKEYSFVYTLAGVIKLNMSLPLSYISLCTTYLVIIIQFSKFLD

>BmorGR53

MAHIKDENQSKQQQKEHETLNKNKLKKVVYTLKPALMLENWFGLSDFLLVNEDELVLLMQTEKFGVILSIFFIVMFAVFVDFPDTETESIMELMDEVPSMVVLSQYFIASITTSSCLSAIAIRIFETFADLDSMLLITTTQDFYNKSRYQTNKYLIILGVSHIISSTLDLLTDDEIVWCKFFVLPIYFLQKLEVLTFCKLIVMIQCRLQIINKYLTNFIEEQEKNKALVFTLAESNPKKTDKFNWIGCPSPNNMKIRDLATMYDVIGTICSLINDLFNIQIFMTLVSTFTYIVIAIWSTLYFYRAPNFTFGTLTTIIIWCITIILSVVVMSFVCERLVSVRNNTKILVNKVIMNYDLPKTMRVQAKAFMELIESWPLKIMVYDMFSVDISLMLKFISVATTYLIVIIQLSHFV

>BmorGR54

MTHAALPRSEAYFLMTLSRSTIVSKRAYGPPDGEWLPSPMDFSNARGRAKPLPTVCLRVCVQNNLSFYRPILIILQLCGYDFDYYNINLVLNVLTKAYCASLTCVVVYATIACCSSIQLSHIWSLIEYGTSVVIIACFRSQTKLFLKQLTTLDVYLRISNRRFVLEKCKIFTITSVIFLLRIVYTSIYCSTHHCFNVLIYFLLSQFALVCLDVNRIWRCIVFDAIRYRLKTLRLRMEENPDCNYYLYVKNNKSIRKNKISFCLFLYRTIADLVDLVSPELNVSRSTTNICGLKTHIIRGAPKTADTSCTRYSSLPEKNQQCISILFLSVACSLPKIVSNAYHLLLIIEDREPLETGGYVLMHTLQVSLLLFTPFIIVECYTMEVEKIKLYLVHRLIDENDTTMRDNIRLFLEYMSVRTFRYRIFRIVPVNATLPLELVNLCVNYVIVLINFTHLYG

>BmorGR55

MERINLLKSFAFLENVMCIYRNFMFYNQRARFIIIGRIVAELVFYIFSAYNGFLLVYTDWFSQNFSVFFIEIISKSSFYVITFFTMVNGILKSREYKTFIFSINKIHDYILNDTDYLKRLKCTNIFCTATIIILFVVTLIRTAIDGSNYGQLSGINARSVIWMLTTILLECQYQTECVVYFGFILFIHAIMKYLNIRVTNTIIKIARSDMAVKRIPKYIIGRTELKDETDTGVDVNNVVDLEEVRYWVFIYRQLGLTTELLQKCFGMQTAFIFVTAVLNQIITVFRVIAVFIYGSLANRGAEHSIIANFLFTLLYRLPGLLMIIVGGQMVQNQTDMLRRSMARLNNIISNNPHRETFSALSDFHRMIVKNPVKIYVLSVLPVGAYMLPLFMTLLINHIIILLQFNHVA

>BmorGR56

MKKIRLLRSIVFLENLLCIYRNFLFFNKKARAIILIHITIELVLYVLSIVNNSFIIYSYFHSDNRSMLIVFTTICCFYVVTFVSIVMGILRSEEFKDLVTSLELINKFFTNNKTYLKSLGRSNTMIIAITTILYCVTCIGIAVDKITLNDFYEFTSSDVIWTVSSTLLELRYQTECVVYFGIEYLFLIFTKHLNLLVKEAIKKVSLDNNGTVKDVPISSDAVTKNEVKRWATIYRQLMMSSKLLQACFSLQIICVFVSAVINFITTAFRMVKVSVLGSIATDMNEIIIVNLIFTLLYQNIGLVLIIVTGQRVWNQILLLNVLLARLYNGILIQPCRDTLRTLKNLQRMVVKNPVQIKMLSVLPVGSYMLPMFMTLSVSYIIVMLQFGHVV

>BmorGR57

MEEIKAIKLVTFIENCICVYRNYAMCTKRNKKIISLRIIVEIIIVFFVNINNILLLHKYYNGSGLLYIIYLFLVVYYINYMFCIFYGALQGKAYRQLIFCFNKINAIAKRDKSYKKSLARLKNMCIVISIALLIISALSVFVDRSNSWNIYEVSLRDSLLILSKIHMDFFYHFEYVVYFTHIKIFHLTLRYLNSRVKMAQFEMKMTRRDVHDEGERNIRILLTKELTTEWAVLYKCLVFGTKTMKSLFGLQMLIAMVMSFVNFTLSLYGIILICSIEQSQTASQHNLLLILTYYTATMLLIFIVAQSVYNEVEMLKRNLARMYNILAVDSDETQQKLVKDFLRMVYKNKVEIKMLSIFPVGMPMLTFFLSLSASYVVVMVQFSNVF

>BmorGR58

MSSRRVLYRAEVLLSNNVDAHVQDMLKPLNFFQFILFFPKYTIRDGYITPNSLIRNIWSATGAFVFISICVFRILTMNKIAVYDTFTTMLLISKYFDVALYCIGFIVNTYVNIAYSNVNVLLYLKLQTIKTFIPRNNEIMKNVKWYSVILIIVLFCGTLAMFSFFHLSFSYFNIFDLTTDLAVFSFDLNLVYACSVLNFLAQSLDELNKEIWRLGNAKVTVCKDGSKPDWNGINLTYINVLDAYNYFKEAFRLLIFFHTFKTLTHMFIYIQSIIELCKKFYPGDDYDAITVGAVVGVWFFRNITLQCLVGVSCQNFYSATSNTESICAVQVGSIVSDEHKLFLKAVRRLNNVVFYKWSMYGMFIVDATLPRRLIELIATYTVVFLQFAFK

>BmorGR59

MPYKKDSNRCEVLLYNNVDTDLQDMLRPLNFIQTIYLSPKYTIKDGYITPNSLFCNILSAAGAIVFFSICVYRILTASKIGTFEGFSTTLLITKYFDAILFSLGFVANAYVSIRLSHLNVLLYLKLQAIKTFVPCKKIMQKVKYYSIVLIIGLIIVRLIMYIHFHWSLGYLSYLDLITDLGVISFDLNLVYASSIVKFLGYNLEELNKEILRLDEIKATMDEEGSKPDWNGIRRTYLKFSEAYNYFKDAFRILILFHTLNTFAHVFIYVQSVIELCKAPADNYMGGRAHSPPGVKWLLEPLDIYNSAAFSVLIAVIVWLLRNIILQSLIGISCQSFYSATSNTQSICSILVRSVLSGEYNQWFLLKKYDVETHFFIAYVVGRAHSPSGVKWLLEPIDIYNVNSTTHLEI

>BmorGR60

MLTPRSDLCNEKLSPSFPSGKTTAADKDDTEARCQVDSSLERLLLPFNLVQHVSFIPMYSIRRGLVSPDGPLAYLYSLLGFCLFTSVSVYRNAIMHGTRLSSLHLFTLYSDLVSFVINYSLSLICNVVNSKSNVEFVCRLQRLQTVLRRNQREQEQFARSNWAHLAVVTALYLAVVGLLNVVVLKQSLPDTLYLLLLFCIDVNVLYATRMLALLRCYLQLWTRKINEKAFNPVHHNMFTAYLDILQEYEVYTTLFKKIITYYVLETFLHGLLYVQVAIQICKSIRRSGRFSEQLMMIVSIFTWTIKNMIIMTLHNVECEKFYLAVEQAVAACQTQRASTTRCREEKRLYKNVCRVSRAAFSRERGWGLLAAGAALTLRFMDLATTYVTVLLQFAFVSRT

>BmorGR61

MSIRFEKDLLHNYVEIELQYFLRPFNVMQSLFFQSKYRIVDNFILPNTLFKNIMSFVVSVLCALSFIYTIISVWQNTHATSFHALVTSVYLSYNIYGILIGSVLIIWLSDRNIEFVLKIQDLIKILEFNKCFLIEYAFINSIIMAAIFILNFLLYGYFVVHLQKFALGLTFSAIVCILNQDLDIIYVIIFANILKKCASRWTVEARQKNNFNDQGKWVKLFNAFLNLTESYQLYQKIFEFYELLRRVGIVFLGLQLTVCRVCSNDIKSIQCTVMLHAFQLICVWIVKKFITLSILSFEMEIFYEKLREIETVCIILVSSDNPSERELKIWKNIIRVSSCSVRKTTACGLCEVGAALPQWLLQATTAYTIVLLQFHITTFSRATNDIYDLD

>BmorGR62

MNDLFLSKIVKWTKTTKYKLDDDFQSLFRVFNIAQAMNLCPKFLIYDKYITNNAWFIHILAISSFIVLVCLDSFFANFRLVLSEAMGPPFYGFSFYFISILYENIGVIIQITMNGYLTKNNVLIITKLQDTFKDFRTTDYITKSNRWTNWFIFFIYMNFIANYSYFNFYVNTFSFHKFCFAFIKMCFDLNIVYTIFIFKMIGDSLTMFKDTAFCSKNMKLYEVSNRVYWNKMLRLYSNILDVFELSKRTLNFFIFYFVSNILLRILSHVQLAILMNSINWLQHVAYSNIVMVLLTLAKEGIILIVLIAKCEKIYCVIGDVQTACQLALGNAACPEKRRFCKNVRRSSSAAFSKIYICNILAVDAKLAVSLMSVTTTYTIVMLQAILIK

>BmorGR63

MQIGNAVIHLKSTKLTTMNTISPTTKLLKIFALNSNIEEIDLKCSTKLRITMTAFVLCSLIFYSLYYKFIYVFDYVNISIKITDCVQMVYDFCQYIVDLYFVTNYGRNISSEYFQQYKIIDKILEVVCYEIIKHRIVKLLWVFMCIWFSSSCFDFIAWFLNYGWITPLVYSVAYIFLLIKILTTLDLSAHIMNVEIRLKMIADLIHHYYMSCEDNFQAEETLCHKNWLNSKERAKYYELQFRIHALKQLSCNNNEIKLLSRCYLMLTEQVEIINRMYGFRILLNSLSLLIDMVRFTNISVRIMIGSQNLAYNCGYFPAVSSIFRLLTCGAVIINLVSHCERVYYQRTRICNVIDHMIVNKNLSRESTEALQEFRNLVQNHPIEFNMANFFQLNYSLLVSIASVVVTYTIILLQSVN

>BmorGR64

MKISLRKIVSIRNMTLIQNMFGFYHKFTDNRAIGVLLKIFCGFYSLFLSFLCINCTPRFTNDFLTYDIFFFVIEYLTSVLVCLLYDGQYFLNYLYDLKLIDREAGIEESLEKLPISQPLFSLIFITRVIYLLSCLLMFDGIKDSLFLPAQSSVFGANFTEFARTIGYFPRVIMFEMFYKRVNYLKSQLRNDLAHANLYPIGFVCSKVIMKYINFYKLLLRNLQQNSLQFKILMSMSSLYIIIKALASAYAFIYREDGVHVFIFIEFATGVFLFFVMSSIIISIFNEIEDIRQIVLAQLRYCKQGANTKRVQDALTILNIRCFKYALCRIYTVDFTFILRILDVSVTYVIVLVQFTHILD

>BmorGR65

MKISLRKIVSIRNMTLMQNMFGFYHKFTDNRSIGVLLKIFCGFYSLFLSFLCINCIPRFTNDFLTYNIFFYVIEYLTSVLVCLLYDGQYFLNYLYDLKLIDREAGIEESLEKLPISQPLFSLIFITRVIYLLSCLLMFDGIKDSLFLPAQSSVFGANFTEFARTIGYFPRVIMFEMFYKRVNYLKSQLRNDLAHANLYPIGFVCSKVIMKYINFYKLLLRNLQQNSLQFKILMSMSALYIIIKALATAYAFIYCEDGIHVFIFIEFAIGVFLFFVMSSIIISIFNEIEDIRQIVLAQLRYCKQGANTKRVQDALTILNIRSFKYALCRIYTVDFTFILRILDVSVTYVIVLVQFTHVLD

>BmorGR67

MRERKKKFNKLLNTRNYNNIVEALLPSDSIRKISGVSVVYLAVNSENRIVTKFSFIGTIFFLFWYILYFYCTYKAHSEDQTILRTIYNTKLKRYGDDFERIASIIYVTYSMWKVPFRMSGNQVFIQRIVDIDSAIENMGEAVDYNKNAKTALVISIAQLGDFLVRMFCIWLSLENLSVIVPTEKLYQVVYTDALSFVITSHYCFSLIVLRGRYKYINKVLSEIKTRSAWEYKVFVRNKVAPDLEKVQRLQDRIVCEKIKACARIYSMLYKATEAINRMYGTALVLTMLLYLVFIILYMFYFMEATASGLLYDIKKYVDFLICVFWQMSHALSIIYANVYFSESITREVCKF

>BmorGR66

MKRKLKKFFPNKEYNNIVEATHLWKLIRKLTGLSVLTLESKEGNRIETRFSSLGFVFFLLWFTIYFYCTYKAHNEDQTILRNIYSTKLQRYGDDFERITSIIYVLYSMWKLPFQISGNRLLLQEIVDIDKAIESVGVTIDYKKNATFALFIYIGQIATYLFRLFCVWGCLGNLNSPVPVEKLYQDIFTDALSLLLTSQYCFSLVILRDRCRYINKILCGIENRESSRLRLFVYSSMPGAEKDITCRKIKDCSKIYGMIYKAVESTNITYGFALVLTMLLYLIFIILYMFYFMEATAAGLFLDTKKYIDFLICVLSELLHAMLIIFLNIYFSEETVKETRTTSFVIHGIINSDFNTQAKTEAIHFSTQLLHQIPKFTASGLVELNYSLLYEVGGGILVVTPMGSGNNLATDGPRVCSPI

>BmorGR68

MRFGLKAGAAVVTILRPYNLCLKNIFKPFYVMLSLLGLFPYSIRFLGGKQFLIKPKSIYTNAVCALSLMLSMTLFLIFHIDHIIYKSTEDNSLTEGFMTQVNYIIEMLNLEIFCVVYYFSSFLNRNKFVKVLNTVAVWSDRISISGIKTLSFLRLKIHFSIGILMFLLISQVCVNFTRVDSLWKKVLVMFTFNIPQMIQFTAILFYYILVNMVITLLVIIQENISISTRDTKTSSFIRVEHRMPLSLKQLELIYIKAFELKRDINKAFEAPILLTTMQCFHSIVSESHIIYHGAVMEPHMVLHSIMNCSVWILYQLFKLYILASTGHLLQEKIQHFSNLIHFHGKGLTVYGLFPLDGTLMFKVVASAAMYLIILVQFDKRN

>SfruGR1

MNKEHGFRVYNPNPVNKETRKREMFQRIDEKDGIKEYDAKDLYGPEITDKDGALLDAHDSFYITTKSLLVLFQIMGVMPIMRVPKHAQTTKRTTFNWISKATLWAYLVWGLECIIVVKVGRERLANFQNSSNKRFDEVIYNIIFLSILIPHFLLPIASWRHGPQVAIFKNMWTHYQLKYLKITGTPIVFPNLYSLTWGLCVFSWGLSFAVILSQHYLQDDFELWHSFAYYHIIAMLDGFCSLWYINCNAFGTASRGLAMNLHKALGAEHPALKVAQYRHLWVDLSHMMQQLGRAYSNMYGIYCMVIFFTTTISLYGALSEILEHGLSYKEMGLFVIVGYCMTLLYIICNEAYHASRKVGLEFQVRLLNVNLGAIDRSTQREVEMFLVAISKNPPIMNLDGFTNINRELFTANVSFMSTYLIVLMQFKLTLLRQSARKTLKSIVKAVFNTSTTMLDDDFDEEGEE

>SfruGR2

MTIPDHLFDEGINNTLFDRDMRHIQQNKTVYEKTQRDYEQEQRDLLSSQDGDTCEIHDQFYRDHKLLLVLFRALAVMPITRSRPGTITFSWKSRATMYAVCFYIAATAVVLIVGYERIMILRSIRRFDDYIYAILFVIFLVPHFWIPFVGWGVAHQVAIYKTNWGKFQVRYYRVTGENLQFPNLKTTIVIISVGCLLLAVCFLLSLCILMDGFLLIHTSAYYHIITMINMNCALWYINCKGIKIASQSLSECFRRDVEQECSAQLISRYRYLWLNLSELLQSLGNAYARTYSTYCLFMFANITIAVYGALSEIVDHGFGFSFKEMGLFVDAAYCSTLFFIFVDCSHKSTLTVAAGVQDTLLSIDVLAVDRPTQKEIDHFIQAIEMNPAVVSLKGYAHVNRELLTSAISMIAIYLIVLLQFKISLPKDPQTVGT

>SfruGR3

MSFYTSNSLFPTTVPIPNGFPVQIDEKPKNKIIFLDATPVRTPVKPISPNAVAPMRNNLVEPHISNDIIYENIKPVFTVLRIMGVLPITRPASCINQFQIASASMLYSVLVFCSLVSYVLYLSLHKVQILRTAEGKFEEAVIEYLFTVYLFPMIAVPILWYETRKIADVLNGWVEFEVVYKQLSGRTLPVKLYKKALAIAIIIPILSTTTVIVTHITMVHFKPMQLVPYVFLEILTYMLGGYWYLLCETLSICANILAEDFQSALRHIGPAGRVAEYRALWLRLSKLSRDTGIANCYTFTFVNLYLFLIITLSIYGLLSQISEGFGIKDIGLALTAIYGIFLLFFICDEAHYASHNVRTNFQKKLLMVELSWMNTDAQTEVNMFLRATEMNPSQISLGGFFNVNRTLFKSLLATMVTYLVVLLQFQISIPDESQSQDDEEDAPLNITSATTEALTTTTTVMTTILTTLAKKKKKN

>SfruGR4

MKRNHVKKLNEHKYDDFLPILNNVFRKARYFGISGYGFNLFFAWSLILFTMLVIAETVAVWKVVRYLGGWLMSASENGLIGRMSGAIFYANALISLFLSSKFVHSWRNLYIYWLSTETNTALKFPPDVRMKKRAVIIIIFVTSVASLEHILSMISATGVGFPIGEFIYRYVTLSHEFLLRAQDYSPWNGIPIFILSKLATVLWNFQDLIIILISIGLSSRYKRLNSYVRQMVTVEKRTETKPKFGTELYLQIQVWRRLREAYVRQSTLVRMVDRKLGSLVLLSNINNLYFICLQIYLGIHKPSASTISRCYFLFSLSWLILRACSVVIAASDVHLHSQSALKLLHSCPSANYNIEMKRLQYQLSHDFVALTGMGFFSLRRGLLLEVAAAILKYELVLIQYDK

>SfruGR5

MRNAWSTAIGNISVGSVNTVNSLFKSWRKIAPTYTMDLYSLEKFKKYKKDWSYPVNIRYQEPVIQEKEKPCSTFQSAMKATLTIGQFFGLNPVQGIRGNDPAKLRFRLLSWRCAFTAISLMGQFTMAFVLFLSLFKEAASTVDTATTLLFYGFGFTTTILFFRIAIKWPQLSMVIARGEAADPNTDAKLSRKFNIACAVILSLAFVEHGFSELHGMSIALDCHPEKPLYETFMRGSFAWLFLYIPYNDFVGFLAHFFNIQSTFNWNFTDVFVICMSTYLTARLEQVNQRIIAAKDKNLPSSFWRTMREDYNRTVHLVRQVDKMIGGVVFISFASNLFFVCSQLLHTLAGGIKASPKCRPDLPDDRRFFGGYEHSIYFVYSFLFLVVRSLAVSLTASKVHAASIEPAHALYDVSSANYCVEVERFLDQIHGDTVALSGLQFFHVKRGLILTIAGTIVTYELVLMQFTGVTPSSPDPG

>SfruGR6

MSRVFFVSQNKVDIVKHKSPRSITPFFDALRCTLIVGQVFSLLPFVGVFTNIASNVKFIKTSWKCVYSLLSLIGQMFMAVLCINKLARSNVTLNGTSPVIFYVTTCVTMLMFFQVARRWPQLVQHIAKAEDMDPNFDCGLAKKCNITCAVVLILALLEHILSLLSAFAGAAACYGNMDSYQGFVTHFYPWVFNYLPYSLVLGIITQFLHFQSTFIWNFSDLFVICMSYYLTSRLEQVNKKLLAAQGKYLPEVFWRATREDYCRATQIVRKVDDVISGVVFISFANNLFFICLQLFNTLEDGLKGTGECSNKGKKIVVSKNGPLGGHEAATYFLFSLVYLLSRSVAVSLIASQVNSASAVPAPVLYDVPSPVYCVEVQRFLDQVNGDKVALSGLQFFSVTKGLLLTVAGTIVTYELVMFQFNSSTPTLNITAPTVLTPSTLSLTTLSS

>SfruGR7

MSKANYGQFLRNTNLILPKQPNHDEFLIAMENVFRWSCLIGVLGAKKWICYVWSAFILIVLLVMESQAIWKVIKALGGWAIDTAGQRSVTARLAGTIFYTIAIISLILASKLFRSWGQLSAVWVRVERVMAVKVPSDGTLKRRMYLTIGFMTVCALLEHLLSIVSAIGLDCPPNLIMKRYILISHGFMILRHEYSDWFPIPLAFMSTIATMLWNFQDQLIVLISMGLTSRYSRLNQCLAKICALEKKQMDSDQKTEATKVYTWRKLREAYVKQAMLVRKVDDAIGGIIILSCFCNFYFICLQLFLGITQSKSSEPLRTVYYFTSLGWLCFRVIIVVLAASNINVHSQIALNHIYTHDTLYYNVEMGRLQDQLSKDYVALSGKGFFYLSKSILLQMAGAIITYELVLIQFDDKGTDDVQLNLTKNGIGVD

>SfruGR8

MSGQDFKQFLGNSKLLLPEQPVHDDFLDAMEKVFHWSCIFGIFGSKRYLSILWSALILVSLVVIEYLAIWKVIRALTGVARDTSGHRSITARLAGTIFYSISIMSLFVASKLWYYWRSSISLLWAKVERSVGVKVPTDNTLKRRMYLVFGLMTFCSMFEHAVSMVASVGLDCPPSLIMKRYVLVSHGFIFMGQDYSEWFAMPLIIISTLASLLWNFQDQLIVLISIGLTSRYYRLNGYVAKLCELEKHQTEFNKKSEALKVYTWRKIREAYVKQAMLVRRMDEALGGIIMLSCSCNFYFICLQMFLGITQGLSTDIMTGIYYVVSLAWLCIRVISVVLSASGVNTHSKTALKYLYTYETQCYNVEVERLQDQLTKDYIALSGMGFFYLNKTILLQMAGAIITYELILIQFDGQGQIDAKTTPSIELNVTNI

>SfruGR9

MIRSNWISEHNYSLRLSPIARVIYGVESAKVEEVTAAPVPTESEARSNRPTHCVVGGAHAFILRISSFFGLAPLRFEARSNGFTVSISSAMCVYSYILVTVLVICTIFGLVAEINVGVELSVRMSSRMSQVVSTCDVLVVVATAGAGVYGAPQRMRNMLKFMENIASVDTSIGGQYSLVTERKLCGIILAILIFFSILIADDFTFYALQAKKLDREWDVVTNYLGFYLLWFVVLILELQFAFTALSVRARFSAVNDALALTARQVSVPVEKPKSSSPLNIYAIRVAPADSARSANVSLLVETMTGREHVVIIKRTASGEPRLIVSPCDAVRRLAALHGTLCDVVNSIDDSYGLPLIVILISTLLHLIVTPYFLIMEIIVSTNRIHFLVLQFLWCVTHMLRMIVVVEPGHYTIAEGKRTEALVCRLMTSAPSTGVLPSRLEIFSRQLMLQSVSYAPMGMCTLHRPLVASVIGAVTTYLVILIQFQRYDS

**IRs**

>DmelGluRIIC

MWQRILLLGCMWSAFFMCRSRGQQINIGAFFYDDELELEKEFMTVVNAINGPESEQTMRFYPLIKRLKPEDGSVTMQEHACDLIDNGVAAIFGPSSKAASDIVALVCNSTGIPHIEFDISDEGIQAEKPNHQMTLNLYPAQAILSKAYADIVQNFGWRKFTIVYDADDARAAARLQDLLQLREVHNDVVRVRKFHKDDDFRVMWKSIRGERRVVLDCEPNMLVELLNSSTEFGLTGQYNHIFLTNLETYTDHLEELAADNETFAVNITAARLLVNPDPPPYSLPYGYVTQRDNIVYESSDPPRTLIHDLIHDALQLFAQSWRNASFFYPDRMVVPRITCDFAASGGRTWAMGRYLARLMKGTSGVNNTNFRTSILQFDEDGQRITFNIEVYDPLDGIGIAIWDPRGQITQLNVDVKAQKKMIYRVATRIGPPYFSYNETARELNLTGNALYQGYAVDLIDAIARHVGFEYVFVPVADQQYGKLDKETKQWNGIIGEIINNDAHMGICDLTITQARKTAVDFTVPFMQLGVSILAYKSPHVEKTLDAYLAPFGGEVWIWILISVFVMTFLKTIVARISKMDWENPHPCNRDPEVLENQWRIHNTGWLTVASIMTAGCDILPRSPQVRMFEATWWIFAIIIANSYTANLAAFLTSSKMEGSIANLKDLSAQKKVKFGTIYGGSTYNLLADSNETVYRLAFNLMNNDDPSAYTKDNLEGVDRVRKNRGDYMFLMETTTLEYHREQNCDLRSVGEKFGEKHYAIAVPFGAEYRSNLSVAILKLSERGELYDLKQKWWKNPNASCFEEPDPDATPDMTFEELRGIFYTLYAGILIAFLIGITEFLVYVQQVALEERLTFKDAFKKEIRFVLCVWNNRKPIVAGTPISSVRTTPRRSLDKSLDRTPKSSRRVVIGRSSEEMREMAQGSGSSSGSNNAGRGEKEARV

>DmelGluRIIA

MRLCPVVIYAFIIIIGFLEGIIALGGDDRNEITVGAIFYENEKEIELSFDQAFREVNNMKFSELRFVTIKRYMPTNDSFLLQQITCELISNGVAAIFGPSSKAASDIVAQIANATGIPHIEYDLKLEATRQEQLNHQMSINVAPSLSVLSRAYFEIIKSNYEWRTFTLIYETPEGLARLQDLMNIQALNSDYVKLRNLADYADDYRILWKETDETFHEQRIILDCEPKTLKELLKVSIDFKLQGPFRNWFLTHLDTHNSGLRDIYNEDFKANITSVRLKVVDANPFERKKTRLTKVDQILGNQTMLPILIYDAVVLFASSARNVIAAMQPFHPPNRHCGSSSPWMLGAFIVNEMKTISEDDVEPHFKTENMKLDEYGQRIHFNLEIYKPTVNEPMMVWTPDNGIKKRLLNLELESAGTTQDFSEQRKVYTVVTHYEEPYFMMKEDHENFRGREKYEGYAVDLISKLSELMEFDYEFMIVNGNGKYNPETKQWDGIIRKLIDHHAQIGVCDLTITQMRRSVVDFTVPFMQLGISILHYKSPPEPKNQFAFLEPFAVEVWIYMIFAQLIMTLAFVFIARLSYREWLPPNPAIQDPDELENIWNVNNSTWLMVGSIMQQGCDILPRGPHMRILTGMWWFFALMMLSTYTANLAAFLTSNKWQSSIKSLQDLIEQDKVHFGSMRGGSTSLFFSESNDTDYQRAWNQMKDFNPSAFTSTNKEGVARVRKEKGGYAFLMETTSLTYNIERNCDLTQIGEQIGEKHYGLAVPLGSDYRTNLSVSILQLSERGELQKMKNKWWKNHNVTCDSYHEVDGDELSIIELGGVFLVLAGGVLIGVILGIFEFLWNVQNVAVEERVTPWQAFKAELIFALKFWVRKKPMRISSSSDKSSSRRSSGSRRSSKEKSRSKTVS

>DmelGluRIIB

MHGLQFLVLLALAIASGANEDTLVIKIGAIFFDTEMKLADAFSAALEEVNAINPALKLDAIKRYVTVDDSIVLQDISCDLIGSGVAAIFGPSSKTNSDIVEVLCNMTGIPHLQFDWHPQQSNRERMNHQLTVNVAPMELFLSAAFSDILASKTFDWKSFTIAYERSSHLIRLQHILAWKQLHKAGIKMQEFERGDDYRILWKRINNAREKFVLLDCPSDILVDVINASIGYNMTGSFNHLFLTNLDTHLSGIDGFYSRDFTVAVAAVRIRTYVPPPVHDEIDVFDNSVDTRFSSLGSQLVYDSIVLFYNALLEISQRPGFYIPNFSCGRGFWQPGPRLVEQMKQITPKMVKPPFKTQRLQINADGQREDFNLEVYNPIIDRVTHIWNKEFQLVDFEKLRENSTQALKQKRLQNKEDFSQKPIRYTVATRVGKPYFSWREEPEGVHYEGNERFEGYAVDLIYMLAQECKFDFNFEPVRDNKYGSYDANTDEWDGIIRQLIDNNAQIGICDLTITQARRSVVDFTVPFMQLGISILSYKEPPPKADIYAFLNPYNAEVWLFVMIAMMITAFALIFTGRIDQYEWDQPVENVNREMERQNIWHLSNALWLVLGSMLNQGCDLLPRGLPMRLLTAFWWIFALLISQTYIAKLAAFITSSKIAGDIGSLHDLVDQNKVQFGTIRGGATSVYFSESNDTDNRMAWNKMLSFKPDAFTKNNEEGVDRVKLSKGTYAFLMETTNLQYYVQRNCELTQIGESFGEKHYGIAVPLNADFRSNLSVGILRLSERGELFKLRNKWFNSNESTCDSNVPTIDDGQFDMDSVGGLFVVLIVGVVVGLVIGVAEFLWHVQRISVKEKIPPMLALKAEFYFVIRFWLTRKPLHTYRQSRDSTSTGYSSLEQITSASSAKKKKKTRRIEK

>DmelClumsy

MYSLFLTHFLLIALPVLADIDRSQFMVGSIFTSDKDESEIAFRTAVDRANILERNVELVPIVVYANTDDSFIMEKMVCNLISQGVIAIFGPSTGSSSDIIASICDTLDIPHIVYDWIPNESIPDREHSTMTLNVHPDNLLLSQGLAEIVQSFAWRSFTVVYETDKELQQLQDILQVGEPISNPTTVKQLGPGDDHRPFLKEIKLSTDNCLILHCAPDNLLKILQQANELKMLGEYQSVFIPLLDTHSIDFGELSGVEANITTVRLMDPSDFHVKNVVHDWEEREKREGRYFKVDPNRVKSQMILLNDAVWLFSKGLTELGIFEELTAPDLECRRKKPWPFGKRIIEFIKARSEETSTGRIDFNENGQRSFFTLRFMELNSDGFLDLATWDPVNGLDVLNDDEESEKRVGQKLSNKTFIVSSRLGAPFLTLREPQEGEILTGNSRYEGYSIDLINEIAKMLNFKFEFRMSPDGKYGALNKVTQTWDGIVRQLIDGNADLGICDLTMTSSRRQAVDFTPPFMTLGISILFSKPPTPPTDLFSFLSPFSLDVWIYMGSAYLFISLLLFALARMAPDDWENPHPCKEPEEVENIWSIMNTTWLSIGSLMGQGCDILPKAASTRLVTGMWWFFALMMLNSYTANLAAFLTNSRQANSINSAEDLAAQSKIKYGAMAGGSTMGFFRDSNFSTYQKMWTAMESASPSVFTKTNDEGVERVQKGKNLYAFLMESTTLEYNVERKCDLVQIGGWLDYKSYGIAMPFNSPYRKQISAAVLKLGELGQLAELKRKWWKEMHGGGNCEKSDEDGGDTPELGLENVGGVFLVLGLGLLSAMVLGCTEFLWNVKSVAIEEKISLKEAFKSEALFAARIWITTKPVHTSSESGSSNSSSSSSSRSKHSFKSQGLSMKSLKSSGYQDVEASVHSKLKKIGSMFSLKSQKTVTPPPEIGWKLDKSTQIDVVPTSDVDQELIPEVEPHLPHRHHHHHHHRHHHHHHQPDQEHDRNPSPPE

>DmelGluRIID

MHFCWISLIILSLSRVQAQFYGGNAYEASSGQSIRLGLITDDATDRIRQTFEHAISVVNNELGVPLVGETEQVAYGNSVQAFAQLCRLMQSGVGAVFGPAARHTASHLLNACDSKDIPFIYPHLSWGSNPDGFNLHPSPEDIANALYDIVNQFEWSRFIFCYESAEYLKILDHLMTRYGIKGPVIKVMRYDLNLNGNYKSVLRRIRKSEDSRIVVVGSTTGVAELLRQAQQVGIMNEDYTYIIGNLNLHTFDLEEYKYSEANITGIRMFSPDQEEVRDLMEKLHQELGESEPVNSGSTFITMEMALTYDAVRVIAETTKHLPYQPQMLNCSERHDNVQPDGSTFRNYMRSLEIKEKTITGRIYFEGNVRKGFTFDVIELQTSGLVKVGTWEEGKDFEFQRPPQAVNFNDIDDGSLVNKTFIVLISVATKPYASLVESIDTLIGNNQFQGYGVDLIKELADKLGFNFTFRDGGNDYGSFNKTTNSTSGMLKEIVEGRADLAITDLTITSEREEVIDFSIPFMNLGIAILYVKPQKAPPALFSFMDPFSSEVWLYLGIAYLGVSLCFFIIGRLSPIEWDNPYPCIEEPEELENQFTINNSLWFTTGALLQQGSEIAPKALSTRTISAIWWFFTLIMVSSYTANLAAFLTIENPTSPINSVKDLADNKDDVQYGAKRTGSTRNFFSTSEEPIYIKMNEYLNAHPEMLMENNQQGVDKVKSGTKYAFLMESTSIEFNTVRECNLTKVGDPLDEKGYGIAMVKNWPYRDKFNKALLELQEQGVLARLKNKWWNEVGAGVCSAKSDDDGPSELGVDNLSGIYVVLVIGSIISIIISILCWCYFVYKKAKNYEVPFCDALAEEFRIVIRFSENERPLKSAQSIYSRSRNSSQSIESLKTDSEENMPVED

>DmelGluRIIE

MFFNHFVILWSLFSIHISVNWAQYENFGGYDNYQSLESVPIGLLTDQNTEQMNIVFDHAIDVANQEVGTSLTSLKEEVNYGDAYQSYGKLCRMLETGIAGVFGPSSRHTAVHLMSICDAMDIPHIYSYMSENAEGFNLHPHPADLAKALYSLITEFNWTRFIFLYESAEYLNILNELTTMLGKSGTVITVLRYDMQLNGNYKQVLRRVRKSVDNRIVVVGSSETMPEFLNQAQQVGIINEDYKYIIGNLDFHSFDLEEYKYSEANITGLRLFSPEKMAVKELLMKLGYPTDQDEFRNGSCPITVEMALTYDAVQLFAQTLKNLPFKPMPQNCSQRTESVRDDGSSFKNYMRTLRLTDRLLTGPIYFEGNVRKGYHLDVIELQPSGIVKVGTWDEDRQYRPQRLAPTTAQFDSVDNSLANKTFIILLSVPNKPYAQLVETYKQLEGNSQYEGYGVDLIKELADKLGFNFTFVNGGNDYGSYNKSTNESTGMLREIMTGRADLAITDLTITSEREQALDFTIPFMNLGIAILYLKPQKATPELFTFMDPFSEEVWWFLGFSFLGVSLSFFILGRLSPSEWDNPYPCIEEPEELENQFTLGNSIWFTTGALLQQGSEIGPKALSTRTVASFWWFFTLIVVSSYTANLAAFLTIEKPQSLINSVDDLADNKDGVVYGAKKTGSTRNFFMTSAEERYKKMNKFMSENPQYLTEDNMEGVNRVKTNTHYAFLMESTSIEYNTKRECNLKKIGDALDEKGYGIAMRKDWPHRGKFNNALLELQEQGVLEKMKNKWWNEVGTGICATKEDAPDATPLDMNNLEGVFFVLLVGSCCALLYGIISWVLFVMKKAHHYRVPLRDALKEEFQFVIDFNNYVRVLKNSASIYSRSRQSSMSVASVAQESQ

>DmelCG3822

MRSSGVLVLPLLLLQLILNCRKAQSLPDIIKIGGLFHPADDHQELAFRQAVDRINADRSILPRSKLVAQIERISPFDSFHAGKRVCGLLNIGVAAIFGPQSSHTASHVQSICDNMEIPHLENRWDYRLRRESCLVNLYPHPNTLSKAYVDIVRHWGWKTFTIIYENNDGIVRLQELLKAHGMTPFPITVRQLSDSGDYRPLLKQIKNSAEAHIVLDCSTERIHEVLKQAQQIGMMSDYHSYLVTSLDLHTVNLDEFRYGGTNITGFRLINEKIVSDVVRQWSIDEKGLLRSANLTTVRSETALMYDAVHLFAKALHDLDTSQQIDIHPISCDGQSTWQHGFSLINYMKIVEMKGLTNVIKFDHQGFRTDFMLDIVELTPAGIRKIGTWNSTLPDGINFTRTFSQKQQEIEANLKNKTLVVTTILSNPYCMRKESAIPLSGNDQFEGYAVDLIHEISKSLGFNYKIQLVPDGSYGSLNKLTGEWNGMIRELLEQRADLAIADLTITFEREQAVDFTTPFMNLGVSILYRKPIKQPPNLFSFLSPLSLDVWIYMATAYLGVSVLLFILAKFTPYEWPAYTDAHGEKVESQFTLLNCMWFAIGSLMQQGCDFLPKALSTRMVAGIWWFFTLIMISSYTANLAAFLTVERMDSPIESAEDLAKQTRIKYGALKGGSTAAFFRDSKISTYQRMWSFMESARPSVFTASNGEGVERVAKGKGSYAFLMESTSIEYVTERNCELTQVGGMLDTKSYGIATPPNSPYRTAINSVILKLQEEGKLHILKTKWWKEKRGGGKCRVETSKSSSAANELGLANVGGVFVVLMGGMGVACVIAVCEFVWKSRKVAVEERLSAILNE

>DmelCG5621

MISTEASFPLGFILTSLLLAFPGCRGERTNVGLVYENTDPDLEKIFHLAISKANEENEDLQLHGVSVSIEPGNSFETSKKLCKMLRQNLVAVFGPTSNLAARHAMSICDAKELPFLDTRWDFGAQLPTINLHPHPATLGVALRDMVVALGWESFTIIYESGEYLPTVRELLQMYGTAGPTVTVRRYELDLNGNYRNVLRRIRNADDFSFVVVGSMATLPEFFKQAQQVGLVTSDYRYIIGNLDWHTMDLEPYQHAGTNITGLRLVSPDSEQVQEVAKALYESEEPFQNVSCPLTNSMALVYDGVQLLAETYKHVNFRPVALSCNDDSAWDKGYTLVNYMKSLTLNGLTGPIRFDYEGLRTDFKLEVIELAVSGMQKIGQWSGEDGFQENRPAPAHSLEPDMRSLVNKSFVVITAISEPYGMLKETSEKLEGNDQFEGFGIELIDELSKKLGFSYTWRLQEDNKYGGIDPKTGEWNGMLREIIDSRADMGITDLTMTSERESGVDFTIPFMSLGIGILFRKPMKEPPKLFSFMSPFSGEVWLWLGLAYMGVSISMFVLGRLSPAEWDNPYPCIEEPTELENQFSFANCLWFSIGALLQQGSELAPKAYSTRAVAASWWFFTLILVSSYTANLAAFLTVESLVTPINDADDLSKNKGGVNYGAKIGGATFNFFKESNYPTYQRMYEFMRDNPQYMTNTNQEGVDRVENSNYAFLMESTTIEYITERRCTLTQVGALLDEKGYGIAMRKNWPYRDTLSQAVLEMQEQGLLTKMKTKWWQEKRGGGACSDADEDSGAVALEISNLGGVFLVMGVGSFFGIFVSLLEMVLGVKERSDENQEAPDSDASSLGFANLGGVYLVMFVGSCFGSIYGLVNCVVSVYLRARENKVSFKTELLDEIRFILQCSGNTKAVKYPKNSSRSNASSKSKGSSMSVDSLPEDTSEADASGKHNHGKK

>DmelCG9935

MLIASGFLLFQFLSYGLGVPPLVRIGAIFSNQPGMYNSELAFRYAIHRLNMDKSLLPETTVDYYVEYVNRFDSFETVQKVCKLIRVGVQAVFSPTDSVLATHINSICDALDIPNIGRSAHDFSINVYPSKQLVNYAFNDVIQYLNWTRFGILHEKENGIINLHQLSRSFHGEVHMRQVSRDSYVSALNEFKGKEIHNIIIDTNSNGISILLKNILQQQMNEYKYHYLFTSFDLETYDLEDFKYNFVNITSFRLVDTADVGVKQILKDIGLYSHHIFKKPYLNLHIKKSTILESEPALMFDSVYVFAIGLQTLEQSHSLTLLNISCEEENSWDGGLSLINYLNAVEWKGLTGPIQFKDGQRVQFKLDLIKLKQHSIVKVGEWTPHGHLNITEPSMFFDAGSMNVTLVVITILETPYVMMHYGKNFTGNERFYGFCVDILETISREVGFDYILDLVPDRKYGAKDPETGEWNGMVAQLMKYKADLAVGSMTITYARESVIDFTKPFMNLGISILFKVPTSEPTRLFSFMNPLAIEIWIYVLIAYFLVSLCIYIVGKLSPIEWKCINACDLENISIGNQFSLTDSFWFTIGTFMQQSPDIYPRAMSTRIISSTWGFFSLIIVASYTANLAAFLTTERMINPIENAEDLASQTEISYGTLDSGSTMTFFRDSVIETYKKIWRSMDNKKPSAFTTTYEDGIKRVNQGNYAFLMESTMLDYIVQRDCNLTQIGGLLDTKGYGIATPKGSPWRDKISLAILELQERGDIQMLYDKWWKNTDETCTRKNTSKQSKANSLGLESIGGVFVVLIAGIIVAAVVAFFEFWYNFRYNYEATPSQSVVNNKYNQDGILESERNYTPPDRSFWIEIAEELRYASWCMNKQKRPALTRTCSKCTIPKGQRINKL

>DmelCG11155

MVRKKREIVIKENIQGRSYLKKICCSYIILSILVISNALPPVIRVGAIFTEDERESSIESAFKYAIYRINKEKTLLPNTQLVYDIEYVPRDDSFRTTKKVCSQLEAGVQAIFGPTDALLASHVQSICEAYDIPHIEGRIDLEYNSKEFSINLYPSHTLLTLAYRDIMVYLNWTKVAIIYEEDYGLFNLMHSSTETKAEMYIRQASPDSYRQVLRAIRQKEIYKIIVDTNPSHIKSFFRSILQLQMNDHRYHYMFTTFDLETYDLEDFRYNSVNITAFRLVDVDSKRYLEVINQMQKLQHNGLDTINGSPYIQTESALMFDSVYAFANGLHFLNLDNHQNFYIKNLSCTSDQTWNDGISLYNQINAAITDGLTGTVQFVEGRRNIFKLDILKLKQEKIQKVGYWHPDDGVNISDPTAFYDSNIANITLVVMTREERPYVMVKEDKNLTGNLRFEGFCIDLLKAIATQVGFQYKIELVPDNMYGVYIPETNSWNGIVQELMERRADLAVASMTINYARESVIDFTKPFMNLGIGILFKVPTSQPTRLFSFMNPLAIEIWLYVLAAYILVSFALFVMARFSPYEWKNPHPCYKETDIVENQFSISNSFWFITGTFLRQGSGLNPKATSTRIVGGCWFFFCLIIISSYTANLAAFLTVERMISPIESASDLAEQTEISYGTLEGGSTMTFFRDSKIGIYQKMWRYMENRKTAVFVKTYEDGIKRVMEGSYAFLMESTMLDYAVQRDCNLTQIGGLLDSKGYGIATPKGSPWRDKISLAILELQEKGIIQILYDKWWKNTGDVCNRDDKSKESKANALGVENIGGVFVVLLCGLALAVVVAIFEFCWNSRKNLNTENQSLCSEMAEELRFAMHCHGSKSRHRPRKRSCLNCSSVPTYVPSNVSTSNVGVYYNYFN

>DmelGlu-R1

MHSRLKFLAYLHFICASSIFWPEFSSAQQQQQTVSLTEKIPLGAIFEQGTDDVQSAFKYAMLNHNLNVSSRRFELQAYVDVINTADAFKLSRLICNQFSRGVYSMLGAVSPDSFDTLHSYSNTFQMPFVTPWFPEKVLAPSSGLLDFAISMRPDYHQAIIDTIQYYGWQSIIYLYDSHDGLLRLQQIYQELKPGNETFRVQMVKRIANVTMAIEFLHTLEDLGRFSKKRIVLDCPAEMAKEIIVQHVRDIKLGRRTYHYLLSGLVMDNHWPSDVVEFGAINITGFRIVDSNRRAVRDFHDSRKRLEPSGQSQSQNAGGPNSLPAISAQAALMYDAVFVLVEAFNRILRKKPDQFRSNHLQRRSHGGSSSSSATGTNESSALLDCNTSKGWVTPWEQGEKISRVLRKVEIDGLSGEIRFDEDGRRINYTLHVVEMSVNSTLQQVAEWRDDAGLLPLHSHNYASSSRSASASTGDYDRNHTYIVSSLLEEPYLSLKQYTYGESLVGNDRFEGYCKDLADMLAAQLGIKYEIRLVQDGNYGAENQYAPGGWDGMVGELIRKEADIAISAMTITAERERVIDFSKPFMTLGISIMIKKPVKQTPGVFSFLNPLSQEIWISVILSYVGVSFVLYFVTRFPPYEWRIVRRPQADSTAQQPPGIIGGATLSEPQAHVPPVPPNEFTMLNSFWYSLAAFMQQGCDITPPSIAGRIAAAVWWFFTIILISSYTANLAAFLTVERMVAPIKTPEDLTMQTDVNYGTLLYGSTWEFFRRSQIGLHNKMWEYMNANQHHSVHTYDEGIRRVRQSKGKYALLVESPKNEYVNARPPCDTMKVGRNIDTKGFGVATPIGSPLRKRLNEAVLTLKENGELLRIRNKWWFDKTECNLDQETSTPNELSLSNVAGIYYILIGGLLLAVIVAIMEFFCRNKTPQLKSPGSNGSAGGVPGMLASSTYQRDSLSDAIMHSQAKLAMQASSEYDERLVGVELASNVRYQYSM

>DmelGlu-R1B

MRFGLKLSCLWPSFLLWLTWSSGGGGGSGVGVSAQPSLTEKIPLGAIFEQGTDEVQSAFKYAMLNHNLNVSSRRFELQAYVDVINTADAFKLSRLICNQFSRGVYSMLGAVSPDSFDTLHSYSNTFQMPFVTPWFPEKVLTPSSGFLDFALSMRPDYHQAIIDTIQFYGWRKIIYLYDSHDGLLRLQQIYQGLRPGNESFQVELVKRISNVSMAIEFLHTLEQIGRFENKHIVLDCPTEMAKQILIQHVRDLRLGRRTYHYLLSGLVMDDRWESEIIEFGAINITGFRIVDTNRRLVREFYDSWKRLDPQMSVGAGRESISAQAALMYDAVFVLVEAFNKILRKKPDQFRNNVQRRSQTLMVAQAAASTSSDGYNYSASGGGGGNGGAGGGFAGSDSGGSGGMASRALDCNTAKGWVNAWEHGDKISRYLRKVEIEGLTGDIKFNDDGRRVNYTLHVVEMTVNSAMVKVAEWNDDAGLQPLNAKYVRLRPHVEFEKNRTYIVTTVLEEPYIMLKQVAFGEKLHGNNRFEGYCKDLADLLAKELGINYELRLVKDGNYGSEKSSAHGGWDGMVGELVRKEADIAIAAMTITAERERVIDFSKPFMSLGISIMIKKPVKQTPGVFSFMNPLSQEIWVSVIFSYIGVSIVLFFVSRFSPHEWRLVQQQPQQSQSPDPHAHHEQLANQQPPGIIGGAPLPAPPGPPTPGAQTAAGAAALQAALSAGSPGSGGSSSAVVNEFSVWNSFWFSLAAFMQQGCDLSPRSVSGRIAAASWFFFTLILISSYTANLAAFLTVERMVTPINSPEDLAMQTEVQYGTLLHGSTWDFFRRSQIGLHNKMWEYMNSRKHVFVPTYDEGIKRVRNSKGKYALLVESPKNEYVNAREPCDTMKVGRNLDTKGFGIATPLGSALKDPINLAVLTLKENGELIKLRNKWWYEKAECSTHKDGETSHSELSLSNVAGIFYILIGGLLVSVFVAILEYCFRSRDSRSASSGSGMGLGMGLGGGMSGGSLGKANGSMMLGPSSAVPGGMPSSHQRSTLTDTMHAKAKLTIQASRDYDNGRVGYLNCASLQYYPPAQLSATPPDAGDSLHMNAHGQV

>DmelNmdar2

MMPSRVKLKRGTDGPTPTPTPMPTTMRKHTPIATLNTASCQHNSTTSRRKRILTPPSGPISLLLLTVLTLLILDTRSCQGLRLTNGGGSLSKGAAANKEQLNIGLIAPHTNFGKREYLRSINNAVTGLTKTRGAKLTFLKDYSFEQKNIHFDMMSLTPSPTAILSTLCKEFLRVNVSAILYMMNNEQFGHSTASAQYFLQLAGYLGIPVISWNADNSGLERRASQSTLQLQLAPSIEHQSAAMLSILERYKWHQFSVVTSQIAGHDDFVQAVRERVAEMQEHFKFTILNSIVVTRTSDLMELVNSEARVMLLYATQTEAITILRAAEEMKLTGENYVWVVSQSVIEKKDAHSQFPVGMLGVHFDTSSAALMNEISNAIKIYSYGVEAYLTDPANRDRRLTTQSLSCEDEGRGRWDNGEIFFKYLRNVSIEGDLNKPNIEFTADGDLRSAELKIMNLRPSANNKNLVWEEIGVWKSWETQKLDIRDIAWPGNSHAPPQGVPEKFHLKITFLEEAPYINLSPADPVSGKCLMDRGVLCRVAADHEMAADIDVGQAHRNESFYQCCSGFCIDLLEKFAEELGFTYELVRVEDGKWGTLENGKWNGLIADLVNRKTDMVLTSLMINTEREAVVDFSEPFMETGIAIVVAKRTGIISPTAFLEPFDTASWMLVGIVAIQAATFMIFLFEWLSPSGYDMKLYLQNTNVTPYRFSLFRTYWLVWAVLFQAAVHVDSPRGFTSRFMTNVWALFAVVFLAIYTANLAAFMITREEFHEFSGLNDSRLVHPFSHKPSFKFGTIPYSHTDSTIHKYFNVMHNYMRQYNKTSVADGVAAVLNGNLDSFIYDGTVLDYLVAQDEDCRLMTVGSWYAMTGYGLAFSRNSKYVQMFNKRLLEFRANGDLERLRRYWMTGTCRPGKQEHKSSDPLALEQFLSAFLLLMAGILLAALLLLLEHVYFKYIRKRLAKKDGGHCCALISLSMGKSLTFRGAVFEATEILKKHRCNDPICDTHLWKVKHELDMSRLRVRQLEKVMDKHGIKAPQLRLASSSDLLNHHHLKERPPLLGNLSLAASAQDLYRWSYKTEIAEMETVL

>DmelNmdar1

MAMAEFVFCRPLFGLAIVLLVAPIDAAQRHTASDNPSTYNIGGVLSNSDSEEHFSTTIKHLNFDQQYVPRKVTYYDKTIRMDKNPIKTVFNVCDKLIENRVYAVVVSHEQTSGDLSPAAVSYTSGFYSIPVIGISSRDAAFSDKNIHVSFLRTVPPYYHQADVWLEMLSHFAYTKVIIIHSSDTDGRAILGRFQTTSQTYYDDVDVRATVELIVEFEPKLESFTEHLIDMKTAQSRVYLMYASTEDAQVIFRDAGEYNMTGEGHVWIVTEQALFSNNTPDGVLGLQLEHAHSDKGHIRDSVYVLASAIKEMISNETIAEAPKDCGDSAVNWESGKRLFQYLKSRNITGETGQVAFDDNGDRIYAGYDVINIREQQKKHVVGKFSYDSMRAKMRMRINDSEIIWPGKQRRKPEGIMIPTHLRLLTIEEKPFVYVRRMGDDEFRCEPDERPCPLFNNSDATANEFCCRGYCIDLLIELSKRINFTYDLALSPDGQFGHYILRNNTGAMTLRKEWTGLIGELVNERADMIVAPLTINPERAEYIEFSKPFKYQGITILEKKPSRSSTLVSFLQPFSNTLWILVMVSVHVVALVLYLLDRFSPFGRFKLSHSDSNEEKALNLSSAVWFAWGVLLNSGIGEGTPRSFSARVLGMVWAGFAMIIVASYTANLAAFLVLERPKTKLSGINDARLRNTMENLTCATVKGSSVDMYFRRQVELSNMYRTMEANNYATAEQAIQDVKKGKLMAFIWDSSRLEYEASKDCELVTAGELFGRSGYGIGLQKGSPWTDAVTLAILEFHESGFMEKLDKQWIFHGHVQQNCELFEKTPNTLGLKNMAGVFILVGVGIAGGVGLIIIEVIYKKHQVKKQKRLDIARHAADKWRGTIEKRKTIRASLAMQRQYNVGLNSTHAPGTISLAVDKRRYPRLGQRLGPERAWPGDAADVLRIRRPYELGNPGQSPKVMAANQPGMPMPMLGKTRPQQSVLPPRYSPGYTSDVSHLVV

>DmelIR8a

MELPLLVLLLALRFAGSEVLKITFWIEPVQRAEFDTDIAMVLKELDALRLDVKVDDTTLTLTRSEDGLDMQRFCEILSTVGASAVIDLTYSHWEEGYNLVRSLGIGYVRLERIMRPFLDMFGDFMRQKRANNVAMVFMNARDAVEAMQQMLVGYPFRTLIMDASQTDPGQHFLERIRSLRPAPTYIALFARAAAMNGIFEKVQKADLFQRPLEWHFVFLDTRDRVFKYRRQAELCTRFTLNPRAICRSMPMPDLYCGSGFTMQRAMLLNVLRSLINAAQVSPGYPLAIYQDCNATASSSEVSDPLEKDDYNWLDMVHWSNFLAYAPPLPHIQDQFQSPVPGLTFAVNISAGYYSSEHEAKTDLAAWSSVGEMRLLNETISPARRFFRIGTAESIPWSYLRREEGTGELIRDRSGLPIWEGYCIDFIIRLSQKLNFEFEIVAPEVGHMGELNELGEWDGVVGDLVRGETDFAIAALKMYSEREEVIDFLPPYYEQTGISIAIRKPVRRTSLFKFMTVLRLEVWLSIVAALVGTAIMIWFMDKYSPYSSRNNRQAYPYACREFTLRESFWFALTSFTPQGGGEAPKAISGRMLVAAYWLFVVLMLATFTANLAAFLTVERMQTPVQSLEQLARQSRINYTVVKDSDTHQYFVNMKFAEDTLYRMWKELALNASKDFKKFRIWDYPIKEQYGHILLAINSSQPVADAKEGFANVDAHENADYAFIHDSAEIKYEITRNCNLTEVGEVFAEQPYAVAVQQGSHLGDELSYAILELQKDRFFEELKAKYWNQSNLPNCPLSEDQEGITLESLGGVFIATLFGLVLAMMTLGMEVLYYKKKQNALEITQVRPVNDSSGSGGNSSTAPPTATSTTKQAWHIPVLEAEEKPAKVSPPPSFETATFRGKKLPARITLGDGKFKPRHGLYARRNLGASDSHSGYME

>DmelIR25a

MILMNPKTSKILWLLGFLSLLSSFSLEIAAQTTQNINVLFINEVDNEPAAKAVEVVLTYLKKNIRYGLSVQLDSIEANKSDAKVLLEAICNKYATSIEKKQTPHLILDTTKSGIASETVKSFTQALGLPTISASYGQQGDLRQWRDLDEAKQKYLLQVMPPADIIPEAIRSIVIHMNITNAAILYDDSFVMDHKYKSLLQNIQTRHVITAIAKDGKREREEQIEKLRNLDINNFFILGTLQSIRMVLESVKPAYFERNFAWHAITQNEGEISSQRDNATIMFMKPMAYTQYRDRLGLLRTTYNLNEEPQLSSAFYFDLALRSFLTIKEMLQSGAWPKDMEYLNCDDFQGGNTPQRNLDLRDYFTKITEPTSYGTFDLVTQSTQPFNGHSFMKFEMDINVLQIRGGSSVNSKSIGKWISGLNSELIVKDEEQMKNLTADTVYRIFTVVQAPFIMRDETAPKGYKGYCIDLINEIAAIVHFDYTIQEVEDGKFGNMDENGQWNGIVKKLMDKQADIGLGSMSVMAEREIVIDFTVPYYDLVGITIMMQRPSSPSSLFKFLTVLETNVWLCILAAYFFTSFLMWIFDRWSPYSYQNNREKYKDDEEKREFNLKECLWFCMTSLTPQGGGEAPKNLSGRLVAATWWLFGFIIIASYTANLAAFLTVSRLDTPVESLDDLAKQYKILYAPLNGSSAMTYFERMSNIEQMFYEIWKDLSLNDSLTAVERSKLAVWDYPVSDKYTKMWQAMQEAKLPATLDEAVARVRNSTAATGFAFLGDATDIRYLQLTNCDLQVVGEEFSRKPYAIAVQQGSHLKDQFNNAILTLLNKRQLEKLKEKWWKNDEALAKCDKPEDQSDGISIQNIGGVFIVIFVGIGMACITLVFEYWWYRYRKNPRIIDVAEANAERSNAADHPGKLVDGVILGHSGEKFEKSKAALRPRFNQYPATFKPRF

>DmelIR21a

MSYYWVALVLFTAQAFSIEGDRSASYQEKCISRRLINHYQLNKEIFGVGMCDGNNENEFRQKRRIVPTFQGNPRPRGELLASKFHVNSYNFEQTNSLVGLVNKIAQEYLNKCPPVIYYDSFVEKSDGLILENLFKTIPITFYHGEINADYEAKNKRFTSHIDCNCKSYILFLSDPLMTRKILGPQTESRVVLVSRSTQWRLRDFLSSELSSNIVNLLVIGESLMADPMRERPYVLYTHKLYADGLGSNTPVVLTSWIKGALSRPHINLFPSKFQFGFAGHRFQISAANQPPFIFRIRTLDSSGMGQLRWDGVEFRLLTMISKRLNFSIDITETPTRSNTRGVVDTIQEQIIERTVDIGMSGIYITQERLMDSAMSVGHSPDCAAFITLASKALPKYRAIMGPFQWPVWVALICVYLGGIFPIVFTDRLTLSHLMGNWGEVENMFWYVFGMFTNAFSFTGKYSWSNTRKNSTRLLIGAYWLFTIIITSCYTGSIIAFVTLPAFPDTVDSVLDLLGLFFRVGTLNNGGWETWFQNSTHIPTSRLYKKMEFVGSVDEGIGNVTQSFFWNYAFLGSKAQLEYLVQSNFSDENISRRSALHLSEECFALFQIGFLFPRESVYKIKIDSMILLAQQSGLIAKINNEVSWVMQRSSSGRLLQASSSNSLREIIQEERQLTTADTEGMFLLMALGYFLGATALVSEIVGGITNKCRQIIKRSRKSAASSWSSASSGSMLRTNAEQLSHDKRKANRREAAEVAQKMSFGMRELNLTRATLREIYGSYGAPETDHGQLDIVHTEFPNSSAKLNNIEDEESREALESLQRLDEFMDQMDNDGNPSSHTFRI

DN

>DmelIR31a

MNLLISMFILILAAGEGEIIPSMEESVVTNFVKSLVKTKQAIVFSCLFKDFKEISLALMRINQFVSVVNLNQSYSLTSILTRENYARTSVMVNARCSGSSELLFEASENRYFNKTYQWFLWGVDLEVQSLFPLNLNYVGPNAQITYVNETADGYAYWDIHSKGRHLKSNLEINLIATLINDTLNIARDIFHLQSIDFRGQFNGLTLRGASVIDKEDIISNEQIESILSRPTKDAGVAAFIKYHYELLGLLRERFNFTVNFRNSRGWAGRLGNTTFRLGLLGIVMRNEADIAASGAFNRINRFAEFDTIHQSWKFETAFLYRYTSDLDTHGKSGNFLSPFSDRVWLFCLLTLGAFSIIWVLFEIIDYKILRIRVNSQKLEHLNQKSSVICIKTTCIERILQTFGACCQQGLDPNPVDRSVRFLVMTLFLFSLVMYNYYTSSVVGGLLSSSDQGPSTVDEITASPLKISFEDIGYYKVLFRESQNRSITRLIEKKLSSSRSLNELPIFSHIEDAVPYLKAGGFAFHCEVVDAYPVISEYFDANEICDLREVSGLMEVEILNWILHKNSQYTEIFKTAMCNAQEKGFVERILRRRQIKKPACQSLYTVYPVSLSGVLPGFVILICKSINKFS

>DmelIR40a

MHKFLALGLLPYLLGLLNSTRLTFIGNDESDTAIALTQIVRGLQQSSLAILALPSLALSDGVCQKERNVYLDDFLQRLHRSNYKSVVFSQTELFFQHIEENLQGANECISLILDEPNQLLNSLHDRHLGHRLSLFIFYWGARWPPSSRVIRFREPLRVVVVTRPRKKAFRIYYNQARPCSDSQLQLVNWYDGDNLGLQRIPLLPTALSVYANFKGRTFRVPVFHSPPWFWVTYCNNSFEEDEEFNSLDSIEKRKVRVTGGRDHRLLMLLSKHMNFRFKYIEAPGRTQGSMRSEDGKDSNDSFTGGIGLLQSGQQADFFLGDVGLSWERRKAIEFSFFTLADSGAFATHAPRRLNEALAIMRPFKQDIWPHLILTIIFSGPIFYGIIALPYIWRRRWANSDVEHLGELYIHMTYLKEITPRLLKLKPRTVLSAHQMPHQLFQKCIWFTLRLFLKQSCNELHNGYRAKFLTIVYWIAATYVLADVYSAQLTSQFARPAREPPINTLQRLQAAMIHDGYRLYVEKESSSLEMLENGTELFRQLYALMRQQVINDPQGFFIDSVEAGIKLIAEGGEDKAVLGGRETLFFNVQQYGSNNFQLSQKLYTRYSAVAVQIGCPFLGSLNNVLMQLFESGILDKMTAAEYAKQYQEVEATRIYKGSVQAKNSEAYSRTESYDSTVISPLNLRMLQGAFIALGVGSLAAAALNNTINVRSLNSRDKFICGGPVKIWYYLVLLLWYYFNRGLVGIYQLWHKTSIRNTGKGMPFLGE

>DmelIR64a

MHWWLLVFLPLSCQGLPEHELLELELDYGLAEPQRTSLLQSSLILQFSQDYKHIPRITYFTCQKPHLQTPNQIPNAAEHRDAFAAKNFQLIKSLYESELFVRIVLLDVLAQSPTSGRPNRPGNGPTGGFSQTPSQAQSNSEWLEGVLRMEALRQIAVVDLACGAVSRRFLELASAKMLYSEKFHWLLIEDFAWHGRTQTAEGSGKRDDGEMEEEEPPGQQIQATDDEDLPSIESFLGGMNLYMNTELTLAKRMSEAAHYTLFDVWNPGLNYGGHVNLTEIGSFTPTEGIQLHTWFRTTSTVRRRMDMQHARVRCMVVVTNKNMTGTLMYYLTHTMSGHIDTMNRFNFNLLMAVRDMFNWTFVLSRTTSWGYVKNGRFDGMIGALIRNETDIGGAPIFYWLERHKWIDVAGRSWSSRPCFIFRHPRSTQKDRIVFLQPFTNDVWILIVGCGVLTVFILWFLTTIEWKLVPHDGSALIKPKGGAPPRHHYQQQQQQEQVEAPVRPITAVSVVVSKEKVEEKQEEYEDSTPIDAGTLWQRCYQKLNKYIKDRKAKQKKAPERVGLFLESVLFFVGIICQQGLGFSTSFVSGRCIVITSLLFSFCIYQFYSASIVGTLLMEKPKTIKTLSDLVHSSLKVGMEDILYNRDYFLHTKDPVSMELYAKKITSVPTTKENEADEDEPVDPNPVSTDPAKSYRDIVHSHETGAHAKDNAASNWLDPETGLLRVKHERFAFHVDVAAAYKIIAETFSEQDICDLTEVSMFPPQKTVSIMQKNSPMRKVISYGLRRVTETGILTYHFNVWHSRKPPCVKKIETSDLHVDMDTVSSALLILLFSYAITLMILGTEILYSKWHNRIQLKWVGAT

>DmelIR75a

MQLVQLANFVLDNLVQSRIGFIVLFHCWQSDESLKFAQQFMKPIHPILVYHQFVQMRGVLNWSHLELSYMGHTQPTLAIYVDIKCDQTQDLLEEASREQIYNQHYHWLLVGNQSKLEFYDLFGLFNISIDADVSYVKEQIQDNNDSVAYAVHDVYNNGKIIGGQLNVTGSHEMSCDPFVCRRTRHLSSLQKRSKYGNREQLTDVVLRVATVVTQRPLTLSDDELIRFLSQENDTHIDSLARFGFHLTLILRDLLHCKMKFIFSDSWSKSDVVGGSVGAVVDQTADLTATPSLATEGRLKYLSAIIETGFFRSVCIFRTPHNAGLRGDVFLQPFSPLVWYLFGGVLSLIGVLLWITFYMECKRMQKRWRLDYLPSLLSTFLISFGAACIQSSSLIPRSAGGRLIYFALFLISFIMYNYYTSVVVSSLLSSPVKSKIKTMRQLAESSLTVGLEPLPFTKSYLNYSRLPEIHLFIKRKIESQTQNPELWLPAEQGVLRVRDNPGYVYVFETSSGYAYVERYFTAQEICDLNEVLFRPEQLFYTHLHRNSTYKELFRLRFLRILETGVYRKQRSYWVHMKLHCVAQNFVITVGMEYVAPLLLMLICADILVVVILLVELAWKRFFTRHLTFHP

>DmelIR75b

MNFSVLESHFKEAQIFVDADVTYVTHDPFSKNFLLYDVYNKGRQLGGELNITADREIFCNKTNCRVERYLSELYTRSALQHRKSFTGLTMRATAVVTALPLNVSIKEIFDFMNSKYRIQLDTYARLGYQARQPLRDMLDCKFKYIFRDRWSDGNATGGMIGDLILDKADLAIAPFIYSFDRALFLQPITKFSVFREICMFRNPRSVSAGLSATEFLQPFSGGVWLTFALLLLLAGCLLWVTFILERRKQWKPSLLTSCLLSFGAGCIQGAWLTPRSMGGRMAFFALMVTSYLMYNYYTSIVVSKLLGQPIKSNIRTLQQLADSNLDVGIEPTVYTRIYVETSEEPDVRDLYRKKVLGSKRSPDKIWIPTEAGVLSVRDQEGFVYITGVATGYEFVRKHFLAHQICELNEIPLRDASHTHTVLAKRSPYAELIKLSELRMLETGVHFKHERSWMETKLHCYQHNHTVAVGLEYAAPLFIILLGAIILCMGILGLEVIWHRHCTLH

>DmelIR75c

MTSWPLYRLIVFNLLEINLSNLMVFHCWSIKEAFPLVEMLNQNGIFSQYIDVQNPDNLANVHKEYLDSDLVSLNADVTYVSREDEERFILHDVYNKGSHLGGKLNITVDQTLQCNRSHCQVKEYLSELHLRPRLQHRMDLSSVTFRLAALVSVLPINSSEEELLEFLNSDRDSHMDSISRIGNRLIMHTQEILGFNVQDAFGGAIGMLTNESAELCTTPFVPSWNRLHYLHPMTEQAQFRAVCMFRTPHNAGIKAAVFLEPFMPSVWFAFAGLLIFAGVLLWMIFHLERHWMQRCLDFIPSLLSSCLISFGAACIQGSYLMPKSAGGRLAFIAVMLTSFLMYNYYTSIVVSTLLGSPVRSNIRTIQQLADSSLDVGFDTVPFTKTYLVSSPRPDIRSLYKQKVESKRDPNSVWLSPEEGVIRVRDQPGFVYTSEASFMYHFVEKHYLPREISDLNEIILRPESAVYGMVHLNSTYRQLLTQLQVRMLETGITSKQSRFFSKTKLHTFSNSFVIQVGMEYAAPLFISLLVAYFLALLILILEICWARYAKKKFSTIIPQNQ

>DmelIR75d

MKVQVAHWLPLIFFLLVSGTPRVAGSWRSEYSRQDPDPKTRWGNQLPDMLVAYYRHHGVHSLMLVVCHTDIADFRLWKLWQHFNLNNFYVQVSTESSLRDLQHVDALDEHKDAPPPKSFHANNSTHWETSFLLPALPYKMGILLLEFSSECALNLLRWSAASEHNYFTTNRFWLLLTEDPGDIDLLEDPEIFIPPDSELRVLHYENVGNFSCSLIDLYKVAAWKPLKRTLVGHNIRNSRHVIHALQHFGSAITYRQDLEGIVFNSAIVIAFPDLFTNIEDLSLRHIDTISKVNHRLMLELANRLNMSYNTYQTVNYGWRQPNGSFDGLMGRFQRYELDLAQLAIFMRLDRIALVDFVAETYRVRAGIMFRQPPLSAVANIFAMPFENDVWVSILMLLIITTVVLVLELFFSPHNHDMSYMDTLNFVWGAMCQQGFYVEVRNRSARIIVFTTFVAALFLFTSFSANIVALLQSPSDAIQSLSDLGQSPLEIGVQDTQYNKIYFTESTDPVTKNLYHKKIASKGENIYMRPLLGMEKMRTGLFAYQVELQAGYQIVSDTFSEPEKCGLMELEPFQLPMLAIPTRKNFPYKELIRRQLRWQREVSLVNREERKWIPQKPKCEGGVGGFVSIGITECRYALGIFGCGAAVSFVLFLFEFIFRHFKQVYRIIKGYREVQR

>DmelIR76a

MENLLVESYYFSTVLSFFAQQFFADSHATCIFWHPAFDFRLETVHPMPLIIMDWHRWANRSDQDVYDYKIKEDEFEGKGIPYNDWTLRLTVAIERSHCETFIAFQEQIPEFARYFYHASIYSIWRSLRNRFMFVYTKEFEDKKDSYLSGYIFQDQPNILVITSQYLNSSTFEIKTNRFVGPRNFNKNPEPVEFYILQRFDAKGTKATWETQSAMSSKMRNLKGREVVIGIFDYKPFMLLDYEKPPLYYDRFMNTTDVTIDGTDIQLMLIFCELYNCTIQVDTSEPYDWGDIYLNASGYGLVGMILDRRNDYGVGGMYLWYEAYEYMDMTHFLGRSGVTCLVPAPNRLISWTLLLRPFQFVLWMCVMLCLLLESLALGITRRWEHSSVAAGNSWISSLRFGCISTLKLFVNQSTNYVTSSYALRTVLVASYMIDIILTTVYSGGLAAILTLPTLEEAADSRQRLFDHKLIWTGTSQAWITTIDERSADPVLLGLMEHYRVYDANLISAFSHTEQMGFVVERLQFGHLGNTELIENDALKRLKLMVDDIYFAFTVAFVPRLWPHLNAYNDFILAWHSSGFDKFWEWKIAAEYMNAHRQNRIVASEKTNLDIGPVKLGIDNFIGLILLWCFGMICSLLTFLGELWRGQG

>DmelIR76b

MATGIELLVAAALCVACPPLNDSPPTNLIQMGENGTLSPVTELPMDVDASEAGFDADAPVETLETINRKKPKLREMLDWIGGKHLRIATLEDFPLSYTEVLENGTRVGHGVSFQIIDFLKKKFNFTYEVVVPQDNIIGSPSDFDRSLIEMVNSSTVDLAAAFIPSLSDQRSFVYYSTTTLDEGEWIMVMQRPRESASGSGLLAPFEFWVWILILVSLLAVGPIIYALIILRNRLTGDGQQTPYSLGHCAWFVYGALMKQGSTLSPIADSTRLLFATWWIFITILTSFYTANLTAFLTLSKFTLPYNTVNDILTKNKHFVSMRGGGVEYAIRTTNESLSMLNRMIQNNYAVFSDETNDTYNLQNYVEKNGYVFVRDRPAINIMLYRDYLYRKTVSFSDEKVHCPFAMAKEPFLKKKRTFAYPIGSNLSQLFDPELLHLVESGIVKHLSKRNLPSAEICPQDLGGTERQLRNGDLMMTYYIMLAGFATALAVFSTELMFRYVNSRQEANKWARHGIGRTPNGQSVAPSRWLRGWRRLNSGHGQLLGASTHGQNVTPPPPYQSIFNGGSHGDPLNRWRRPLANGNALGNGVLLGGDSEGGVRRLINGRDYMVFRNPNGQSQLVPVRSPSAALFQYSYTE

>DmelIR84a

MIKLQVKVISWPLIILTAFLRVLQIESINTNFLELAAFEDFLRSEHLSHVLVVRGDDADGDWKIECHQKLLANYRVQFYRPEMSANFEDLMFYGSPRTAVLVLNSEHVLVRRQVFGVASEAGYFNNSLAWFILGSGRESLPVEQLIDQLLSGYRMGIDADITVALRGPDNASMLFYDVYRISRQANTPLIIEKKGLWTHSGGYQKFGNFKNTWVIRRRNFLNVTLIGSTVLTEKPPGFGDMEYLADDKQLQQLDPMQRKTYQLFQLVERMFNLSLAISLTDKWGELLDNGSWSGVMGQVTSREADFAVCPIRFVLDRQPYVQYSAVLHTQNIHFLFRHPRRSHIKNIFFEPLSNQVWWCVLALVTGSTILLLFHVRLERMLSNMENRFSFVWFTMLETYLQQGPANEIFRLFSTRLLISLSCIFSFMLMQFYGAFIVGSLLSESARSIVNLQALYDSNLAIGMENISYNFPIFTNTSNQLVRDVYVKKICKSGEHNIMSLQQGAERIIQGRFAFHTAIDRMYRLLLELQMDEAEFCDLQEVMFNLPYDSGSVMPKGSPWREHLAHALLHFRATGLLQYNDKKWMVRRPDCSLFKTSQAEVDLEHFAPALFALALAMVASALVFLLELFLHWLPDFRRRLGTMST

>DmelIR92a

MLLQPLVMHLSQLLRIIVGQYFAEFPSILIVYNNSASTTPLQLEYLSALELVLRELSKPIRLQWINVAFLKDLNDLEDQVMGALNSSVTEGFITILSQTHHFIHARYYATRNANVRLKDKRYLFLCEDESPAELLCMDILQFYPHHLMVRPGTETAPTGPTGPHPDPRRGGGASVSTKNKDDGEGGAGNKTTSPYRDINFELWTQKFVGAVGNLDALLLDAFLPNETFANRVELYPNKLLNLQRRSLLVGSITYVPYTITNYVPAGQGDVDPIHPQWPNRSLTFDGAEANVMKTFCQVHNCHLRVEAYGADNWGGIYDNESSDGMLGDIYEQRVEMAIGCIYNWYDGITETSHTIARSSVTILGPAPAPLPSWRTNIMPFNNRAWLVLISTLVICGTFLYFMKYVSYRLRYSGTQVKFHHSRKLEKSMLDIFALFIQQPSAPLSFDRFAPRFFLATILCATITLENIYSGQLKSMLTFPFYSAPVDTIEKWAQSGWKWSAPSIIWVHTVQSSDLETEQILARNFEVHDYSYLSNVSFMPNYGFGIERLSSGSLSVGDYVSTEALENRIVLHDDLYFDYTRAVSIRGWILMPELNKHIRTCQETGLYFHWELEFIDKYMDKKKQEVLMDLANGHKVKGAPQALDVRNIAGALFVLAFGVAFAGCALVAELLIHRMDLSK

>DmelIR93a

MNPGEMRPSACLLLLAGLQLSILVPTEANDFSSFLSANASLAVVVDHEYMTVHGENILAHFEKILSDVIRENLRNGGINVKYFSWNAVRLKKDFLAAITVTDCENTWNFYKNTQETSILLIAITDSDCPRLPLNRALMTVECRINAVVFVDQTILEENALLVKSIVHESITNHITPISLILYEINDSLRGQQKRVALRQALSQFAPKKHEEMRQQFLVISAFHEDIIEIAETLNMFHVGNQWMIFVLDMVARDFDAGTVTINLDEGANIAFALNETDPNCQDSLNCTISEISLALVNAISKITVEEESIYGEISDEEWEAIRFTKQEKQAEILEYMKEFLKTNAKCSSCARWRVETAITWGKSQENRKFRSTPQRDAKNRNFEFINIGYWTPVLGFVCQELAFPHIEHHFRNITMDILTVHNPPWQILTKNSNGVIVEHKGIVMEIVKELSRALNFSYYLHEASAWKEEDSLSTSAGGNESDELVGSMTFRIPYRVVEMVQGNQFFIAAVAATVEDPDQKPFNYTQPISVQKYSFITRKPDEVSRIYLFTAPFTVETWFCLMGIILLTAPTLYAINRLAPLKEMRIVGLSTVKSCFWYIFGALLQQGGMYLPTADSGRLVVGFWWIVVIVLVTTYCGNLVAFLTFPKFQPGVDYLNQLEDHKDIVQYGLRNGTFFERYVQSTTREDFKHYLERAKIYGSAQEEDIEAVKRGERINIDWRINLQLIVQRHFEREKECHFALGRESFVDEQIAMIVPAQSAYLHLVNRHIKSMFRMGFIERWHQMNLPSAGKCNGKSAQRQVTNHKVNMDDMQGCFLVLLLGFTLALLIVCGEFWYRRFRASRKRRQFTN

>DmelIR7a

MFHHLWLLMGLRSLAMGALHPPQPEAMTPLVAAALEILAEQVSPSQSTLAVMDLTQDAEHRDERQEQLMTIILRSVGSEMALRTFQKPPAEVPASFVVFLVNSAQAFNTLGFHFTDIHSTREFNFLILLTHRMSSRAERLQVLRDISRTCVRFHTSNVILLTEKRDGVVLVYAYRLLNMDCDLSVNLELIDIYKNGLFRHGHEARSFNRVLSLSGCPLQVSWYPLPPFVSFIGNSSDPEERAQIWRLTGIDGELIKLLASIFDFRILLEEPCNKCLSPDIKDDCSGCFDQVIISNSSILIGAMSGSHQHRSHFSFTSSYHQSSLVFIMHMSSQFGAVAQLAVPFTVIVWLALVVSSLLLVLVLWMRNRLVCGRSDLASHALQVLTTLMGNPLEARSLPRSSRLRILYAGWLLLVLVLRVVYQGKLFDSFRLPYHKPLPTEISELIRSNYTLINQEYLDYYPRELTVLTRNGSKDRFDYIQGLGKEGKFTTTSLIATMEYYNMMHWSTSRLTHIKEHIFLYQMVIYLRRHSLLKFAFDRKIKQLLSAGIIGYFVREFDACQYRKPFEEDYEVTPIPLDSFCGLYYISLIWLSAAVVAFILELLSQRIVWLRRIFE

>DmelIR7b

MKYWLYILSCCSLVASTMESSSDWDLAEALAQVVANSEMGRFKTLYIYTHTNSQSTGGHLEELLDQVLMIVPNNLQARRLLLQQSMEYKPYVHAVLALVDGLPSLSAIYARIRATQDLSHTLIYMSMPTDAYGEEMQATLRFLWRLSVLNVGVVLRPPGDHILMVSYFPFSALHGCQVISANVVNRYQVGTKRWASQDYFPSKLGNFYGCLLTCATWEDMPYLVWRPDGSGSFVGIEGALLQFMAENLNFTVGLYWMNKEEVLATFDESGRIFDEIFGHHADFSLGGFHFKPSAGSEIPYSQSTYYFMSHIMLVTNLQSAYSAYEKLSFPFTPLLWRAIGLVLILACLLLMLLVRWRHHHELPRNPYYELLVLTMGGNLEDRWVPQRFPSRLVLLTWLFATLVLRSGYQSGMYQLLRQDTQRNPPQTISEVLAQHFTIQLAEVNEARILASLPELRPEQLVYLEGSELQSFPALAQQSGSSARVAILTPYEYFGYFRKVHPMSRRLHLVRERIYTQQLAFYVRRHSHLVGVLNKQIQHAHTHGFLEHWTRQYVSAVDEKDESVARIASTSYSTLDGIDGDPSLSESEEDQQVAPVRQNVLSMRELAALFWLILWANLGAVVVFVLELLLPRIKLRKILRKMKKSTRASATTTSTLSSPSTTKDIPFSCKDGFQDSWPKCSLLVS

>DmelIR7c

MLHSAVHNVSLVYALVWAIDNYYGMATSTPLAVVQFPTSRESRRLHNDLIDAALGRSSGTGRIQFLLEDDRVEMTETDTDPPPPSGLTGRPIAIWFLDSLRSYFRLEMYLNQLGSPYKRNGFFLVIYTGLEDQPMESLKIMFRRLLNMYVLNVNVFLQRDGTVHLYTYYPYGPHHCQSSLPVYYTAFQDLAAPANGFGLTKPLFPRKLTNMHGCEMVVATFEHRPYVIIEDDPKTPGGRSIHGIEGLIFRSLAERMNFTIKLVEQKDKNRGEILPDGNFTGILKMMVDGEVNLTFVCFMYSKARSDLMLPSTSYTSFPIVLVVPSGGSISPMGRLTRPFRYIIWSCILVSLIFGFVLICLLKITALPGLRNLVLGRRNRLPFMGMWASLLGGLALYNPQRNFARYILVMWLLQTLILRAAYTGQLYLLLQDVEMRSPIKSLSEVLAKDYEFRILPALRTIFKDSMPTTNFHAVLSLEESLYRLRDEDDPGITVALLQPTVNQFDFRSGPNKRHLTVLPDPLMTAPLTFYMRPHSYFKRRIDRLIMAMMSSGIVARYRKMYMDRIKRVSKRRNLEPKPLSIWRLSGIFVCCAGLYLVALIVFILEILTTNHRRLRRAFNVINRYAA

>DmelIR7d

MDIRCVVALLLGLCKVQAVVWPHQHLLEEQLASQISATLQKIFINGLAVYNFGVFISTSYEEMDRDRVILVHQVLNRNLYPPNFPVAVVLASKMNRKITAQVFTQLLFVQNAEQAIAIAEGVNRNGLCVIVLLTSQPERPIMTKIFTYFMQERYNINVVILVPRLHGVQAFNVRPYTPTSCSSLEPVEIDIKDGDLWDVFPRRLKNLHGCPLSVIVWDIPPYMRINWKSSDPMDGLDGLDGLLLRIVARKMNFTLKLIPNEPNGLIGGSSFMNGTFTGAYKMLRERRANITIGCAACTPERSTFLEATSPYSQMSYIIVLQARGGYSIYEVMLFPFEKYTWLLLSTILGLHWIVGSRWRMPSPILAGWMLWIFVIRASYEASVFNFIQNSPVKPSPRTLDQALSGGFRFITDHASYRMTLKIPSFQGKTLISAGQPVDVFDALLKAPWKTGAFTSRAFLADHLVRHRKHRNQLVILAEKIVDNMLCMYFPHGSYFAWEINKLLFNMRSFGIFQHHSQILAWDNLPTTTDTDTPGKRIHSSTESVATGFAESMSFVVAALNCLMGALCISIVVFGLELLSRRRHWTGLEWLFERV

>DmelIR7e

MNHINEFVARAVLHVVHHYILSVTPSLVLTLCCRSNHTCNFYNKMMSTLFREWGLAPLQIVNVLRGVPWHPVPGRRHFNVIFTDSFAAFEEIRMEYYSREYNYNEHYFIFLQARDRLLQGEMRLIFDYCWRYRLIHCSIQVQKSNGDILFYSYYPFGEHGCSDMEPQLINRYNGSMLVEPDLFPRKLRNFFGCPLRCALWDVPPFLTLDEDQEEVLRVNGGYEGRLLLALAEKMNFTIAVRKVHVNMRDEALEMLRRDEVDLTLGGIRQTVARGMVATSSHNYHQTREVFGVLASSYELSSFDILFYPYRLQIWMGILGVVALSALIQLIVGRMLRERMGSRFWLNLELVFVGMPLLECPRSHTARLYCVMLMMYTLIIRTIYQGLLYHLIRTHQLNRWPQTIESLVQKNFTVVLTPIVQEVLDEIPSVQHMRFRLLEANSELDPLYFLEANHQLRQHVTASALDIFIHFNRLSADKVHQRGEQGSGAHFEIVPEDIISMQLTMYLAKHSFLIDQLNEEIMWMRSVGLLSVWSRWELSESYLRNEQSFQVLGTMELYAIFLMVLVGLIVGLLVFILELVSMRSIYLRKLFT

>DmelIR7f

MQGEDANLYVARALRLVIENVLAQLSTTLVVTISTRHLGTAHWFEYMMNILMDSWRMVAVQLLRIRPDLVVNPVPGRKRVSLLMVDSYQGLLDTNITASNANFDDPDYYFIFLQARDHLIPKELQLILDHCLAHFWLHCNVMIQTAQVEVLVYTYYPYTADACQKAYPIPVNTFDGRKWKASQMFPDKLSQMHGCPLTVLTWHQPPFVELVWDPKHNRSRGSGFEIQLVEHLARRMNFSLELVNIALLRPNAYRLAEGSSEGPIEKLLQRNVNISMGYFRKTARRNQLLTTPMSYYSANLVAVLQLERYRIGSLALLVFPFELSVWMLLLLALLIHLGIHLPSARRGNEEDGGGGLQVVALLLGAALARLPRSWRHRFIAAHWLWASIPLRISYQSLLFHLIRLQLYNTPSFSLDQLLAEGFQGICTANTQRLLLEMPQLARDPDSIQSVDTPFDWDVLNVLTRNRNRKIFAVANQDVTLSFLHSSAHPNAFHVVKQPVNVEYAGMYMPKHSFLYEKMDDDIRRLDASGFIHAWRRASFASVHRKEQVHMTSRRYINHAKLSGIYMVMAGLYLLAGLLFAGEVLLRQRN

>DmelIR7g

MNVTSLLNFESMKYIGAQTQAASINHHVAQALRVFIEDFYQRIAPAFIVVLSCRRPSPMNFYRNIMQLLYESVDTMIVQLVLVELGRPRRIAGPRTHNLLLVDSLDALLDIEIHTYTAQSDTSEYYFIFLQQRDALIPHDMQGVFAYCWRHQLINCNVMTQSSGGQVLLHTYFPYAPGQCNDSQPTRINMFLGESWKHRDYFPSKLHNLNGCPLIVLARKVSPFLDLDEGQRELRGLEGRLLQELSRRMNFSIQFSGLQDQLKNRTTWTEKQLLQKLVQERIAHLAIGYVRKRIQYATNLTPVFPHYSNRVVGCLLLNAHNLTSLEIWSFPFQALTWICLVAGDRLALVLAVYAASLGLPIDPPERPSLQLLFASWLIFGLIVRSMYSALLFFILRYHLHQRLPGNLQDLTHGDYAAVMGRTTLQDLREVPSLQDLLGLKSVIVTSEREEEVLRTLDRCTLREGAGSHPLFFGLISQDALLHLTQRGHRAGAYHIIPQDVLEQQLAIYLQKHSHLASHLDHLVMSIRSVGLVHHWAGQMASERYFRSRFLYREKRIRQPDLWAVYILTAGLYLLSLVVFICELLASRRAGL

>DmelIR10a

MAVLGTVFLLFMLDLKTLNLTRLNGLLVEPTRDLPQLELWLRAGSDHQDAENPYVQWFLLRTEIPLSIVTYQENRYWMDDPFGRRNLVLVMSLDQLLTNRGAAAPIQKASTFFYILADQDKDLSADEQLRLEGSCRQLWTQHKVYNRFFLTRDGVWIYDPFKRRDSAFGRLVRYYGSETLDKLLFRDMAGYPLRIQMFRSVYTRPEFDKETGLLTRVTGVDFLVAQMLRERLNFTMLLQQPEKKYFGERSANGSYNGAIGSIIKDGLDICLTGFFVKDYLVQQYMDFTVAVYDDELCIYVPKASRIPQSILPIFAVGYDIWLGFVLTAFACALIWLTLRVINLKLRIVSLGNQHIVGQALGIMVDTWVVWVRLNLSHLPASYAERMFIGTLCLVSVIFGAIFESSLATVYIHPLYYKDINTMQELDESGLKVVYKYSSMADDLFFSETSPXWNRDLRADVIDEVARFRNKAGVSRYTSLILESSHFTLLRKIWVVPECPKYYTISYVMPRDSPWEDAVNALLLRFLNAGLIVKWIQDEKSWVDIKMRSNILEADAESELVRVLTIGDLQLAFYVVIGGNLLAFLGFLAEHFRWKLQKKGV

>DmelIR11a

MRFAILWLFSGCLLPGIQVGIWVVVRAQPTGRDVLLSRLGNQQNELNTRRLANASSYLTRNYIANRINTLVVREICVECPYELSERQRQLVDQILASLAPELSVLLHKGTAEETTWEYTLFVVNDHTAFTGQVFIFPDELLEREFFCIVVVSEIQSRQFVRQTVGSIVKSNLQMHFVNVVVVAQLEDGTVGTYSYKLFKANCTPGITVRQINHFDRITGKPQQSMPDLYPVRNGHLGDCPFNVGAAHMPPHLIYKRHKDPPPASNVSIPAEDLAGIDWDLLQLLAKALKFRIQLYMPQEPSQIFGEGNVSGCFRQLADGTVSIAIGGLSGSDKRRSLFSKSTVYHQSNFVMVVRRDRYLGRLGPLILPFRGKLWGVIIVILLLAVLSTCWLRSRLGLSHPIEDLLTVIVGNPIPDHRLPGKGFLRYLLASWMLLTLVLRCAYQARLFDVLRLSRHRPLPKDLSGLIKDNYTMVANGYHDFYPLELTCRQPLDFSARFERVQRAAPDERLTTIALISNLAYWNHKHPNISRLTFVRQPIYMYHLVIYFPRRFFLRPAIDRKIKQLLSAGVMAHIERRYMQYENKRKVASNDPVLLRRITKSIMNGAYRIHGLVIVLATGMFILELLAGRSNGRLRRWMEWVHQ

>DmelIR20a

MLASLNRSTGLSAELLDLYGLVVHFLLSGEHTTLVYFNPAGLDCSWGVLWQRNLTAHPQIVWQRNYSYPDLYYQFNAKLLVLACLPMDSRAAIQLEILANSLSHLRTVVRLLIEVAGPDQVTLARQYLSFCLRRSMLHVELYFRDYHHSLILYSFRAFPSFELVMRWISVGQGVKLFLHKLDDLRGHRLRVIPDLSPPNTFFYRDARGDNQVTGYLWDFLATFAGRLNAGLEVVRPSWRAGSASDSSYMLEYSAKGLIDVGLTTTLITKWNLWAIHQYTYPLLVSSWCTMLPVEKPLATPDLFGRIVCPTLAMTLLLIILVTWLVFRQLRCLTRLKNSRPARIVPHLLTLLLLTTCSAQLLSLLIFPPYHVRIASFEDLLRGDQKILGMRNEFYNFDGAFRARYAGVFYLIDDPNELYDLRNHFNTTWAYTMPYIKWLVIKTQQRHFSKPLFRWSKDLCFFDFMPTSVIVAPDSIYWESIKDFTFRIHQAGLMKHWIRKSFYDMIKAGKMSIKDYSDLETLKPLNIGDLEIVWRVCGAAIAVASAIFIMELLYFYINVFFNSL

>DmelIR41a

MFIDLSWSLVLSAIVGKYLNESTICIFWNDKFEFQLLHKSDYISFVGINIKSFDDNGGHYIIDTGLKKKELQNKHLFLDELVIKIIISIEVTHCETFVVFDKDIDRFVNAFNKASVYSIWRSLHNKFVFAHIANESPESRNHFFEDQPNILFVVRDHSSASSFDIKTNKFVGRKAENPSQMILVDRYLASEQRFQFGKSLFADKLNNLQGREVIIAGFDYPPYTVIKHNMSTNAQDMGVSGESDFKNVYIDGTETRIVLNFCEQFNCTIQIDSSAANDWGKVYPNMSGDGALGMLINRKADICIGAMYSWYEDYTYLDLSMYLVRSGITCLVPAPLRLTSWYLPLEPFKETLWAAILLCLCAEATGLVLAYKSEQALYVLPGYREGWWTCTSFGVCTTFKLFISQSGNSKAYSLTVRVLLFACFLNDLIITSIYGGGLASILTIPSMDEAADTVTRLRFHRLQWAANSEAWVSAIRASDEALVKDILYNFHIYSDDELLRLAQDQHMRIGFTVERLPFGHFAIGNYLGPQAIDQLVIMKDDIYFQYTVAFVPRLWPLLDKLNTLIYSWHSSGFDKYWEYRVVADNLNLKIQQQVQETMTGTKDIGPVPLGMSNFAGFIIVWILGSAIATLTFLLELSLTYILKQSNLK

>DmelIR47a

MRQIKLLVWLLVVGVVSSTEQLQFLKNFLEAVHKERSISTILLIQRKVHKNDFLHGLYPIFWPIICLDETKRVELVNNFNKDFLALVYMESEADTLLLSALAADLNHIRDARIMIWLQMSPSENFLDRIVFQASKQKFLNLVVIENTLKTRRFYPFPQPKVQVIDKPFEEKEIYPALWRNFMGKNAIAVPDLVPPRSFNSFDPKTGHRRESGSIYNVFKAFTQRYNITMLLKWPLIRNTTQEEIIGKSVRGEIDLPITGQLISFRHPNGSRSQPLLGMTALSIAVPCGPELPMFDRFFLFYGLATPITITGYYVLLNTIEIILGTLSDRIKRHPRRKKILNLVLNLRVFSCILSLPTPQGNRLRSVKGQLTMVMSITGLILSCIVAAQTSTILTMKPQYRHIKNFQELSDSNITVVCNHLNYLTIKQQMDPKFMAKFMQNIWIVNSIEQMKMIFDLNTSYAYQTFSYKKDPFTLLQMHTTRKAFCRTPGLDLVSGLAYTAVLEKNSIYALALQDYTLKAFSAGLVYYWAEESIRDLISTVGRTQFEKLPIVIGYQSLKLQDYNVCWKILLIGGALAFCVFIVEVVVGLINRRI

>DmelIR47b

MREAQIIIFLLTSAAAVTLKQYEFLXSFLKAGEQEQTITTLLMMQKHVHTKNLLQGLYPXPWPIIHFVETQRIKFIALLYMSSEKDIFLSSLAANLKFERLDKPFGKSNIFPVLWRNYMGXIALTLDHLVEPRSFYWTDPRTNIKRRTGYIYMLITNFAEQHNITLQLXSPPNEDMSQMVIIERTHKGPRSTHNWADDQLETFERXQDSLLPWHGSMAIVVPCGQEMSAYERFHAAHAFRAPIIFFGFHIFLSLIDFLLRTISDRIRCNPRRIQLLQTVLSLCVLRCILSTSLPNSNXLRSRLRDNSPXXXVLQAXSYSALWXLTGTAXQXHNRDFQSHKLHDYXTTDGSXHSIEVPGLLKARNXXIXLFHIFSSLGTKFDLRIGSAGSHTSGVEFRYYELLDRXSSLENNIVSQVFTILKLPYSRFRVLKLEDCRGCWQTLFVGFSIATFVFIVNVLMGFFRNINQKK

>DmelIR48a

MHLLITETYMIIGKTLHDILNELNERLIISTNIIFCKQFDNLIHFEAQTSRFVYSSLEAFNITSLWNHVGNDNKLFVIVGNVPPYELFAKLELSSPENCTQFILNNTVDMCADALVKNSKAFSVSRELRIAPANVIVPHGKPLLSYRYLAAPFNTKVWIALGTYVFLISGFLCLIHWLRSGKWDFSQNLLEVYSSLLFTVFHLKATNGIERYILFGVLFISGFVYSTSYLRLLKSMLIAETFEKQIQTFEELAESNIPLLINPYDRMIFQHHHIPKSLWTAVRTVSSETLLNHRSHGYVRLCPAILTASKIPSHTHRHLFSVCRFSHEQEVVPKGSSXXSLVPCIRKRNREXNHLGCLSGVSWPGISXFFHYGALGGEAFGSILLHDANYFPSPRLFRRLAELHYGSY

>DmelIR48b

MILQQSSNLLKLLLLLAISSVRTQGLNDIIIELNQRLLISNNFLYCNQSDKLNEYEIKYLQHMPPISLMIFTSIESMNFTQVEYNLGADNKLFLIMGNEEPPYDFLHALNLHFQFAEYIIVIDEPVDLKKSTKWLDFVNHLWQQGYVQLLIYTSYDEKLYHKIIFPETVIEETLVEQYISIRGSFNNLYGYPVRVAAYNNAPRSMLYVNRWGKHIFAGFYMRFLRAFIDARNGSFVPVLTPSNSPGNCTLNLVNETVDVCADALAANPAAFSLTHGFRIASANVLVTHAKPLHSYRYLTAPFQWSVWACLVIYVLLVVNFLSFIGWLRSGKWEFSKYLLEVFSSLLFSGFYLKEIRGRERYILFGVLFIAGFVYSTEYLGLLKSMLISEVFEKQIDTFEALVESNITLMVDPYDKILFAKYNMPEILSPIMELVSFETLLKHRNRFDQDYAYILFSDRMALYDYAQQFLKHPKLLRIPIDFSFLYTGIPMRKRWFLKHHLGRAWYWAFESGLTRKLALDADFEAVRVGYLSFLITEHVEAQPLNVDYFVMPAIALAIGYILALLSFVIEMTAWRIREFLGCRKATMTSTGCSEGGHVDVD

>DmelIR48c

MSLLRIILIIIFLRIVSSIPDTIISHLSAELQIKIQIYFGLGNDLYDFSRLDGNYQKIIISHNISEEFKTYHDEPVLIIIRLERDLNLNLATLDVLRSYLTDRQYNDILLIDNDEENLNSYVDIRKAYWNAGFSQVLIYNSQQRTWSIKPYPYLQIRPTSLKEYIENRNTRNLMGYPLRVLVTNDPPHCFVDKDELPGSPNRYKGSIVTMLKIFADQLNATFQANPFREFRRYSTADCVQMVSDDEIDACGSIFIRTYTYATSQPVRLNRVVIMAPFGNPIEKFYYFFRPFDLYVWIGTGIIVVYIAVMGSLLHRWHFKEWNVGQYLLLAVQTLLNRELSLPQSSSGSKFMLLLLLFAIGFILSNLYVALLSMMLTTKLYQRPIENLADLKAANVNILLQTHNIRPNSVYGSSEELRERFLLVEESQHLEKRNGLDPSYAYVDSEDRMDFYLYQQKFLRRRRMKKLSNPVGYTWAVQVIKQNWVLEKHYNDHVQRFFETGLQNKLVDDVHELAVKAGFLHFFPTQTQTIEPLRLEDIVMAAMVLGGGHALAVICFLVELFA

>DmelIR51a

MYNVLVLFLLLFTRAQMEPHRRGHNMTLLRSVLTVIRGRENWKNTPIFLGGHCNSDDLNNLMSWLQNTMEVTCHTVDTSTSAKNENALGHFNINADNSLGLLFCQSSHELIWFNMDKRLRRLRGIRLIVILSDKRSSSSKAIMSTFKRLWHFQFQXNFQGYVVSTPVENDIPRVFFVKDKKTGRKQIRGFGYRTFVEYLHRYNASLHVSNSQQEHAINSSVNMGRIINQIVDGQLEISLHPYVDVPENMGDNSYPLLIASNCLIVPVRNEISRYMYLLLPLNQSSWILLLGSVIYISGVLYYIQPGLLHRTWDQRIGLNILDSISRIINICSPSRIYNPSLRYFIVSVHLSILGFVVTNLYSIMLGSFFTTLVVGEQVDSMQQLIQXQQKVLVKYYEVSTFLRHVEPDLVDGVAQLLVGVNASEQVSALLGFNRSYAYPFTLERWEFFSLQQQYAFKPIFRFSSACLGSPIIGYPMKSDCHLQSSLNMFIMRIQAAGLLRHWVVSDFNDAMRAGYVRLLENFLGFHSLDVDSLRLRWAVLLCGWLLSTLIFLCER

>DmelIR51b

MCKVLTLLVVILLLALTNAAYNVTLLKSVLSLISTREPWINTPIFVGHNTQGGDLNDLIIWLHQTMGVTSLTMNLFLQPEHIRPLGHFKITRYNGIALFFCHDKHDIMWLTLDRNLRKLRRIRLIIILRNQRSGSQGAIKSIFNALWQYQFLNVLVLQRDQLYSYTPYPAMRFFKLDIHTEPLFPHAARNFHGYVVSTPAENDIPRVFHVHDPLTKSRKVLGYAYRTFVEYLDHYNASLRLTNPDENLDPTTSVNMNHIVQLIIDGQLEISLHPYVFTPPTATKSYPLLIYPNCLIVPMRNEIPRHMYLLRPFQLYSWYILLFAVFYITGILYCISPKLNKSSWPQRLGLNFLDAISKILFISPPITIYRPTWRHLIIFLQLSVLGFMSTSWYNIELDSFFTTIVVGEQVNSMDQLVHQQQRVLVKEYEINTFLRHVEPRLVEKVSRLLVPVNASEQVSALLSFNRSFAYPFTEERWQFFAMQQQYAFKPIFRFSSACLGSPHIGYPMRVDSHLETSLNHFILKIQDTGLLNHWVVSDFNDAMRAGYVRFVDNVLGYQSIDVDTLRLGWCVLGIGWILSALVFSCEYWHLYPWRFIA

>DmelIR52a

MALGWSVIILGFIGQLSAQILNYTQSRDLELLEGSLFRVLSRLNLEEEYNTLLIYGKECVFHSLLRKLEISAVTVPSGSTDYDWSFSTAILILSCGYDAENEENSYTLMKLQRTRRLIYLEDNSEPESVCMRYSLKEQHNIAMVKSDFDQSDTFYSCRLFQTPNYVEGHFFKDQPIYIENFQNMRGATIRTVADSLVPRTILYRDEKSGETKMMGYLGHMINTYAQKLNAKLHFIDTSKLGAKKPSVLDIMNWVNEDIVDIGTALASSLQFKNMDSVWYPYLLTGYCLMVPVPAKMPYNLVYSMIVDPLVLSIIFVMLCLFSVLIIYTQHLSWKNLTLANILLNDKSLRGLLGQSFPFPPNPSKHLKLIIFVLCFASVMITTMYEAYLQSYFTQPPSEPYIRSFRDIGNSSLKMAISRLEVNVLTSLNNSHFREISEDHLLIFDDLSEYLVLRDSFNTSFIFPVSVDRWNGYEEQQKLFAEPAFYLATNLCFNQFMLFSPPLRRYLPHRHLFEDHMMRQHEFGLVTFWKSQSFIEMVRLGLASMEDLSRKRNEEVSLLLDDISWILKLYLGAMFISSFCFILEILRCGERCKRLWRCRW

>DmelIR52b

MTWLVILLCFLGYMAAHIADISVQNQSLMDNELINLLLKLRNEEFYDTLLVYGKDCEFHSVIKNVDVAVVLVSDSMNFEWNFSSLTLILSCGPDIDNGGPNSTSIKLQRNRRLVLLKEDFQPSNICNIYTQKEQYNIALVRENFTKSKSIYTCRYFQDPNVDEVNLSGTKPIFIEQFQNMKGKAIRIVPDLLPPRVMLYQDANDGELKMIGYVANLITNFAQKVNATLQLDFLKPSTSITEISRMAKDDELDMGITLEASLNTSNLETSSYPYLLTSYCLMVQVPAKFPYNLVYALIVDPLVLGIIFVLFLLLSVLLIYSQKMSWQDLSVANILLNDKSLRGLLGQSFPFPLNASKKLRLIFTILCFASIMLTTMYEAYLQSFFTNPPSEPEICSFQDVGSYNRRIAMSALEVNGLIKTNNSHFREIRMDDLEIFDNMPECYELRDAFNLSYNYVVTGDRWRSYAEQQTLFKEPVFYFARDLCFSRLIFLSVPLRRHLPYRHLFDEHMMQQHEFGFVNYWMSHSFFDMVRLGLTSLKDLSRPLAYTPSLLMDDISWIMKIYLAAIVLCVFCFLLEIGVDKWKRWMKFRNLQILNTC

>DmelIR52c

MVWLIIILFCLGNSSSQILDVTNNSHLDFDYRLFGLLQRLQVEKSYDTLLVYGEDCAIPSLFERLQVPAVLVSSGSTNFDWNFSSLTLILSCNFQDEREENYRTLMKLQTSRRLILLKGHIKPESVCDFYSKKEQHNVAMVKENFYQLEVVYSCRLFQDQNYEKLNLFDGKSIYKDQFRNMHGAPIRTLSDKEPPRTIPYIDSKTGEEKFKGYVGMLISQFVKKVNATMQIREDLIKDDEEVSFVDITNFTSNDILDIGICEARTLEMSNYDAISYPYLMSSYCFMAPLPDSLPFSDVYMAIVAPSILIMFLIIFCICSVLIIYIQERSYRSLTIRSVLMNDICLRGFLAQPFPFPRQYNRKLKLIFMLVCFSSLISTTMYTAYLQAFLWGPPIEPRLTSFDDVKKSRYTMAINIYEREFLEALNVSLEDVEIYDYGKFSKLRSTFNTNYLFPVTALQWFTINEEQKLFKYKIFYYCDAFCLNQFDILSIPLRRHLPYRDIFEEHMLLQKEFGLTKYWIDQSYRDMIRANLTTFKDFSPLLENDYIEVHNLYWVFTMYFVGMGMGLCFFILEILRPLRYWRNCKIKCEYCYAFLKNFAK

>DmelIR52d

MVRIIIILLCLGYTKARILDATNTNHTDLEERLLSLLLRLQQEQFFNTLLIYGEDCAFSSLSRRLQVPTILVSSGSTSFEWNYSSLALILTCEFKAEREENYQTLKKLQMNRRLILLNGNIKPDSVCDFYSKKDQYNIAMVNNNFHQVGIIYACRLFQERNYEKVYLSEGNPIYVDQFRNMQGALLKSITFNLIPGSMAYRDPKTGQEKHIGYVANLLNNFVEKVNATLDMQVKLHKAGKKTSFYNITKWASEDLVDIGMSYAAYFEMTNFDTISYPYLMTSTCFMVPLPDMMPNSEIYMGIVDPPVLVVLIAIFCIFSVMLNYIKQRSWRSLSLVNVLLNDICLRGFLAQPFPFPRQSNRKLKLISMLVCFFSVITTTMYTSYLQSFMWGPPIDPKMCSFADLENSRYKLAIRRYDIEMLRPFNVSMDHVVVFDESSQLEYLRDSFDDNYMYPMSALSWSAFKEQQKLFAFPLFYYSEKLCLKPISFFSFPIRRHLPYRDLFEEHMLQQNEFGLSTYWIDRSFSDMVRLKLATMNDFSPPRLEDYIEVSDLSWVFGMYFTGLGISCCCFGLELLGLPSWTRRLRLTNWLRVRN

>DmelIR54a

MWTVITGIVLWAPVLVAGSAVDFIFRAAAEHSLSVIMIRIDYCPYNWAKDIFENQTIPVVVLSDSETFINIRMFSRPLHVACLPGHELQKDLALLENFTSSLMDFPSQKKIVYISNNFSDPTRMDYIFETCYHRRIWNIVGLLASDEHRYFYRYHLYPSFRTEYRSLESSTIFDKDFPNMHGHPLTVMPDQWLPRSVLYVDRRTGKQILAGSVGRFFHVLSWKLNATLQLSKKVTTGRFLNATALKELSESFSVDVPASLTIMERVEQLASTSYPMEVTHVCLMVPVARRIPIKDIYFILSSASNMFLAIVIVSSYGLALNLLRNMTHRDVRLVDFVLNDKALRGILGQSFNLPLSRSFSTRLIFLMLGIVGLNVSSIFGAGLDTLMAHPPRQFQARSFAGLRRTKIPLVTTEEDFPTWMKLRVPMLVVNVSEYNHLRNGRNTSNAYFASRLYWNLFSEQQKRFTRELFIYSTDDCLWSLALLSFQWPQNSLFTEPVSQLILEVNANGLYDFWVGMHYYDMTAAGLSGLEDPSLQLKEREHPTSLRIVDFQWMWQAYGTFMVIAILVFLLEVSWHRITSLFVSLVY

>DmelIR56a

MGSRFFIRNLILFGLLASSNMQIPFGELEKKFELDVDFLLGVTELVGHIQGLYSITVYADCIDIHPSIQQRIMDKFMVPVNTIGSNLSRPNYHKLDNSRIRIVLFTGLNDTILVNLNKTDVPYSDNFYMLAYASAIKNKCIELDFIEEVFTLLWKMSIQNAILLIRGEFMMEMWSYLYMGKIHKIKLTKPNSYLESLRKYNYRFSLEVINDPPAIFWYNSSEQADVTGGGNLSVSGPLGLIIINFLRHLNVTIDIVPIPGKQTSQYELFQQPDNLRAENGVNMVGSALLKYSPMVTQSRMCLLVSNRRMIPFSRFLDRLVSPGVHKLTFVSSIGIFVIKYFSHRPRSFVDAIFCTIRFFFAIPLPSIILNRLPVVDRFIEVFIIIFVQILLSSNISITTSALTTGFWEPPIINVETMRASGLHILTEDPTILQAFKENILPSSLADLVILVDEDTYFHHVTTLNNSYVYVVQAHNWQIFRLYQQQMTNEPFEIASEELCSKWRILGIPLNPKSPLRFMFKDYFYRILESGLREQWVHSGFKKFCEFNNLKKLPVDSVDSWQPLSIEFYSNVIRAYIIGLVIATLAFVAELLHNGYRRKNVKKT

>DmelIR56b

MLLDTDLASGVIRSPYSFDIPHAFIFNETQFVVPKFCGPYMEIVKHFAEVYHYQLFLDSLESLPKKSVVEQDIISGKYNLSLHGVIIRPEETSDFFNATQHSYPLELMTNCVMVPLAPELPKWMYMVWPLGKYIWTCLFLGTFYVALLLRYVHWREPGNATRSYTRNVLHAMALLMFSANMNMSVKLKHASIRVIIFYTLLYIFGFILTNYHLSHMTAFDMKPVFLRPIDTWSDLIHSRLRIVIHDSLLEELRWLPVEYQALLASPSRSYAYVVTQDAWLFFNRQQKVLIQPYFHLSKVCFGGLFNALPMASNASFADSLNKFILNVWQAGLWNYWEELAFRYAEQAGYAKVFLDTYPVEPLNLEFFTTAWIVLSAGIPISSLAFCLELFIHRRKQRRPQYERFECYDY

>DmelIR56c

MRSSFRICLFLLTTYHPSHGWNMQHLLNLLAPFGRMNVFQEIVWFVSPHQRLDQLDEFIMRIDEAFGKSATQTVVNNNTEMRMIYSSARRNHMSFVFTTGAEDPIMKVFSKVLLGRHFYVSMVIYVDKVGDMHPIYDLLTFAYNQQFFNSMVHFESMEGVNQLFGVSKFPVMSFENRTDFLKYMGKIWKQVQNARSDVGGFGFTTPLRQDLPHLFQSQGHYDGSTYRIIETFVRFINGSFKELIMPPDSLGGQVINMKDALQLIRERKMEFCAHAYALFMSDEELEKSYPLLVVQWCLMVPLYNSVSTYFYPLQPFDWNVWFFALGALLALVLLELMWLRMFGGWSGYRGAVLNSFCYIINVPIEGQLQQPCLLRFLLLATVFFHGFFLSAYYTSNLGSILTVNLFHAQINTMNDIVSAQLPVMIIDYEMEFLLNLNKELPQEFLELLRPVDSAVFSEHQTSFNSSFAYFVTEDHWEFLDEQQKHLKQRLFKLSSICFGSYHLAFPLQMDSSLWRDIEYFTFRIHSSGLLNFYARSSFGSALHAGLVQRMPDTQEYTSAGLQHLAIAFILLLVMSFLAGIVFVLETLSR

>DmelIR56d

MDNRAAELILRERNIFPTNGSDNITLLNNMFVLEMFYRITQLYHFKNFIFYISERLDLNNKDSQEFFHNFWTYFPMAPNLIITREHHLGIPMMQFISTPSLVMVFTTGKDDPIMELASHNQQGIHWLKTIFVLFPSLQSRDFETNPESLAQFTAEIKDVYDWVWRKQFINTFLITIKDNVFILDPYPTPSIVNKTGVWQAEEFFHKYAKNMKGYLVRTPILYDMPRVFKSDRPTNRYEKNFIHGTSGNLFLGFLEFVNATLMDTSANVTADYLNMTNLLDLVSQGVYETLIHSFTEITTKFVVSYSYPIGINDCCIMVPYRNQSPADQYMHEALQENVWVLISLFTLYITVAIYLCSPLRPRDLSAAFLQSICTLTYSVPTFIIRTPTLRMRYLYILLAIWGIVTSNLYISRMTSYFTTAPPVRQINTVQDVVEANLRIKMLAIEYERMAKSPLQYPESYLNQVDLVDKHMLDLHRDPFNTSFGYTVSSDRWRFLNLQQLHLRKPIFRLTEICEGPFYHVFPLHKDSHMRSVMTEYIMIAQQAGLMNHWERETFWEAVHLHRIHVHLFDDEPMALSLDFFSSLLRTWTLGLILAGLAFAAEMKWHEHVTFKRRPVIRITRKPRSFLRRFMKL

>DmelIR56e

ERXAFRNQWAFCFPRTXAIEVVLSAWSPXCPGQRSKPQPISXPHHXGSCWRKRKWKXKPRLLVVDKRTLVEHLNSLNDGYAYCIIAGHWQVGMM

>DmelIR60a

MWCNNPGLIIIIFLGQILNLCQGIVNLSNETANTVIFMLPEKDLGPDVWKAGVGCLDSFAQIFFFRNPKERFTRAYNLMLVHAFHLSSPADQIQEGFSKLINEAVTNPGPPDREELFQMRVASDYNITNGTEDKGELILADNYVIVVDSVDRLKELMKKKIVEMRSWNPGARFLVLFHNATCRNRPLGVASNIFKDLMEMFYVHRVALLYANSTMNYNLLVNDYYSNVNCRILNVQSVGQCHDGKLYPNNAVVKASMQDYVSGFSPRNCTFFACSSISAPFVEADCILGLEMRILGFMKNRLKFDVNQTCSLESRGEMDGPANWTGLLGKVQNNECDFVFGGYYPDNEVADHFWGSDTYLQDAHTWYIKMADRRPAWQALVGIFEAYTWIGFILILIISWLFWFTLVMILPEPKYYQQLSLTAINALAVTISIAVQERPICETTRLFFMALTLYGLNVVATYTSKMIATFQDPGYLHQLDELTEVVAAGIPFGGHEESRDWFENDDDMWIFNGYNISPEFIPQSKNLEAVKWGQRCILSNRMYTMQSPLADVIYAFPNNVFSSPVQMIMKAGFPFLFEMNSIIRLMRDVGIFQKIDADFRYNNTYLNRINKMRPQFPETAIVLTTEHLKGPFFILVVGSCWAALTFIGELIIHRWRTQLVSTSEQQDRRSDKRRRRRRRRKPEKDNRWQRQVQVAPVVRFTPVKRRKVFQGQTSQK

>DmelIR60b

MRRSLYLIIAIGLVDVHCVSLRYILNALENELQYRAILLVESASEIESCWEQKYIQGAVPILNFNANQSLYLKDALNTNILALVCLNENVESTMQALYENLEDMRDTPTILFVLSDSKVQDVFLECLRRKMLNVLAFKGLDRGFVYSFRAFPTFRVIERNVMDILQYFEQQLEDLGGHTLTTLPDNIIPRTVVYKSPDGSRQLAGYLYPFLRNYVSTINATLKVCWHLVPEDGMIQLGEVVRLSEIHDVDFPLGMHGIEHGSTSQNVPLEVSSWFLMLPMEPSLSRAQFFIMLGFEKVTPVLLLLTILLSTAHRIEMGLRPSWRCYVLGDRVLQGTLGQAFFLPRRLSVKLMLVYSLILLNGFTFSNYSITSLETWLVHPPSGHPIHSWEQMRTLNLKVLIVPSELDSMTKALGKQFTESNSDLFELSKSGNFQDKRLAMDQSYAYPVTCTLWPLLEHAQIRLPKPEFRRSREMVLIPLLIMAMPLPKNSMFHKSLNRYRALTHQSGLYEFWFKRSFNELVALRKIHYKVNGDHQIYRDFEWQDFSYVWLGFVGGTIASILVLLAEIGYHRWQLNQN

>DmelIR60c

MEMRLALFFTFACLAGAHDGSLRNMLKSLEDELGYRTILLLEGFVYSFKAFPTLRVVKRRVKDVRRYFEPQLEDLGGCVLKVVPDGIMPRTMVYQGEDGELQMGGYLSHFIRNYVSTINASLQIRWDLFPEDGDFDMDSLTGSNHVDFPLGLGSLSFQTLHKDVAMEISSWFLMLPMEPSLPRARFFIRFGISLYLIPLIILLAIVLSNAHRFEAGLTPSWRCCSMGNTVLRGVLAQAFVLPKGLSPKLMFVYWLLLVSGFFVSNYVIVYLTAWLIQPPTSDPVTDFDQMRRAKLKILMVPTDMDYLKSIRGAEYVDAHSDVFQTADSTDFQTQRMSMELHFAFSVTGTLWPLLRQAQVKLHRPIFRRSKEMVFLPFVIMGMTMPNNSIFLSSLKQYRLRTSEAGLYLLWFKKSFSELVAIHKISYKEDWVHDSYSDLKWEDFLFAWLGFLGGTTVSCLALLAEIGYHRWLWKRTHQ

>DmelIR60d

MRLAIYVAFLSSIGNRSGFLSSLLMSLGKELHYKTILLVGGSSTCWSLEPFETGVPILNLRGENNAYPQDTFNSQMLALACLQTESEDAVKLLYRSLKDMRDTPTLLFASSEEHIHDTLFLGCFRENMLNVLALTASSKEFIYSYQAFPTFRVIKRKLVEIHRYFEPQLKDLGGHIVSALPGNIMPRTMCYRNAEGERQLAGYLNTFIRNYVESINGTLRISWGLVPEDDMRHLTISRLSKIQHVDFPLGIIPLYNKTDKQHVYMEISSWFLMLPMETSVPRAHLFVKLGLERLLPIIVVVGAVLGNAHRIEVGLGPSWRCYYLADKVLRGALAQPIVLPRRLSPKLMLIYSLLLLSGFFLSNYYMASLTTWLVHPPASDRILEWDQLRYLHLKVLTIPEEFKYMSLILGTDFMTAYGSIFQLTNSTDFQRRRISMDPSYAYPVTTSLWPFLELSQVRLRRPLFRRSYDMVLQPFQVMSLPLPRNSIFHKSLLRYAALTRETGLYYYWFRRSYYELVALGKISYKEEEGNPYCDLKWNDFRIVWLAFLGGTIISCLALLLEVAHYRWHLGNSSL

>DmelIR60e

MVIKMISFLLVSVLLCLVGASDSESMQVQVLQDLNLALQTELNVFIDFECCATSEILHKLDSPRILLSSNSREARDLRIRGNFTESTLIIVSVMDSDLNPLVASLLPRLLDELHELHIVFLSNEEPGFPKQDLYTYCFKEGFVNVILMSGKGLYSYLPYPSIQPISLSNVSEYFDRARIIRNFQGFPVRILRSTLAPRDFEYSNEQGGLVRAGYLFTAVKELTYRYNATIESVPIPDLPEYDVYLAVAEMLHTKKIDIVCYFKDFSLEVAYTAPLSIIREYFMAPHARPISSYLYYSKPFGWTLWAVVISTVLYGTVMLHLAARGARVEIGKCLLYSLSHILYNCHQKIRVAGWRDVAIHGILTIGGFILTNVYLATLSSILTSGLYDEEYNTLEDLARAPYPSLHDEYYRSQMKAKTFLPERLRRNSLSLNATLLKAYRDGLNQSYIYILYEDRLELILMQQYLLKTPRFNMIRQAVGFTLESYCVSNSLPYLAMTSEFMRRLQEHGISIKMKADTFRELIHQGIYTLMRDDEPPAKAFDLDYYFFAFVLXTVGLISSLLVFFAELVSGHL

>DmelIR60f

MRFHLNIANSGLLGLHLCPTRSALPEQNPCFSKAGAVIXNLTLPWRRWRERCLLGALRPXTLPTPELQCXSKYLPXRKSQQENASSGLPGFCXGDXQTELHRGSRAIALPRSPYHDLYYVWIAYLGGTMIGIGMLAVEIACFKWDLLRRPPIXMY

>DmelIR62a

MYLQFLFALFLSRYQIVATENFDRAFELALFLDRIGRVHRLHAITIVNSLGSVDPSYLDDLHRGLMCNSSNHFYMLPQMTATDKDSSHVHFSSLQDEETIYLVFARDSKDAVIYLQAERARGRRYTRTMFLLRKQESQKDIKYFFELLWKLQFRSALVVVAARNFYQMDPYPTVRVIRMRRLSSYDPHHVFPPANRKNFRGYRMRLPVQQDVPNTFWYKNRRTKAWELAGLGGILINQLMMHLNVTMDLFRFEVNGSSLLNMAALTDLIVKGKVELSPHLYDTLQSNTSVDYSYPTQVAPRCFMIPLDNEISRSLYVFLPFSLTMWLCLLFVLLVVHFVYVRRLIPDGHFWAILGVPGAGQVRYGNRKPVRRFSTFLILFGIFILGQTYSTKLTSSLTVTLIRRPDNSLEELFLLPYRILVLPTDVYAIVDSLGHAEQFSTKFSCTDAENFSQKRISMHPEYIYPISTIRWRFFDMQQRFLRKKRFYFSKICHGSFPYQYQLRVDSHLKDALHRFLLHVQQAGLHDLWLDTCYRKAHRMGYLKDFSTLAELEEKLRLRPLALNLLVPAFSLFLCGMLGSGIAFLVEIRHSFGCRQKPPSINRNPGD

>DmelIR67a

MLPILVPVLLLFNETSWINPILTSIYKDRHHETVLLLQHSQHGNASGLERFPWPVFSFNEQMDFYVRGKYNSEMLVLIWQTGNSDWDLDLWQALDRSLLNMRKVRVLLLRKWEKIPTADVAATAEHLLFLHVAVIGQGNRIYRLQPYAPQSWLQVDPIESPIFIKIRNYFGRYIVTLPDQFPPRSIVYRNPKTDEIQMTGYVYKFLLEFIRIYNFTFRWQRPIVQGERMNLILLRNMTLNGTINLAISLCGFETPSXLGVFSDVYDMEEWYIMVPRAQEISIADVYVVMVSGNFLIVLIIFYFIFTILDTCFGPLLLKERVDWSNLMLNERMISGIMGQSFNMSARNTISSKVTNATLFLLGLVLSTLYAAHLKTLLTKRPTSQQISNFKQLRDSPVTVFFEEAERFYLKHAWDRPIRYIKDQLNFRETIEYNALRMGLNRSNAFSALTSEWMIVAKRQELFKQPIFTVQPELRVIQTSVLLSLVMQSNSIYEDHINDLIHRVQSAGIVEYWKHQTLREMITMGMISQKDPFPYVAFREFKVGDLFWIWLLWVSFLFMSFVIFLCELLVDCFISKTLIRNKRPH

>DmelIR67b

MELLYLNTLQSLSLLEGNRLVQTVQELNNIYQTELNVFLEFGNGADILESAQGTFVPTLWIKNPQNQKVMKGNFTSCTLTILYLEDEHLDRGLYYLANWLWEYHHLEVLIFFNGGSYDKLIQIFSRCFNEGFVNVLVMLPGSDELYTFMPYQDLKILNLKSIKEFYSLSRKKMDLNGYNITSGLVIAGAPRWFSFRDRQNRLILTGYMLRMIVDFTNHFNGSVRLMNVLTVNDGLELLANRTIDFFPFLIRPLKSFSMSNILYLENCGLIVPTSRPLPNWVYLLRPYAFDTWIAWLIMLIYCSLALRILSKGQISISAAFLKVLRLVMYLSGSRDMGTRPTTRRLFLFVILTTSGFILTNLYVAQLSSNSAAGLYEKQINTWEDLDKSDSIWPLIDVDIKTMEKLIPDRTKLLKKIVPTLEADVDTYRRNLNTSCIHSGFFDRIDFALYQQKFLRFPIFRKFPHLLYQQPLQISAAFGRPYLQLFNWFVRKIFESGIYLKMKDDAYRHGIQSGLLNLAFRDRHLEVKSNDVEYYYLIAGLWFGGLTLATVCFLLELLIGYAKIKVTISCKMNIM

>DmelIR67c

MFCWLIFLNIILLSDRSESWSAREVIHQFNHDQQLQLNIYLDCNDVELQIGQEVSNLFVNSTADKMKILGRFSSHSLIIACFKDSTRNRTLNGVKELLWGLQYLPILFVVDSNMDFYFQQALRHGFIHVLALNFMNGSLYTYKPYPKVEVHQIKDMQKFYKLTKLRNLQGQAVRTTVETMTPRCFRYRNRHGQLVYAGYMYRMVKEFISTYNGTEEHVFGNVDTVPYKEGLAALKNGEIDMMPRIIHALEWYYFYRSHILYNIKTYIMVPWAEPLPKSLYFIQPFRGTVWITIMVSFVYASIVIWWIRYRQQGNSSLTQSFMDVLQLLFQLPLSKIWHFNMGTHQVVSFIVLFVFGFMLTNLYTAQLSSYLTTGLFKSQINTFDDLFREKRTLLVESFDAEVLHNMTKEKIIQKEFESIILITSIEEVFKHRKSLNTSYAYEAYEDRIAFELSQQRYLRVPIFKILKEVYDQRPVFVALRHGLPYVELFNNYLRRIFESGIWIKLQEDSFLEGIASGEISFRKSKSREIKIFDKDFYFFAYILLGMGWCVSTIALFLELWSFKYSVTNVLHEG

>DmelIR68a

MRCLWILIVAFISLAMATSIPIPIANPAPLSGYEMQLKILLQKILWVANVKRCFAVITDDLHYPIYDRIFFESVGRRVIPFFVMRTNESDDLQRPSRQVELFVKAIKSSDCELNVITILNGWQVQRFLGYIYDNRSLNMQKKFVLLHDLRLFESDMIHLWSVFIDAIFLKRQLDNKYTISTIAFPGILSGVLVMKNIANWELGKGLNGRILFADKTSNLFGTSLPVAISEHVPMVLWANATKSFQGVEVEIMNALGKALNFKPVYYKPNQTENMDWTELDGGASVAYGSGNPDGYAQNGTHIDSMLVDEVAAHSARFAIGDLHLFQVYLKLVELSAPHNFECLTFLTPESSTDNSWQTFILPFSAGMWVGVLLSLFVVGTVFYAISFLNAIINGNVSSEFFRCLRPNRNVPMDPKIYRRISFRIAISRYRSSKGDRMPRDLFDGYTNCILLTYSMLLYVALPRMPRNWPLRVLTGWYWIYCILLVATYRASFTAILANPAARVTIDTLEDLLRSHIPPSTGATENRQFFLEANDEVARKVGEKMEVFGYSDDLTSRIAKGQCAYYDNEFYLRYLRVADESGSALHIMKECVLYMPVVLAMEKNSALKPRVDASIQHLAEGGLIAKWLKDAIEHLPAEALAQQEALMNIQKFWSSFVALLIGYVISMLTLLAERWHFKHIVMKHPMYDVYNPSLYYNFKRIYPQH

>DmelIR68b

MKFLVGLLLQWYLPGIYALAEIACRIAVEQNVQVTYLYRCASCPASFDADYSALELDLYRCVGSRLPVITRNMEAHELEPFRRTDSLSIFQIPAAEKGDSLVRRILDMLNPHQRRKHMHKYLFVWPNAGRHQLLRLFRGSWAKKLLYGLAITGRENGTFDFDPFAWGGLQVIQRLDGEVPYARKVKDLRGYPLRFSMFTDPLMAMPRSPVETAGYQAVDGVAARVVGEMLNASVTYVFPEDNESYGRCLPNGNYTGVVSDIVGGHTHFAPNSRFVLDCIWPAVEVLYPYTRRNLHLVVPASAIQPEYLIFVRVFRRTVWYLLLVTLLVVVLVFWVMQRLQRRIPRRGVIQFQATWYEILEMFGKTHVGEPAGRLSSFSSMRTFLMGWILFSYVLSTIYFAKLESGFVRPSYEEQVDRVDDLVHLDVHIYAVTTMYDAVRSALTEHQYGLLENRSRQLPLGIATSYYQPVVRRRDRRAAFIMRDFHARDFLAITYDSQAERPAYHIAREYLRSMICTYILPRGSPFLHRLESLYSGFLEHGFFEHWRQMDLITRVGASPDAEEFLEDLGDQTDTDSGSNELAIRNKKVVLTLDILQGAFYLWSVGIGISCLGFAVEHAHWFWRRQTLRNAVEARTS

>DmelIR85a

MSIQWLKHILLLAILVNLAGTRENHIPLDLKKSSIVMVKMSQILCKARIKVLFVYFENQTSHEHTGQILKEVTKCDISNQNTPLEAVKDDGILMYMVMITTNISQPLELSLIRKKSAAKHRSHVFLLVRDADTVSDAWMRASFRQFWKIWLLNIVILYWRDGRLNAYRYNPFMDNYLIPVDNKPNEVPTLEQLFPKTIPNMQRKPLRMCIYKDDVRAIFWRQGTILGTDGLLAAYVAERLNATMMITRPHSYNNHNLSSDICFLEVAKEYVDVAMNIRFLVPDTFRKQAESTVSHTRDDLCVIVPKAKTAPTFWNIFRSFGSLVWALILVSVLVANVFCYILKSEVGRVPMQLFAGALTMPMTQIPPNHSIRLFLIFWLYFGLLICSAFKGNLTSMMVFQPYLPDINQLGALARSHYHIIIRPRHVKHIQHFLTLGHKHESRIREQMLEVSDTQMYEMMRNNDIRFAYLEKYHIARFQVNSRVHMHLGRPLFHLMNSCLVPFHAVYIVPYGSPYLGFLDSLIRSSHEFGFERYWDRIMNSAFIKSGVKVVNRRRGSGNDEPVVLKLQHFHAVFALWLVGIGMACIVLAWEHLTHNYNLAVTKRRD

>DmelIR87a

MSTPEQRFWLAALLFLLSQHSEVRGFGINLMKVQTEDKGQEACILALLRKYFDSGDGLSGSVLCINRNYQLPNIEEQLLRGVNNYENYPWSLLITNSREGPSPAKFLMNEKPQCYFLIVDNLEDEDLDEVFEHWKGMVNWNPLAQFVVYLASLEETDEEMNDLMVELLLTFINKKIFNVNVIGQSEENQFYYGKTVFPYHPDNNCGNRVISVELLDACDYPSEETDSEDENDEDEGDGAQEEDDGPQEEGDGEQEEEDGPQEQEDGDQAKGDEGQENDDGGLENKVENEFRIGASDDDELENDLSSNSSEPEAIIEEFFRAKFEDKFPRDLSGCPLTASFRPWEPYIFRNSEEQPVDDYYYGLQGDEDDYNDTSPNYGESDDESYADPGEDGDGAIPDTETQSGGKLKLSGIEYEMVQTIAERLHVSIEMQGENSNLYHLFQQLIDGEIEMIVGGIDEDPSISQFVSSSIPYHQDELTWCVARAKRRHGFFNFVATFNADAGFLIGIFVVTCSLVVWLAQRVSGFQLRNLNGYFPTCLRVLGILLNQAIPAQDFPITLRQLFALSFLMGFFFSNTYQSFLISTLTTPRSSYQIHTLQEIYSNKMTVMGTSEHVRHLNKDGEIFKYIREKFQMCYNLVDCLNDAAQNEHIAVAVSRQHSFYNPRIQRDRLYCFDRRESLYVYLVTMLLPKKYHLLHQINPVIQHIIESGHMQKWARDLDMRRMIHEEITRVREDPFKALTFDQFRGAIAFSGGLLLVASCVFAFELCYVKYVYRTEKRERKTKKITKKVHNIKIQHD

>DmelIR94a

MALPKQLKFINIFLVLLIIYGSSDGTENQHEIFLNRLLQAVHNERSVETLFLLHHSNLANCSLQDWNPPRIPTIRSNELTVFNVEKTFNHNALALVCLMKNSYREILNTLAKSFDCMRQERIILMIHRKSDSKFIEDITHEVKNLQFLHLIVLIVQEKYNGQVFASTLRLQSFPEPHFKRIRNVFAIQRIFYRPINFHGKVLNAIPNDIPILFVALNEMFTEYARRYNSTLRIQNRTIKEDIEITEDNYDIDMKIQLHNSQNFLHHMNIAMDIGSNSLIILVPCATELRGLDIFKELGVRTLTWLALLFYIIFVLVEMLFVFISNRFNGRNFTMRYTNPLINLRAVRAILGQTSPISNRYSLSIQHFFVFMSLFGTLFGGFFDCKLRSFLTKRPYYSQIENFSELRKSGVTVVVDHTTRQFIEQEINANFFRDEVPNVRTTTIQELINHVYSYDRKFAFVANSIPWRTFREEMKSINQKILCDSKNLTILENVPLTFSIRRNAIFSHHLRNFIINAADSGMITCWFKMAGKVIRKHIKTTLRESEQQPSHLPLSFDHFKWLWAVLCIAYVMSFMVFVMEILWSKYQRRTRSVSIV

>DmelIR94b

MSLIFNLLFILILSQAVSQETEFLQLKYLNNIVRSMIKLHKMETLVIVKHHLDNNCSLQNWNAHGMGIIRTNDQGKLIMKDTFNSRTLAIICIGQNSHITLLRNVFETFGKVQQKKIILWTQMELKEKFFQEISKKSRDLKLLNLLVLKAVTKDKLLIYRLNPFPSPHFKRIENIWTPNDTLFMDTKFNFHGMTAVVKHDYNWTIQMGNIRKFPISRIEDKEVIEFALKYNLTLQFFNDVERFDIELRKRIILKSNSTQPIDSGIPMVFSSLLIVVPCGNYLSIQDVIKVSGIEKWIFYIILVYVIFVLIEITFLGVTILISRQSRHQMIPNTLVNLCAFRAILGLPFPETRRTSLSLRQLFLAIALFGMIFSIFINCKLSSMLTNPCPRPQVNNFEELKTSGLTVVMDHDAENFIEKEIGVDFFNQYMPRKVTLTFTERAKLLFSLKGNHAFTLFSESFAIIESYQRSKGLRAHCTSEDLIVAERVPRIYILENNSILDRPLRRFIRQMQESGITNHWLKNIPSSLEKNLMQITIPYDRERVHPLSIEHLTWLWCILILGYSISMIVFFVEMSLKRRKKNLENRAPNICIC

>DmelIR94c

MSKVFKLLVLPLIYLSLTKGSKNPQLKFLRELINVIEEGREIRTIMVIKHSRDEYCHLDQWNPRGSPILRTNEMGSIRISGYFNDQAVILACMGENSDYGLLKSLANAMDNMRQERIILWSEREPTKMLMDYISQQADRYNFAQIIIVTMNEDVDAVPSLHQLNPYPTPRFRQITNISNIRRTSFFGCGLSFQGKTAILKESVVSNIRFKVWSPSGPIPLSELKDYEIVQFAVKYNLSLKLYDQNESKSDHFDIQLGPLFITKDFPTQMAFVSPNTACSLIVIVPCSPKWRFMDVLHKLGVLKLIGCLLIAYAVFVLIETLILWLTHRISGREVRLTSLNQLLNPRAFRGILGLPFPEFRRSSISLRQLFLVISVFGLVYSNFVSCTLSALLTKPAQNPQVRNFKELRDSGLITIMDKYTHSFIEKHIDPEFFDHVLPHYLILQKKEALRMIWNFNDSYSYVMYTTTWKSLNTVQKSFDERVFCESESLTIAWNLPRMYVLGNNSVLKWMLSRYITYMPQTGIPDSWTEQLPKVLKLLYNVTSPRRIKEGAVPLSIQHLSWIWHLLFIGESIATLVFIVEILLQKSNQHTSNMRERSSED

DDFV

>DmelIR94d

MGQLHLLLVALVLLSPGGDSFYHSLIHHLNRELKIEYVLLLGNFDTTWLDILWQLPVSVLQIKEHSRETYSLLENPSHNVLTIAFVNDSPEDILEILYRNLRMLNTQPVLLVIRKSTIRVNSLLEWCWHHQLLKVVAIAQDFMESLIVYSYNPFPVLQFIERRLDNSTVIFEKRLENLHGYEVPIALGGSSPRLIVYRDLEGKLIFSGPVGNFMKSFEQRYNCRLVQPYPFDESAISPARDLIASVQNGSVQIALGAIYPQVPYTGYSYPIELMSWCLMMPVPEEVPHSQLYSMVFSPMAFGITIVAMVLISLTLSMALRLHGYRVSFSEYFLHDSCLRGVLSQSFYEVLRAPALIKAMYLVICLLGLLITSWYNSYFSTFVTSAPRFPQLTSYESIRHSNIKIVIWKPEYEMLLFFSENMEKYSSIFQLQEDYKEFLHLRDSFDTRYGYMMPMEKWSLMKEQQRVFSSPLFSLQDDLCVFHTVPIVFPMVKNSIFKEPFDRLILDVTATGLLSRWRDMSFTEMIKAGQLGLEDRGHPKEFRAMKVGDLIQIWRFVGWMLGLATIVFLLELICFWRHKMWQNMKYMFCRNKNI

>DmelIR94e

MDCPKWILSGLCLISLVSGATVIELLGTLKLELDFEYVLLMKNRNFSLSDQVWNGTSLTKDVMDEVQVPVLQFNENVSYFLHNSISRRLVTLGFMSDANLDEHRGLLTALVANLRHMTTSRVIFLVQSKASTDFLYELFRNCWRKKLLNVIVIFQDFETTSTFYSYSNFPILQIEERIYETSLQTLPIFPDRLRNLHGYEMPVILGGTAPRMIAYRNKKGNVVYDGTVGHFMTAFQQKYNVKFVQPLQAKNPLDFAPSMQTVGAVRNETVEISISLTFPTIPPFGFSYPYEQMNWCVMLPVEADVPPFEYYTRVFELAAFLLTLGTLVLISCLLASALSLHGYATNISEFLLHDSCLRGVLGQSFVEVFRAPTLVRGIYLEICVLGILITAWYNSYFSSYVTSAPKQPPFRTYDDILASKLKVVAWKPEYAELVGRLLEFRKYETMFLVEPDFNRYLALRDTLDTRYGYMITTNRWVLINEQQKVFSRPLFQKRDDFCFFNNIPFGFPLHENSVFMEPVQKLIMELAETGLYYHWITTGFSELIDAGEMHFVDLSPHREFRAMQIQDLQYVWYGYAFMVVLSSLVWLLENLAYTVKSKTIFPTHFMQRNKK

>DmelIR94f

MSGMWQQVLLAETSNWFRSDVLQRFWTHLRVEIRFRTMLNYRLESCDCWFDNVLGSDNSTALLWNDQTYPHYLRRRQDTDILVVSCLRFHQYQEVLLALSLMLDQMRSMPVVLQLCGDEDSMQELNSARLLLKHSQDLKMPNVVLLSSTFFTSATLYSYEMFPEFNVQKLVYQAYLTLFPYKLGNLKGHPIRTVPDNSEPLTIVRKTLNGSIAIDGLVWQFMIEFAKHINATLQLPIEPHPEKSIKLVQILDLVRNQTVDIAASLRPYSLNVQRSSTHIYGSPMMVGNWCMMLPTERVIGSHEALTRLMKSPWTWLILLLFYSVHRFLAQKTRLRSSLIHLIKLLINLSLICFLQAQLSAYFIGPQKVNHISNMQQVEESGLKIRGMRGEFMEYPIDMRSRYASSFLLHDLFFDLAQYRNSLNTSYGYTVTSVKWELYKEAQRHFRRPLFRYSEEICVQKLSLFSLIQQSNCIYCYRSRIFILRMHEAGLIRLWYRRSYYVMVTAGRFPIGDLSTVHRAQPIRWTEWQNVVLLHGVGLLFSVVVFVIELTVHYANVCLNNL

>DmelIR94g

MSTAVNSVHSKLVSLISRGQELTSIFFYAPAKEKCHLEDTISSATWGLPLVIWRTDRTVILNGFIGEGLLVLACLPGFHWRALLGSLARSLKYLRQARILIELMQDRDEFLVSEVLQFCLSQDMINVNAIFDDFPETENLSSFEAYPSFEVVNQTFTPDTQVSDLYPNKMLNLRGGVIRTMPDYSEPNTILYQDKEGNKEILGYLWDLLEAYAHKHNAQLQVVNKYADDRPLNFIELLDAAQSGIIDVGASIQPMSMGSLSRMHEMSYPVNQASWCTMLPVERQLHVSELLTRVIPYPTLALLLLLWIFYEVLRGRWRRHSRLQSIGWLVLATLVSSNYVGKLLNLFTDPPSLPPVNSLAALMESPVRIISIRSEYSAIEFTQRTKYSAAFHLALHASILIGLRNAFNTSYGYTITSEKWKIYEEQQKRSSKPVFRYSKDLCFYEMIPFGLVIPENSPHRAPLHSYTLLLRQAGLHDFWVNRGFSYMVKAGKINFTAVGERYEAKTLTITDLRNVFIIYVSVLLISLILFTCELFVSWVN

YWLGF

>DmelIR94h

MLSNISFSSAPELVDLYGLVLKFLVSSETTLFYFNPTGQKCSWETLPRTILSNHPQIIWFREETYPGLYKRHSSNLFVMACLSSTSYDGQLQLLAESLTRYRSVRVLIEVQDKEGSFLASQILLLCQQHSMLNVVLYFSRWTRTLNVFSYLAFPYFKLLKQRLSGSLRPKIFINQLKDLQGYKIRVQPDLSPPNSFSYRDRHGECQVGGFLWRIVENFSKSLKGDTQVLYPTWAKAKVSAAEYMIQFTRNGSSDIGVTTTMITFKHEERYRDYSYPMYDISWCTMLPVEKPLSVEILFSH

VLSPGSALLLILAFILFFLIVPQLIKCLGITFRGRLIGMASRIFALVMLCSSSAQLLSLLMSPPLHTRIKSFDDLLTSGLKIFGIRSELYFLDGGFRAKYASAFHLTENPNELYDNRNYFNTSWAYTITSVKWNVIEAQQRHFAHPVFRYSTDLCFSSETPWGLLIAPESFYREPLQHFTLKINQAGLITQWMTQSFHEMVRAGRMTIKDYSRTNLMKPLRIQDLRKCWVIFAVGLGTSTVVFTIELLLIYTNVFLNSL

>DmelIR100a

MATTLQLIMLALVGGTLGQANNTDHKQVLTSIVKQLEGGLELHLRTSEDGGNDLVQFLMQEKSSIIISAKQEEVPSRAKIMRHHFFIFDGVHQMQEIRTSLFNTDGFYILALENNTIEDDVLLMEFAADVWLQHGHSRIYYVQLSKKSVLLFNPFLQRLVVVQDSKTYSRIYKDLEGYHLRIYIFDSVYSSVIGDGENKVLSVTGADAKLAKTVARQLNFTADFVWPDDEFFGGRLANGEYSGGVGRAHRGEVDIIFAGFFIKDYLTTHIQFSAAVYMDELCLYVKKAQRIPQSILPLFAVHMDVWLCFLLVGLLGALVWLILRAVNLILGIEGVPDGSRATRISYFGAARRIFVDTWVIWVRVNVGRFPPFHSERIFVASLCLVSVIFGALLESSLATVYIRPLYYRDVNTLRELDESGQPIYIKHPAFKDDLFYGHNSEVYRRLDAKMMLVAEGEERLIEMVSKRGGFAGVTRSASLQLSDIRYVMTKKVHKIPECPKNYHIAYVLPRPSPYLEEVNRIVLRLVAGGIVGLWTGEAKERAKWSIQRFPEYLAELDVGRWKVLTLSDVQLAFYALTIGCLLSAIVCMAEILLGRQRRLHSPK

>HarmiGluR1

MVFNTFYATRYTGVLLLNAVILSAQPFAVEKIAVGAIFDQNTEEIQNVFKYAMTIHNQNISSRRLELQAYVDVINTADAFKLSRLICNQFARGVFAMLGAVTPESFDTLHSYTNTFQMPFVTPWFPEKVIPPSSGLIDHAVSMRPDYHKAIVDTILYYGWKEIIYMYDSHDGLLRLQQLYQTMQPGRTAFRIALVKRINNASDAIEFLLALEQYDRWGNKRIVLDCNAKNAKSILVEHVRKVQLGRRTYHYMLSGLVMDDHWENEVTEYGAVNITGFRIVDHSRKIVRDFMDGLRRMDPRFKGTISAETALMYDGVQVLMDALGRLWRKKPDAFRSALRRAAGQANSTKVIDCNPGKSWVVPFEHGDKISRLIKKTDIEGLTGNISFNEEGHRHNFTLHVVEMTVQSAMLKVATWSDAHGLQVATPRYVQLRSPASYDTNKTYIVTSFLEEPYLMQKPIEFGQKEELFGFCKDLMDVIAKKMGIKYKLKLSNDANYASDALPDIHSGVVGEIVRKEADIAIAPFAVTPERERLVDFSEPFLTLDTPIAYTRTPRQLSDTFSFLRPLSKEIWLCVLFSFFAVSIVLFLVSRFSPHEWKSVSISDTQLDHTMSSTSEIILHNEFSIWNSFWFSLGSFMQQGSDVVPRSLSGRIVGTVWWFFALILVCSYTANLAAYLIVERIAEPALSTVSYSPNIAHTESSSLNFRNNFVKDPVLNEEAISAYSDDGEACGPSRVCRYKHVNFAFATAKGSPLREAINLAIVNLKKEDFITKLWRKWATYNKKPDCEMIKDEETTITEMTLSQVAGIFYVLVGGLALALGVALVEFCQHGRAEAARANVPLRAALRAKARLASRTERKTPPQRTQGDHERLGWNGAAFAGYFTSGTQISQEDAVHASFTHV

>HarmiGluR2

MSRLLLKWLAATLCLLRVHGDRTLGAIFDDGTFLLEAAFNVAIAAASEDQENPFVANVIKTSPSDITEAENAMCTLLESNVFGVFGPTKKGSLQHIQSIADYLEIPHIITDPVETQNRNWSVINLFPHHLAYSQLFADLIELKGWTDFTIIYEGAELLPFFDSILSMQDLDTGQKILIKIVQLPDGDDFRSQLKFIKKSGSVNYIINCRRETLPLVLEQAQQVGIMSDEHSYLIMNPDFQTIDIDPFKHGGSSITGIRMFDPSLESIQNFITSLNEKVAELSENEIENAIAENGLTLDLALVYDAVTLFVSTLNAMSLEEGSNVTCDDAESWGFGSSIVNYARTMEVDGLTGIIKFDEDGFRSEIEIDVLEIMSYGLDKVGTWTLEDGFVETKDNVSPAEQEGSESMKGKHFVVLTALSAPYGMLKESLKKLEGNDRYEGFGIELIDELAKINEFNYTFDIQEDGVYGSYDKKTGKWNGMMEKIMDGRADFAITDLTITAARQKAVDFTSPFMNLGITILYKKPTKEPPDLFSFISPFSMGVWGWLAGAFVGVSCLLFILGRLAPEEWQNPYPCIEEPETLDNQFTLANSFWFTLGSVLTQGSEIAPIAVSTRMAGSMWWFFTLIMVSSYTANLAAFLTVESKFYAIKSVNDLASNPYGMTYGAKKGGATFSFFKESDNLLYQKMYHYMEDHPELQTATNDQGLDRVKSDSENYAFLMESTSIEYMVERNCDVAQVGGLLDSKGYGIAMKKNSPYRQPMSESILQLQEEGKLTRMKDKWWKEKRGGGACADDDAGGGEAQPLVLANVGGVFIVLAAGSGMAVVCAFVEMVFDVWMISRKMKVSFREELKAELKFILSFSGDTKPVRHRESTGSGSGGSKDDGEKNADAESPDDDDRADPSPTPRSERSGHSHHTLHSRRQSNAVQMAKMRKYSLRSAM

>HarmiGluR3

MWPGWRWVVLMVNAVAALSPVVKIGAVFTEDARGGSTELAFKYAVYRINKERLLLPNSTLVYDIQYTPSRDTFKTYKKACAQIQSGAVALVSGVGPLLGNTLQHMSASLHAPHLTVGPFAPENLNNTFTINLYPPKDLLTKAFAEFLSYLNWTRMGVIYEDYGYGELNILDIAKDGRDMYAVRCRDAKEYRRGLALLKAQQIEHIVVDTDPKRVRQLARAILQLQMNNENYHYVFTSFDFELFDMEDFYYNRVNMSGWRLVDRHSDKVKESLQVMEKFHPIGASIISGGHIKTEPALLYDAVQILCQALAITEDIHPGNVSCDKDTPSLHGKAIYDNINTIQAHGLTGPLEFKQGIRKNFHLQLMRLTGGEKGGMVVSGTWSPAEGLAITDPAAYTRDPPPNVTLTVVTVEEKPYVMVKEGWNLQGNARFEGFCIDLLARVAARAGFHYRLRLVPDNMYGARDPDTGHWNGIVRELMDRKADIAVASMTINYAREAVIDFTKPFMNLGIGILFKVPTSQPTRLFSFLNPLAIEIWLYVLAAYILVSFTLFVMARFSPYEWSTSTHVCGHETKLLTNQFSVCNSFWFITGTFLRQGSGLNPKATSTRIVGGIWWFFTLIILSSYTANLAAFLTVERTVLPIQSAADLAAQHHIHYGTLNGGSTMSFFRDSNIDIYQKMWEHMSSASPPALVSSYEEGVRRVLAGNYAFLMESTMLDHRVQRDCNLTQIGGLLDSKGYGIATWKGSPWRDKISLAILELQEKGVIQILYDKWWKNTGDVCNRDGKDSKANPLGVQNIGGVFVTLLCGLALAIVVAILEFCWNTKKNASQGRQSLCSEMGQELRTAMRGGSSSRTVLRPGCSRCSPATHVPPATSRYQHSRSSSVELKELRWS

>HarmiGluR4

MRGANAIFLILFFGHLSALPDTIRIGGLFHPEDEKQEVAFRYAVERVNADRAVLPRAKLLAQVETISPQDSFHASKRVCHLLRSGVAAIFGPQSAPAAAHVQSICDTMELPHLETRWDYRTRRESCLVNLYPHPAALSRAYVDLVRAWGWRSFTIVYENSDGLVRLQELLKAHGPSELPVAVRQLPDSHDYRPLLKQIKNSAESHIVLDCTTERIRDVLQQAQQIGMMSDYHSYLITSLDLHSVDLEEFKYGGTNITALRLLDPERADVQRVVRDWVYDEARKGRKLQLGHTTAKENMTFIKTETALMYDAVHLFAKALHDLDTSQQIDVRPLSCEAEDTWPHGYSLINYMKIVEMKGLTGVIKFDHQGFRSDFTLDIIELTRDGLQKAGTWNSSEGVNYTRSYGENQKQIVEILQNKTLIVTTILSSPYCMRREASEKLTGNAQFEGYAIDLIHEISKILGFNYTFKLAPDGRYGSYNRETKEWDGMIRELLEQRADLAIADLTITYDREQVVDFTMPFMNLGISVLYRKPIKQPPNLFSFLSPLSLDVWIYMATAYLGVSVLLFILARFSPYEWDSPRNCLDEPPVLENQFTLLNSLWFTIGSLMQQGSDIAPKAVSTRMVAGMWWFFTLIMISSYTANLAAFLTVERMDSPIESAEDLAKQTKIKYGALKGGSTAAFFRDSNFSTYQRMWSFMESARPSVFATSNKEGEERVVRGKGAYAYLMESTTIEYVVERNCDLTQVGGMLDSKGYGIAMPPNSPYRTAISGAVLKLQEEGKLHILKTKWWKEKRGGGSCRDETSKSSSTANELGLANVGGVFVVLMGGMGVACVIAVCEFVWKSRKVAVDERKEEASLCSEMASELRSALKCPGGGAGGGGGGPGGARDGAGSPYLHYGFSTKSQLH

>HarmiGluR5

MAKNKGTRMWSYHVFLAVVYCGGQLVTADIDRRRFSNPTYYNVGGVLSSNESIAFFKDTISNLNFKDQYVPRGVTYHDYSMLMDPNPIKTALNVCKDLIAHRVYAVVVSHPLTGDLSPAAVSYTSGFYHIPVIGISSRDSAFSDKNIHVSFLRTVPPYSHQADVWVDVLKHFNYMKVIFIHSSDTDGRAILGRFQTTSQSVDEDVDRKVVVEQVIEFEPGLDSFSDRLMDVKGAQARVFLMYASKTDAEIIFRDATFLNMTTVGYVWMVTEQALDAANAPEGLLGLRLVNATNEHAHIQDSIYVLASAIRDMNTSEEIHAPPSDCDNSGSIWTTGRLLFDYVRKQRLENGATGHVAFDDHGDRVHAEYDMVNVRAQGEHVAVGKYFYSKETQKMRLELKEQEIIWMGRSTSKPEGFMIPTHLKVLTIEEKPFVYSRRIDDGSECTPEEIPCPHYNASEDTDQLYCCKGFCMDLLKHLSKAINFTYSLALSPDGQFGNYIIRNFSQPGAKKEWTGLIGELVYERADMIVAPLTINPERAEFIEFSKPFKYQGITILEKKPSRSSTLVSFLQPFSNTLWILVMVSVHVVALVLYLLDRFSPFGRFKLANIDGTEEDALNLSSAIWFAWGVLLNSGIGEGTPRSFSARVLGMVWAGFAMIIVASYTANLAAFLVLERPKTKLTGINDARLRNTMENLTCATVKGSAVDMYFRRQVELSNMYRTMEANNYDNAEQAIEDVKNGKLMAFIWDSSRLEFEAAQDCELVTAGELFGRSGYGVGLQKGSPWADLVTLAILDFHESGIMESLDNLWILRNNMLNCEENEKTPNTLGLKNMAGVFILVLAGIIGGIVLIVIEVVYKRHQIRKQKRMEIARHAADRWRGAVEKRKTLRAAILPSQRRAKSNGVKEAGSISLAVERGARRRDEPRVPRYLPAYTPDVSHLVV

>HarmiGluR6

MHVLVPLLLVCVSQCVSAQEGPPIGGIFYKDSEDMKAALEVSAKSFNFTASIKEVSTRGEVLEISKYVCQLAEEGVIGIIDGTGGRSSEIIQGLCDALELPHISIEHNDLYSDDWFVLNMYPSPTAYNMVLQKLILHKEWKNFTLLYTKGHSLIRVSELLQMGNDTLVVSLRELSGSDYRDVLIDAKHNGYKNFVVDSPSRYLEQVLLHAQQVGMMAEEHSYIFVSPDLFTLDMSRFKYGGVNMTGFRLVELQDKDNEKLWNFTSTLNLETGKTFKPEQLKTQVLLIHDAVEVFAAAFKKVKVQPEALSCDNYQAWSFGSTLLNFMKTNKVEGLTRSLIFDGVGQRTDITFNILELTSAGNQSIGNWTNNELKINRPLVADAEITQESALRNKSLRVLISLAPPYGYMRKSDKKLEGNDQYEGFTIDLIDKLSEILGFSYEFAVEDDYGTKTESGEWTGMALQLREERADLAICDLTITAVRQSGIDFSTPFMTLGIGILYKEPSKQPPEMFSFMAVFSKEVWYYMMLIQLALGATMIFVGRISNKEWQNPVPCIESPEELNNQFSFANSVWLIIGSVMQQGSEIAPIAIGPRMITSVWWFFTMVMVASYVGTLVAFLTVEKNVLPFETVQELYESKSITYGAKEKGSTKQFFENSTNPIYQAMFKKMKAHNWLAKENDIGVYWAETQNYAFFMESTSLEYYKERHCDLLQVGGLLDSKSYGIGMKKKSPYKKYIDDALLKLKENGEIEKLRNIWWKEKRGGGKCGEKRDADQKQLGMKNMLGAFVVLGVGCLIGLFISIIDMLWGVFKRSVKYSTTFKYELIEELKFALKFSGHIKPVKRPQKAIDGSFEALAKAEGKDDIRSLHSIRSCDTHRTHHSHSSRHSSRSLSVAFAKRRSYS

>HarmiGluR7

MCWYLLVLLLCVQKCTPQFVYTEMTEISYQIVGIFEKDATIQMAAFNDSLNHVHVQEVTIRPATLQPRRTDSYSVWRELCSNNAIQAVAVFGPQNPITDGAIRDQCAIANIPHIQATWQSMDPDLELNEETTAQEEGEDEESEIPFKKISINFYPDSEEIALAYGKLLQYYKWGGFAALYEDNFGLLRVQKILAEVSLQSQIFMYKLDPKGDNRRIFKALRKFQVSRFLLDCHSDHILRYLGEADNAKLVSEYQHFVLVSMDTSTVAESLIKMPSNITWLSITQYDKLKDGGHYLATRVGNWRSNEESPSVVQFKLDSLIMDDVASHLVKALDVKDIAQPPVFSCTGDEEPWAHGAAYQQKILKTQSYGVTGNVEFDQRGRRINYVLYINEIHIRDRQTIGRWESATGMINETKRLDSSAANQQSSKEFVVISRRAKPYFSFKEKCEKTECKDDDRFEGFSVDLVDNIFRILREEKYNYTYRFIHDYDMEYGKVDPTTHTWTGLVGYLLDKKADLAVCDLTITEERKKVVDFSVPFMSLGISILYTQDRKVQPGMFSFLNPYTFEVWMHTATAYCVVSIVLFICARISPADWENPEPCEKDPEELENIWTFKNCAWLTMGSIMTQGCDILPKAIGTRWVCGMWWFFAVIVCQTYIAQLSASMTSALENEPINSVDDLAKQTKIRYGAIVGGSTLEFFKASKDKTYRHMYETMAANPAVLVTSNDEGEERVLKSKNTYAFFMESSTIEYKLKRNCKLKKVGGELDSKDYGIAMPANSPFRTDINRAILRLKELTTLDKIKNKWWHEKYGAQKCEPTVDENDIEGDLEMENLMGAFVVLVVGLVFCLFITAIEFMNEVRNIVVREQVTHKEVFIKELKASLNFFQLQKPVLRNPSRAPSIASSDSDERRENQAKAIENFMNLEKAV

>HarmiGluR8

RTSPAPPPPRAPSSITAALVVPHKAFGTRDYTKAEKAALSKLPRKLKLFSHVRLNITLSTQGLTPSPMSILDSLCKEFLAVNVSAILYLMNHEQYGRSTASAQYFLQLAGYLGIPVIAWNADNSGLEKRASHASLRLQLAPSIEHQTSAMLSILERYKWHQFSVVTSAIAGHDDFIQAVRERVTALQDRFKFTILNAIVVKRSSDLNELVTSEARVMLLYATREEAAEILSAAGDLHLTGENFVWIVTQSVLGSMQQPNKFPVGMLGVHFDTSSSSLIAEIATAVKVFAYGVESYVSEPENIRYPLGTRLSCSGAGAGEARWSTGERFYRHLRNVSVEGEAGRPSIEFTPDGELKAAELKIMNLRPALGEQLVWEEIGTWNSYPRERLIIKDIVWPGGLHTPPQGVPEKFHMRITFLEEPPYINLAPPDPVSGRCSLDRGVICRVAPEVEVAGLEAGAAHGNSSLYQCCSGFCIDLLQQLAEQLGFTYELVRVEDGRWGTLHHGKWNGLIADLVNKKTDMVLTSLIINSDREAVVDFSVPFMETGVAIVVAKRTGIISPTAFLEPFDTASWMLVGAVAIQAATFSIFFFEWLSPSGFDCSTGNNSKRVPQNRFSLCRTYWIVWAVLFQASVHVDSPRGFTARFMTNMWAMFAVVFLAIYTANLAAFMITREEYHELSGLDDPRIARPLTQRPPLKFGTVPWSHTDATLAKYFTEPHAYMARYNRSTVSAGVTCVLTGELDAFIYDGTVLDYLVSQDEDCRLLTVGAWYAMSGYGLAFTRNSKYVSMFNKRLLDLRANGDLERLRRYWMTGTCKPNKQEHKSSDPLALEQFLSAFLLLMAGILLAALLLLLEHVYFRYLRSHLAASSAGSCCALVSLSMGQSLTFHGAVVEAAARGFGERSHCRSAVCAAQVWRARHERDMAVARARQLA

>HarmiGluR9

MATRDGTRDLKLLSKNTSDSGEAAGAPHAAQTTYCIHKPTPASTMITLKAFIIAISTVCLFIHNSHALRLTAFPIGGLFNRETLPSSLQVFSNMIEANRMTTYHGRSLVSKVVDSYSTSLELCPFTSEDRGIVALIDARPTYGICDITCLLCNRLNITHLSLGWEPTDTQSEDFFSFAYYPPPEMISKAYATLIKDLGFDKFTILYEDDGSFIRLQQVINTWPTTMEPILFRKLDPYGDNRETFKYIFKVARMSYHVLDCNITNIHKYMNEIVQVENATEFQSFILTNLDAYSLDFKAIDDLMANVSTLHLTTPSEATWKDKGMLGARAFGLETALAADALSHLEKAIRSLQLTTDNLPDPPPLCQKSTRSEYEESAWPLGYDLREALIKTTTKGFSGHIEFDSEGRRTNFMLHYSKLDKESQFVYAGDWDYKTNVITKKDHVDDRSLALKPGSKIRVVTKTGSPYFSVVDTPEGKVYRGYCVDLIDAIFKYIKETRKEHFEYEFYIAPGNEYGNQIEGTNKWTGIIGELMDHKAHLGICDLTITSERNSVLDFSIPFMTLGISLLFREEDPEAPDRFSFIKPLSLDVWLYLATTYVIVSFVLLVCARMSQDDWVNPHPCNQNPENLENIWSLYNCMWLTMGSIMTQGCDILPRAAGSRWIAGVWWFFALIVTASYTANMSTFLSNSRRSNVINDVKELSEQNKISYGAVYNGSTYRFFQTSNDTVYSKIWSVMNAAKPTVFTTSNLEGKDRVTRSNGKYAFFMESTSIEYYIKRNCTLKMLGSKLDSKEYGIAMPKNYGFKGKIDHAILSLQELGELEKLKKKWWEDEDNQEHCEKIVKEEDDNGSLQMKNTSGIFLVLGTGGILGLIVAIIDFMFHARQISVKEKVTFKEALSSEWHASLNPRELHKPTAPPRSAPPSTASPSPQRERSQSRAVSVLRAASSFINFDEIY

>HarmiGluR10

MLLRGVYVTVVTIILILGNVGAVLRKFETLKTTVNIGAIFPPNTVAEVAFASALARASMESEHYHFVMKAVYSPYGDSFAASKAACELLSGGVIAVFGPTDPVSASAVEAHCRAARVPHIQAVWRPPPVRGEQQPSPPGINLYPEAVALSKAVALFIDDSDWRSYTLLYDDDHGLIRLQEVLKHTNPEVKWLVRRLVPGEDNRPLLKSLKGTGETRVILDCPADRVLEYLRQANEVKFFEDYMSYVLMSLDAHTLDLEELRYGLSNVTCLRIFDHSDSRTRAYLADWKVRGSDDVKIPRQSYEITVEAALASDAARLITDAVENAPEEFKLEAQEISCDSEDQWESGEDFTNHLLTNPIVGITNKINLDNTTGERMNFSVEIMELSNSGFNSIAKWNPEVGFEYGRSADETSDRLAEKWQNKTFKVVSRIGAPFLVEVVPKEGETLTGNDRYEGYSKDLIHEILKELLHLNYEIEIVPGNGYGSYNKETKKWDGLVGHLLERKADLAICDLTITYERRSSVDFTTPFMTLGISILYLKPTPPEPELFSFLKPFSVDVWIYMAAAYLMVSLLLHILARLAPNDWENPHPCDKSPEELENIWHIKNSCWLTMGSIMTQGSDILPKGYSTRWVCGMWWFFGLIMCSSYTANLAAFLTNAAMDDSIKSAEDLAMQSKIKYGTLIGGSTYSFFKRSNVSIYQRMYGAMESARPSVYVKNNDEGLERVLKGKRDYAYFMESTAIEYQLERHCELMQVGGLLDSKGYGIAMPFDSSYRTAVDNALLKLAESGKLVEIKNRWWKAPPEKACVTEEASEEGAAGELGVENVGGVFVVLGTGCGMAAAMGIFEFLWNVREVAVEQKMTQSEAFWAELTFALSFWETEKPVKHSRPSSSASESQGASRASSVLRSAADLFHLDVFK

>HarmiGluR11

MMALKFILNTFKYILLLSYLAIYCNNIEVSSQIPMADKIPIGVVFDQNTEEIQNAFKFAMLQHSNANKSSLDFQLYVDIINTADAFKLSRLICNQFARSVVAMLGAVAPDSFDTLHSYANTFQMPFVTPWFPEKVIPPSSGLNDYAVSMRPDYHRAVMATITHYGWKNVIYIYDSHDGLLRLQQLYQSLQPGNATFRISNVKRVSNASDVVEYLRAIEKLDRWSNKYVVLDSTTQLAKATLILHVRDVQLGRRNYHYFLSGLVLDDRWEKEVTEFGAINITGFRLLDFSRKIVRDFIDVWRRETISAQAALTYDAVQVLIDAVLRLMRKKPDILRATLRRASQNNSKIMDCNPKNKLIPFEHGEKISRMIKKTEVDGITGSIRFNEQGHRKNFTLQVMEMTVDGDIVKVATWCENKGLIPVIPKLDHPAVPGSYDRNKTYIVTTIEEPPYIIREDPEDPEFRPDEPFKGFCADLTKMISEKLEINYEIKIVRDGKYGNEDPKVLGGWDGMIGELIRKEADIAIAPLTVTLERESVIDFSKPFLSFNLKPNVASNSTGAIFSFLQPLSMEIWLSIVCSLFAVSVVLFIVSRFSPYEWRVVSFTDSHITEHSDLATTKTTVVNEFSFWNSMWFSLGSFMQQGSDINPRSVSGRIVGCVWWFFALIVISSYTANLASYLTLSRISEPSQTYSKVAMCPEDTVDGPKPTIEVPQEPPVDEHGWLAFLMDRSAEPEDKPCEMIVTLTNSGYKDFAVGVPKGSQLRDGVNMALQSLKEEGEIPRLVRKWFTKSECDAPDTDIKGSELTLSQVAGLFYVLVGGLTLAMAVALFEFCKHGRAEAARANVPLRAALQAKTSLSSNTERKMTQSQRGPQREHDRLGWNGGAFGGQYYSPATQIGQEETALHSSFTQV

>HarmiGluR12

LPLLLVDAAPADAAWEALELYPHPQVLAQACAELCEAKGWTRAVLLHTGDARAAALLTARARPLALLARRLPPADDDALLRNLLLVLKKSGLTNFIVWCDAACAVRVLDAAQRVGLLAERHSYIVLSLDLHTQPLHDYSHGGANVTGLRLFDPEAPEVVEVMEQWRKMYAERLGVAADDEGDGDSPEAEEAAAIAAAPPTALLLAHLGTELVAEAWRRLQLPPAAPADCAMGAGAFHADTLLNYLRSEEWSSEDGAGRLVGGAVAWEVDGARREVQLQVVELARGGRLARAGLWAPRAGLSWQRRDPPASDPPPDSMTNRTFTVLIAKSDPYVMMQESTERLTGNDRYEGFCIELIDQLARLLQFNYTFVQQEDGDYGSKDNVTGKWSGMLGRLMEDKNIDFAVTDLTITAERERAVDFTTPFMNLGISILFRTPKQPEPKIFAFLLPFSNGVWLCLGFAYLGTSLLLYVVGRLSHEEWQNPYPCIEEPPALENQFTLANALWFNLGAVLLQGSEIAPVAYSTRAVASVWWLFALVITSSYTANLATLLAKKSSDQVITNVQELADNQLGIDYGAKHGGSTYTFFEHSQSELYQRMFEHMRTRKMPASNVEGIAKVESGKYAFLMESTSIDYLTERNCGVTKVGSLLDSKGYGIAMKKNSPYRQAMNLGLLNLQEAGTLREMQHTWWKEKHGGGACKVSPDSPPPHTPSQEPVLMLLCVQVDEEHESEELNMSNFLGLWLVLVVGSALGIVLSCCDLAWAAARRARASGRRFHTHFWEELRFVFRFEQSVKPLQGPLSGTPSSSARSEAAEAEAEAEAEAEAEPEETPEPRARSGSGAAGARRRRSSMHAASLRLARHTNRDSATPARR

>HarmIR1.1

MHQARLRKMILIGVLFGLMQAVSGFNLDVANLAADYVKHKDLRHVCYFTCQSRYYNTILVHKLTKQSVRVSVRRIDESVNRDVVRAASRSTGAVGLLLDAHCRGTPLVLLEASKNKLFDAMHPWLILTNIEDADNCTDYIQQSFQQLNLSVDADIAVASYNGGDNYTLTDVYNFGTIQGNNLEVNHLGSWRPETGLEIKLKGYKYYNRWNFQNLTLRAISVIVDQPEMFYPEMLSEMTYTAGVAAMTKITSQMLNTLKEQHNFRFNYSIAGRWIGSPKRNSTLAVTNALFWEEQDLSSTCARIFPKWLDWVDIIHPPTTNLQTKFYYLIPETGVGQYENRFLTPMSHGVWGCAFIAGIACTLVLTGAAWMESRPKPGLYAFFSVFAAVCQQGYEDGVQLLESYSSQGRRLTLLVIGLTSMLLYNYYTSSVVSWLLNAAAPSIGNLDGLINSDFELIFEDIGYTRGWLDNSGFFYYSGFKNVKEDELRDKKVTKAKRTVPVLQTVNTGVELLRTGKYAFHTEPYTAAQVISKTYEDEELCNLGALQMMLPAHVYIMAQKRSPYKEFFDWSLLRLLERGHVKAIRARFAGTMPACSGARPRALALGQAAPAFLMLLLCVLLSWIILAFEVLWSRVQLKKRGP

>HarmIR1.2

MISYQILLVLILPCIIFGLNQNTLKLGIEYFKYRDVKFVCLLTCEKYSSWALQYSKSASTVSIAVSETSILRSKVNYNRVENCLRRKTYGLGVIIDTNCEMAADALYFASQNMWLDSHHKWLLIDDDKAKLEVYNDTEIENVIYEDRNSTLIDILSNLNISVDADIVVAEKGNSSYNLYEVYNYGKIQGGNLIVNEIGFWSHENGFNLNINLNGYKYYRRWDFQNISMRMILVAQRASKNFDLESLTGPEPVPGVAIITQTPTDILYIVAKIHNIRYVSTITDRWIGSYEKNSSKVVSTSLYFREQDVSPVIRGLSTVYERIDVINPPLTSIETRYYYRIPTMGPGKFENQFLRPLSTTAWWSVIGVSTLCAGLLLLSALLEQRPSSVQYAVFSVVASLCQQFFQDIDDSGTKRISTARKVTILVTGLSCVLLYNYYTSSVVSWLLNGPPPSINSLKELLESPLELIYEDIGYTRSWLQSPSYYFNKRNAPIEDELRQKKVFNKKKNAPLLEPLVQGIKMVQKGGYAYHTEVNSANALISRTFSQSELCELGSLQSMEKTLLHPCLQKHSPYKEFMTWSLMRLSEQGIVSCIQIRRSSFEVKCEGSSPRALALGGAAPAFILLLGGYMLATVIMLVERLVFKMKHENQVK

>HarmIR2

MRTANVVKSSLDTIFIIIIHKILLLSAYDNADICKLVSIDLLSGITKYFYTSKKFQYKEQFKIGFLRIKESGLLNRLVSTDFEEPKCTKSHIIQISMQHIAIPLTILAVTSVLSTIIMVAEKIHYNRNMRWPYFN

>HarmIR2.1

MALVNMTNVTLRVATQYNCDSLRNRARTSDPHTFDPMWRETILGCMILDLLRDMYGFKIPWIAATTAACGLRQSSAARDNSQPMKMYGAAAEARDSRARPTNFGCCIFFSMCYVFDNPERNVTVIGGLNNASQSYRDSVDVYSRSMTLTQDLAEQIYPIHALHTWKLGFLLSRPGNKIFSTFYSKPFSRPVWNCLYCFGLLIIVFFYILKRWEFSVIGGWQICFVYEALLVVGAYCQHIPPIDPRLPSRRIAYLIFFTFVYIVYTYYTSNLLSNLVNDKDHGIDLPMLADSDYVFLAVNHMMMAIFERSQIYHYNRNISLVVKKLMRIHTVSIPDGLEAVKTGKYALLSDFITVYPYMKKIRTLNVTQARPLVAFPVKQHFGALGRVVVVSAASAELSLDRVFVESSCCTHLETALRHV

>HarmIR7d.1

MEVRDTEVNHTLEHFVTEDPASELGILAAKVAYYNFEWRFLTIVMYNTVQAIGLNTFLMHYEKSVIVKLGRFLPARRSAVPQMIIFGEDASEISSTIRWTVRAKYDSNGKFIIICAHHEQECDELKIFQTLQSLYMFNAVVLKTSNKTKESLAYSYDFLSEGKCKNSVPYKVNLTTDCFNDNCFKNLYPERLSNFRKCPLIMSTIEQPPFMYLHNLTSKPTGIDGDIMRLVADMLNATLHLKPPYDGADSGHFANNNWTGSLGDIYNNHSHASVCSAPITSGKYGNFQISFTYYSMDIVWATRLPAQQAPWQKLLHPLNIYIRIILLLMFICIIFMNTFCKTNLFKVISKIRDVFKIAPPKYSLLFYSWVVFLGVPILRTPERRSFVVTVYTWIWFCFIMRSAYQAALMNSLKNPAYLDNLKTFQEVLKENYPFGGLDSLKEYYIDDRAIYDKWKVVELKNLDKTLDDILEGSTDFVLASNKEFIKHHIMKYNGTKQLQIIPQKIVNSPTVVYFKKFSPLVPPMNFALRIAFEAGFIQRTYVRYLDHDKKLLQRLRSKQAEPLSMEHFAGCFVLLVLGWFVSLTYFAVEYICGNLDDE

>HarmIR7d.2

MSNQMLYGHLTANMAISELNHFFDTNVTAGSQLGDMAAKVAIQNFDLRHATMLFFNSTLCYGVEVFLQFYHYNIVINRAKVLARKTTRQFVLFASDTADIELLLDSIISFEMDNTGKFIIICESSTPKECDEQDIMVLCWNYRIVNMVFIRQEETEAVGFTYYPVADGICNNLKPIKLNSHNHYTKTTYGEIFRKKFRNLNFCPIIASTFIQPPYMYIKNGIPTGIDGDLLRMLIHGMNASLKMMTPSRGTGWGFREKNGTWMGSLADVYDDLANFSMTSAAITLTRFTDFQISSGYSTSKVVWVSESAQVQNVALKLLHPFEKNTRFLLIASFLLVICCAFVLKSSCWTAMCNEENQSSRSVVFYSWMICMGQAVEKLPTKSAYVQMTLFFIWYCFLVRTAYQVYLISSLKGRFYDSQFESIDEAINAQYPFGGGPALKDYYVDYPFVYKNWVNIDTQQIAPTAVNISKGMNFVLAMNIDAARVVMKTRKAKLHILPEPIIISPTVLFFKKYSPLSETVNTILHRLIAAGFPDKLYKIYSSTFDIVDQGSDEGETLKMSHFTACYVVLILGWIVSAIFFSLEVYFGKIYKHPS

>HarmIR7d.3

MIWVLRILVILQLMLRSCSASSNLIETAVNISTTTFNLHFTTTVIWKQNESECVTGYLQSYPGSVVLSPWATYNDSKVREINETIGFKQTIYFATNLLEYEIIMELINEVIRFPIRFILVLENPVKSISELSAFIEVTARNDQADLILISENDAGEVSLSTFFPYSEGLCGNYTPVFLKYGDDLWPKKFSNFYQCPIRTALLEYFPYVTVHFEKGKITSVGGYDGKILMIILKKLNASLEVTSAYNNVFGTYVNGTATGSIGDLATEKADILIPADILTEKRYTVTLPSHTYHTVDIRWVGPKQREVYDWLKFIIPDKTNFTYLHLLVYILFVIVAMLVRKCKPHLTSATNRILYQSFIILLGQSARFVTKSWLLNSLFVLWIWFCFFFRIDYQADLVDALQTLDLEPPFESIEEAVTKVDGYGGVEVVVDYYKDTPLERNYKVIPMNELKNYIRRIVEGENFILATDIALVKLLEPYVQILKKRISATGACFYMRPGWPAAKDVDDVIFSLVEAGFIENLLSDNNNHRWIVNRMNADDVLQPKSLSVEKLSTCFYGLGIMWLICFIILLIEIVHHNKHHNK

>HarmIR7d.4

MFVDFSKTLYFAIFFLKIFSCSALNDITQCAVEVANYNFFYRLPAVILYRDVNDKLVTDFLKAYKGTVMIESRGVEPAKQVVIIVDNYQSFIRLLSMLKPDLRGRNLLNGRVKLLIVINSNRRKLDHINGILWSYYVTDVVIVTKDKQGRIALYTYYPYKNHLNCQNTEPVLIGYWSRNMSYSNMYPDKMTNMHECPLYISTNKIYDQATERKIPLQTIKKAIVRLLRDIMNFTPIVSARDYISIDSDRAKNWSDSLNDVISGFANISTCTIPLGVDRLGLLDYSMPYFRIRIAWLAPPVAPGPVWWRLLSPLNGYLWLILLVVTFLVISLPFVLKLKRIKYFCHRYFKNFDKVQGAAFRVWGALLGQTIRVAPRRFRDFYIVGLWLWFTFVVRNAYQSVLIGALKTDTLTGNFANLKETVDNGYNFGGRAGIYSHFEFDPLIRDGFEIIPEVKFEEVFRDLIDGKKKFVLAASLEYAYAYCLAQGKKENECGHVLPDSILTVPLVVWMKMYSPFVRPLTTWLPRMIESGLLEKDAVLKTSYVTTITSDPSPLTRHQTLSCFLCLGFGCLISLVILLLEIVRKKTSEYTVVKKSQLVELDRESKFVIDF

>HarmIR8a

MSFHYLFLLIFLINLGCVLSELSLRFVFIIESQEQDLTHEISKSLKLAESVRTDVKLDDAIVVLDRETEDESYRMLCSSLSKGVSMIIDLSWTPWEMAEDLAAETGVPVVRTLLGSQQLVKALDEYLESRNATDAAIILESESDVDRTLYELLGASNIRVWVHAGLTRDSAKALKTMRPEPSFYVIVGENGFIMDTYRRAVKEKLVRRDYRWNLVLTDYSTLELSQLVLPTVTLQPDPGECCKLMKREDCSCPNDFQRKQYILNALIQYIAEVYSKLDRDLPLVTSSISCEEPEAVMNSTRDRLYRQFAEDAEISNETLFYWDMDRSGLFLRSRFILSTYKPTAGQQTIATWSADEEYKLLPGVELEPLKMFFRIGTAPAVPWTLMKLDPETGEQMYDDDGQPLYEGYCIDLIARLSETMSFDYEIVSPKSGGFGKKLPNGTWDGVVGDLMRGETDIAISALTMTAEREEVIDFVAPYFEQTGILIVIRKPIRKTSLFKFMTVLRTEVWLSIVAALVLTGFMIWLLDKHSPYSARNNPHAYPYPCREFTLKESFWFALTSFTPQGGGEAPKALSGRTLVAAYWLFVVLMLATFTANLAAFLTVERMQTPVSSLEQLARQSRINYTVVEGSSVHQYFINMKFAEDTLYRVWKEITLNATSDQAQYRVWDYPIREQYGHILLAINASEPVPDAKTGFQQVNEHTDADFAFIHDSAEIKYEVTRNCNLTEVGEVFAEQPYAIAVQQGSRLQEELSRALLELQKERFLEQLAGKYWNESARQACPDADESEGITLESLGGVFIATLFGLGLAMITLAWEVFYYKRKEKNKVQTLNTKPEKVAFESKSTLETKVAESVAKLKKRGKKGNLAKNVTFGDTFKPVAEKGVSYISVFPKDYRP

>HarmIR21a

MAVSWFIVIFLLFYPVYGEEVLVEYYPSQSILDTQHKVKNKREIKSNAAAIALDNSNNVSTREINWRKFNQEKTDDDAKIKKRALDPVFRGHPKTREELWNEHFLNETTKFDQTPSLVHLLHNITLTYLKDCTPVILYDSQVKSKESYLVQNLLKGFPMSFVHGYINDGGELVERKLLHASTDCQNYILFLTDIKASAKILGKQPKNKIIIVARSSQWAVQEFLASVVSRMFVNLLVIGQSFKEGDDANLESPYILYTHKLYTDGLGASQPVVLNSWNHGKFSRNVNLFPPKMTEGYAGHRFLVAAANQPPYVFRRIKADLDGGNPRVAWDGVEIRLVKLLAERNNFSIEIIEPLELHLGSGNAVAKEITSGRADIGIAGMYLTTDRIRDLDVSQAHSQDCAVFITLMSTALPRYRAILGPFHWHVWVALTFTYLFGMFPLAFSDKHTLRHLLHNSGEIENMFWYVFGTFTNCFTFLGKNSWSKTDKITTRLLIGWYWIFTIIITSCYTGSIIAFVTLPVFPETVDTIKQLLAGFYRVGTLDRGGWEKWFLNSSDPQTKKLLNKLEFVPNVEAGIRNTTKAFFWPYAFLGSKAELEYIVQANFTATKSKRAALHISNECFAPFGITIGFPNNSVYSEKMSLDISRMIQSGIIDKIADEVRFEMQRSVTGKLLAAGSGTIKIPSAEEKGLTLEDTQGMFLLLGAGFIIAATALVSEWMGGFTRRCRFQRKVDTPISVNSREHLIPTPKTDIGSEIKIIGDTESRLHFDSRPSTAASRDTLEGQIINVTEDNIDVHNSFNVDRFDSRRSSSLDLDREVREIFEKDQKRRRIVSQDMESVDEHGPTVSRVAFGDPIKHEK

>HarmIR25a

MSTVTVLLLFNLVHIAFGQTTQNINVLLINEESNALAEKAFEVAKEYVRRNPSLGLAVDPVIVVGNRTDAKSFLENVCRKYNDMLLAKKTPHVVLDFTMTGVGSETIKSFTEALGLPTISGSFGQVGDLRQWRTLNANQTRFLLQVMPPADILPEAIRAVVTKQDITNAAIIFDEFFVMDHKYKSLLQNIPTRHVITPVKSFEANEIKTQLESLRNLDIVNFFIVGSLRTIKNVLDAADKNQYFGRKTAWFALSLEKGDISCGCKNATIVHMRPTPDANSRDRLGKIKTTYSMNGEPEITSAFYFDLSLRTFLSIKSLLDSGKWPNDMKYITCDDYDGKNTPNRTLDLKTAFQEIKETPTYAPFFIPQDDPMNGRSYMEFSTDLLAITVKDGASISSHSLGSWKAGLSSNLTLTDPNNMSNYSAQLVYRIVTVEQKPFIIRDDKAPKGFKGYCIDLIEEIRQIVKFDYEIILAPDGNFGTMDENGNWNGIIKELVDKKADIGLASLSVMAERENVVDFTVPYYDLVGITIMMKLPRTPTSLFKFLTVLENDVWLSILAAYFFTSFLMWVFDKWSPYSYQNNREKYKEDEEKREFTLKECLWFCMTSLTPQGGGEAPKNLSGRLLAATWWLFGFIIIASYTANLAAFLTVSRLDTPIESLDDLSKQYKIQYAPLNGSAAMTYFQRMANIEEKFYEIWKEMSLNDSLKEVERAKLAVWDYPVSDKYSKMWQAMEEAVLPNTIEEAIQRVRDSKSSSEGFAWLGDATDVKYHVMTSCDLQSVGDEFSRKPYAIAVQQGSPLKDQFNNAILQLLNKRKLEKLKEIWWNNNPETMKCEKQDDQSDGISIQNIGGVFIVIFMGIGLACVTLGVEYWWYKWRKRPVVGDVTQVEPAKSTRNNVDKQGEGFTFRGRNLGLTFKPKF

>HarmIR31a

MLAQAVVEFFKNKVVSSIIVLACWPAVDQMKFTRQLSQYGMSATFSCEPAILDKIHPQYLQGVLYMVRDDDNLAFFKKVKRVHFTMKYKWLLLGDHVPEALRDIRYDSDVTFLKWGRNNRNETKKSTNLALLPEPPTSPIYFYDVYVHLREGISIHYWAHWTKESGFVVTHDREKILRRLDLQRYPLRIATPVGHYSSDKYDGTFVDYLKDETMLDQDPGIRSAHSASMLLTEAVNAQDVLIENELWATVVNNNSMFLMVSNGQADLSGGVLRLLLDRSFKLDYVTPLWPFRVGFTYLAERESSSNMYLEPFSPGVWWSCLGIMAILAMVEWITAKTPKEREGALYTVLTTWLQQDASAVPEGASGRWAFTVLSISAMLVHAYYTSAIVSALMSTGRGGPETLKALGDSKYAIASEDYDYMRYLFFDVETTWDDLEYLKKKKKTSKFYQELERGVELIQQGNTAFHSEYNQIYPHFKTFSDDHICKLQHIDTIPEILTWVTTTKHGQWTEVLRIAGSWLQETGLRKRLVNRLRIPQPPCRASLLAERVKLGDIAPLLALTMLGAILSVVLLGIEIMYAKAKGRKLQEGDAKHALEDSDDSSEVTDVVYNYLA

>HarmIR40a

MELLLLFLLINGVQSLLDIQDVTSDVMTSLPKDFSIAVKDIAEGMPTKAITIVRGNSTNIRSQDIFELLCLLSQHKVLTTNLDIATKENKQKYYEFLGKALDVSDQRTSLILCEPYECENILFELTENNLIHSMILYFFYWSYGPVSDTFLMTMKEAMRVAVITNPRESVFRIYYNQGTPDRLNHLTLVNWWAGTLYKSPVLPPTDKVYHNFRGRVFEVPVLHAPPWHFVKYNNDNTITVTGGRDDKLLALMSKKLNFKYRYYDPPDRSQGSSISSNGTFKGTLGLIWKRKADFFLGDVTMTWERLQAVEFSFMTLADSGAFLTHAPAKLSETLAIIRPFQWEVWPLVFATLLVTGPALWIVIAAPSLWQRRECDQLGLFSSCCWFTTTLFLRQSSSKEPSSTHKARLVSVLISLGATYVIGDMYSANLTSLIAKPSRERPIGTLQALEEAMRDYGYELVVESHSSSLAILENGTGVYGRLANLMRRQRVQRVRNVEVGVRLVLSHKRVAVLGGRETLYYDTERFGSHNFHLSEKLYTRYSAIALQIGCPYLETFNNVLMTLFEAGIIAKMTTDEYKNLPEQARRSDPVTESDKQGNELMGDSTTASQAPQSESAKGLQPVSLRMLRGAFCLLGIGHLLAAISLAVEIQLHRRSKRRHEPPPSEHRKTQKLLVLGKSVMLFKRGYKRVCATVYTGIDKALGPEVKD

>HarmIR41a

MLVSTPALAPLEILLNTIINRYLQSAYCITVFSETPFTFILPTSFISLIPNETILVEQIFNVSETGCSDYIVRMRDPQIFMEAFERVVHIANVRRSDRKIIFLPYDEEYNEENDVNLPSLVFAMKGSKYLANMLMIVNHASVNQDCKIFNLVTHQFVGRSEEATHLPKYLDSWDSCTQKFENEANLFPHDLTNLYGKVVRVACFTYKPYALLDIDPAIEPLGRDGVEIRIVDELCRWINCTVEIVKEDVDQWGEIYANESGGIGVIGSVVEDRADIAITALYSWYEEYRVMDFSVAGVRTAITCIAPAPRLLSSWEMPLMPFTWYMWLAVVFTYFYASTGLLTAQGCSTTTYPFFNVFGMMIGQSQYESRASWRIRGVTGWLLIAGLILSCAYGAGLASTFTVPRYEPSIDTVQDIVDREMEWGATHDAWIFSLTLSTEPLVKQLVSQFRIHSFDELKRKSYTRSMAYSIEKLPAGNFAIGEYITQEAVLDMMLMLEDFYYEQCVVMMRKSSPYTEKVSQLVGRLHQSGLLLAWETQVALKHLNYKVQVEVRLSRSKNDVGITKPLNLDNVVGIFIVYAIGLTISFAIFLGEIYVHQRKKKNEVLHID

>HarmIR60a

MSVDKIFLVNVVIFIQVHISDGVVNPFGPTVVNDYTNCISKVIDNEFHEPGLLIFANTNNVSTSVTRIRTKLLKRLHKEIKFSIEIMSPNNEVEICDLDNYNLGVLHVDVYVAIPFANYFVIIIDSYTDFSFLASKLIRSRSWNPFAKFIILLFNFVQDDKVNIDYVERVLSCLFKYNAINVIIAVPKANNFRNAIIYSWRPYDPPKYCGYFNETAKDRLVVQNMCESGVLKHDRKVFDNKIPHDMEGCVIEVLALQRHPFISDDEYDANIEKLMIDAMLKRFKMKARYNFIDGYRGERENVGEWNGGLKKLASKSGHLLLGGIFPDFDVHEDFETSVTYLADAYTWVVPRAHKSAAWVALVIIFKSLVWYSVIAGFFLCGITWKIIAELSEDSDYNRSFRHCFLNTWITVLGFVSYLHPVKESLRVFFVFLNIYCMLFSTAYQTKLFEVLTNPSYEYQIQTVEELVESGLKFGGFEELHDLFYNSTDPFDYRIGDQWTDITNITEAMIDVAVHRNFSLLCSRLELAHISGITPELSDSVGNYKYYTFTDNVFSVPIETIALRGFPFMMEFSTTITIFKQSGLNEGLRQHFAHFNERRRARQLRALLKEKSDVNPLSSEHLQGGFLALALGYVSGTLALIVEVIVNCDYVQNKFANFKRRVNLLS

>HarmIR64a

MNLFCNLLLTLSVTEVPLVIDILKHKNIKNAVLFQCYNNHFVSGVHKIFNENNVLIASSKIISNGTYIIPLDRKKTGIVVDTSCEGWNSVLDSRTVSFKDYSFIIIAEHLSPILEALSRYPILVDSDVIVAHKLNQSYNLYEVYNTGFKLKGKYVIRLLGHWNSSLYIEDLNRWDLGGAFVKTAVVVINPTKLTNQTTEQYMEKPIRMQVKVDTVHRMKFFILLKYMRDMYNFRFDMHRVHSWGYKRNGTFDGMVHALYCGQAELGGAPIFYRIDRWELVQYVSEVWTSRHSFIFRHPKYPGGFYTIYTRPLSGIVWYCVIAMLVVTAVILWAMLLVQNTWGDNEDSSLSLAGIIIWGAICQQGIAINRESTSTKLLIFITFMYAVTLYQYYNATIVSSLLLEAPRNIRTLKDLLDSDLKAGAHDIVYNYDYFKRTTDPVAIELYHKKVVTATQHNYFPAEKCMDLVRRGGYAIHIDTSVAFPLIKATFNEREICDTTLVQMYPQQRMGVVMRKNTQYREHVAYAIRRFSEAGLPQRLRSDVDEPMPECAHTPDSSVFCVGIREFSTPLLVLALGMLVSVLLLICEIVLHRVVQRAGLRDFVH

>HarmIR68a

MFKIIVLFCFFTAVKLDTFPILKDLHERKDLEYVLIDLINVLMREYEVTCIAIICDEVYLNVFSGPLFKRTSAIPYVMTVVEDYEDLLSPNFVTLESLRAARKEGCNVYVILLANGLQASRLLRFGDRHRILDTRAKFIMLHDFRLFHSELHYIWRRIVNIIFIKHHNKMTGTAKSRPWFELSTVPFPNPIKGVFVPRRVDIWKNENFHYKRPLFADKTSNLNGEVLNVVYLDHVPSVVVMKNNVSTKLGGVEVEILHTLAEKMNFKPRAYQAANAEIHKWGQKQPNGSFSGLLGEMVNGRADVALGNLQYTPYHLGLTDLSIPYTSQCWTFLTPEALTDNSWKTLILPFKLYMWIAVLLVLLITGTIFYGLAKNHMNLQEYRRKSKSKRSNIDDENTKPGLYLFGEIINSILYTYGMLLVVSLPRLPMGWSIRLLTGWYWLYCILLVVSYRASMTAILANPAPRVTIDTLKELVDSKVTCGGWGTQSKKFFEQSLDEYSQRIGDKFETIDDPMEAANKVAQGVYAYYDNSDFLKYISVVRKNSFMDPKQNNTVNNTEVTGRKDTQRNLHIMTDCVVNIPISIGFHKNSPLKPLADIYMWRVVEVGLVEKWLNDAMYQIRTLETSEDEVKALMNLKKLYGAFIALAIGYSLSAICLIGELIHWHIIVKRDPNFDKYALHLYYLHKNKKH

>HarmIR75d

MELISFILSYFITKDLSMMTAFICWTPERTSELCRSASGAGVRLQLATDFHQPPPMTPRGRFREAMLLDLTCPHAALIMEAASSSRGFNYRHSWLLLHNSSERGLIADALSPYEILPDADVVWSAPDALLDVYKIKPATPYLLTDLGLTRNCSRHQLRALWGALPSAVTRRRDLKNVTMKGISVVTEPYNFKGWSDLRNRQIDTFPKFTYPLMMLAAQDMHFRFDLRQMDVYGVSHNGSFDGLVGRLQRNDAEVGLASIFIRPDRMQVADYISETCVLLCAFIFRQPARSAVSNVFLAPFSAGVWAASAGVAAAAALLLVALRAVLQRTRQRDDLALFTLPETLTFALGTLCQQGFHTTPGVTSVRLVMFSTLLASLFVFTAYSAKIVAILQTPSDALRTIDDLTRSPITIGVQDTTYKKVYFLESPDESTQQLYRRKILPQGERAYHSVVDGIARVRTGLFAFQVESSSGYDIIRQTFTEREKCSLKEIEAFKLPLVAVPMRKNSGYRELFATRLRWQREVGLMSRERRVWLVSRPRCDAAGGGFVSIGIGDVLPALQVLGLGALISLTLLAAELAAHAIRNSRRWNHI

>HarmIR75p

MNIFTFIFLLLIHKSVYAKDANTINFIKLFIQNDQKPTHLIYGGLCWKKNIINKLVVELSTIGVRTSASFKPKSKYQDHAILYLTDLDCAQSKAIISYALSKELFQFTYRWLVLVTSKQLQQSKISLLMNGSVLVDSDLVLAERAGNHFKMVEMHRPGLNGSMITTPRGFYNGSFVDVRPHRELYRRRRNMMGHPITMSNVIQDSNTTREHLPKEDRLELQYDSITKACWSAAKIGFEMINATAKYIFSYRYGYKVDGQWSGMIADLYSNKADVGTNCVIFRDRFDVVTYTDLVAPMRMLFIFRQPPLAYVANVFYLPFSTRVWVTIAVCTAIATVTLYLASKVELVLTKASTQQQLDGGICDALLLTMSAVTQQGCYLEPRRAPGRMMVFVLFTALMALYAAYSANIVVLLQAPSDSIRSLPQLANAKITLAANDVDYNHFVFNQSKEPLHTSIRDRVFPENGKARLYSLADGVERIRKGLFALHSVAEPVYRQIEATFLESEKCDIATVDYLVTFDSFTPVRKGSPYLELIRVVHKQIRESGIQSAIRKRFLVSKPHCTTKMSSFSSVGLMDMKPVLILMLYGVCLSVTIAAAEILVFKLSEHRKKYKVESTEPPSP

>HarmIR75p.1

MNLKILYLLSFLYVFMVKCDGNFNSDVIMSFVTLEERPTSLLIPHLCWSQHALTSLVRSMTSVAVTTATSLQYNRTEYHLQYVLIMADLACPGTDRFLIKASKEGYFKAPYRWLLLNYDDDKTVLKDVHMLVDSDVVVTKRISDQEYWFIEAYKISENSEVIYTLRSKWGRMEDHRSSNVLSSRRHDLRGHILTMSNVITDSNETRVHMNDRLNLHQDSITKMSYAVVKICFEMLNATERLMFTHTWGYKDKNGQWQGIVDQLLKKEADLGTLTIFTQERMMAVDYIAMVGSTAVRFVFREPPLSYISNIFALPFSGAVWLAIFICVLGCSIFLYIASKWEASMGMHPLQLDGSWADVLILMIGAVLQQGCTLEPRYAAGRCVTLILFLALTILYAAYSANIVVLLRAPSSSVRSLPDLLNSPLKLGASDFEYNRYFFKKLNDPIRKSIYEKKIAPKGKKANYYSMKEGVERIRKGLFAFHMELNPGYRLIQETYQEDEKCDLVEIDYINEIDPWVPGQKRSPFKDLFKINFLKIRESGVQANIHQRLTVPRPRCSGHVSTFSSVGITDMYPAMLMTLYGMLLAPAVLLMEIMYHRLMIARQQKRGTSDYDHIPFRH

>HarmIR75p.2

MLSMRIILQAFILVLILVERKAFARDHTLVHFIKSYVQNEEKPTILIMNNLCWEKKVVVSLANEMSKIGSRSSTSMGVDSRYYYHDLLYLLDLDCPGAEDIIALATARNLFRSPYRWLVITAWSKNANIAALWNSPLLADSDLVLAAGSGGVLKLVELHKPSPNGTMISTLRGFYNGSLYDVRPHRELFRRRRDVMGHTITMSNVIQDSNTTVYHLPREDGMEPQYDSISKICWMNVKLAFQMLNATPGYVFSYRWGYKVNGKWSGMIDDIHSGRAELGTNCVVSDIERLDVVAYTDRLAPFRVRFVFRQPPLPYVANIFSMPFSKNVWIAMSVCAVLSTATVYLAAKWEAKEGKGPTQLDSIGDAMLLTFSAIGQQGCVMEPRRLSGRMMVFVLFTALMALYAAYSANIVVLLQAPSDSIRSLPQLANAKITLAANDVDYNHFVFKLHKDPVREIVYKRIDPEKGKKHFYDLNEGVERIRQGLFAFHSIVEPVYMRIEQTFLETEKCDLMEVDFLNSYDTFVPVRKESPYLELLRVVFKQIRESGIQSALSKRLQVPKPHCTSKMSSFSSVGLMDMKPVLILMLYGVCLSVTIAAAEILVFKLSEHRKKYKVDSTEPPSP

>HarmIR75q.1

MKYFTLFLNILCLKWCVSILPNNDLQMIVDLANSYEKPTAIVANVCWNSKADEVKLSKMLTNLERPITVRYLRKNKTFANSYPNNHLLLLLDKNCVDAEFFLKQANANKMFSKSYRWLVLGNPIQKESIVPPEFRGLNISVDSEVIMAQKTANNDTLLHTIYKIRPKSEWNIEYYGVWSADYGLNKSDKTIQSNVMRRKDFKGEPLTASSVVEDKRTTLSDLITLRHILVDTVAKSTFRSINPLFDFLNASRRILFNDTWGYKVNGTWNGMIGEINTGKAELCGMVTFMSLERLKILEYFTNPTPVTVKIVFRKPPLSYQNNLFLLPFTTGVWICLGAFVLIMIVVLYINTKWDIKKYEQFNEQNMDQTCLPPTWSDITIFVLSAISQQGSSNELKGTLGRLVMFIVFLAFLFLYTSYSANIVALLQSTSNQIRTLSDLLNSKLELGVEDVPYNRYYFSPAQSASDPIKKAIYETKVAPRGKPNFLTLEEGVKAMQKRPFAFNMNTGTGYRIVSALFQEHEKCGLHEIEYYQNAKAWLCSGKNSPFGEMFKVGYIRIQEHGLTDRENRLAYAKKPVCSVMGSSFDSVNMVDFYPVCLMLLYGMILAFVLLVIEILAHRHQMKKHNQEPDVTQLQ

>HarmIR75q.2

MKTIFFIAALCSLSLCNGQDSQNMQVMLSDVIEASGRPSSVIAKLCWTPSKIIQLHTHLTNKYIQFSANDVIKTDNPEFYDEEQHIVFMADLDCPDIDTYFEKNSARNVFRAPFRWILFGNSSTNDDDIVPRAISNIDVLLDSEVLVLRSVDDVYEMHFIYKISPNNTWQTEFYGTWNAKHRFQKSPRFFEPTSLRRLDIDGYEISICYVLTNNESVNHLSDGLEDHIDTITKVNFPTTNHLLDFLNAKRKYIFANTWGYRVNGSWNGMTGYLVRGEVEVGGSPMFFTFERVSIVDYIASPTPTRSKFVFQQPKLSYENNLFLLPFNTTVWYSTIALVFIIYLVLLLVTKWEWKKTQDLLETREKDAGVLRANVVDIIVLIFGAACQQGSPSELKGSLGRVVMLVLFLALMFLYTSYSANIVALLQSSSSHIKTLEDLLHSRIKFGVHDTVFNRYYFSTATEPVRKAIYEKKVAPPGTTPRFMTMDEGVIQMRKGLFAFHMETGVGYKFVGKYFNEGEKCGLREIQYLQVIDPWLAVRKNTPFREMFKIGTKRIQEHGLQYRENRLMYEKRPKCTGGGSNFVSVSMVDCYPAVLILSYGAIVALFLLALEILAHKKENILRKLNCRKDEMN

>HarmIR76b

MAGIELIISSICNATFCDVPYGGADKGSEVLTPEVINFKSLMQDVNGKNLKVTTYNNTPLSWTEHHNGTVVGKGVAFTVMEILRKKFNFTYDVIEPKRNYELGGRVTDDSIIGLLNSSKVDMAAAFMPTLIAYRKQVSFSIDLDEGVWVMMLKRPKESAAGSGLLAPFNDLVWYLVLAAVLTFGPCITFFTRVRTKLIADGEGVLPLKPSFWFVYSAFLKQGTNLSPEANTTRVLFVTWWLFMILLSAFYTANLTAFLTLSKFTLAIENPRDLYQKNFRWVASAGSSVEHIVKTEGEDLYYLSAMINNGKARFLSVLSDKDFLDPVKRGAVLVKEQTVVDHLMYNDYTSKKDVEESDKCTYVVAPNAFMKKQRAFAYPVGSKLKSLFDPVLTQIFQAGILDFLKRSDLPSTKICPLDLQSKDRKLRNSDLIMTYLVMVAGSATAVAVFAAEIFIKRYLSVKVNKTKKTKDRNKKSKIGKKSTRYDDSRPPPYDSLFGKNPRFNVETTRTKIINGREYYVFETGNGDRKLIPARAPSSFLYRSDK

>HarmIR87a

MFAPLPTLLFFIHFSSTMSENPLLMTTGNSGQTTKTAECVLKLSAKYFVEKKALSGSIVIININSYTSTTQGLLLQTVHSGIKYSIMVKDSFYPHANASHFPEKAKNYMLILEEKSELERNILQLNKLPTWNPLAKAIVFYQLKHNETAEETSIEFINELRDYKLFKTIVFIYDEYNDVVISYTWRPYSDTNCGGRCDSVYVLDRCTNNTIYEFEKQHDMFPSDMKGCPLVAYAVIAEPYVMPPVGKITNSSFDDAYEFAKGGEINLVKIISQFTNMSLITRTSDILENWGVVYQNGTATGAFEVLRNESADLVIGNVEVTRILRKWFHPTVNYLQDEMTFCLPKAGQAPTWDNLVIIFQWTTWVATFFSLVIMGLVFHVFYYREHTNATKWPTNSLLMTFSMLLGWGASFEPKSPTFRILIFAWLCFSINMGISYESFLRSFLMHPRFEKQISTEADLIQSRIPLGGREIYRSYFETNNASSFYLYREYNSTTFSEGVRRAALERNFAVVSSRRQAVYQDQKLGKGAPLIYCFPESNNMYKYGVAILTRRWFPMLERFNNIIRSVSENGLIDKWMNELLIHSVSSEEASTIMPLSIQNLLGAFMFIGFMYGASIVIFIGEVVIGFIEKRKQTKKVMNKRLR

>HarmIR93a

MKVWILSFVCLFVSVSGEDFPSLITANASIAVVLDRQYLGEKYQSILDELKDYIKELARVDLKHGGVIVYYYSWTAISLKKGFLAVFSIASCEDTWDLFSRTEEEELLLFALTEVDCPRLPSHSAITATFTEPGEELPQLLLDLRTSNAFQWKSAIILHDDTLSRDMVSRVVKSLTSQIDEESASPVSVTVFKMKHEINEYLRRKEMHRVLSRLPVKYIGENFIAVVTSDVMTTMAETARDLLMSHTQAQWLYVISDTNAQNGNLSSFINDLYEGENVAYIYNMTDNNPDCKNGIMCFCQELMDAFISALDAATQDEFDVAAQVSDEEWEAIRPSKIQRRNMLLKHMQQHLAAKSRCGNCSTWRALAADTWGATYRGLSDASDLSNVNTNGSSGVIDKIDLLKVGFWRPIDAVRFDDVLFPHIHHGFRGKELPIITFHNPPWTILERNESGAIVKYSGLIFDIVNQLAINKNFTLKIILASVLKKELANDTLADTMHGMDAKLTIAAISKGQGALAAASFTVLADPMPGINYTMPVSIQPYAFMIARPRELSRALLFLLPFTTDTWLCLGLAVILMGPTLYIIHRMSPYYEAMEITRQGGLATIHNCLWYIYGALLQQGGMYLPRADSGRLVVGTWWLVVLVVVTTYSGNLVAFLTFPKQEVPVTTVAELLENRALYTWSITKGSYMEMELKNSDEPKYIALLKGAEMVTTSVGMGGTMTSGSALLQRVRFHRHVIIDWKLRLSYLMRADRLESDNCDFALSAEEFFDEKVAMIVPAGSPYLPVINKELDRMHKAGLITRWLEAYLPKKDRCWKASSMMQEVNNHTVNLSDMQGSFFVLFMGFFSASTVLVLEFLYHRRKRRSELTVIKPYVE

>HarmIR100b.1

MKLLAFLILTTLLAISSSKPGKKLVPSVAIDCPLIPITKHSQTILFDVDVNSDPSIRTCILKSVSEQSVLILTDYDRRPEVNFTNKRIMTNEFPNVIITTNHLSFKAVRCNKSPLLELFMKSNWSWLYIIVTGDSVFSCKNGTMTSKHFRLLEKFMNTIWHRFEVMRVGLAFPIACKQKMIIYHRKRPSTQKLYDRSIKLINATSYKDLLAAINYSGTGLCAHYPIKANIFERYPTSITQCKNLHYYDIHFNLNLTFGYCGLDGMVMHDLLTHFRFNLSSPKNEDCNIYGFAIPGNISGSLGCIVRNELDLSFNSRFMTLYSDEHIYYLHYITTDKLCALVRKTGVVPLWHGPYNVFRPPLWFIIMGVLVLISGIMWVYAIINRTITGQKVMAYWYYLLNAVMTTMIGCSPMKNRSMIIIRSACLSGSILFLAVYQGHTSRVYTTLKHFERISTLDDLYAFGAILYTTPGMRQFTRQLQRPGNKLEEDFFNRSRLILNERIGAGITLEIPRATTLDRKSDAEMKILEHFSDREGRPLIDIVDECFMNYFLSYITRSGFPFFEEIQIFTQRLLEAGLPTKYYKWTQQMLNIPTSLPETRSEPRPFSKIKLKDQRVAFFVLFVGSALSIIVFAVEIFKGPPVEF

>HarmIR100b

MHRIKYFVFVVCIASSNCELRTKLMPAVLPNCPLLPVEKYSQTVVIDINTNTNPELKSCLVKTCGHSVVIINVCGKRYTINMDIDRKSIKTTDYPNIILITKHFTKKTADSGLLKYVVKSLNWSLLYFIVTGQQQYQCLNGTMSTSDMLLLDNIMNSIWHKFKIMRVIVAFPYTCEDKMLVYHGKRPSTDGDCLYDRPVKLINATNKEELLKAIRKSGEKLSENYPIKASIFERYPTSIKDCSNLHYYGHFNLSRSHGYCGLDGIVMNDLITHFNFNLSFPENETCNTYGFAVPGNLSGSLGCIARNELDISFNSRFMTLYSDEHIYYLHYVITDKLCAFVKRTGVIPIWHGAFNVYSPPSWMFIIGVIMIISVIIWGSAIVNKKLTGVKNKSCWYYLYHTLTMTMTGSSPMKQRTLLLMRGSCLAGSVLFLAVYQVSWSGHISRVYTTLRRIEQISTLEELYRSGATLYTSPSFRELSKQLLNKKNKLQVEFFNRSLLTPHETLDIVLQQPFATSLERKSDAEMEILTKYSNEQGPLIDMVAECLHNYYLSYIARSGFPFFEDLQVFAARLKEAGLPEAYYRWTQKMLNIPTSMPEDHSIPRCFRPIRLRDQRVPFGVLFVGTILSLIVFVAEIRKGKSRNVK

>HarmIR85a

MLVIFVILLMCHPSLCFDESSVLFQEVKEYHRDAWLKAEYAYEVVNIMYNSFRQWYFTVTFCEFTYFENRILKYTEQYGYGYNVMLLSGCPYSNNSFVKPRHNRHGETAYLVTSNDLSLDVSETVIAALKRTGIFKPRSAVIFVIKNVLELDNYFYHALSNHFQMLWSSSVTNSVLILKTDRLRMYSYNPFFQEIKDITNVRDVSKLLSKQYNNLYGYGLRLSVFRKVYVSDRTGPVRCDSYLAQTVMKFLNASCYPLPPRDGSTVGDLLENGTATGVTSDLIDGYTDLELNSRILKNTYYGYIDTTYPLDQDELCFLVKKSDTQSTFKTTINLISMEMLLLFFFTFTVFIIITILVRKAENNLLNLNDERRAEDTLIDLIKCFIRQTMDFDFMGPVFRSLVLLIIIYSLIIDCAIDGIITSAITYPRYKPDIETMAQLGASNLTFGIHNRDLKIFNSSLSTDYYELIKNRIVPFSDKKIKEVLEKREYQYATLLRKSDSQYVSRKVSNMRKGKPLFYTVPDCPLPCFIVYGLRYGSPYLNRLNYIIHHLFQAGILQYWSKTEELNADRSRLGAIENKDRKPLNIKNLQEMFYMLAIGELISTLVFIFEILYHKYHKKQ

>HarmIR100f

MTVVLFLTLLLLANGTSKSLMKPENPDDQLLTTCVIDILNKYFIEQKQLTIVLNSTADMKLQDIYSKTNTTIFIRRPFCNFCHYKRVHASYVFFANNSQDFSENFQYLVQEPFWNPYGKFLIVISSLQGDLRVIFDVLLMFHVNNVVLLNGTGPGHLYTYNPFANYACGRYYDEIIGFGTCLNNRANLFPDKLVSGLRNCTFRVSFPHKPPFTVNPSIVKHLIRQQPLIGFEEKLLKVLAEQEHFNITYNDRYGKPFYSVILPNMTAVGPLSLIQRNKTDIIIGGMMLSPARYSALSHVSGHFDYLDELIFAVRKADLVTNSKKIFLEFHPTVWLLLILVFIIYFILLIIVLRPKDKSLIMLKMLSSLVLHGCGMSCRYTVKWLVLIWLVFAVIINIFYQTSLYSLTNNPIREHQINEEHELNNLKACIEPALVAYLRAEHIPNNITLSEEDINDCDNIARNVHKVGQNRKLYTVLRKMMFEFSKDHFTDPWGHSPIHTFTKPLSKTISAILLYKGFPTTHQLKVTLLRLIESGLVNKFLGEHMHLKKIKFVFPREPFRAHVIIPWNLFAVGCILALITFFLETLHHRKYKIYFRGDY

>HarmIR100e

MLTVHFLLTLSLASCRSDNIHIIEPQTKMPLIMCITHVIEKYFSEQKVLTYVDMDSDDNVLLKAIHSLKATAVVFRQPFIRSPFRHRGYLIAAKTSNIFADHFKKLRQEPTWNPAARFLIVIKILKEDELKKIFDVLLESHVFNSLLINATDEAQIYTYNPFDKYACGHYYSEVIYYGECSQTKTDLYPNKLVTGFKNCSFRASVPHRPPYSVDRAQLNNITQTDILGTEQLVLKLLGEKEQFQVNYTYDYDKFIYSTISPNMTVSGPMKTLTNNESDIIFGGIWLVASRSDAFTYLYGHLDFEDDLLIIVKRASLVAIWKTTFLEFQPTVWGLLVMTFLLYSVMMIRILGADDKFGVMMTLFKNLLANSVHIPNRSEVKRIFLTWIWFSFLINSIYQSSLFSLTSHPAKEHQVYKIREIFVYKMKPCINPSMLIYIETEMNVSTPIDNNCLDTPKNLYRVCQSDKIFTLAQKFVYQYYKKLYCDRFGQPRVYYFNAPYAQLMFGIFFYKGFPITDRMQTHLLRFKENGLVSKCLKDHYYSRKIKHHFHEKEFEARFILPWVMHLIGCVLATIVFIFELLSKRFKWFQR

>HarmIR100c

MTKRIVVLFYLTFHGSFIQLTDAENIPELYNFAVNKKYNTVGCINSILTKCFPCGSLTTFVNPNANMDQLIKTTNAENICHSIIVRSFDDCDWYIWTNVYVITAPDLLAFWFGMLDLSRDLFWNPRAKFIIQVDYLGANGEGIEDVFKIVIKHRMYNVVLLQNLKDDAVIYTYHPFENNSCGKRFDKIITLGKCENEDDIVDYFPNKIPSEMKNCTFNVVATDDVPNFISKSSNYTVYGKYVSGLEQFVLDTIAEREGSFVEYEVITGDATFGVVLPNRTTTGLLNYIDRNKADIAAGGFILMQNRVELFDYIWGFNYAAFYLYTPAAGNQVWRRVYREFGLRTWTLTGAALVLMIVVGLVIKRLINDKSFSVLYIWGYFFGNSNGGFSTHKKFKLLMLMWSFFAFCISSFYNTALVGLVSVHVQEQPHSLNVGNLKTLSYEPCISDNSRLFFQYAYNQTLPVGKDIHNCTNTDSSLELVASTKKYYAVEMEYSYKLKEYQYMDKQGKPKLESWLFSNTNVIVVYLVRGFPFIEKFQDYAHRLYESGLTLNHFKLISLRSYSVLQRHPKPFSVTKLLDLKMHFGILVAGWVLSFICFLLEIWYNNINISKKIQRVYVN

>HarmIR100d

MFFLVMFYSIVSAGAFLTTDHENIESLTNCVVKIIKKVDYNRYTKVVDVTLMNIKNDIKLSALHNLTGVRFVSRRFFWNTNDLSNKYYLVMSEDFQELEEGLKEVTSDIFWNPLAGFIIVLKSHKHHSSHDITDLLHTYNIFFASLILHKDDKYFIYKYNFTASNRCYKAGHLTLWASCSDFCSEKQLPILIEGNIRNCQYKFISRNLWPFTNFDTAFKGTEQWFVALFEKQYGVKIDLKKFGKIDKCETITYDTKYIMLKKVENNEVEGAVGGYSITEEYSGNVSHSYPISIDYMYYILPHKKYVGPLVAVLHGSTGTFVLIGLLFVIFCIAAKFLSIFAAQKDFSQDVLMVFGYLLNKCSANRISAGWPQAIVFSSLLFTSFIFPYAIQANLYSVTTEPVRGQEPKTSQDLKNYKAVLYTDFQYRHNKEFSGYLDCGNRINCLMLVKNCPDKSCYTVMTSAHYFANLWQLTDDECKMTTYILREPYITSLRTIYLRRGSVLIRPIKEFLLRIINSGVLSKYSRDLYIREWLKRKCHHRSEHVPLSMSSTYYVFMMLIAGYCLSVITFICEFMARSQIIIRRRT

>HarmIR100h

MLVAYILFFLLYEANSYKIPTPCPEYNKKLVKCVTDIVREHYSEKKVITYVGDKFEDEELLKAINNAGTVSIVSLKSTKRMIIPHEAYLLSGKNATFIAKHFPKVRRETSWNPLARFLVLVKNLTESDLKIVFDMFLQLHARHVIIVNATEDAHLYGYNPFDNYGCGKRYDYIFSYGKCAKAFYKELYVNKIITKLRNCTFNVVITQWPPYTILPTNDSNDLSPLRHGAEPYLFQLIGRMLNFKINITYDYTAVEEFPTVSTDMEAVGSLKKLQDNQADAAIGGMLLTPSRALAFSFVYGHLAYTDEIRFIVKRASDVPPWKNVYLEFHWTVWVILLLTLMFYSILVILLLQTNDKSYVVLVLLSNLLLHGHSFRSRSTVKTVLILWMWFAYLVNTFYQSTLFSLTTNPAQEYQISNEEDLARFRLKPCFSKVMENYYRESVQSNDGYQRIKGCDGLVESVHTVANSEDLYTILLNGQFRYNMQEFRDKYGTSRVVALPKPYSKVMYAIFLYKGFPMINYFLHKSLRLRELGLVDKVLKKLTYRRLIKYRFHEKEFQTRFAIPWIIYVFGCSIAIITFIIEINMPRH

>HarmIR100i

MLVAWVFTILLNFGQCQNILPPEAEDNQELRQCVTDILDKYFNESIELTYVNMETDNEDLLQTVYKSQKFSLVTRNSMYQSVLPNYGYLIITRNVNTFIEYFKYLQTDTTWNPYARFLIIIETLEDEDIKSVFDILLRKHVNNVVVMNGTTDAHLFTYNPFDNYACGKYYTDIIDYGPCKENPNDLYPNKLVTGLKNCTFRAAIVHRPPFTVNPLKAPKTILGTEEYILKALAELEHFEVTFNYSVNPAVFSSAFPNMTAFGPMEMLQNNETDVIFGGNMMVLTRGQAFSFLNGYHDYNDELRFVVKRASLVPLWKTVYIEFDTTVWMLILLALVVYSVMVIYLLQTKDKGFVFMELLDNLLTHSRDIRCSMTIKCILMIWVMFAYLINTCYQSSLFSLTTNPSKEHQVASEEDIIQYKYKPCFSVALKTFLSLEITEGMLSTRSSPADIDQNACSTTIQAITTVSRTKGVFSLIPNYIYLYNKPSFNDKFGNPLIYYFDKPFAKFLYCFYFYKGFPISNRMRMNAIRMRENGLADKSMKDHFFKRALKQRFSQQEFETRFVLPWGLYIIGCTISIITFLVEYMSQSQLKIQEQHVL

>HarmIR100g

MSAAILFSILCLNYCTSANGFYLGEELRNENLIVCVTGIIEKYFSEYKGLTHVQFETNSEHVRLMRAIHSSAITALVTQDPNQQLRVSHQGYLITASTAKEFTDNFSTFVDDLSWNPFARFLIVVRRVHEEELYQIFDVLLRWHVNNIILVNGTNDAQLYSYNPFDNYGCGKIYKDVIRHGFCLETTSNLYPNKVVTGLRNCTLRAAIPHRPPFTLRPYMIEQNDRMKMGSEQYLFCLLAETEQFKVEFYYEGDYYPLSKLYDNISIPGPMENLRNNKYDVMFGSVMLVASREARFTYLHGHLDYHDEIRFIVKKANDTANWKNAYFEFHARVWWTVLAVFIVHSTLLNIHLRSKDKTDTMLKMFDLLLSHGCKMPHRMSARCLFLIWVWFAFLINNLYQSALVSLVRTPVKEYQVKDEEDIVRMQLKPCISPPLLQYMYTESNITNSFNSNCTNPFSSINFVAKSKHYYSVVQKTVYFYNKKQFCDEEGENTVYFFKKPYAKLIFGIFFHKGFPISDRMRTNAIRLRENGFVKKKLKDHIFAREIKIKYHDKGFKCRITVPWFIYVGGGSLAVIVLIVEIIWSRYKE

>HarmIR100j

MRAVYLLVFLIYSGNCVKIPIAGPKFSEKLIQCVIGIIREYFTGSKVITYVGNSYQNEELLKALNNANIMSVITRRSTIKRTSKHQAYLITASNATFFAQRFIKTTKEPSWNPNARFLIIVTDLDGDLKLMFNTFLKLHANNVIVVNATDDAHLYSYNPFDNYNCGKRYDEIIEYGKCSQAHAYDLYPKKLVTGLKNCTFNILITQWPPYTIINTNDSNNDDPLKSGAEPYLFQLIGEKLGFDINILTNTNDSEEFPTVSSEMTAVGSLKRVQDNEADVVLGGMLLTPSRALAFSYLYGHFVYTDEIRFVVKRASDVANWKYIYLEFESTVWLLLLLALVIYSMLAMILLRTNDKSYVALILLGNLVLHGRSLRTRWSVKYVLIIWVVFAYLVNTFYQSSLVSLVTHPVRDYQISTEEDITKYQLKPCFSSIMGKYYVESVQSGTGFDVTHGCYGLMESVTTVSQSNNMFTILLYGVFQYHEQKFFDEYGNPLIISLPKPYSKVIYSMYMYKGFPMMDQLRHRALQLRENGLVEKVMRDMIFMKRIKHSYHKREYVPRFAIPWLLYFIGCLASIIAFVIEIISKRNIHTEA

>HarmIR100a

MNYHSIFILCIFARTAHGNSTNGLSLLKTGPDLEDQRITMIIDIISLNLKQNGWDTIMCVGKLPDSFYVKIQKIPYSFVVMNLDDPDDYFIEDTVPYHANFIIISCQDYEELEELRQKLVASPYFHPLANILTYYHRREDKATMAKLFFSAWYYKAINSILVQFSDEEETLLVSDFTPYVNEDYKIQPENKFGCWTARNLGMPVVGFDTGYVCVEKCHNVSIHTRLRANNLGTCIGFNTNSVSYNDFTHLRNLKLFEDRTKDLHGFVFRAYAVQVKPFFLIKNHGNGTYTLYARDGMIWNTMAELFNFGIDLSPSVDVMMKPFNFEISIDQIFAFARRKGDLCLFPIYQFDVIVVGLDFTYPFKDSGICILSARAGFETSLFRIKTLRANISTIILFLACFACTWATFTVYKAAEKRHLSFDQIGKDFMNTCRQILMINLYKPPTHHFFRIFLAIALWCFFVINFTSQATIISFFTAVKRGKEVDTFDDVVAKGYPLEAMASPDLILPDTEEKFRIINSRLVNEVDIYGCVDRLKTDPRRFCLMDCSVGRYIKRNRLNHRGEQYLHIAEQDRIHSHYLAMVFSEHSPMTERFSRYMMILCEAGLIRKWEQYRYTDIKDEVTTKPLAFDDLSGIFQVFCFMVGNTLMIFFLELVASRIKTCCQNIKIPNRLPKRNTE

>HarmIR143

MWKYLVYSTLIIVNSVHGKTDTMKLLPKNNEKNASEFNSITDCIISISDKELFFMPTVAILKQAENNIKENLFINELIDKLTLFNIPQIIVEEIDNEENHELYINSLTIAFIETCDDINKIDFNKVDRAMRFLITISDSTKDKCIENLQEIGESISHDAVTFIFRSNEADINEMFTIFPKIDGNTCKEVVDEPKHINTCINGTLDNDDIFPIKNPGNLNKCPFKVGMSTLFPFSTMKNKEKLKLYDRVDDIKGSDFEIMKIINEYFNATLEIYYIFKTEENPYSDVEFIPFVINGSLDACAGGIYRIYGDIVEYSGIYVSQGIFWVYYVEREDRSWQNLMHKLNDIYIFMIFYISYSIIWCLIRLFDGEAVSLMKTLLYCWGALVGASSLQDPRSRKQKFLTLMYLIMCIYLSAYVSMQFYAFLTITAPPHTFKTNSDVMESGRTAYLKDITKYFISDERYTRFANKSADCVSFLDCSEKTLLYNGLTVILQGFFYNFQAATAVNDEARILRATENILTVYNEMIIRKDSPLVEKFQKVMQRLFEAGITRRLFTEAIGISVVAKAKSANTNMISSSYSCQAGCSITLKQFAGVFYAWIFGCIISSVVFILEIFLKREKRLLTKIE

>SfruIR8a

MSIYYLFLLIFFINVGCVISELSLRFVFIIEAQEQDLTRDIGRALKLSETVRPDVKLDDAIVLLDRENDEESNRILCAAISKGVSMIIDLSWSPWQVAEDLASEAGIPLVRTLLGSQQLVKALDSYLESRNATDAALILESESDVDRTLYELLGASNIRVWVHAGLTRDSAKALKTMRPEPSFYVIVGENGFVMDTYRRAVKEKLVRRNYRWNLVLTDYSSVEVGQLVLPTVILQSDPGECCKLLRKDECNCPSDFQRKQHIINSLILYLSEVYSKLEGDLTLTTSSIQCEDPQPSMNTTRDRLFKQFAEDSEISNETVFFWDGDRSGLFLRSRFMLSTYKPEDGQQAIATWSAGEDYKLLPGIELEPLRMFFRIGTAPAVPWTLMKLDPTTGEQMYNEDGQPLYEGYCIDLIARLSETMSFDYEIVSPKTGDFGKKLPNGTWDGVVGDLMRGETDIAISALTMTAEREEVIDFVAPYFEQTGILIVIRKPIRKTSLFKFMTVLRTEVWLSIVAALVLTGFMIWLLDKYSPYSARNNPDAYPYPCREFTLKESFWFALTSFTPQGGGEAPKALSGRTLVAAYWLFVVLMLATFTANLAAFLTVERMQTPVSSLEQLARQSRINYTVVEGSSVHQYFVNMKFAEDTLYRVWKEITLNATSDQAQYRVWDYPIREQYGHILLAINASQPVPDAKTGFQQVNEHTDADFAFIHDSAEIKYEVTRNCNLTEVGEVFAEQPYAIAVQQGSRLQEDLSRALLELQKERFLEQLASKYWNESARQACPDADESEGITLESLGGVFIATLFGLGLAMLTLAWEVFYYKRKEKNKVQTFNAKPEKQAFDAKSSLESKVAQSVAKIRKRGKIGKRGNVTKNVTFGDSFKPVSEKGVSYISVFPKDYRP

>SfruIR21a

MAVPWFCVVFLAYHGVFGAQVIIEYYPSQSVIDMNNKIVRKREVNSSDDVNFNINGSDSDSYWRHFNNDTDDGDIHKRALDPVFHGHPKTREELWNERFLNETTSFDQTPSLVNLLHNITLTYLKDCTPVILYDSQVMSKESYLVQNLMKGFPTTFIHGYINDDGELVEPELIHPTIECLHFILFLSDIKISAKILGKQPENKIIIIARSSQWAVQEFLASVTSRNFVNLLIVGQSFKEGDDATLESPYILYTHKLYTDGLGASKPVVLNSWTHGKFSREVNLFPLKMTEGYAGHRFVVAAANQPPFVFRRIKSDLDGGNPRVVWDGIELRLIKLLAERNNFSIEIIEPREPNLGPGDAVAKEITTGRADIAIAGMYLTNDRIRDMDMSLPHSHDCAVFITLMSTALPRYRAILGPFHWHVWVALTFTYLFGMFPLAFSDKHTLRHLLHNSGEIENMFWYVFGTFTNCFTFLGKNSWSKTNKITTRLLIGWYWIFTIIITSCYTGSIIAFVTLPVFPETVDTIKQLLAGFYRVGTLDRGGWEKWFLNSSDPQTNKLLKKLELVPNVEAGIRNTTKAFFWPYAFIGSKAELEYIVQANFTATKSKRAVLHISNKCFVPFGITIGFPNNSVYSAKMNLDISKMIQSGLVDKITNEVRFEMQRSPTGTLLAAGSGTIKIPSAEEKGLTLEDTQGMFLLLAAGFTIAATALVSEWMGGFTRRCRFQKKSETPTSVDSRENLIITPKTDIDSEIRIIEDTESRLHFEERPSSSVSVDTLEGQVIHVTESSIDVHNTFNVDRFDSRRSSSLDLDREVREIFEKDQKRRRIFSRDMESLDENGSTVSRAAFGDSVKNDI

>SfruIR25a

MSSLTVFLLFYFIRNTFGQTTQNINVLLINEESNALAEKAFEVAKEYVRRNPSLGLAVDPVIVVGNRTDAKVFLENVCRKYNDMLSAKKTPHVVLDFTMTGVGSETIKSFTEALSLPTISGSFGQVGDLRQWRSLNANQTRFLLQVMPPADILPEAIRAIVTKQDITNAAIIFDEFFVMDHKYKSLLQNIPTRHVITPVKSFEANEIKTQLESLRNLDIVNFFIVGSLRTIKNVLDAADKNQYFGRKTAWFALSLEKGDISCGCKNATIVHIRPTPDANSRDRLGKIKTTYSMNGEPEITSAFYFDLSLRTFLTIKSLLDSGKWPNDMKYITCDDYDGKNTPNRTLDLKTAFQEIKETPTYAPFYIPPDDPMNGRSYMEFSTDLLAITVKDGASISSHSLGSWKAGLSSNLTLTDPNNMSNYSAQLVYRIVTVEQKPFIIRDEQAPKGFKGYCIDLIEEIRAIVKFDYEITLAPDGNFGTMDENGNWNGIIKELVDKKADIGLSSLSVMAERENVVDFTVPYYDLVGITIMMKLPRTPTSLFKFLTVLENDVWLSILAAYFFTSFLMWVFDKWSPYSYQNNREKYKEDEEKREFTLKECLWFCMTSLTPQGGGEAPKNLSGRLLAATWWLFGFIIIASYTANLAAFLTVSRLDTPIESLDDLSKQYKIQYAPLNGSAAMTYFQRMANIEEKFYEIWKEMSLNDSLKEVERAKLAVWDYPVSDKYSKMWQAMEEAVLPNTIEEAIQRVRDSKSSSEGFAWLGDATDVKYHVMTSCDLQSVGDEFSRKPYAIAVQQGSPLKDQFNNAILQLLNKRKLEKLKEIWWNNNPESMKCEKQDDQSDGISIQNIGGVFIVIFMGIGLACVTLGVEYWWYKWRKRPAVGDVTQVEPAKFTRNNVDKQGEGFNFRGRNLGLNFKPKF

>SfruIR60a

MSLYKILVIFLLVNKCEGTVNPYGPTVVRDYANCISTIIDNEFNEPGLLIFANTNNVSTSVSNIRTVLLKRLHETMNFSVEIMSPGKEVEICDKDNYNLGVLHVDTFVAIPSANYFIIIIDSYTDFSYLASKLIRSRSWNPFAKFIILLFNYVRNDKINIEYVEKVLSCLFKYNAINIVIAVPQASNVRNAIIYSWRPYDPPKYCGYFNETAKDRLVAVNTCERGRLKYNNSVFEDKVPYDMNGCVMEILALQRQPFISDDEQYTSIEKIMIDRMLKRFKMKAHYHFLEGFRGERENVGEWNGALKKLSSKTGQLLLGGIFPDFDVHEDFETTTTYLADVYTWVVPRAPKSAPWVALVFVFKRLVWYSVIVCFVLCGIAWTVIGRLSGESPYNKSLFHCFLNTWITTMGFVTYLHPKKDSLRVYFVFLNMYCILFSAAYQTQLFDVLTRTSYDHQMNTVQELVDSGIKFGGYEELHDLFYNSTDPFDNLIGEKWVDIENITDALINVAVYRNFSLLCSKLELKHISAVTPALSDNAGNYNYHTFSDNVFNVPIETIALRGFPFMLKFSTTITIFKQSGLNEGLRHQFTEFTERRRARQLRDLLKEKSDVSPLTAKHLQGGFFALFLGYVSGIFTLIAEVLVNTGSFKKKFAQCKRKLNFM

>SfruIR64a

MNLLCNLLLALSVTDVRLVIDIFKLKNLKNGVIFHCYDNYVVSNVHKVLNEHGILVASANIDYNVTYNVATSYPKVGLVIDAACERWTSVLDSDTISFQGYSFIIITEDVTGTTEMLSQYPIEVDSDVIVAHKINQTFNLYEVFNTGTKYRGTYNVRKVGHWNTSLFINSPNRWNLQGIFVKTAVIILTTPRIVNQTIEQYMEKPIKSQIDVDTVHRMKYFIMLKFMRDMYNISYDIHRVSTWGYQRNGSFDGMVNALYQGMAEIGGAPIFYRIDRGERVQYISEVWMSRHSFLFRHPKYPGGFYTIYTRPLSDVVWYCVVAMLAVTAVTLWVMLVVQNHKGDNEDSSLSLAGLVIWGAICQQGISINRESTSTKLVIFTTFVYAVTLYQYYNATIVSSLLLEPPRNIRTLKDILDSDLKAGSHDIVYDRDYFKRTTDPVAIELYHKKVATSAQYNFFTPEEGIALVKKGGFAFHIDTTFAFPLIKATFTEREICETTLVQMYPLQRMGVVVRKHSPYKEHIAYAIRKMYEVGLPPRIQSEIDEPMPECAHTPDSSIFCVGIREFSTPLLALTFGMATSIVVLLCEIIIDRVVQLGRVRDFRH

>SfruIR75a

MILLSVLCCLFLTASGFNLDVINLVTDYVLYKDLRYVCYFTCETPYYNNILVHKLTGENVRVSVRRIDGGGNVDVARVGHQTTAPVGVLVDGHCSQTQALLHQASVSKLFDAVHPWLVLTDIEDDNCTEYVMQTFQWLNLSVNADVAVVANGGDSFTIIDVYNFGRIQGNGLETALLGTWQPDRGLHIVLKGYKYYNRWDFHNLTLRAISVIVDQPKVFYPEMLSEMAYTAGVAAMTKITSQMLNTIKERHNFRFNYSIAGRWIGSPERNSTMAVTNTLFWEEQDLSSTCARIFPKWLNWVDIYHPPTTNLQTKFYYLIPEKGVGQYENRFLTPLSHGVWCCAFFAGIACTLVLAAAARMEDRPKPGLYAFFSVFAAVCQQGYEDGVQLLEQTLSSQGRRLTLLVIGLTSMLLYNYYTSSVVSWLLNAAAPSIANLDGLINSDFELVFEDIGYTRGWLDNPGFFYYSGFKNVKEDELRDKKVTKAKRTVSVLQTVNKGVELLRSGKYAFHTEPYTASQVISKTYEDKELCNLGALQMMLPAHVYIMAQKRSPYKEFFDWSLLRLLERGHVKAIRARFAGTMPACSGAQPRPLALGQAAPAFLMLASFAVLSCLILILEIFCKRVQLKNRGQ

>SfruIR75d

MELISFVLSYFITKDLSMMTAFICWPPEQALELQRGARGAGVRLAVVSEPQRAAPMTPAGYFREAMLLDLNCPDTHLVLEKASGSRVLNRRHSWLLLHNSSAEPALVEETLNAYEILPDADVVWSSPDTMVDVYKTKPNQPLLQVELGLSRNSSHQELLSLWGALPTAVTRRRDLRNVSLKGISVVTEPDNFKGWADLRNRQIDTFPKFTYPLMMLLAQDLHFRFDLRQVDFYGVSHNGSFDGLVGHLQRREAEVGLASLFMRHDRMQVADFFSETCVLACAFIFRQPSRSAVSNVFLAPFSAGVWGASACVAASAALLLVALRRLRQRTRASTDLRLFTLLEAITFALGSLCQQGFHRTPPVTSVRVVMFSTLLTSLFVFTAYSAKIVAILQTPSNALQTIDDLVRSPMTIGVQDTTYKTVYFLESPEKSTQQLYRHKILPQGELAYHSVVDGIARVRTGFFAFQVEKSSGYDIIKQTFTEREKCSLSEIEAFKPPLVAVPMKKHSGYRELFASRLRWQREVGLMDRARRVWLVSRPRCEAAGTGFVSIGLIDVLPALQVLALGAIMSVLLLVGERSVRLLQRHTGHTTTPY

>SfruIR75p

MSAARLCIPQRLTAPSVGDSRDPRQSFMSVRRTGLQGSGIFMVVSIEDPGLLELQWFWEVVAGFSHKKGDEYLLIEELGIECRKTSRNMDAHILVPTLAVFIFLVKYVDSNDFDLDVISSFVTLDERPVSLLIPFVCWKQHDLKSLARRMCEVGVSTATSLQYNRTEYDLQYVLIIADLSCEGTDRFLVKASKEGYFKAPYRWLLLNYEDDDTALLHADILVDSDVVLVNKIHDQEYWFVEAYKISENSEIIHTKRLIWRRNNTKLDKNITFMDQNNVTDTYNTTSPVIYQYNDIKNNRLGVPTNTDVLKVTVTKYGRVEDYRSSNVLSSRRYDLRGHTLTMVNVITDSNETRLHMNDRLYLHQDCITKMSYAVVSICFHMLNATERLLFTHTWGYKDKNGQWQGIVDHLLKKDADLGTLTIFTQERMDVVDYIAMVGTTAVRFVFREPPLSYISNIFTLPFSGTVWLAIFICVLGCSIFLYIASKWEASMGMHPLQLDGSWADVLILIIGAVLQQGCTLEPRYGAGRCVTLILFIALTVLYAAYSANIVVLLRAPSSSVRSLPDLLNSPLKLGASDFEYNRYFFKKLNDPIRKAIYEKKIAPKGKQANFYSMKDGVERIRKGLFAFHMELNPGYRLIQETYQEDEKCDLVEIDYINEIDPWVPGQKRSPFKDLFKINFLKIRESGVQANVHQRLTVARPRCSGHVSTFSSVGITDMYPAMLMTLYGMLLAPAVLLLEITYKRLMTIRNQKRRLVDPDHIPFRH

>SfruIR76b

MAGIELIISSICNATFCEVPYNETYQAPDALAEKDINFMSLMKEVNGKHIKVTTYNNTPLSSTEFENGTVVGKGVAFTIMNILRKKFNFTYEVILPTKNFELGDKISDDSIIGLLNTSKVDMAVAFIPTLLPYREKVSFSIDLDEGVWVMMLKRPKESAAGSGLLAPFNDLVWYLVLAAVLTFGPCITFFTRVRSKLITDDEGVLPLKPSFWFVYSAFLKQGTNLSPEANTTRVLFVTWWLFMILLSAFYTANLTAFLTLSKFTLAIESPRDLYQKNNRWVASAGSSVEHVVKTEGEDLYFLNTMINSGKARFLSVLGDKDFLEHVKKGEVLVKEQTVVDHLMYNDYISKKDVEESEKCTYVVAPSAFMKKQRAFAYPVGSKLKGLFDPVLTQIFQAGILDFLKRSDLPSTKICPLDLQSKDRKLRNSDLIMTYMVMVAGSATAVAVFGAEIFIKRYVSGKLNKNKKSKRKKSKTGKPSRSHDDSRPPPYDSLFGKNPKFNVENTRTKMINGREYYVFETSSGDKKLIPARAPSSFLYRSDK

>SfruIR93a

MVSRVVQSLTSQIDDESASPVSVTVFKMKHEINEYLRRKEMYRVLSKLPVQYIGENFIAVVTSDVMTTMAETARNLLMSHTQAQWLYVISDTNAQRGNLSSLINDLYEGENVAYIYNMTDNSPDCKNGIMCYCKELMDAFISALDAAIQDEFDVAAQVSDEEWEAIRPNKMQRRDKLLKHMQNYIAAKSRCGNCSTWRALAADTWGATYRGFTETTDIVNDSNMNATNRVIDKIDLLNVGIWRPIDAVRFEDVLFPHIHHGFRGKELPIITYHNPPWTILQRNESGAIVKYGGLIFDIVNQLAINKNFTIKVILASVLKKELSNDTTTDMMHSMEAKLTISAIAKGQGALAAASFTVLADPVPGINYTIPVSIQSYAFLIARPRELSRALLFLLPFTTDTWLCLGLAVILMGPTLYIIHRMSPYYEAMEITRQGGLATIHNCLWYIYGALLQQGGMYLPRADSGRLVVGTWWLVVLVVVTTYSGNLVAFLTFPKQEVPVTTVSDLLANRALYTWSINKGSYLEMELKNSDEPKYIALLKGAELVSPTVGKSGTMPSGSSLLQRVRYHRHVIIDWKLRLSYMMRADRLEADNCDFALSAEEFLDEKVAMIVPAGSPYLPVINKELDRMHKAGLITRWLEAYLPKKDRCWKSSSMMQEVNNHTVNLNDMQGSFFVLFMGFFSASAVLLLEFLYNRRKRRSEQIVIKPYVE
