# Supplementary material for: A mouthpart transcriptome for Spodoptera frugiperda adults: identification of candidate chemoreceptors and investigation of expression patterns
Source: Front Physiol. 2023 Apr 25;14:1193085. doi: 10.3389/fphys.2023.1193085 (PMC10166800; doi:10.3389/fphys.2023.1193085)
Supplement: Supplementary file 1 [file DataSheet1.ZIP › Supplementary data/Table S2 (primer).docx]

**Table S2**. Primers for RT-qPCR of candidate *OR*s, *GRs*, and *IRs* in the mouthpart of *S*. *frugiperda.*

| **ID** | **Forward primer (5'to 3')** | **Reverse primer (5' to 3')** |
| --- | --- | --- |
| ***ORs*** |  |  |
| *SfruORco* | ATGTTGTCAGATTGGTAGCA | TCTTCAATGAGCCTGTTCC |
| *SfruOR1* | TCCTCCTGTCTTGTTTAGC | GTCCTCATCCCGTTGTAT |
| *SfruOR2* | GAGGATTGATTGTAGGGATA | TACCGAACAGACGAACAT |
| *SfruOR3* | TCTAGCAGCCTACGAGTC | ACAGCGAAGATGGAATAT |
| *SfruOR6* | CACAATAGACGGAGGCTTAA | ATCAACATCGGACCAAAA |
| *SfruOR12b* | AGTAGTTGTAATAGTGGGTCT | GTCTTGATGCCATTTGAT |
| *SfruOR17* | TTCTGGGCAGGCATTTCTT | CAGCGTCTCGAAGTTCATCC |
| *SfruOR18* | ATGCGTAGTTCTTGTTCC | CTGATTGCCTCATTCATTTT |
| *SfruOR23* | CACATGAATGAGCTGGTA | GTTAGGTAGAAAGGACGA |
| *SfruOR25* | ATTACTTATGGGTTTGGG | ACAGGACGTGTATGAGATG |
| *SfruOR27* | CAGTGACCGCAAATCCAA | ATGTTCCGCATTACAGCA |
| *SfruOR30* | CTTCGTCTGCTGCTGGTG | ATGTAGTGTCCCGCTTTG |
| *SfruOR32* | GTAATACTGCCTATCACCT | CTTCACCCTTTATTTCCAT |
| *SfruOR34* | CTGTTCGTCCTGTTTCCT | CATATCCTCTTCGGCTTG |
| *SfruOR35* | GCCCTTTGACCCTTACCA | AGAACAGTCTCCGCACCC |
| *SfruOR38* | AACGGAGTGCTGTATGTG | GTAGCCAGTGAGGACGAT |
| *SfruOR45* | TCAGTTCCATCGCCATCT | CCTCAAACGCCTTATTCC |
| *SfruOR46* | GTTCCTGTACTGCTGGTG | TATGTAGTGTCCCGCTTT |
| *SfruOR49a* | TGATAATGTCGGGCAACG | TGGCGAATGGAACTGGAA |
| *SfruOR49b* | GAGTACATTAAGAGGAATCGGAAGG | CATAGGCACTGACAATATGACCC |
| *SfruOR50* | TACGCTGCTCAAGGTTTCGG | TGAGGCAGGTGGTCAGGGAG |
| *SfruOR53* | GTTCTTGGTTTCCGTTTG | CTCCCTTAGTATAATCATCTGG |
| *SfruOR57* | TCAACCAGACGACGATGAAG | CGTAACGACAGACAGGCAAC |
| *SfruOR60* | GGGGTTGGACACTGAAGA | AGTTGTGCGAAGGTTGGT |
| *SfruOR62* | TCTTGCTATGGCTCAGTTGG | AACGCTTCTTGAATAAGTTTGC |
| *SfruOR64* | CCTGCTGAGTACCCTGACGC | GAGGAACGCTGTGATGTTGG |
| *SfruOR67a* | CCTTCTTCGCATCCGTTTAT | ATCTGGTCCAGGCTCACATC |
| *SfruOR67c* | CTTACAATGACGCTGCAAAT | TCTTCAACCCTGAGATGACC |
| *SfruOR85c* | CTGAGATGTGGAGGTTGGCTAG | TTTGCTTGGATTCGCTTGTG |
| ***GRs*** |  |  |
| *SfruGR1* | GGTAATCTTCTTCACGACTAC | TCCGAGGTTCACATTCAG |
| *SfruGR2* | GTATCATTATCCGCTCATATCC | ATCCACGATCAGTTCTACAG |
| *SfruGR3* | GGAGTGCTGCCCATTACG | GAACTTGCCTTCCGCTGT |
| *SfruGR4* | TTTCTCCCAATACTAAACAACG | GCTCCTGACATCCGACCT |
| *SfruGR5* | TGCGACAAGTTGACAAGA | CACGACGAGAAACAGGAA |
| *SfruGR6* | CCCGTGCTGTATGATGTG | GCGTCGGAGTAGAGGAGT |
| *SfruGR7* | ATAGCCCTCAACCACATC | AACATCATCCGTTCCTTT |
| *SfruGR8* | CTTCTTAGACGCAATGGA | GCTGTTATGCTTCGGTGT |
| *SfruGR9* | AGGCGGACAATACTCTTTA | AGTACGACGAACCACAGC |
| ***IRs*** |  |  |
| *SfruIR8a* | GACCGCACCCTATACGAG | GTGAGCACCAGGTTCCAG |
| *SfruIR21a* | AGGTTACGCTGGTCACAG | ATTAGGCTCACGAGGTTC |
| *SfruIR25a* | ATAAGGGACGAGCAAGCA | TCAGCCATGACAGACAGC |
| *SfruIR60a* | GTTATCGCCGTACCTCAA | TCCATTACACAGCCATTCA |
| *SfruIR64a* | GAATGGCGTAAGCAATATGT | GAGGAAGGAATAGCGTTAGT |
| *SfruIR75a* | GCCCAGGATAGTGAACCA | TCACCATGCCATCGAAAC |
| *SfruIR75d* | GGACACCACCTACAAGAC | TGATTATGTCGTAGCCAGAG |
| *SfruIR75p* | TCATCATAGGAGCAGTTCTG | AGTGGTGAGTTGAGGAGAT |
| *SfruIR76b* | TGCGGAACAGTGATCTTA | AAACAGCGAGTCGTATGG |
| *SfruIR93a* | ATGGATTCAGAGGGAAGG | GCATCATATCAGTGGTCGT |
| *SfruActin* | TACTCCTAAGCCTGTTGATG | TTATGTCATGGTGCCGAAT |
