# Supplementary material for: A mouthpart transcriptome for Spodoptera frugiperda adults: identification of candidate chemoreceptors and investigation of expression patterns
Source: Front Physiol. 2023 Apr 25;14:1193085. doi: 10.3389/fphys.2023.1193085 (PMC10166800; doi:10.3389/fphys.2023.1193085)
Supplement: Supplementary file 1 [file DataSheet1.ZIP › Supplementary data/Table S3 (summary of assembly) revised.docx]

Table S3. Summary of the transcriptome assembly of *S. frugiperda* mouthpart.

| Length range (bp) | Transcript number | Unigene number |
| --- | --- | --- |
| 500+ | 108,275 | 34,019 |
| 1000+ | 46,732 | 14,295 |
| Total number | 282,631 | 119,928 |
| Total length | 188,846,354 | 68,151,470 |
| N50 length | 969 | 785 |
| N90 length | 278 | 245 |
| Max length | 29,237 | 29,237 |
| Min length | 201 | 201 |
| Mean length | 668.17 | 568.27 |
