# Supplementary material for: A mouthpart transcriptome for Spodoptera frugiperda adults: identification of candidate chemoreceptors and investigation of expression patterns
Source: Front Physiol. 2023 Apr 25;14:1193085. doi: 10.3389/fphys.2023.1193085 (PMC10166800; doi:10.3389/fphys.2023.1193085)
Supplement: Supplementary file 1 [file DataSheet1.ZIP › Supplementary data/Table S4 (Blast).docx]

**Table S4.** Candidate chemoreceptors in the mouthpart of *S. frugiperda*.

| **Name** | **ID** | **ORF**  **(aa)** | **BLASTx best hit**  **(GenBank accession/name/species)** | **Full length** | **Identity**  **(%)** | **E-value** |
| --- | --- | --- | --- | --- | --- | --- |
| **ORs** |  |  |  |  |  |  |
| SfruOR1 | DN36107_c2_g3 | 449 | QYF65492.1\| odorant receptor 1 [*Spodoptera litura*] | Yes | 95 | 0.0 |
| SfruOR2 | DN33707_c2_g4 | 402 | QNS36196.1\| olfactory receptor 2 [*Mythimna separata*] | Yes | 88 | 0.0 |
| SfruOR3 | DN36417_c0_g1 | 379 | XP_022827581.1\| odorant receptor 30a-like [*Spodoptera litura*] | Yes | 89 | 0.0 |
| SfruOR6 | DN40082_c0_g1 | 432 | ACL81183.1\| putative olfactory receptor 6 [*Spodoptera littoralis*] | Yes | 88 | 0.0 |
| SfruOR12b | DN36647_c0_g1 | 388 | YF65502.1\| odorant receptor 12 [*Spodoptera litura*] | No | 94 | 0.0 |
| SfruOR17 | DN33677_c0_g2 | 255 | QYF65507.1\| odorant receptor 17 [*Spodoptera litura*] | No | 95 | 7e-178 |
| SfruOR18 | DN37624_c0_g1 | 398 | AGA16498.1\| olfactory receptor 18 [*Spodoptera litura*] | Yes | 91 | 0.0 |
| SfruOR23 | DN32172_c0_g1 | 412 | AZL96419.1 \|odorant receptor 23 [Helicoverpa assulta] | Yes | 81 | 0.0 |
| SfruOR25 | DN39992_c0_g1 | 416 | QNS36222.1\| olfactory receptor 25 [*Mythimna separata*] | Yes | 75 | 1e-174 |
| SfruOR27 | DN33283_c0_g1 | 429 | QYF65517.1\| odorant receptor 27 [*Spodoptera litura*] | Yes | 98 | 0.0 |
| SfruOR30 | DN44157_c8_g3 | 387 | XP_050551229.1\| Or1-like [*Spodoptera frugiperda*] | Yes | 92 | 0.0 |
| SfruOR32 | DN41457_c1_g1 | 397 | QEY02574.1\| odorant receptor 5 [*Spodoptera littoralis*] | Yes | 72 | 0.0 |
| SfruOR34 | DN32907_c0_g1 | 409 | QYF65523.1\| odorant receptor 34 [*Spodoptera litura*] | Yes | 93 | 0.0 |
| SfruOR35 | DN38262_c0_g2 | 453 | XP_022831643.1\| odorant receptor 85c-like [*Spodoptera litura*] | Yes | 95 | 0.0 |
| SfruOR38 | DN38347_c0_g1 | 418 | QNS36229.1\| olfactory receptor 38 [*Mythimna separata*] | Yes | 86 | 1e-164 |
| SfruOR45 | DN39388_c1_g1 | 429 | XP_022825109.1\| odorant receptor 13a-like isoform X1 [*Spodoptera litura*] | Yes | 94 | 0.0 |
| SfruOR46 | DN33868_c1_g1 | 391 | XP_022817447.1\| odorant receptor 46a-like [*Spodoptera litura*] | Yes | 97 | 0.0 |
| SfruOR49a | DN36640_c0_g1 | 403 | XP_035447756.2\| odorant receptor 49a-like [*Spodoptera frugiperda*] | Yes | 100 | 0.0 |
| SfruOR49b | DN32423_c0_g1 | 393 | XP_022834239.1\| odorant receptor 49b-like [*Spodoptera litura*] | Yes | 96 | 0.0 |
| SfruOR50 | DN34812_c1_g1 | 404 | QNS36220.1\| olfactory receptor [*Mythimna separata*] | Yes | 72 | 0.0 |
| SfruOR53 | DN34590_c0_g2 | 404 | QYF65542.1\| odorant receptor 53 [*Spodoptera litura*] | Yes | 93 | 0.0 |
| SfruOR57 | DN41551_c0_g1 | 397 | QYF65545.1\| odorant receptor 57 [*Spodoptera litura*] | Yes | 89 | 0.0 |
| SfruOR60 | DN36386_c0_g1 | 392 | ABQ82137.1\| chemosensory receptor 2 [*Spodoptera littoralis*] | Yes | 98 | 0.0 |
| SfruOR62 | DN39074_c3_g2 | 371 | ALM26245.1\| odorant receptor 62 [*Athetis dissimili*s] | Yes | 93 | 0.0 |
| SfruOR64 | DN40969_c1_g1 | 413 | AVF19676.1\| putative odorant receptor [*Peridroma saucia*] | Yes | 78 | 0.0 |
| SfruOR67a | DN39579_c0_g1 | 416 | XP_050562343.1\| odorant receptor 67a-like [*Spodoptera frugiperda*] | Yes | 99 | 0.0 |
| SfruOR67c | DN32912_c2_g1 | 390 | XP_050554729.1\| odorant receptor 67c-like [*Spodoptera frugiperda*] | Yes | 99 | 0.0 |
| SfruOR85c | DN42554_c0_g2 | 393 | XP_050557942.1\| odorant receptor 85c-like [*Spodoptera frugiperda*] | Yes | 99 | 1e-176 |
| SfruORco | DN43518_c2_g2 | 473 | AAW52583.1\| putative chemosensory receptor 2 [*Spodoptera exigua*] | Yes | 99 | 2.4e-258 |
| **GRs** |  |  |  |  |  |  |
| SfruGR1 | DN33391_c0_g2 | 464 | XP_022828173.1\| gustatory and odorant receptor 22 [*Spodoptera litura*] | Yes | 99 | 0.0 |
| SfruGR2 | DN28721_c0_g1 | 433 | XP_035439638.1\| gustatory and odorant receptor 22-like [*Spodoptera frugiperda*] | Yes | 100 | 0.0 |
| SfruGR3 | DN30952_c0_g1 | 475 | XP_022815658.1\| gustatory and odorant receptor 24 [*Spodoptera litura*] | Yes | 100 | 0.0 |
| SfruGR4 | DN31889_c2_g1 | 402 | XP_035430303.1\| gustatory receptor for sugar taste 64a-like [*Spodoptera frugiperda*] | Yes | 100 | 0.0 |
| SfruGR5 | DN30906_c0_g1 | 476 | XP_035430301.2\| gustatory receptor for sugar taste 64e-like [*Spodoptera frugiperda*] | Yes | 100 | 0.0 |
| SfruGR6 | DN34523_c0_g1 | 447 | QYF65556.1\| gustatory receptor 6 [*Spodoptera litura*] | No | 95 | 0.0 |
| SfruGR7 | DN37736_c0_g1 | 429 | XP_035429284.2\| gustatory receptor for sugar taste 64a-like [*Spodoptera frugiperda*] | Yes | 100 | 0.0 |
| SfruGR8 | DN33017_c0_g1 | 431 | XP_035429285.2\| gustatory receptor for sugar taste 64a-like [*Spodoptera frugiperda*] | Yes | 100 | 0.0 |
| SfruGR9 | DN40413_c0_g1 | 488 | XP_035448630.1\| gustatory receptor for sugar taste 43a [*Spodoptera frugiperda*] | Yes | 100 | 0.0 |
| **IRs** |  |  |  |  |  |  |
| SfruIR8a | DN34445_c1_g1 | 898 | QYF65596.1\|ionotropic receptor 8a [*Spodoptera litura*] | Yes | 96 | 0.0 |
| SfruIR21a | DN30270_c2_g1 | 852 | XP_035448875.2\| ionotropic receptor 21a [*Spodoptera frugiperda*] | Yes | 100 | 0.0 |
| SfruIR25a | DN42711_c2_g3 | 918 | XP_022828195.1\| ionotropic receptor 25a [*Spodoptera litura*] | Yes | 99 | 0.0 |
| SfruIR60a | DN43581_c1_g1 | 660 | QHB15321.1\| ionotropic receptor 60a [*Peridroma saucia*] | Yes | 80 | 0.0 |
| SfruIR64a | DN38775_c1_g1 | 603 | ARB05666.1\| ionization receptor 64a [*Mythimna separata*] | Yes | 80 | 0.0 |
| SfruIR75a | DN39469_c0_g1 | 631 | XP_035459405.2\| ionotropic receptor 75a [*Spodoptera frugiperda*] | Yes | 100 | 0.0 |
| SfruIR75d | DN36075_c1_g1 | 593 | ADR64683.1\| chemosensory ionotropic receptor IR75d [*Spodoptera littoralis*] | Yes | 95 | 0.0 |
| SfruIR75p | DN38967_c0_g1 | 624 | XP_022816386.1\| glutamate receptor 1-like [*Spodoptera litura*] | Yes | 90 | 0.0 |
| SfruIR76b | DN42735_c2_g1 | 542 | ADR64687.1 \|putative chemosensory ionotropic receptor IR76b [*Spodoptera littoralis*] | Yes | 95 | 0.0 |
| SfruIR93a | DN42070_c0_g1 | 708 | XP_050563436.1\| ionotropic receptor 93a [*Spodoptera frugiperda*] | Yes | 100 | 0.0 |
